# New Binding Site Conformations of the Dengue Virus NS3 Protease Accessed by Molecular Dynamics Simulation

**DOI:** 10.1371/journal.pone.0072402

**Published:** 2013-08-21

**Authors:** Hugo de Almeida, Izabela M. D. Bastos, Bergmann M. Ribeiro, Bernard Maigret, Jaime M. Santana

**Affiliations:** 1 Laboratório de Interação Patógeno-Hospedeiro, Departamento de Biologia Celular, Universidade de Brasília, Brasília, Brasília, Brazil; 2 Laboratório de Microscopia Eletrônica e Virologia, Departamento de Biologia Celular, Universidade de Brasília, Brasília, Brasília, Brazil; 3 LORIA/UMR 7503, Équipe-projet Orpailleur, Nancy Université, Campus Scientifique, Vandœuvre-lès-Nancy, France; University of Akron, United States of America

## Abstract

Dengue fever is caused by four distinct serotypes of the *dengue virus* (DENV1-4), and is estimated to affect over 500 million people every year. Presently, there are no vaccines or antiviral treatments for this disease. Among the possible targets to fight dengue fever is the viral NS3 protease (NS3_PRO_), which is in part responsible for viral processing and replication. It is now widely recognized that virtual screening campaigns should consider the flexibility of target protein by using multiple active conformational states. The flexibility of the DENV NS3_PRO_ could explain the relatively low success of previous virtual screening studies. In this first work, we explore the DENV NS3_PRO_ conformational states obtained from molecular dynamics (MD) simulations to take into account protease flexibility during the virtual screening/docking process. To do so, we built a full NS3_PRO_ model by multiple template homology modeling. The model comprised the NS2B cofactor (essential to the NS3_PRO_ activation), a glycine flexible link and the proteolytic domain. MD simulations had the purpose to sample, as closely as possible, the ligand binding site conformational landscape prior to inhibitor binding. The obtained conformational MD sample was clustered into four families that, together with principal component analysis of the trajectory, demonstrated protein flexibility. These results allowed the description of multiple binding modes for the Bz-Nle-Lys–Arg–Arg-H inhibitor, as verified by binding plots and pair interaction analysis. This study allowed us to tackle protein flexibility in our virtual screening campaign against the *dengue virus* NS3 protease.

## Introduction

Dengue fever (DF) is an infectious disease caused by four distinct serotypes of *Dengue virus* (DENV1-4) transmitted by *Aedes* spp. Milder manifestations of the disease may include fever, rash, headaches, joint and muscle pain, fatigue and vomiting. Re-infection by different serotypes, however, may cause much more significant clinical conditions, like Dengue Hemorrhagic Fever (DHF) and Dengue Shock Syndrome (DSS) [1,2] which can cause death.

**Figure 1:**
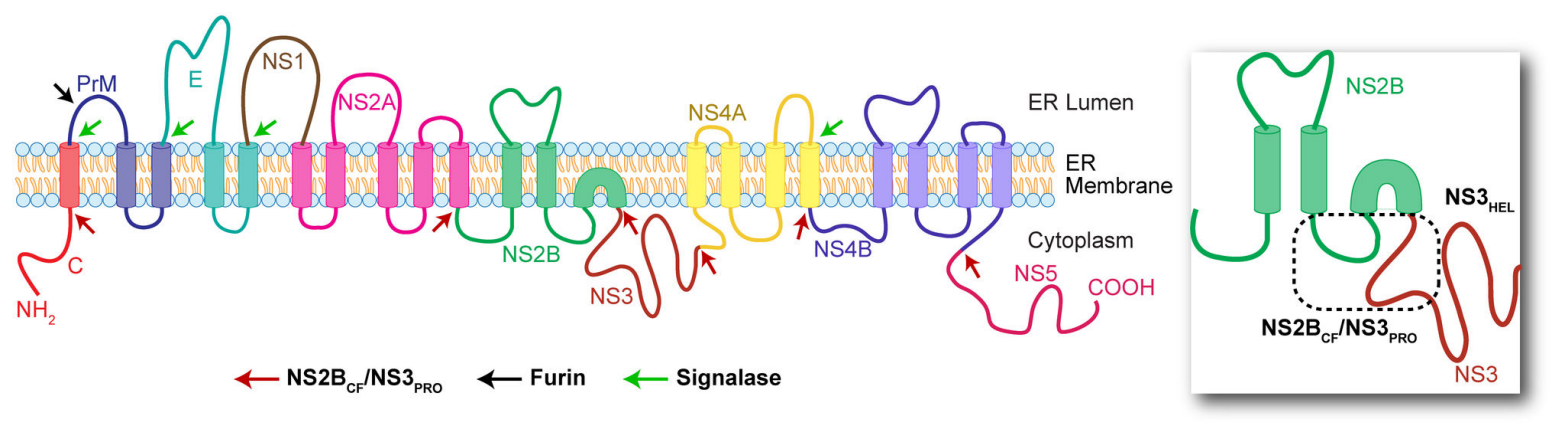
Predicted membrane topology of the Dengue virus polyprotein and its cleavage sites. The polyprotein is composed of three structural subunits: capsid (C), precursor of membrane protein (PrM) and envelope (E), as well as seven nonstructural (NS) subunits: NS1, NS2A, NS2B, NS3, NS4A, NS4B and NS5. The NS3 is a multifunctional protein composed of a helicase domain (NS3_HEL_, see detailed box) and a protease (NS3_PRO_, also observable in the detailed box) domain, which in turn needs the hydrophilic loop of NS2B (NS2B_CF_, marked in green) as a cofactor to be fully active. Red arrows indicate sites processed by the viral NS2B/NS3 protease. Based on Figure 1 from Umareddy et al., 2007 [12], for illustrative purposes only.

DF is estimated to affect over 500 million people every year [3] and has recently been ranked as the most common cause of febrile illness in travelers, surpassing malaria and gastrointestinal infections [4]. Together with the ongoing expansion of mosquito habitats [5] either due to the recent climate changes and to the urbanization of developing countries [6], this fact has drawn the attention of sanitary and health centers around the globe. Two autochthonous cases in Europe [7] and recent outbreaks in southern USA [8] have shown that dengue is no longer exclusively a problem for tropical developing countries. Despite its high incidence, severity and economic burden, there are currently no antiviral treatments nor vaccines for DF. The development of an efficient anti-DF vaccine faces the challenge to provide protection for all four serotypes at once [9], otherwise it may render immunized individuals more susceptible to DHF [10]. Regarding the design of antiviral drugs, viral proteases are often proposed as potential therapeutic targets due to their essential task of processing viral polyproteins into their functional unities [11]. Concerning *Dengue virus* (DENV) and its close relative West Nile virus (WNV), this role is assigned to the multi-domain nonstructural protein 3 (NS 3). NS3 is composed by a protease (NS3_PRO_) and a helicase (NS3_HEL_) domain, with the former being responsible for processing the polyprotein in specific sites (Figure 1, adapted from Umareddy et al, 2007 [12]). The NS3_PRO_ domain (EC 3.4.21.91) belongs to the S7 family of serine proteases, and needs a cofactor, the hydrophilic loop from NS2B (NS2B_CF_) in the case of DENV and WNV, to become fully active [13,14].

This protease has already been recognized as a valuable target for the design of new antiviral inhibitors against *Dengue virus* [15,16], and, therefore, designing potent inhibitors against DENV NS3_PRO_ is an active research line in the fight against DF [17–22]. The use of computational drug-design approaches would be useful here to improve the discovery of putative hits and to help obtain new leads [23–31]. However, previous virtual screening campaigns have fallen short in the identification of new inhibitors, since none was able to find small organic compounds in the submicromolar inhibitory range. It is now widely accepted that protein flexibility is an important factor to be taken into account to ensure the success of virtual screening campaigns [32]. The flexibility of DENV NS3 protease is evidenced in current crystallographic structures by the lack of atomic coordinates for many residues, the difficulty in resolving an inhibitor-enzyme structure, the variable positioning of the cofactor in respect to the protease, and those of several loops [33–36]. This scenario could explain the relatively low success of previous virtual screening attempts against this target. In fact, the flexibility of DENV NS2B/NS3 protease has already been proposed, but not proved, to explain the poor results of current drug design campaigns [21].

To address this issue, we performed molecular dynamics (MD) simulations towards an ensemble docking campaign. Ensemble docking is now widely recognized as an efficient strategy for incorporating protein flexibility in virtual screening campaigns [37–39], especially when the ensembles are extracted from molecular dynamics simulations [40,41]. MD simulations of DENV NS2B/NS3_PRO_ described in the present work intended to sample as closely as possible the ligand binding region landscape prior to inhibitor binding. The obtained conformational MD sample was therefore clustered into families diverging at least 4.5 Å apart. Thus, a limited protein conformational set, shown to contain structures sufficiently different at their binding sites (Figure S1), was chosen to be employed in a subsequent ensemble docking simulation using focused chemical libraries.

## Materials and Methods

### Homology Modeling

We obtained most of our results before the availability of the 3D structure of the Dengue virus 3 NS2B/NS3_PRO_ in complex with its inhibitors [36]. However, as all of the accessible crystallographic structures, including the novel ones, presented missing residues, we needed to model their related segments. In addition to that, we considered three main factors during the construction of our 3D model. Firstly, it had to relate to the full-length sequence of the expression construct we have (DENV 1 NS2B-Gly_[4]_-Ser–Gly_[4]_-NS3_PRO_). Secondly, it had to represent the catalytically active “closed” conformation. Lastly, it needed to be able to take into account the possible influence of N- and C-terminus domains in the NS3 protease active site conformational changes and/or accessibility. This led us to build a 3D model that included the hydrophilic NS2B cofactor region, a glycine flexible link, and the NS3 proteolytic domain in complex with the NDL inhibitor.

To obtain this DENV NS2B_CF_-Gly-NS3_PRO_ model with as much structural information as possible, we performed an alignment among all *Dengue virus* and *West Nile virus* protease 3D structures available in the protein database (PDB) at the time (Table 1). This allowed us to check the completeness of the PDB structures compared to the sequences deposited by the same authors. From this analysis, we were able to gather information to build three different models of our system: Two preliminary models based either solely on the WNV or solely on the DENV PDB structures and a third one mixing data from both DENV and WNV.

**Table 1:** Crystallographic structures used as templates in the homology modeling process and their construct data information.

Virus	PDB id	Structural information	Ref.
DENV2	2FOM	NS2B_CF_-Gly-NS3_PRO_	[33]
DENV4	2VBC	NS2B_18_-Gly-NS3_FULL_^a^	[34]
DENV4	2WHX	NS2B_18_-Gly-NS3_FULL_^a^	[52]
DENV1	3L6P	NS2B_CF_-Gly-NS3_PROΔ10_^b^	[35]
WNV	2FP7	NS2B_CF_-Gly-NS3_PRO_ + NDL	[33]
WNV	2IJO	NS2B_CF_-Gly-NS3_PRO_ + BPTI	[63]
WNV	3E90	NS2B_CF_-Gly-NS3_PRO_ + NKK	[69]

a Instead of using the full hydrophilic loop from NS2B, a short sequence of 18 residues was used, only to provide solubility to the expressed complex.

b The construct has a 10 residues deletion in the N-terminus of NS3_PRO_, in order to facilitate crystallization.

NDL: Bz-Nle-Lys–Arg–Arg-H; BPTI: bovine pancreatic trypsin inhibitor; NKK: Naph-Lys-Lys-Arg-H

For each model, we prepared the PDB templates by removing unused chains, ligands, water and ions. The cleaned structures were aligned using the Discovery Studio Visualizer 3.1 (Accelrys, Inc) based on the sequence alignment of their conserved residues. Prior to submission to the homology software MODELLER [42], the sequence alignment obtained previously was edited to ensure certain features would be found in the resulting model, i.e., the N-terminus region (including NS2B_CF_) from WNV structures and C-terminus from DENV structures in the final model. For each set of templates (WNV only, DENV only or WNV/DENV), five homology models were generated, each with three loop refinements, giving us 45 models. The three lowest energy models (based on each set of templates) were chosen and verified by PROCHECK [43] to ensure their structural quality. We next visually compared these models and retained the one with features from both WNV and DENV structures, as it contained less disordered coils and loops.

Finally, we transferred the atomic coordinates of the NS2B cofactor and those of the NDL inhibitor (Bz-Nle-Lys–Arg–Arg-H) from the 2FP7 PDB template into the final NS3_PRO_ model as obtained above. After arranging the cofactor in place, we built the glycine linker with the homology module of the INSIGHT II package (Accelrys Inc). The inhibitor was deliberately left covalently unbound to simulate conformational changes in the active site prior the formation of the protease/substrate intermediate. Using the AutoPSF Generation Plugin provided with VMD [44], missing hydrogens were added according to their predicted protonation state at pH 7.0 and histidine residues were assigned to their δ-protonated state.

### Molecular Dynamics Simulations

The final model of the complex between the NDL ligand and the NS2B_CF_-Gly-NS3_PRO_ target obtained above was submitted to MD simulations. The complex was firstly solvated with TIP3P explicit water molecules in a box with at least 15 Å apart from any point in the protein, ending up with a box of 80 x 100 x 85 Å. Eight Na^+^ ions were added to ensure electrostatic neutrality. Missing parameters for the ligand were added using the CHARMM22 force field [45]. The NAMD software [46] was used to simulate the dynamic behavior of the complex using the parm.prm parameter file from CHARMM22 in periodic boundary conditions. Long-range electrostatics was evaluated using the particle-mesh Ewald approach [47]. Simulations were carried out in the NPT ensemble using Langevin dynamics and piston to fix temperatures (300 K) and pressure (1 atm). Hydrogen-heavy atom bonds were constrained to their equilibrium values with the SHAKE algorithm [48].

The system was first energy minimized (6400 conjugate gradient steps) and then equilibrated (500 ps) before recording the trajectories. All MD trajectory frames were recorded at 1 ps intervals, for a total of 30 ns simulation.

### Clustering

Once the MD simulations were complete, all frames were aligned by taking into account only heavy atoms in the core region of the NS3 protease alone (residue range from 20 to 168). This was done in order to focus the protein conformational analysis on the binding site region only. The RMSDs were calculated by using the appropriate VMD plugin and the corresponding data were exported as an ASCII matrix by the VMD Multiplot module. For clustering, a previously developed in-house Tcl script was used with a cutoff of 4.5 Å. This script builds a 2D RMSD matrix for identifying conformational families within a given RMSD range.

### Pocket detection and volume analysis

Pockets were detected using METAPOCKET [49], an algorithm using several reliable pocket detection tools to combine their results and improve sensibility. Changes in pocket volume and surface (pocket descriptors) were monitored with MD Pocket [50], a software capable of analyzing topological changes in cavities during MD simulations.

### Analysis software

All molecular representations as well as several other analyses (such as distances between catalytic residues through the trajectory and secondary structure maintenance over time, for example) were performed by VMD. All graphics (RMSD, pair interactions, mobility plots, pocket volume evolution, etc) were plotted using GraphPad Prism 5 (GraphPad Software, Inc). Two-dimensional binding plots from docking results were generated with Discovery Studio Visualizer 3.1 (Accelrys, Inc).

## Results

### Building the 3D model

#### PDB Templates Sequence alignment

**Figure 2:**
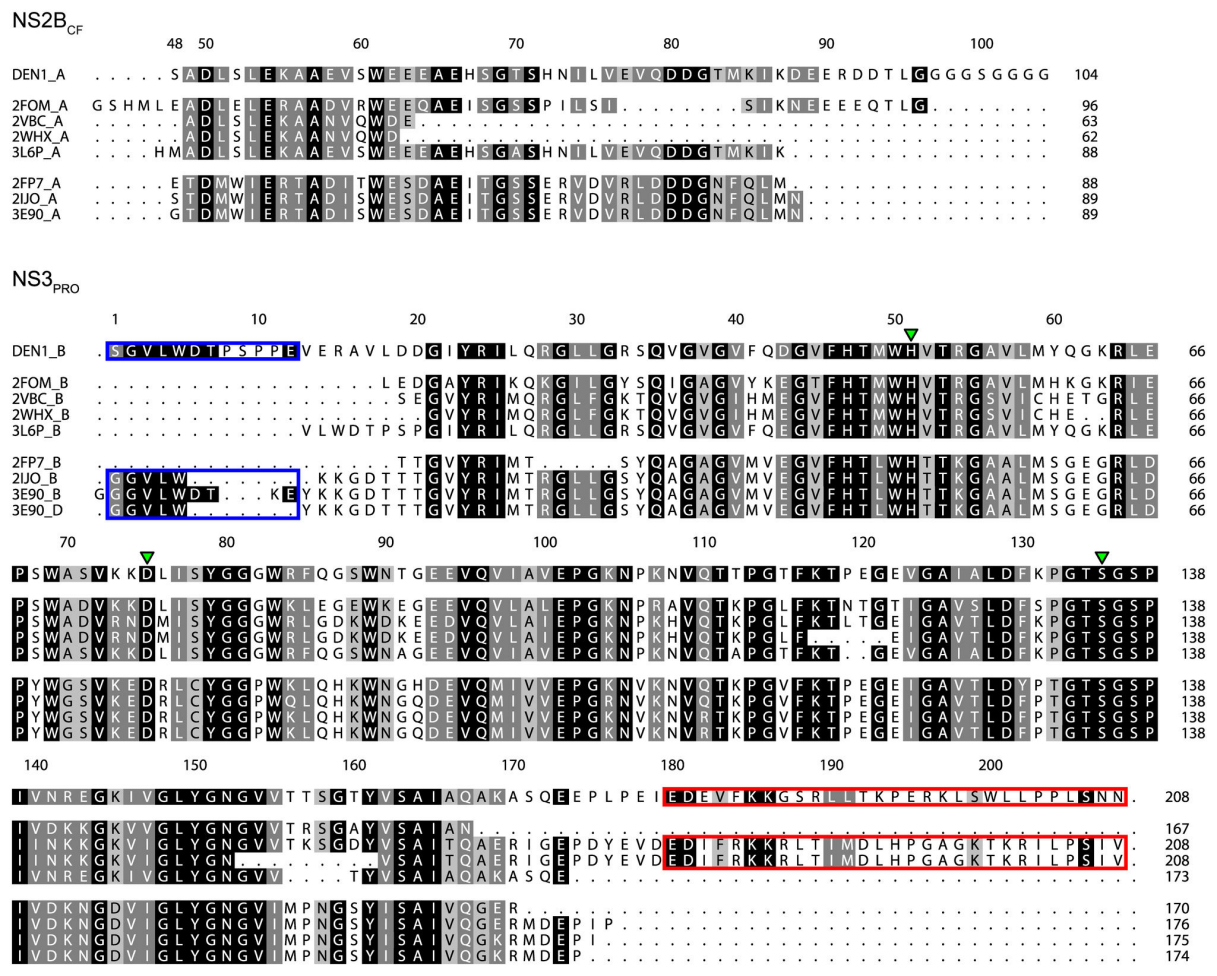
Dengue and West Nile virus NS2B_CF_-Gly-NS3_PRO_ multiple sequence alignment extracted from the protein databank files. This alignment allowed us to verify the completeness of each deposed structure, as well as to fill structural gaps, direct N- (blue box) and C-terminus (red box) regions and the cofactor itself. Green triangles indicate the catalytic triad (HIS_51_, ASP_75_ and SER_135_); sequences are numbered accordingly to the full constructs and shaded based on sequence similarities (black for identical, dark gray for strongly similar and soft gray for weakly similar residues).

To build a homology model based on the available homologous PDB structures, we first performed a manual sequence alignment analysis of these templates (Figure 2). This alignment shows highly conserved residues between the DENV and WNV NS3_PRO_ proteins, with an overall identity of 40.6% (64.2% similarity). One block of 5 residues, numbered 59 to 63, seems to vary according to the DENV serotypes. A lack of structural information at C- and N-terminal regions was found, probably due to their high flexibility, and hence little information from those residues could be utilized for molecular modeling. NS3_PRO_ N-terminus data were more likely to be found in West Nile virus structures (Figure 2, blue box), while those of C-terminus were better provided by full (with both protease and helicase domains) NS3 Dengue virus structures (Figure 2, red box).

#### Templates structural alignment

**Figure 3:**
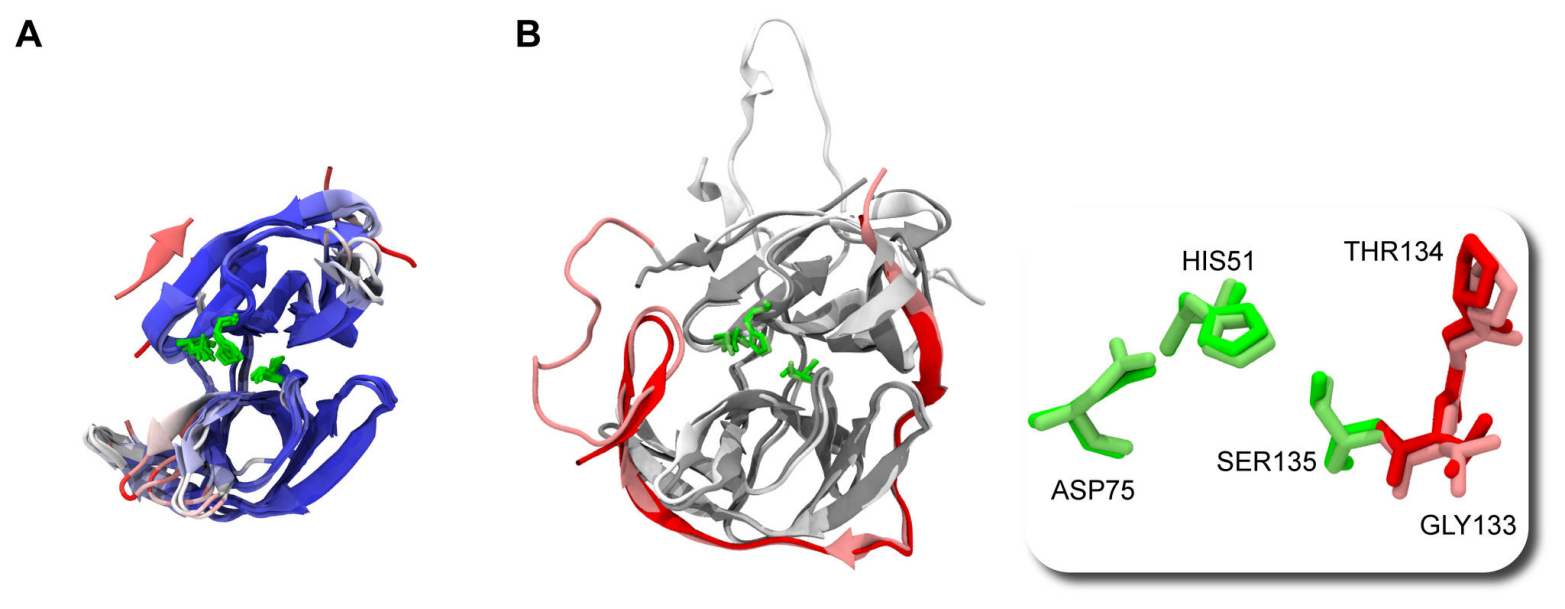
Structural alignments between (A) core region from all templates used in homology modeling, and (B) obtained model and recently available ligand bound DENV NS3_PRO_ structure (PDB id: 3U1I) [36]. In (A), residues were colored based on their Qres factor, obtained after a STAMP structure alignment performed with the VMD Multiseq plugin [68]. Color ranges from dark blue (highly conserved positions) to red (not conserved at all). In (B), crystallographic structure was colored with gray (NS3_PRO_) and red (NS2B_CF_); homology model was colored in lighter shades – white (NS3_PRO_) and pink (NS2B_CF_). In addition to the highly conserved active site residues position, the oxyanion hole was also preserved in this model (boxed detail).

Regarding the PDB structures, while much has been said about different conformations related to distinct serotypes [35] and about the influence of ligands and co-factors [33], the protein core structure is well conserved even when compared between DENV and WNV (Figure 3A). The RMSD values for each template main chain atoms compared to the reference protein 2FOM are given in table 2, showing that active site residues position is similar among them, irrespectively of whether the cofactor was in the open or closed conformation.

**Table 2:** Homology modeling templates and their catalytic triad main-chain atom RMSD to the reference protein 2FOM, based on the 126 conserved residues from the NS3_PRO_ domain.

PDB id	RMSD (Å)			
	Protein	HIS_51_	ASP_75_	SER_135_
2VBC	1.3984	0.448	0.796	0.815
2WHX	1.4121	0.503	0.851	0.764
3L6P	1.3000	0.263	0.323	0.468
2FP7	1.3620	0.412	0.833	0.717
2IJO	1.3330	0.504	0.996	0.784
3E90	1.2913	0.543	0.846	0.696

The situation is different for the C-terminal turn (Figure 3A, residues 151 to 167, marked in shades of red, bottom left corner) that adopted several spatial conformations among DENV structures, but not among WNV, indicating that this conformation may be stabilized by ligand binding.

#### Homology Modeling

Our strategy was initially to build a robust model of the NS3_PRO_ domain and next to add the NS2B_CF_, the ligand and the linker pieces to achieve the active conformation of the NS2B_CF_-Gly-NS3_PRO_ model, which was later used in the MD simulations. For that purpose, we first built two preliminary NS3_PRO_ models based exclusively either on the DENV or on the WNV structures. Besides belonging to different species, the main difference observed was the absence (DENV) or the presence (WNV) of ligands. Among the five models generated by MODELLER from each template set, those with the lowest energies were analyzed. Overall, the models did not differ much at the protein core, whereas, given the divergence on the amount of information between templates, N- and C-terminus were quite distinct. For the model based on the DENV structures, the C-terminal end was arranged more concisely in an alpha-helix shape, which was not observed in the model based on WNV NS3_PRO_. On the other hand, the WNV-based model had its N-terminal end projected into a beta strand that contributes to one of the beta-sheets found in the protein (data not shown).

The superposition of these two 3D models based exclusively on the WNV or DENV NS3_PRO_ provided different features that were considered to build a third NS3_PRO_ model. In this last model, the protein core was modeled as the conserved consensus of all template structures, while the N-terminal region came from WNV proteases and the C-terminal end from the DENV proteases. After visually comparing the structures of the three models, we finally retained the third one, which contained less disordered coils and loops, to be used in the next building steps to obtain the final NS2B_CF_-Gly-NS3_PRO_ model.

After being verified by PROCHECK, the complete NS2B_CF_-Gly-NS3_PRO_ model was found to be of acceptable accuracy (over 96% residues in allowed regions) and was chosen as definitive to carry on further studies (File S1). Figure 3B shows the structural alignment of the finished homology model with the newly available ligand-bound DENV structure (PDB id: 3U1I) [36]. The alignment has a C_α_ atom RMSD of 0.9 Å for 195 atoms, which gives consistency to our built model. Additionally, as pointed by Knehans et al. [24], the oxyanion hole was also present in our model (Figure 3B, boxed detail), even though we did not used restraints during homology modeling. Those features, together with our model completeness, including both inhibitor and cofactor in the active conformations, as well as the gap-filling loop grafts directed by structural evidences found both in DENV and in WNV structures, made our NS2B_CF_-Gly-NS3_PRO_ model a good starting point for MD simulations and the structure-based virtual screening campaign.

### Protein model overall stability and flexibility

#### Molecular dynamics

**Figure 4:**
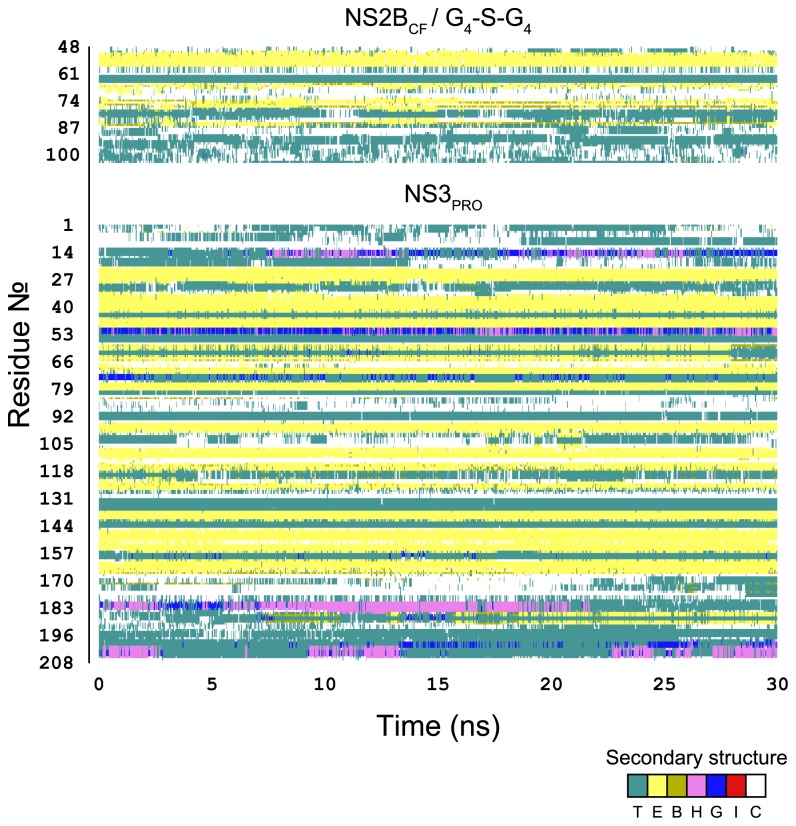
Secondary structure timeline analysis as computed by the timeline plugin contained in VMD. In the graphic, the β-sheet components turn (T) and extended conformation (E) are represented in teal and yellow respectively; isolated bridges are in dark yellow; degrees of helix are in pink (α-helix), blue (3-10 helix) and red (π-helix); random coils are in white.

The timeline analysis of the secondary structure pieces during the 30 ns of MD simulations (Figure 4) showed that protein domains were stable during the trajectory, especially concerning extended conformations (in yellow). Turns and coils (teal and white, respectively), including the polyglycine linker (NS2B residues 95 to 104), constantly alternated between those two types of conformations.

**Figure 5:**
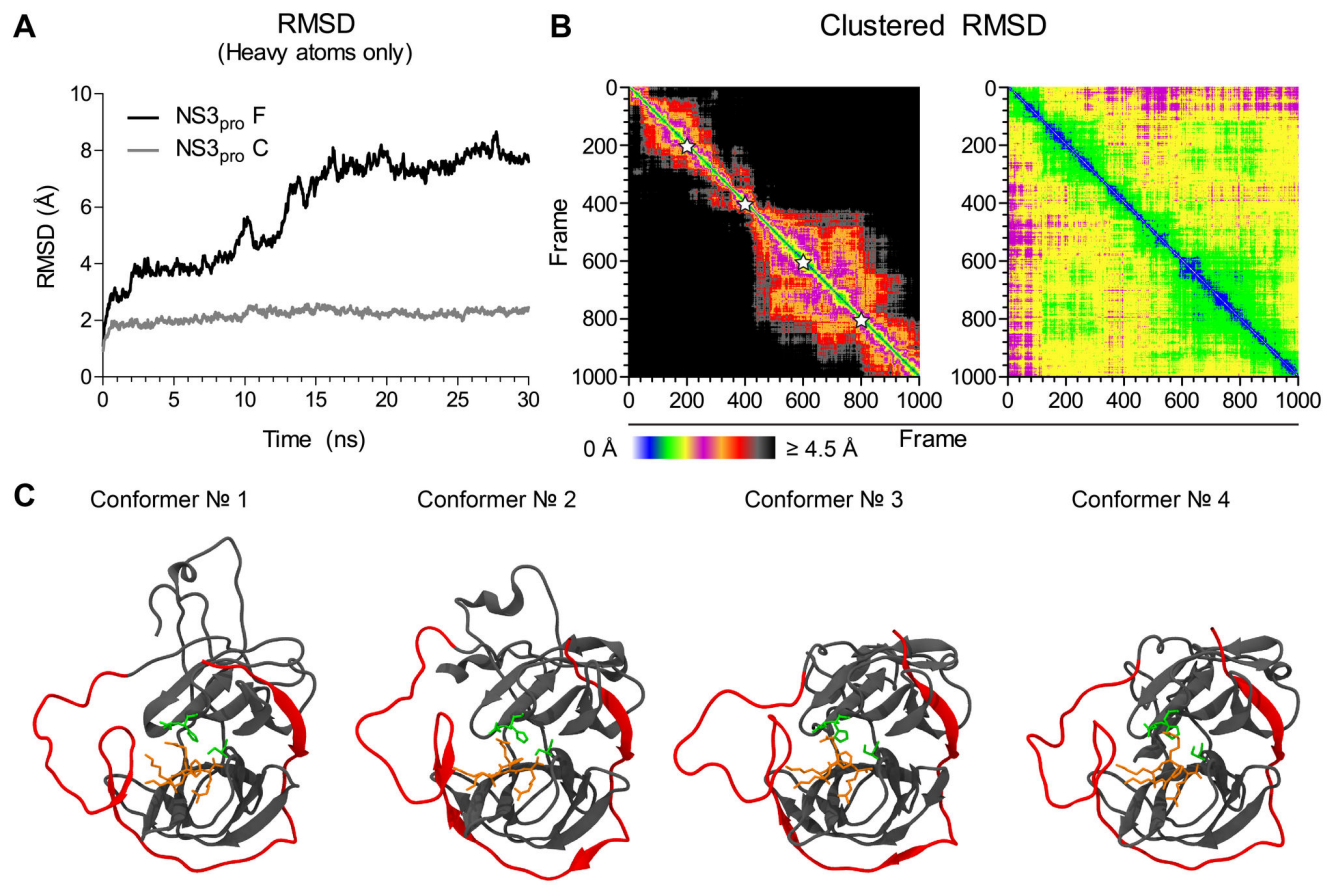
Single- and two-dimensional RMSD plots from the MD simulation trajectory, and their representative conformers. In (A), RMSD values from the whole construct (NS3_PRO_ F, black line) were compared with RMSD values from the protein core only (NS3_PRO_ C, gray line). In (B), a two-dimensional RMSD plot was utilized to provide information about conformational families during trajectory. Color ranges from black (as out of the cut-out distance) to white (as in identical structures), with intermediate values depicted in the color scale. White stars mark the representative frames. (C) Snapshots of representative conformations of each family extracted from the clustered (two-dimensional) RMSD analysis (NS2B_CF_ and linker in red, NS3_PRO_ in gray, active site residues in green and NDL in orange).

After the MD simulation run, both the ligand and the cofactor remained closely bound to the NS3_PRO_ domain (Figure S2). Each frame collected from the trajectory file was then aligned to the first one based on their heavy atoms. RMSD analysis shows a 4-stages curve, with an increasing value in the first 2 ns, after which the model stabilizes for the next 5 ns, followed by a new increase and final stabilization after 15 ns (Figure 5A, NS3_PRO_ F). However, this stabilization is achieved soon after equilibration (before 1 ns of simulation), and with much lower RMSD values (average RMSD of 2.2 *versus* 5.97 Å), when we analyze the protein core alone (residues 20 to 168, Figure 5A, NS3_PRO_ C). As the secondary structure modifications during MD simulations could not account for such a high deviation when analyzing the whole model, we supposed that this was rather due to domain flexibility, which in turn could regulate the access to the binding site.

As it would be impossible to explore each frame separately in vHTS studies, we decided to use a two-dimensional plot clustering technique capable of investigating the structural relatedness of different parts of a MD trajectory (Figure 5B). By doing so, 4 distinct conformational families were detected when analyzing the whole sample, but only two (and with a high degree of relationship) could be detected while analyzing the protein core alone. By choosing the most conserved spots in the two-dimensional RMSD plot, representatives of each conformational family (snapshots corresponding to frames 200, 400, 600 and 800, Figure 5C) were selected for conformational analysis of the binding site. As the last cluster (frames 800 to 1000) contained several conformations related to the previous one (frames 400 to 800), we picked up the frame 800, instead of 900, as an averaged representative of the last family of conformations.

#### Principal component analysis (PCA)

**Figure 6:**
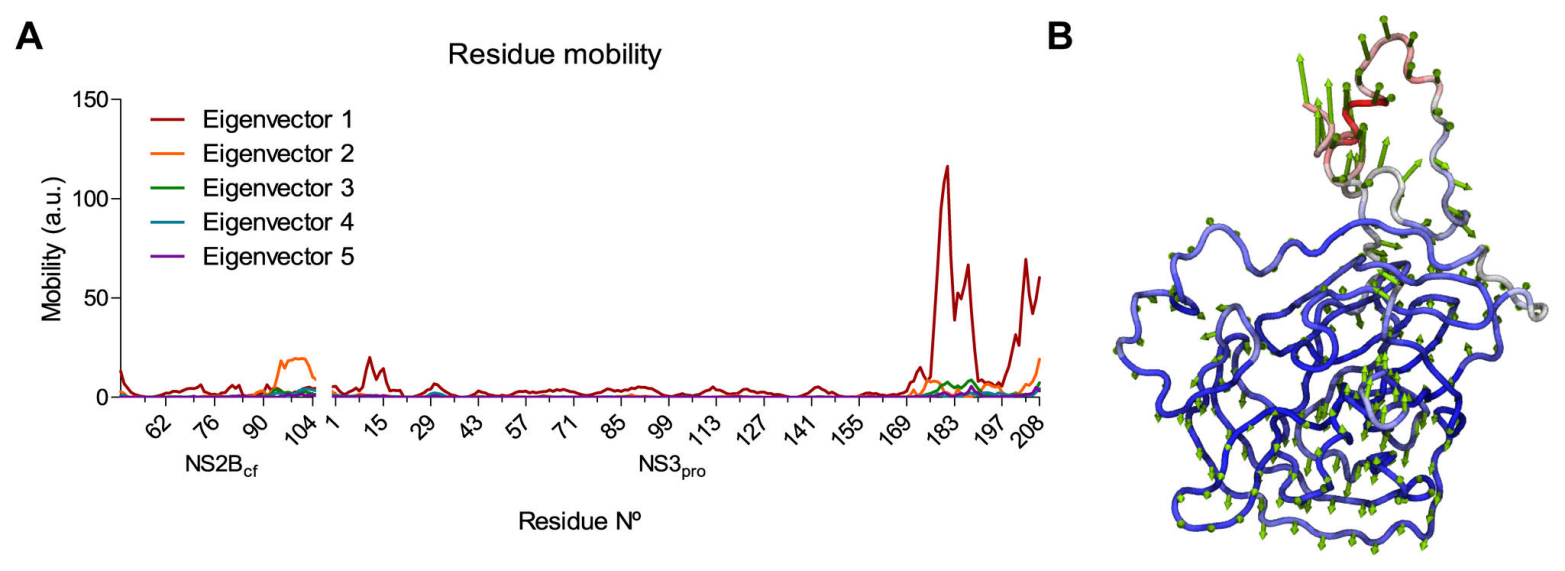
Individual residue mobility in principal component analysis. (A) The greatest eigenvector represents NS3_PRO_ C-terminal domain shifting, and the second eigenvector represents NS2B_CF_ C-terminal and glycine linker shifts. (B) Normal mode visualization from the first eigenvector, also revealing small motions that may be important to binding site plasticity.

To verify which motions could account for RMSD variations, we finally performed a principal component analysis (PCA) from the MD trajectory. Results for the first 5 eigenvectors confirm that the greatest motions were contained within the first two eigenvectors and they indeed came from the NS3_PRO_ C-terminal end (Figure 6). This region has been described as highly flexible, as noticed by differences in the two crystallographic structures for the full (both proteolytic and helicase domains) NS3 construct [34,51,52]. However, this fact does not exclude the possible existence of small but significant motions that may be important for binding site plasticity (Figure 6B). To investigate further this question, we also performed 2D ligand-receptor interaction plots.

### Protein binding site and binding modes flexibilities

#### Binding pocket detection and evolution

The DENV protease has a very shallow pocket [33,53], a fact that led us to employ the METAPOCKET servers. This server utilizes several redundant already validated geometric algorithms to search for proteins cavities. Thus, where one algorithm may fail, another one can correct the results, providing accurate topological information. This chosen pocket detection strategy showed to be in accordance with experimental data, with almost 70% (15/22, Table 3) accuracy. Both S1 and S4 sub-pockets were fully detected, with less favorable results for sub-pockets S2 and S3 with 37.5 and 33.3% accuracy, respectively. It is noteworthy to say that this method was capable of detecting several residues not present in the experimental data. Those residues may constitute useful sub-pockets for designing new competitive molecules.

**Table 3:** Pocket residues as detected by METAPOCKET compared to those found experimentally.

Sub-pocket	NS2B		NS3	
	Detected	Expected	Detected	Expected
S1	-	-	D129	D129
	-	-	F130	F130
	-	-	K131	K131
	-	-	P132	P132
	-	-	G133	G133
	-	-	T134	T134
	-	-	S135	S135
	-	-	Y150	Y150
	-	-	Y161	Y161
S2	-	D80	-	V72
	D81	D81	-	K73
	-	G82	-	K74
	T83	T83	D75	D75
S3	M84	M84	-	-
	-	K85	-	-
	-	I86	-	-
S4	-	-	V154	V154
	-	-	V155	V155
N/A^*^	-	-	Q35	-
	-	-	V36	-
	-	-	W50	-
	-	-	H51	-
	-	-	G151	-
	-	-	N152	-
	-	-	G153	-

Detected pockets were identified by METAPOCKET, as described in Methods. ^*^ Detected residues which may be important to ligand binding, but were not previously reported in literature.

**Figure 7:**
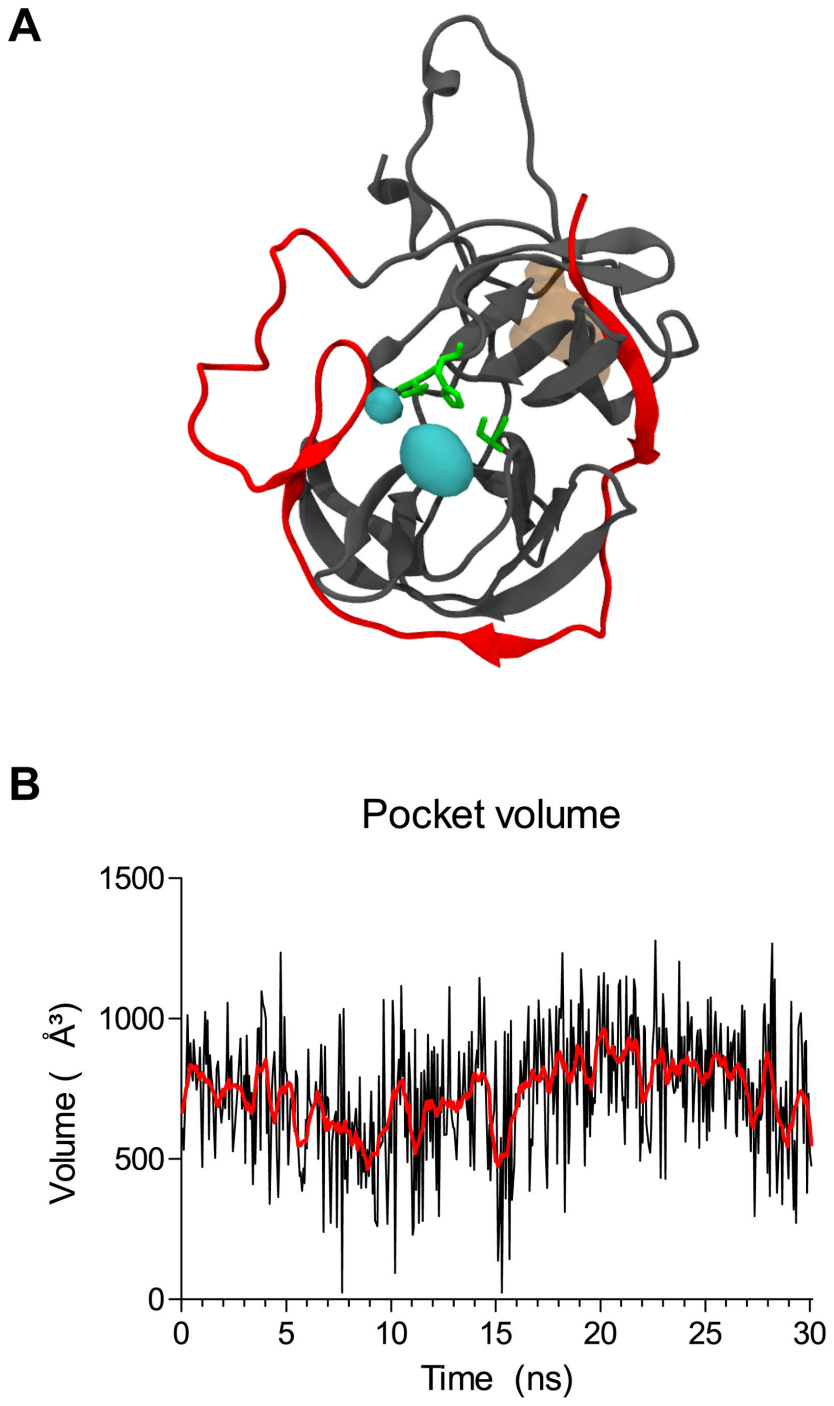
Pocket detection and evolution during MD simulation trajectory. Active site pocket (A) was identified by METAPOCKET and later monitored for changes in volume (B) by MD Pocket. In (A), DENV NS3_PRO_ is represented in gray; NS2B_CF_ and the glycine linker are in red. Active site residues are represented by green sticks, and the detected pocket is shown in a cyan surface. The pocket opposed to the active site, usually found in ligand bound structures (35) is depicted in a brown surface, merely for illustration. In (B), the red line is a smoothed curve of the black line, intended to clarify the breathing behavior of the active site.

Following detection, the protease pocket coordinates (Figure 7A) were subjected to the MD pocket algorithm to be monitored for volume changes. As in many proteases [54–57], the pocket volume presented a ‘breathing’ behavior, adopting a sinusoidal curve when plotted against time (Figure 7B). This suggested that, even if the protein core remained stable during the same simulation, the binding site accessibility seemed to change, probably due to allosteric modulation provided by the more flexible domains.

#### Protein/ligand interactions evolution

**Figure 8:**
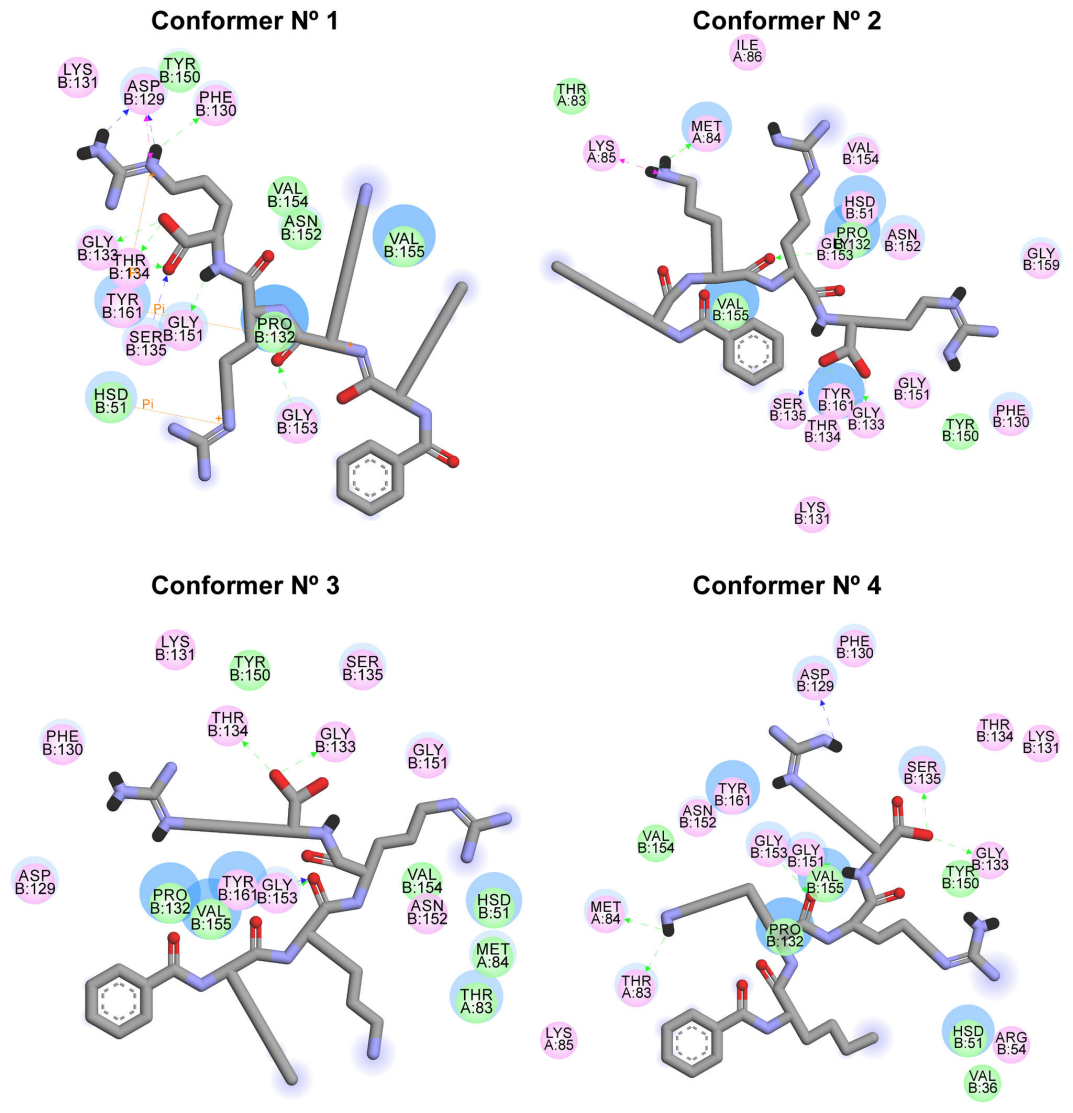
Binding mode fluctuations during the MD simulation. Both ligand and binding site plasticity contributes to changes in the binding mode between the ligand and protein. The solvent accessible surface of an interacting residue or atom is represented by a blue halo around it. The diameter of the circle is proportional to the solvent accessible surface. Pink circles indicate residues involved in hydrogen-bond, polar or charged interactions; green circles indicates residues involved in van der Waals interactions; π-interactions are represented by orange lines; green arrows indicate hydrogen-bonds interactions with amino acid main chains; blue arrows indicate side chain hydrogen-bond interactions. Arrowhead directs towards the electron donor.

Ligplots of the protein/ligand binding modes (Figure 8) showed changes in both the protein sub-pockets conformation and in ligand interactions, indicating that the binding site is definitely not static and that this plasticity could be explored for competitive structure based drug design.

Noteworthy was the fact that the residues from the S2 sub-pocket were represented only by THR_83_ in the conformations 2-4, but this could not be depicted in the conformation 1. In this case, the protein relaxation promoted by the MD simulation may have exposed this residue so it could be detected by this method. The residues from the S3 pocket (MET_84_, LYS_85_ and ILE_86_) showed the same pattern, all absent in conformer 1 but with variable incidence in the other conformations. As for residue interactions, some were more conserved than others. In all conformations, residues GLY_133_ and GLY_153_ were involved with hydrogen bonds between their main chain atoms and the C-terminal oxygen from P1 and the backbone oxygen from P2, respectively. On the other hand, π-interactions seemed to need a more constrained conformation, as this was observed only with the aromatic residues TYR_161_ (with both P1 and P3) and HIS_51_ (with P2) of the first conformation. Hydrogen bonds coming from SER_135_, GLY_151_ and THR_134_ were less conserved, but also related to the C-terminal oxygen from P1. Interactions between P3 and S3 were observed only in conformations 2 and 4; side-chain interactions from P1 were observed only in conformation 1 and 4.

**Figure 9:**
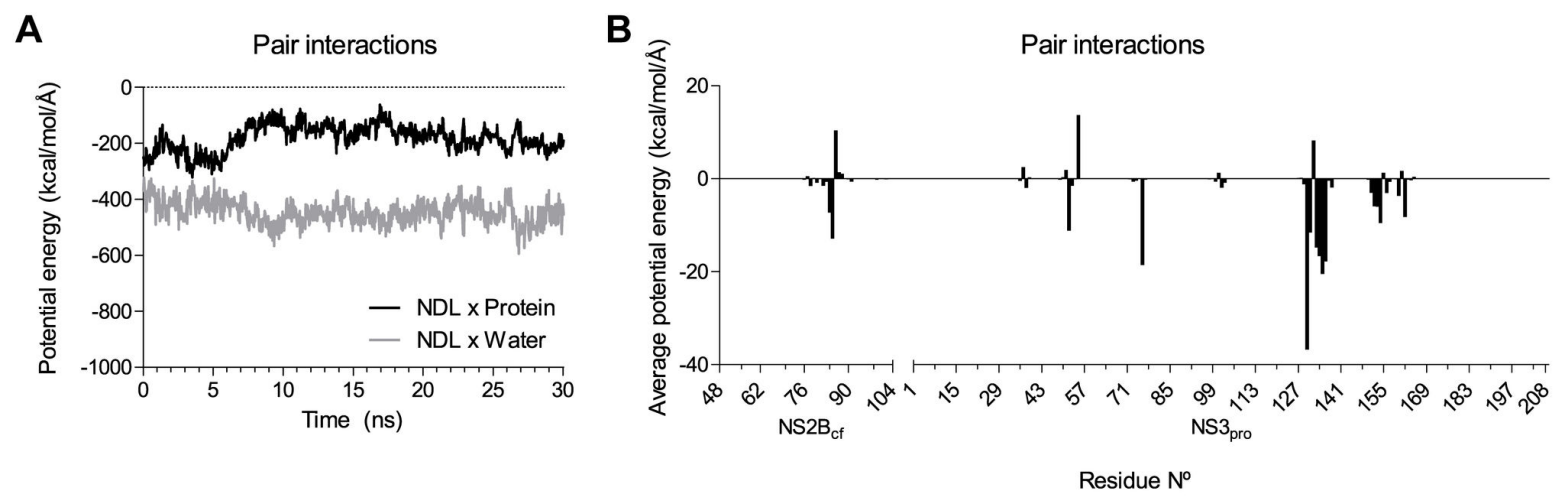
Total pair interaction energies (potential plus kinetic). (A) Pair interactions from the ligand NDL *versus* protein (black line), and NDL *versus* water (gray line). (B) Average total energy of pair interactions from the ligand and each protein residue, allowing for contact points mapping.

To investigate further the plasticity of the DENV NS2B _CF_NS3_PRO_ binding site and the possible consequences on ligand binding, we analyzed the protein/ligand interactions during the whole MD trajectory. For that purpose, we calculated the pair interaction energies between the ligand NDL and the protein (Figure 9A, black line), the ligand and surrounding water (Figure 9A, gray line) and for the ligand with each protein residue (Figure 9B). Contact points between ligand and protein could be mapped by plotting the average total energy (potential plus kinetic) of each residue. Similar to results obtained from the binding plots, the main residues involved in the interactions with the ligand were from the sub-pockets S3 (residues MET_84_, LYS_85_ and ILE_86_), and all the residues from S1 and S4. The greatest interaction averages came from ASP_129_, followed by THR_134_ and SER_135_ from S1. However, the evaluation of pair interaction forces was more sensitive for detecting residues from the sub-pocket S2, as it was able to recognize residues ASP_75_, LYS_73_ and VAL_72_, none of them found within any conformation inspected through the ligand-binding plots.

When analyzing the ligand/protein and the ligand/water interactions together (Figure 9A), we could see that while the energy of the former increases (thus, diminishing ligand-protein attraction), the latter seems to decrease proportionally (enhancing the ligand-water attraction). This is normally expected, as during MD simulations, the protein-ligand complex becomes more relaxed, allowing water to interact with the residues from the binding site. This results in decreased interactions between ligand and protein. It is interesting to note that, despite the attraction between ligand and protein being diminished at the beginning of the simulation, the complex achieves stabilization after 10 ns. In fact, one can say that during the last 5 ns of simulation, the protein-ligand interaction increases again, approaching the initial state.

## Discussion

One of the limitations in using crystallographic structures in structure-based drug design is the fact that those structures are often related to a particular energy minima state, which is a single conformation among many possible ones. Although this does not represent a significant problem, it may undermine virtual high-throughput campaigns. To circumvent this issue, one may use a range of strategies for simulating protein flexibility, among which we can highlight the ensemble docking with structures derived from molecular dynamics simulations [38,40,41].

In the last few years, several DENV NS3_PRO_ automatic docking studies were published based on homology models [23–26,58–60]. In our work, besides investigating protein flexibility, we were careful to build our DENV NS3_PRO_ model in the predicted active conformational state (including the oxyanion hole), a concern also present in the study by Knehans et al. [24]. However, most of the other studies were based either on the retracted apoenzyme structure [26,59], or in the open holoenzyme conformation [23,25,58] and were based on a rigid protein system with a single conformation.

The first homology model for the DENV NS2B/NS3 protease was done by Brinkworth et al. in 1999 [61]. However, as it was built based on the more distantly related hepatitis C virus NS3/NS4A protease (PDB id 1JXP [62]), several structural differences (Cα RMSD of 11 Å) were observed when comparing it to the recently resolved DENV-3 NS2B/NS3 protease (PDB id 3U1I [35]). Also, this work did not take into account the ligand-binding induced fit mechanism, as it was yet to be proposed [33,63], and thus, ligand was absent from their model. Later, in 2007, Kee et al. [64] used this same model for automatic docking studies. In 2004 and 2005, respectively, Niyomrattanakit et al. [65] and Chanprapaph et al. [60] built homology models based on the now retracted apo DENV NS3_PRO_ structure. Similarly, Ganesh et al., in 2005 [59], and Tomlinson et al., in 2009 [26], used homology models based on the retracted structure for automatic docking studies. For studying the importance of domain motion between the NS3_PRO_ and NS3_HEL_ domains through MD simulations (1 ns), Rosales-León et al., in 2007 [66], built a full DENV NS3 model, but also based on this retracted structure. In 2010, two groups [29,67] independently published a similar approach for building active conformational models, where the cofactor and the ligand were extracted from the WNV NS2B_CF_/NS3_PRO_ structure (PDB id 2FP7) and fused to the DENV NS3_PRO_. However, the oxyanion hole is not in the active conformation in the work by Wichapong and colleagues [67], and could not be accessed in the work by Frecer and Miertus [29] for further comparisons. Also, neither models presented linker regions nor grafted loops.

Regarding protein flexibility, Ekonomiuk et al. [31] also postulated that it would be important to access new conformations for vHTS in the case of the WNV protease. However, instead of performing a long MD simulation, they preferred a short run (1 ns) to look for a conformation able to better accommodate a benzene ring later used for fragment docking. Figure 5A shows the importance of performing a longer simulation to our purposes: even if a protein seems stable during shorter trajectories, they can surpass greater conformational changes after longer simulation times, revealing important motions that otherwise would not be detectable. These motions can be related to important conformational states found during protein equilibrium, which in turn could be subjected to vHTS. When comparing the one- and two-dimensional RMSD graphs, we can observe that the variations in the former are related to conformational changes in the latter, which are maintained in a clustered family. This means that conformational changes observed in the one-dimensional RMSD plot last for some time, which is extremely desirable for finding new inhibitors that may bind to this protein state.

Despite presenting higher conservation during the trajectory, the binding site also presents considerable plasticity that may be important in structure-based drug discovery. In the work by Ekonomiuk et al. [31], backbone changes as low as 0.8 Å between the crystallographic structure and their model extracted from MD simulations were enough to better accommodate three different fragments used in vHTS. In our case, the average binding site heavy atoms deviation was approximately 2 Å, reflecting changes both in pocket volume (Figure 7B) and in ligand binding modes (Figure 8).

In an already mentioned work, Wichapong et al. [67] performed 10 ns of MD simulations to examine ligand-protein interactions in three different models. Irrespective of the differences between this work and that of Wichapong and colleagues’ (for example: the inhibitor was covalently bound to SER_135_, the absence of the linker region and grafted loops, and the position of the oxyanion hole), our analysis seems to be in accordance with theirs. However, as they did not perform time-wise analysis related to different points in the trajectory, changes in the binding modes through the simulation could not be evaluated.

Despite the fact that almost every binding pocket residue found in the literature was also detected in our study, binding modes were sometimes quite distinct. With the recent publication of the ligand-bound structure of the DENV-3 NS3 protease [36], using the same inhibitor used in our study, we were able to compare the protease sub-pockets. While in the crystallographic data GLY_151_ interacts with the P2 residue from the inhibitor, in our study it interacts promptly with the P1 residue. Other changes include GLY_153_, part of the S3 sub-pocket in the crystallographic structure, but conserved in S2 in all conformations from our model; and the residue TYR_161_, which was capable of making π-interactions with both P1 and P3 residues in our studies, while it is described as a S1 sub-pocket residue in the crystallographic structure. Also, LYS_85_ from the cofactor NS2B was able to produce a charge-charge interaction with the P3 LYS from the inhibitor, a fact not observed in the work from Noble et al. [36]. Altogether, those changes in binding mode, either between our models and the crystallographic structure or among different conformations extracted from our MD simulations, indicate that the binding site indeed presents significant flexibility that could be explored in structure-based drug design.

## Conclusions

This article describes the analysis of the *Dengue virus* NS3 protease flexibility through molecular dynamics simulations, as a part of an ensemble docking campaign for identifying potential new leads for drug design. As a result, we were able to clearly distinguish four different conformational states, which are being used in a subsequent virtual high-throughput ensemble docking campaign. By doing so, we hope to discover new promising molecules with therapeutic potential to treat this life-threatening disease.

## Supporting Information

Figure S1 **The binding site of each representative conformation identified by our clustering strategy.** The NDL inhibitor is displayed as a stick model (C, O and N atoms in white, red, and blue, respectively). Residues participating in the binding site are displayed both as thin sticks and as transparent surfaces colored accordingly to the residue name. The plasticity of the binding site environment and in the binding modes is evidenced by the differences observed during the molecular dynamics simulations.
(TIF)

Figure S2 **Snapshot from the last frame of the 30ns trajectory, showing that both the inhibitor and the cofactor remain closely bound to the NS3 protease.** The NS2B_CF_ is maintained in the “closed” conformation. NS3_PRO_ is depicted in gray, NS2B_CF_ (and linker) in red. Active site side-chains are represented as green sticks, and the NDL inhibitor in orange.
(TIF)

File S1 **PDB file of the final model with all its components: NS3_PRO_, NS2B_CF_, glycine linker and NDL inhibitor.** For displaying it in a molecular viewing program (such as VMD), please copy the content of the. doc file, paste it into a new document in a simple text editor (MS Windows notepad, for instance), and save it with a. pdb file extension.
(DOC)

## References

[1] Halstead SB, Nimmannitya S, Yamarat C, Russell PK (1967) Hemorrhagic fever in Thailand; recent knowledge regarding etiology. Jpn J Med Sci Biol 20 Suppl: 96–103. PubMed: 5301574. 5301574

[2] Zellweger RM, Prestwood TR, Shresta S (2010) Enhanced infection of liver sinusoidal endothelial cells in a mouse model of antibody-induced severe dengue disease. Cell Host Microbe 7: 128–139. doi:10.1016/j.chom.2010.01.004. PubMed: 20153282. 20153282 10.1016/j.chom.2010.01.004 PMC2824513

[3] Beatty ME, Stone A, Fitzsimons DW, Hanna JN, Lam SK et al. (2010) Best practices in dengue surveillance: a report from the Asia-Pacific and Americas Dengue Prevention Boards. PLOS Neglected Trop Dis 4: e890. doi:10.1371/journal.pntd.0000890. PubMed: 21103381. 10.1371/journal.pntd.0000890 PMC2982842 21103381

[4] Schwartz E (2008) Seasonality, Annual Trends, and Characteristics of Dengue among Ill Returned Travelers. pp. 1997 to 2006. Emerging Infectious Diseases 14: 1081–1088 doi:10.3201/eid1407.071412. 10.3201/eid1407.071412 PMC2600332 18598629

[5] Caminade C, Medlock JM, Ducheyne E, McIntyre KM, Leach S et al. (2012) Suitability of European climate for the Asian tiger mosquito Aedes albopictus: recent trends and future scenarios. J R Soc Interface, 9: 2708–17. doi:10.1098/rsif.2012.0138. PubMed: 22535696. PubMed: 22535696 22535696 10.1098/rsif.2012.0138 PMC3427500

[6] Gubler DJ (2011) Dengue, Urbanization and Globalization: The Unholy Trinity of the 21(st) Century. Trop Med Health 39: 3–11. doi:10.2149/tmh.2011-S05. PubMed: 22500131. 10.2149/tmh.2011-S05 PMC3317603 22500131

[7] La Ruche G, Souarès Y, Armengaud A, Peloux-Petiot F, Delaunay P et al. (2010) First two autochthonous dengue virus infections in metropolitan France, September 2010. Euro surveillance : bulletin européen sur les maladies transmissibles = European communicable disease bulletin 15. p. 19676 PubMed : 20929659 20929659

[8] Franco C, Hynes NA, Bouri N, Henderson DA (2010) The dengue threat to the United States. Biosecurity Bioterrorism Biodefense Strategy Practice Sci 8: 273–276. doi:10.1089/bsp.2010.0032. PubMed: 20718665. 10.1089/bsp.2010.0032 20718665

[9] Whitehead SS, Blaney JE, Durbin AP, Murphy BR (2007) Prospects for a dengue virus vaccine. Nat Rev Microbiol 5: 518–528. doi:10.1038/nrmicro1690. PubMed: 17558424. 17558424 10.1038/nrmicro1690

[10] Konishi E (2011) Issues related to recent dengue vaccine development. Trop Med Health 39: 63–71. doi:10.2149/tmh.2011-S01. PubMed: 22500138. 22500138 10.2149/tmh.2011-S01 PMC3317602

[11] Steuber H, Hilgenfeld R (2010) Recent Advances in Targeting Viral Proteases for the Discovery of Novel Antivirals. Curr Top Med Chem 10: 323–345. doi:10.2174/156802610790725470. PubMed: 20166951. 20166951 10.2174/156802610790725470

[12] Umareddy I, Pluquet O, Wang QY, Vasudevan SG, Chevet E et al. (2007) Dengue virus serotype infection specifies the activation of the unfolded protein response. Virol J 4: 91. doi:10.1186/1743-422X-4-91. PubMed: 17888185. 17888185 10.1186/1743-422X-4-91 PMC2045667

[13] Falgout B, Miller RH, Lai CJ (1993) Deletion analysis of dengue virus type 4 nonstructural protein NS2B: identification of a domain required for NS2B-NS3 protease activity. J Virol 67: 2034–2042. PubMed: 8383225. 8383225 10.1128/jvi.67.4.2034-2042.1993 PMC240272

[14] Clum S, Ebner KE, Padmanabhan R (1997) Cotranslational membrane insertion of the serine proteinase precursor NS2B-NS3(Pro) of dengue virus type 2 is required for efficient in vitro processing and is mediated through the hydrophobic regions of NS2B. J Biol Chem 272: 30715–30723. doi:10.1074/jbc.272.49.30715. PubMed: 9388208. 9388208 10.1074/jbc.272.49.30715

[15] Natarajan S (2010) NS3 protease from flavivirus as a target for designing antiviral inhibitors against dengue virus. Genet Mol Biol 33: 214–219. doi:10.1590/S1415-47572010000200002. PubMed: 21637471. 21637471 10.1590/S1415-47572010000200002 PMC3036867

[16] Lescar J, Luo D, Xu T, Sampath A, Lim SP et al. (2008) Towards the design of antiviral inhibitors against flaviviruses: the case for the multifunctional NS3 protein from Dengue virus as a target. Antiviral Res 80: 94–101. doi:10.1016/j.antiviral.2008.07.001. PubMed: 18674567. 18674567 10.1016/j.antiviral.2008.07.001

[17] Nitsche C, Behnam M a M, Steuer C, Klein CD (2012) Retro peptide-hybrids as selective inhibitors of the Dengue virus NS2B-NS3 protease. Antiviral research 94: 72–79. doi:10.1016/j.antiviral.2012.02.008. 22391061 10.1016/j.antiviral.2012.02.008

[18] Tiew K-C, Dou D, Teramoto T, Lai H, Alliston KR et al. (2012) Inhibition of Dengue virus and West Nile virus proteases by click chemistry-derived benz[d]isothiazol-3(2H)-one derivatives. Bioorg Med Chem 20: 1213–1221. doi:10.1016/j.bmc.2011.12.047. PubMed: 22249124. 22249124 10.1016/j.bmc.2011.12.047 PMC3279297

[19] Tomlinson SM, Watowich SJ (2012) Use of parallel validation high-throughput screens to reduce false positives and identify novel dengue NS2B-NS3 protease inhibitors. Antiviral Res 93: 245–252. doi:10.1016/j.antiviral.2011.12.003. PubMed: 22193283. 22193283 10.1016/j.antiviral.2011.12.003 PMC3266433

[20] Nitsche C, Steuer C, Klein CD (2011) Arylcyanoacrylamides as inhibitors of the Dengue and West Nile virus proteases. Bioorg Med Chem 19: 7318–7337. doi:10.1016/j.bmc.2011.10.061. PubMed: 22094280. 22094280 10.1016/j.bmc.2011.10.061

[21] Schüller A, Yin Z, Brian Chia CS, Doan DNP, Kim H-K et al. (2011) Tripeptide inhibitors of dengue and West Nile virus NS2B-NS3 protease. Antiviral Res 92: 96–101. doi:10.1016/j.antiviral.2011.07.002. PubMed: 21763725. 21763725 10.1016/j.antiviral.2011.07.002

[22] Cregar-Hernandez L, Jiao G-S, Johnson AT, Lehrer AT, Wong TAS et al. (2011) Small molecule pan-dengue and West Nile virus NS3 protease inhibitors. Antivir Chem Chemother 21: 209–217. doi:10.3851/IMP1767. PubMed: 21566267. 21566267 10.3851/IMP1767 PMC3095516

[23] Frimayanti N, Chee CF, Zain SM, Rahman NA (2011) Design of new competitive dengue ns2b/ns3 protease inhibitors-a computational approach. Int J Mol Sci 12: 1089–1100. doi:10.3390/ijms12021089. PubMed: 21541045. 21541045 10.3390/ijms12021089 PMC3083692

[24] Knehans T, Schüller A, Doan DN, Nacro K, Hill J et al. (2011) Structure-guided fragment-based in silico drug design of dengue protease inhibitors. J Comput Aid Mol Des 25: 263–274. doi:10.1007/s10822-011-9418-0. PubMed: 21344277. 10.1007/s10822-011-9418-0 21344277

[25] Frimayanti N, Zain SM, Lee VS, Wahab Ha, Yusof R et al (2012) Fragment-based molecular design of new competitive dengue Den2 Ns2b/Ns3 inhibitors from the components of fingerroot (Boesenbergia rotunda). In Silico Biol 11: 29–37 doi:10.3233/ISB-2012-0442. PubMed: 22475750. 10.3233/ISB-2012-0442 22475750

[26] Tomlinson SM, Malmstrom RD, Russo A, Mueller N, Pang Y-P et al. (2009) Structure-based discovery of dengue virus protease inhibitors. Antiviral Res 82: 110–114. doi:10.1016/j.antiviral.2009.02.190. PubMed: 19428601. 19428601 10.1016/j.antiviral.2009.02.190 PMC2680748

[27] Tomlinson SM, Malmstrom RD, Watowich SJ (2009) New approaches to structure-based discovery of dengue protease inhibitors. Infect Disord Drug Targets 9: 327–343. doi:10.2174/1871526510909030327. PubMed: 19519486. 19519486 10.2174/1871526510909030327

[28] Shiryaev Sa, Cheltsov AV, Gawlik K, Ratnikov BI, Strongin AY (2011) Virtual ligand screening of the National Cancer Institute (NCI) compound library leads to the allosteric inhibitory scaffolds of the West Nile Virus NS3 proteinase. Assay Drug Dev Technol 9: 69–78. doi:10.1089/adt.2010.0309. PubMed: 21050032. 21050032 10.1089/adt.2010.0309 PMC3033206

[29] Frecer V, Miertus S (2010) Design, structure-based focusing and in silico screening of combinatorial library of peptidomimetic inhibitors of Dengue virus NS2B-NS3 protease. J Comput Aid Mol Des 24: 195–212. doi:10.1007/s10822-010-9326-8. PubMed: 20306283. 10.1007/s10822-010-9326-8 20306283

[30] Ekonomiuk D, Su X-C, Ozawa K, Bodenreider C, Lim SP et al. (2009) Discovery of a non-peptidic inhibitor of west nile virus NS3 protease by high-throughput docking. PLOS Neglected Trop Dis 3: e356. doi:10.1371/journal.pntd.0000356. 10.1371/journal.pntd.0000356 PMC2613028 19159012

[31] Ekonomiuk D, Su X-C, Ozawa K, Bodenreider C, Lim SP et al. (2009) Flaviviral protease inhibitors identified by fragment-based library docking into a structure generated by molecular dynamics. J Med Chem 52: 4860–4868. doi:10.1021/jm900448m. PubMed: 19572550. 19572550 10.1021/jm900448m

[32] Spyrakis F, BidonChanal A, Barril X, Luque FJ (2011) Protein flexibility and ligand recognition: challenges for molecular modeling. Curr Top Med Chem 11: 192–210. doi:10.2174/156802611794863571. PubMed: 20939788. 20939788 10.2174/156802611794863571

[33] Erbel P, Schiering N, D’Arcy A, Renatus M, Kroemer M et al. (2006) Structural basis for the activation of flaviviral NS3 proteases from dengue and West Nile virus. Nat Struct Mol Biol 13: 372–373. doi:10.1038/nsmb1073. PubMed: 16532006. 16532006 10.1038/nsmb1073

[34] Luo D, Xu T, Hunke C, Grüber G, Vasudevan SG et al. (2008) Crystal structure of the NS3 protease-helicase from dengue virus. J Virol 82: 173–183. doi:10.1128/JVI.01788-07. PubMed: 17942558. 17942558 10.1128/JVI.01788-07 PMC2224403

[35] Chandramouli S, Joseph JS, Daudenarde S, Gatchalian J, Cornillez-Ty C et al. (2010) Serotype-specific structural differences in the protease-cofactor complexes of the dengue virus family. J Virol 84: 3059–3067. doi:10.1128/JVI.02044-09. PubMed: 20042502. 20042502 10.1128/JVI.02044-09 PMC2826037

[36] Noble CG, Seh CC, Chao AT, Shi PY (2012) Ligand-bound structures of the dengue virus protease reveal the active conformation. J Virol 86: 438–446. doi:10.1128/JVI.06225-11. PubMed: 22031935. 22031935 10.1128/JVI.06225-11 PMC3255909

[37] Huang SY, Zou X (2007) Ensemble docking of multiple protein structures: considering protein structural variations in molecular docking. Proteins 66: 399–421. doi:10.1002/prot.21214. PubMed: 17096427. 17096427 10.1002/prot.21214

[38] Korb O, Olsson TSG, Bowden SJ, Hall RJ, Verdonk ML et al. (2012) Potential and limitations of ensemble docking. J Chem Inf Model 52: 1262–1274. doi:10.1021/ci2005934. PubMed: 22482774. 22482774 10.1021/ci2005934

[39] Sinko W, Lindert S, McCammon JA (2013) Accounting for receptor flexibility and enhanced sampling methods in computer-aided drug design. Chem Biol Drug Des 81: 41–49. doi:10.1111/cbdd.12051. PubMed: 23253130. 23253130 10.1111/cbdd.12051 PMC3540989

[40] Amaro RE, Li WW (2010) Emerging Methods for Ensemble-Based Virtual Screening. Curr Top Med Chem 10: 3–13. doi:10.2174/156802610790232279. PubMed: 19929833. 19929833 10.2174/156802610790232279 PMC3086266

[41] Osguthorpe DJ, Sherman W, Hagler AT (2012) Exploring protein flexibility: incorporating structural ensembles from crystal structures and simulation into virtual screening protocols. J Phys Chem B 116: 6952–6959. doi:10.1021/jp3003992. PubMed: 22424156. 22424156 10.1021/jp3003992 PMC3376248

[42] Eswar N, Webb B, Marti-Renom MA, Madhusudhan MS, Eramian D et al. (2006) Comparative protein structure modeling using Modeller. Current protocols in bioinformatics / editorial board, Andreas D Baxevanis. [et al.]Chapter 5: Unit 5.6. doi:10.1002/0471250953.bi0506s15. 10.1002/0471250953.bi0506s15 PMC4186674 18428767

[43] Laskowski RA, MacArthur MW, Moss DS, Thornton JM (1993) PROCHECK: a program to check the stereochemical quality of protein structures. J Appl Crystallogr 26: 283–291. doi:10.1107/S0021889892009944.

[44] Humphrey W (1996) VMD: Visual molecular dynamics. J Mol Graph 14: 33–38. doi:10.1016/0263-7855(96)00018-5. PubMed: 8744570. 8744570 10.1016/0263-7855(96)00018-5

[45] Brooks BR, Brooks CL, Mackerell AD, Nilsson L, Petrella RJ et al. (2009) CHARMM: the biomolecular simulation program. J Comput Chem 30: 1545–1614. doi:10.1002/jcc.21287. PubMed: 19444816. 19444816 10.1002/jcc.21287 PMC2810661

[46] Phillips JC, Braun R, Wang W, Gumbart J, Tajkhorshid E et al. (2005) Scalable molecular dynamics with NAMD. J Comput Chem 26: 1781–1802. doi:10.1002/jcc.20289. PubMed: 16222654. 16222654 10.1002/jcc.20289 PMC2486339

[47] Darden T, York D, Pedersen L (1993) Particle mesh Ewald: An N⋅log(N) method for Ewald sums in large systems. J Chem Phys 98: 10089. doi:10.1063/1.464397.

[48] Ryckaert J-P, Ciccotti G, Berendsen HJ (1977) Numerical integration of the cartesian equations of motion of a system with constraints: molecular dynamics of n-alkanes. J Comput Phys 23: 327–341. doi:10.1016/0021-9991(77)90098-5.

[49] Zhang Z, Li Y, Lin B, Schroeder M, Huang B (2011) Identification of cavities on protein surface using multiple computational approaches for drug binding site prediction. Bioinformatics (Oxf, England) 27: 2083–2088. doi:10.1093/bioinformatics/btr331. PubMed: 21636590. 10.1093/bioinformatics/btr331 21636590

[50] Schmidtke P, Bidon-Chanal A, Luque FJ, Barril X (2011) MDpocket: open-source cavity detection and characterization on molecular dynamics trajectories. Bioinformatics (Oxf, England) 27: 3276–3285. doi:10.1093/bioinformatics/btr550. PubMed: 21967761. 10.1093/bioinformatics/btr550 21967761

[51] Assenberg R, Mastrangelo E, Walter TS, Verma A, Milani M et al. (2009) Crystal structure of a novel conformational state of the flavivirus NS3 protein: implications for polyprotein processing and viral replication. J Virol 83: 12895–12906. doi:10.1128/JVI.00942-09. PubMed: 19793813. 19793813 10.1128/JVI.00942-09 PMC2786852

[52] Luo D, Wei N, Doan DN, Paradkar PN, Chong Y et al. (2010) Flexibility between the protease and helicase domains of the dengue virus NS3 protein conferred by the linker region and its functional implications. J Biol Chem 285: 18817–18827. doi:10.1074/jbc.M109.090936. PubMed: 20375022. 20375022 10.1074/jbc.M109.090936 PMC2881804

[53] Salaemae W, Junaid M, Angsuthanasombat C, Katzenmeier G (2010) Structure-guided mutagenesis of active site residues in the dengue virus two-component protease NS2B-NS3. J Biomed Sci 17: 68. doi:10.1186/1423-0127-17-68. PubMed: 20735839. 20735839 10.1186/1423-0127-17-68 PMC2933675

[54] Miller DW, Agard D a (1999) Enzyme specificity under dynamic control: a normal mode analysis of alpha-lytic protease. J Mol Biol 286: 267–278. doi:10.1006/jmbi.1998.2445. PubMed: 9931265. 9931265 10.1006/jmbi.1998.2445

[55] Schmidt A, Lamzin VS (2005) Extraction of functional motion in trypsin crystal structures. Acta Crystallogr D Biol Crystallogr 61: 1132–1139. doi:10.1107/S0907444905016732. PubMed: 16041079. 16041079 10.1107/S0907444905016732

[56] Martin JR, Mulder FA, Karimi-Nejad Y, van der Zwan J, Mariani M et al. (1997) The solution structure of serine protease PB92 from Bacillus alcalophilus presents a rigid fold with a flexible substrate-binding site. Structure 5: 521–532. doi:10.1016/S0969-2126(97)00208-6. PubMed: 9115441. 9115441 10.1016/s0969-2126(97)00208-6

[57] Liu S-Q, Meng Z-H, Fu Y-X, Zhang K-Q (2009) Insights derived from molecular dynamics simulation into the molecular motions of serine protease proteinase K. J Mol Model, 16: 17–28. doi:10.1007/s00894-009-0518-x. PubMed: 19466463. 19466463 10.1007/s00894-009-0518-x

[58] Tomlinson SM, Watowich SJ (2011) Anthracene-based inhibitors of dengue virus NS2B-NS3 protease. Antiviral Res 89: 127–135. doi:10.1016/j.antiviral.2010.12.006. PubMed: 21185332. 21185332 10.1016/j.antiviral.2010.12.006 PMC3026091

[59] Ganesh VK, Muller N, Judge K, Luan C-H, Padmanabhan R et al. (2005) Identification and characterization of nonsubstrate based inhibitors of the essential dengue and West Nile virus proteases. Bioorg Med Chem 13: 257–264. doi:10.1016/j.bmc.2004.09.036. PubMed: 15582469. 15582469 10.1016/j.bmc.2004.09.036

[60] Chanprapaph S, Saparpakorn P, Sangma C, Niyomrattanakit P, Hannongbua S et al. (2005) Competitive inhibition of the dengue virus NS3 serine protease by synthetic peptides representing polyprotein cleavage sites. Biochem Biophys Res Commun 330: 1237–1246. doi:10.1016/j.bbrc.2005.03.107. PubMed: 15823576. 15823576 10.1016/j.bbrc.2005.03.107

[61] Brinkworth RI, Fairlie DP, Leung D, Young PR (1999) Homology model of the dengue 2 virus NS3 protease: putative interactions with both substrate and NS2B cofactor. J Gen Virol 80: 1167–1177. PubMed: 10355763. 10355763 10.1099/0022-1317-80-5-1167

[62] Yan Y, Li Y, Munshi S, Sardana V, Cole JL et al. (1998) Complex of NS3 protease and NS4A peptide of BK strain hepatitis C virus: a 2.2 A resolution structure in a hexagonal crystal form. Protein Sci Publ Protein Soc 7: 837–847. doi:10.1002/pro.5560070402. 10.1002/pro.5560070402 PMC2143993 9568891

[63] Aleshin AE, Shiryaev SA, Strongin AY, Liddington RC (2007) Structural evidence for regulation and specificity of flaviviral proteases and evolution of the Flaviviridae fold. Protein Sci Publ Protein Soc 16: 795–806. doi:10.1110/ps.072753207. PubMed: 17400917. 10.1110/ps.072753207 PMC2206648 17400917

[64] Lee Y, Tan S, Wahab H, Yusof R, Rahman NA (2007) Nonsubstrate Based Inhibitors of Dengue Virus Serine Protease: A Molecular Docking Approach to Study Binding Interactions between Protease and Inhibitors. Asia Pac J Mol Biol Biotechnol 15: 53–59.

[65] Niyomrattanakit P, Winoyanuwattikun P, Chanprapaph S, Angsuthanasombat C, Panyim S et al. (2004) Identification of residues in the dengue virus type 2 NS2B cofactor that are critical for NS3 protease activation. J Virol 78: 13708–13716. doi:10.1128/JVI.78.24.13708-13716.2004. PubMed: 15564480. 15564480 10.1128/JVI.78.24.13708-13716.2004 PMC533897

[66] Rosales-León L, Ortega-Lule G, Ruiz-Ordaz B (2007) Analysis of the domain interactions between the protease and helicase of NS3 in dengue and hepatitis C virus. J Mol Graph Modell 25: 585–594. doi:10.1016/j.jmgm.2006.04.001. PubMed: 16762573. 10.1016/j.jmgm.2006.04.001 16762573

[67] Wichapong K, Pianwanit S, Sippl W, Kokpol S (2010) Homology modeling and molecular dynamics simulations of Dengue virus NS2B/NS3 protease: insight into molecular interaction. J Mol Recognit JMR 23: 283–300. doi:10.1002/jmr.977. PubMed: 19693793. 19693793 10.1002/jmr.977

[68] Eargle J, Wright D, Luthey-Schulten Z (2006) Multiple Alignment of protein structures and sequences for VMD. Bioinformatics (Oxf, England) 22: 504–506. doi:10.1093/bioinformatics/bti825. PubMed: 16339280. 10.1093/bioinformatics/bti825 16339280

[69] Robin G, Chappell K, Stoermer MJ, Hu S-H, Young PR et al. (2009) Structure of West Nile virus NS3 protease: ligand stabilization of the catalytic conformation. J Mol Biol 385: 1568–1577. doi:10.1016/j.jmb.2008.11.026. PubMed: 19059417. 19059417 10.1016/j.jmb.2008.11.026

