# Supplementary figures and images for: New Binding Site Conformations of the Dengue Virus NS3 Protease Accessed by Molecular Dynamics Simulation
Source: PLoS One. 2013 Aug 21;8(8):e72402. doi: 10.1371/journal.pone.0072402 (PMC3749139; doi:10.1371/journal.pone.0072402)

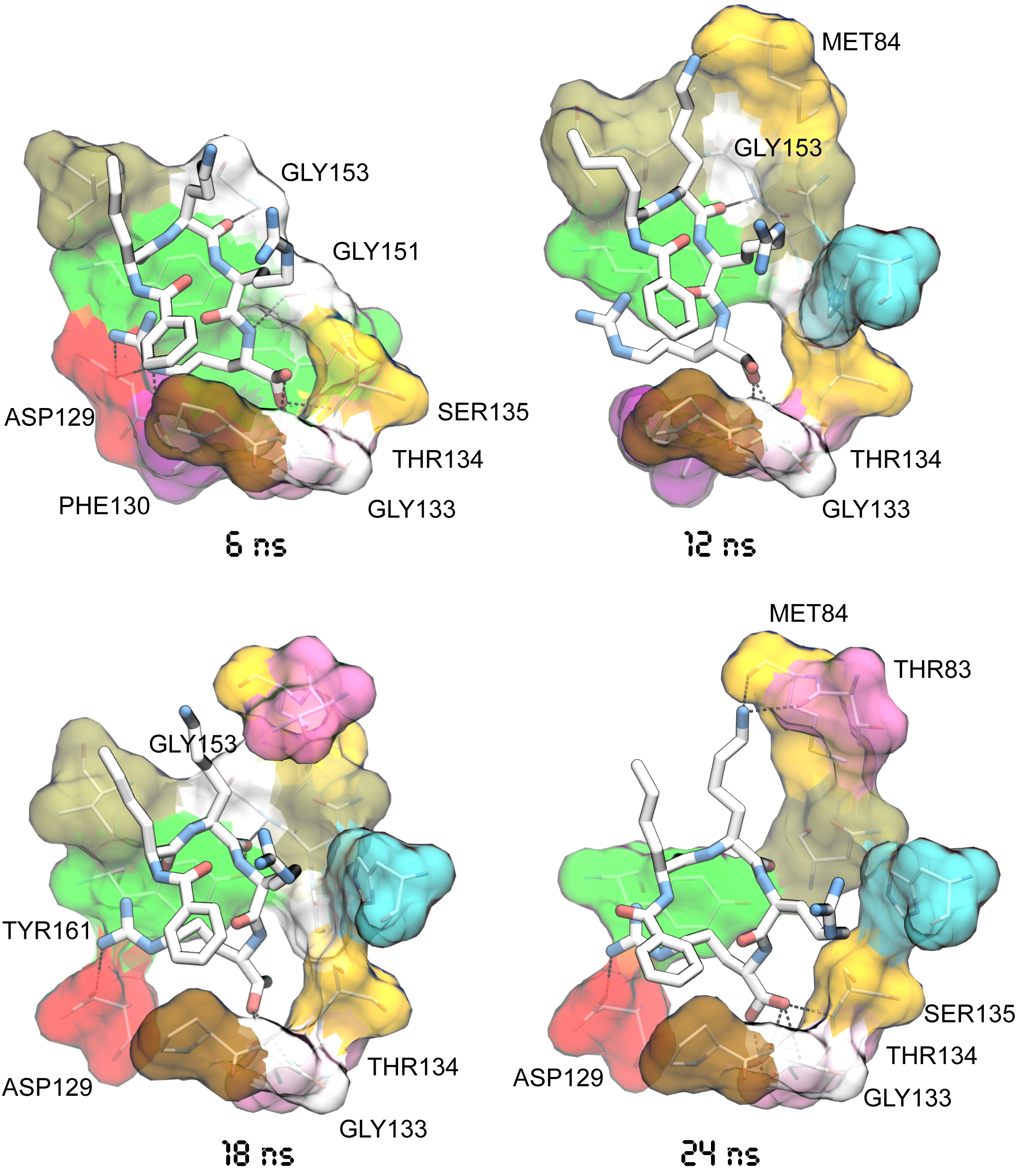

Supplement: Figure S1 — The binding site of each representative conformation identified by our clustering strategy. The NDL inhibitor is displayed as a stick model (C, O and N atoms in white, red, and blue, respectively). Residues participating in the binding site are displayed both as thin sticks and as transparent surfaces colored accordingly to the residue name. The plasticity of the binding site environment and in the binding modes is evidenced by the differences observed during the molecular dynamics simulations. (TIF) [file pone.0072402.s001.tif]

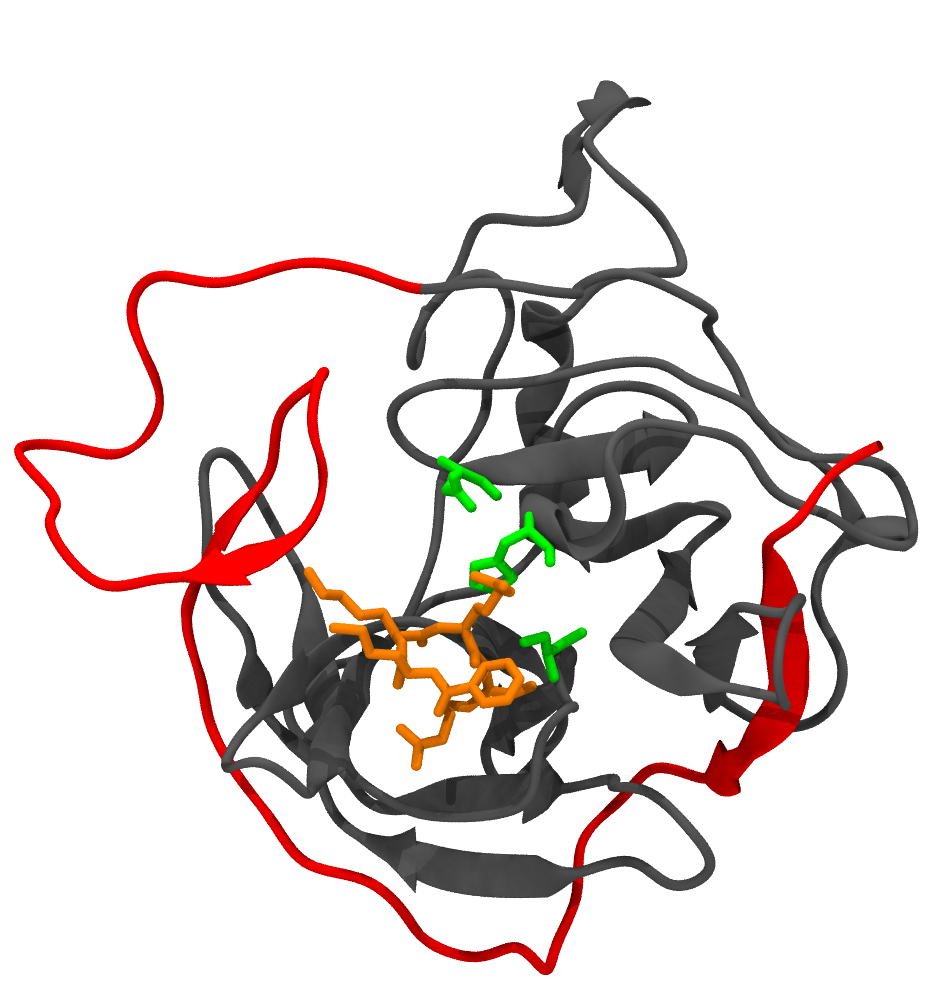

Supplement: Figure S2 — Snapshot from the last frame of the 30ns trajectory, showing that both the inhibitor and the cofactor remain closely bound to the NS3 protease. The NS2BCF is maintained in the “closed” conformation. NS3PRO is depicted in gray, NS2BCF (and linker) in red. Active site side-chains are represented as green sticks, and the NDL inhibitor in orange. (TIF) [file pone.0072402.s002.tif]
