# Supplementary material for: New Binding Site Conformations of the Dengue Virus NS3 Protease Accessed by Molecular Dynamics Simulation
Source: PLoS One. 2013 Aug 21;8(8):e72402. doi: 10.1371/journal.pone.0072402 (PMC3749139; doi:10.1371/journal.pone.0072402)
Supplement: File S1 — PDB file of the final model with all its components: NS3PRO, NS2BCF, glycine linker and NDL inhibitor. For displaying it in a molecular viewing program (such as VMD), please copy the content of the. doc file, paste it into a new document in a simple text editor (MS Windows notepad, for instance), and save it with a. pdb file extension. (DOC) [file pone.0072402.s003.doc]

TITLE DENGUE VIRUS PROTEASE IN COMPLEX WITH NDL INHIBITOR

COMPND MOL_ID: 1;

COMPND 2 MOLECULE: SERINE PROTEASE COFACTOR NS2B;

COMPND 3 CHAIN: A;

COMPND 4 MOL_ID: 2;

COMPND 5 MOLECULE: SERINE PROTEASE NS3;

COMPND 6 CHAIN: B;

COMPND 7 MOL_ID: 3;

COMPND 8 MOLECULE: NDL INHIBITOR;

COMPND 9 CHAIN: N;

KEYWDS SERINE PROTEASE, ER MEMBRANE, VIRAL PROTEIN, HYDROLASE-HYDROLASE

KEYWDS 2 INHIBITOR COMPLEX

AUTHOR H.DE-ALMEIDA

JRNL AUTH H.DE-ALMEIDA,I.M.D.BASTOS,B.M.RIBEIRO,B.MAIGRET,J.M.SANTANA

JRNL TITL NEW BINDING SITE CONFORMATIONS OF THE DENGUE VIRUS NS3 PRO-

JRNL TITL 2 TEASE ACCESSED BY MOLECULAR DYNAMICS SIMULATION

JRNL REF PLOS ONE 2013

CRYST1 0.000 0.000 0.000 90.00 90.00 90.00 P 1

ATOM 1 N SER A 48 12.574 -8.518 18.324 1.00 0.00

ATOM 2 HT1 SER A 48 12.115 -7.626 18.632 1.00 0.00

ATOM 3 HT2 SER A 48 12.767 -9.120 19.154 1.00 0.00

ATOM 4 HT3 SER A 48 13.447 -8.258 17.836 1.00 0.00

ATOM 5 CA SER A 48 11.635 -9.198 17.428 1.00 0.00

ATOM 6 HA SER A 48 12.123 -9.352 16.468 1.00 0.00

ATOM 7 C SER A 48 10.500 -8.192 17.232 1.00 0.00

ATOM 8 O SER A 48 10.668 -7.087 17.729 1.00 0.00

ATOM 9 CB SER A 48 11.227 -10.538 18.059 1.00 0.00

ATOM 10 HB1 SER A 48 11.978 -11.280 17.739 1.00 0.00

ATOM 11 HB2 SER A 48 10.250 -10.913 17.679 1.00 0.00

ATOM 12 OG SER A 48 11.247 -10.322 19.479 1.00 0.00

ATOM 13 HG SER A 48 10.497 -9.687 19.635 1.00 0.00

ATOM 14 N ALA A 49 9.416 -8.570 16.532 1.00 0.00

ATOM 15 HN ALA A 49 9.163 -9.494 16.235 1.00 0.00

ATOM 16 CA ALA A 49 8.320 -7.639 16.278 1.00 0.00

ATOM 17 HA ALA A 49 8.328 -6.870 17.057 1.00 0.00

ATOM 18 C ALA A 49 7.024 -8.439 16.391 1.00 0.00

ATOM 19 O ALA A 49 6.965 -9.623 16.070 1.00 0.00

ATOM 20 CB ALA A 49 8.424 -6.991 14.887 1.00 0.00

ATOM 21 HB1 ALA A 49 7.547 -7.176 14.262 1.00 0.00

ATOM 22 HB2 ALA A 49 8.514 -5.913 14.983 1.00 0.00

ATOM 23 HB3 ALA A 49 9.289 -7.341 14.330 1.00 0.00

ATOM 24 N ASP A 50 6.017 -7.703 16.872 1.00 0.00

ATOM 25 HN ASP A 50 6.286 -6.841 17.318 1.00 0.00

ATOM 26 CA ASP A 50 4.705 -8.235 17.224 1.00 0.00

ATOM 27 HA ASP A 50 4.858 -9.143 17.811 1.00 0.00

ATOM 28 C ASP A 50 3.887 -8.631 15.993 1.00 0.00

ATOM 29 O ASP A 50 3.257 -9.686 15.945 1.00 0.00

ATOM 30 CB ASP A 50 4.021 -7.174 18.098 1.00 0.00

ATOM 31 HB1 ASP A 50 2.958 -7.393 18.244 1.00 0.00

ATOM 32 HB2 ASP A 50 4.083 -6.168 17.673 1.00 0.00

ATOM 33 CG ASP A 50 4.746 -7.208 19.448 1.00 0.00

ATOM 34 OD1 ASP A 50 4.090 -7.475 20.452 1.00 0.00

ATOM 35 OD2 ASP A 50 5.971 -7.017 19.460 1.00 0.00

ATOM 36 N LEU A 51 3.952 -7.727 15.005 1.00 0.00

ATOM 37 HN LEU A 51 4.488 -6.888 15.125 1.00 0.00

ATOM 38 CA LEU A 51 3.336 -8.020 13.718 1.00 0.00

ATOM 39 HA LEU A 51 2.334 -8.376 13.935 1.00 0.00

ATOM 40 C LEU A 51 4.108 -9.165 13.028 1.00 0.00

ATOM 41 O LEU A 51 5.329 -9.274 13.140 1.00 0.00

ATOM 42 CB LEU A 51 3.265 -6.738 12.857 1.00 0.00

ATOM 43 HB1 LEU A 51 2.819 -6.972 11.889 1.00 0.00

ATOM 44 HB2 LEU A 51 4.276 -6.380 12.658 1.00 0.00

ATOM 45 CG LEU A 51 2.472 -5.585 13.519 1.00 0.00

ATOM 46 HG LEU A 51 3.010 -5.305 14.427 1.00 0.00

ATOM 47 CD1 LEU A 51 2.424 -4.303 12.654 1.00 0.00

ATOM 48 HD11 LEU A 51 3.299 -4.178 12.002 1.00 0.00

ATOM 49 HD12 LEU A 51 1.525 -4.289 12.022 1.00 0.00

ATOM 50 HD13 LEU A 51 2.383 -3.418 13.307 1.00 0.00

ATOM 51 CD2 LEU A 51 1.050 -6.004 13.949 1.00 0.00

ATOM 52 HD21 LEU A 51 0.783 -7.014 13.642 1.00 0.00

ATOM 53 HD22 LEU A 51 0.946 -5.939 15.033 1.00 0.00

ATOM 54 HD23 LEU A 51 0.282 -5.349 13.533 1.00 0.00

ATOM 55 N SER A 52 3.339 -10.027 12.336 1.00 0.00

ATOM 56 HN SER A 52 2.342 -9.925 12.325 1.00 0.00

ATOM 57 CA SER A 52 3.936 -11.097 11.552 1.00 0.00

ATOM 58 HA SER A 52 4.884 -10.724 11.156 1.00 0.00

ATOM 59 C SER A 52 3.026 -11.341 10.344 1.00 0.00

ATOM 60 O SER A 52 1.849 -10.982 10.352 1.00 0.00

ATOM 61 CB SER A 52 4.182 -12.345 12.442 1.00 0.00

ATOM 62 HB1 SER A 52 5.057 -12.138 13.063 1.00 0.00

ATOM 63 HB2 SER A 52 4.470 -13.187 11.809 1.00 0.00

ATOM 64 OG SER A 52 3.124 -12.769 13.308 1.00 0.00

ATOM 65 HG SER A 52 2.594 -11.990 13.522 1.00 0.00

ATOM 66 N LEU A 53 3.613 -11.946 9.301 1.00 0.00

ATOM 67 HN LEU A 53 4.606 -12.078 9.342 1.00 0.00

ATOM 68 CA LEU A 53 2.782 -12.226 8.130 1.00 0.00

ATOM 69 HA LEU A 53 1.761 -12.309 8.490 1.00 0.00

ATOM 70 C LEU A 53 3.066 -13.629 7.578 1.00 0.00

ATOM 71 O LEU A 53 4.043 -14.253 8.004 1.00 0.00

ATOM 72 CB LEU A 53 2.900 -11.083 7.106 1.00 0.00

ATOM 73 HB1 LEU A 53 2.444 -10.180 7.515 1.00 0.00

ATOM 74 HB2 LEU A 53 2.299 -11.336 6.237 1.00 0.00

ATOM 75 CG LEU A 53 4.347 -10.774 6.672 1.00 0.00

ATOM 76 HG LEU A 53 4.922 -11.688 6.793 1.00 0.00

ATOM 77 CD1 LEU A 53 4.438 -10.420 5.176 1.00 0.00

ATOM 78 HD11 LEU A 53 3.472 -10.434 4.670 1.00 0.00

ATOM 79 HD12 LEU A 53 4.869 -9.435 4.988 1.00 0.00

ATOM 80 HD13 LEU A 53 5.061 -11.142 4.649 1.00 0.00

ATOM 81 CD2 LEU A 53 5.016 -9.702 7.555 1.00 0.00

ATOM 82 HD21 LEU A 53 4.477 -9.521 8.489 1.00 0.00

ATOM 83 HD22 LEU A 53 6.038 -9.988 7.807 1.00 0.00

ATOM 84 HD23 LEU A 53 5.071 -8.736 7.044 1.00 0.00

ATOM 85 N GLU A 54 2.094 -14.062 6.728 1.00 0.00

ATOM 86 HN GLU A 54 1.444 -13.380 6.380 1.00 0.00

ATOM 87 CA GLU A 54 1.892 -15.398 6.136 1.00 0.00

ATOM 88 HA GLU A 54 2.862 -15.765 5.798 1.00 0.00

ATOM 89 C GLU A 54 0.951 -15.216 4.893 1.00 0.00

ATOM 90 O GLU A 54 0.051 -14.368 4.927 1.00 0.00

ATOM 91 CB GLU A 54 1.271 -16.360 7.190 1.00 0.00

ATOM 92 HB1 GLU A 54 0.599 -17.059 6.691 1.00 0.00

ATOM 93 HB2 GLU A 54 0.669 -15.784 7.892 1.00 0.00

ATOM 94 CG GLU A 54 2.312 -17.203 7.957 1.00 0.00

ATOM 95 HG1 GLU A 54 3.242 -16.641 8.049 1.00 0.00

ATOM 96 HG2 GLU A 54 2.546 -18.096 7.376 1.00 0.00

ATOM 97 CD GLU A 54 1.904 -17.638 9.369 1.00 0.00

ATOM 98 OE1 GLU A 54 0.997 -18.448 9.565 1.00 0.00

ATOM 99 OE2 GLU A 54 2.513 -17.154 10.320 1.00 0.00

ATOM 100 N LYS A 55 1.226 -16.031 3.831 1.00 0.00

ATOM 101 HN LYS A 55 1.979 -16.679 3.946 1.00 0.00

ATOM 102 CA LYS A 55 0.632 -15.924 2.476 1.00 0.00

ATOM 103 HA LYS A 55 0.534 -14.858 2.257 1.00 0.00

ATOM 104 C LYS A 55 -0.764 -16.591 2.422 1.00 0.00

ATOM 105 O LYS A 55 -1.018 -17.567 3.128 1.00 0.00

ATOM 106 CB LYS A 55 1.568 -16.609 1.436 1.00 0.00

ATOM 107 HB1 LYS A 55 1.469 -17.694 1.504 1.00 0.00

ATOM 108 HB2 LYS A 55 2.607 -16.389 1.684 1.00 0.00

ATOM 109 CG LYS A 55 1.315 -16.164 -0.023 1.00 0.00

ATOM 110 HG1 LYS A 55 1.485 -15.089 -0.070 1.00 0.00

ATOM 111 HG2 LYS A 55 0.268 -16.330 -0.280 1.00 0.00

ATOM 112 CD LYS A 55 2.185 -16.847 -1.109 1.00 0.00

ATOM 113 HD1 LYS A 55 1.866 -17.879 -1.261 1.00 0.00

ATOM 114 HD2 LYS A 55 3.227 -16.895 -0.785 1.00 0.00

ATOM 115 CE LYS A 55 2.112 -16.058 -2.435 1.00 0.00

ATOM 116 HE1 LYS A 55 2.627 -15.108 -2.277 1.00 0.00

ATOM 117 HE2 LYS A 55 1.071 -15.847 -2.677 1.00 0.00

ATOM 118 NZ LYS A 55 2.664 -16.637 -3.656 1.00 0.00

ATOM 119 HZ1 LYS A 55 3.432 -17.316 -3.485 1.00 0.00

ATOM 120 HZ2 LYS A 55 1.906 -17.042 -4.252 1.00 0.00

ATOM 121 HZ3 LYS A 55 3.053 -15.876 -4.245 1.00 0.00

ATOM 122 N ALA A 56 -1.655 -16.008 1.570 1.00 0.00

ATOM 123 HN ALA A 56 -1.352 -15.278 0.950 1.00 0.00

ATOM 124 CA ALA A 56 -3.066 -16.395 1.476 1.00 0.00

ATOM 125 HA ALA A 56 -3.203 -17.320 2.040 1.00 0.00

ATOM 126 C ALA A 56 -3.501 -16.698 0.017 1.00 0.00

ATOM 127 O ALA A 56 -4.175 -17.694 -0.234 1.00 0.00

ATOM 128 CB ALA A 56 -3.940 -15.286 2.091 1.00 0.00

ATOM 129 HB1 ALA A 56 -4.638 -14.871 1.362 1.00 0.00

ATOM 130 HB2 ALA A 56 -4.522 -15.665 2.929 1.00 0.00

ATOM 131 HB3 ALA A 56 -3.333 -14.457 2.453 1.00 0.00

ATOM 132 N ALA A 57 -3.129 -15.811 -0.931 1.00 0.00

ATOM 133 HN ALA A 57 -2.622 -14.975 -0.720 1.00 0.00

ATOM 134 CA ALA A 57 -3.552 -16.005 -2.323 1.00 0.00

ATOM 135 HA ALA A 57 -3.371 -17.055 -2.567 1.00 0.00

ATOM 136 C ALA A 57 -2.733 -15.147 -3.301 1.00 0.00

ATOM 137 O ALA A 57 -2.163 -14.112 -2.968 1.00 0.00

ATOM 138 CB ALA A 57 -5.041 -15.653 -2.506 1.00 0.00

ATOM 139 HB1 ALA A 57 -5.442 -15.221 -1.595 1.00 0.00

ATOM 140 HB2 ALA A 57 -5.196 -14.910 -3.293 1.00 0.00

ATOM 141 HB3 ALA A 57 -5.634 -16.531 -2.764 1.00 0.00

ATOM 142 N GLU A 58 -2.779 -15.670 -4.547 1.00 0.00

ATOM 143 HN GLU A 58 -3.259 -16.538 -4.695 1.00 0.00

ATOM 144 CA GLU A 58 -2.163 -15.025 -5.698 1.00 0.00

ATOM 145 HA GLU A 58 -1.227 -14.582 -5.350 1.00 0.00

ATOM 146 C GLU A 58 -3.069 -13.894 -6.280 1.00 0.00

ATOM 147 O GLU A 58 -4.288 -13.833 -6.098 1.00 0.00

ATOM 148 CB GLU A 58 -1.834 -16.100 -6.754 1.00 0.00

ATOM 149 HB1 GLU A 58 -1.142 -15.681 -7.490 1.00 0.00

ATOM 150 HB2 GLU A 58 -2.751 -16.366 -7.291 1.00 0.00

ATOM 151 CG GLU A 58 -1.222 -17.405 -6.195 1.00 0.00

ATOM 152 HG1 GLU A 58 -0.965 -18.068 -7.025 1.00 0.00

ATOM 153 HG2 GLU A 58 -1.960 -17.945 -5.601 1.00 0.00

ATOM 154 CD GLU A 58 0.053 -17.216 -5.356 1.00 0.00

ATOM 155 OE1 GLU A 58 0.077 -17.621 -4.200 1.00 0.00

ATOM 156 OE2 GLU A 58 1.027 -16.667 -5.844 1.00 0.00

ATOM 157 N VAL A 59 -2.328 -13.017 -6.977 1.00 0.00

ATOM 158 HN VAL A 59 -1.366 -13.243 -7.134 1.00 0.00

ATOM 159 CA VAL A 59 -2.861 -11.790 -7.542 1.00 0.00

ATOM 160 HA VAL A 59 -3.590 -11.399 -6.833 1.00 0.00

ATOM 161 C VAL A 59 -3.625 -12.062 -8.861 1.00 0.00

ATOM 162 O VAL A 59 -3.170 -11.741 -9.961 1.00 0.00

ATOM 163 CB VAL A 59 -1.689 -10.785 -7.671 1.00 0.00

ATOM 164 HB VAL A 59 -2.099 -9.861 -8.085 1.00 0.00

ATOM 165 CG1 VAL A 59 -1.131 -10.427 -6.275 1.00 0.00

ATOM 166 HG11 VAL A 59 -1.714 -10.874 -5.469 1.00 0.00

ATOM 167 HG12 VAL A 59 -0.100 -10.753 -6.129 1.00 0.00

ATOM 168 HG13 VAL A 59 -1.157 -9.351 -6.114 1.00 0.00

ATOM 169 CG2 VAL A 59 -0.561 -11.248 -8.627 1.00 0.00

ATOM 170 HG21 VAL A 59 -0.825 -12.142 -9.194 1.00 0.00

ATOM 171 HG22 VAL A 59 -0.341 -10.466 -9.356 1.00 0.00

ATOM 172 HG23 VAL A 59 0.371 -11.478 -8.106 1.00 0.00

ATOM 173 N SER A 60 -4.808 -12.656 -8.679 1.00 0.00

ATOM 174 HN SER A 60 -5.059 -13.133 -7.832 1.00 0.00

ATOM 175 CA SER A 60 -5.584 -13.033 -9.849 1.00 0.00

ATOM 176 HA SER A 60 -5.086 -12.723 -10.771 1.00 0.00

ATOM 177 C SER A 60 -6.982 -12.365 -9.813 1.00 0.00

ATOM 178 O SER A 60 -7.363 -11.726 -8.824 1.00 0.00

ATOM 179 CB SER A 60 -5.584 -14.552 -9.798 1.00 0.00

ATOM 180 HB1 SER A 60 -4.569 -14.915 -9.988 1.00 0.00

ATOM 181 HB2 SER A 60 -6.162 -15.000 -10.621 1.00 0.00

ATOM 182 OG SER A 60 -5.980 -14.919 -8.467 1.00 0.00

ATOM 183 HG SER A 60 -6.728 -15.554 -8.496 1.00 0.00

ATOM 184 N TRP A 61 -7.580 -12.495 -11.028 1.00 0.00

ATOM 185 HN TRP A 61 -7.127 -13.038 -11.737 1.00 0.00

ATOM 186 CA TRP A 61 -8.944 -12.127 -11.411 1.00 0.00

ATOM 187 HA TRP A 61 -9.416 -11.612 -10.571 1.00 0.00

ATOM 188 C TRP A 61 -9.662 -13.459 -11.744 1.00 0.00

ATOM 189 O TRP A 61 -9.283 -14.138 -12.703 1.00 0.00

ATOM 190 CB TRP A 61 -8.887 -11.238 -12.686 1.00 0.00

ATOM 191 HB1 TRP A 61 -8.314 -11.739 -13.466 1.00 0.00

ATOM 192 HB2 TRP A 61 -8.368 -10.301 -12.482 1.00 0.00

ATOM 193 CG TRP A 61 -10.262 -10.943 -13.267 1.00 0.00

ATOM 194 CD1 TRP A 61 -11.070 -11.827 -13.994 1.00 0.00

ATOM 195 HD1 TRP A 61 -10.818 -12.853 -14.223 1.00 0.00

ATOM 196 NE1 TRP A 61 -12.213 -11.197 -14.365 1.00 0.00

ATOM 197 HE1 TRP A 61 -12.979 -11.593 -14.837 1.00 0.00

ATOM 198 CE2 TRP A 61 -12.207 -9.891 -13.899 1.00 0.00

ATOM 199 CD2 TRP A 61 -10.981 -9.695 -13.218 1.00 0.00

ATOM 200 CE3 TRP A 61 -10.702 -8.482 -12.651 1.00 0.00

ATOM 201 HE3 TRP A 61 -9.771 -8.336 -12.124 1.00 0.00

ATOM 202 CZ3 TRP A 61 -11.611 -7.433 -12.775 1.00 0.00

ATOM 203 HZ3 TRP A 61 -11.383 -6.479 -12.339 1.00 0.00

ATOM 204 CH2 TRP A 61 -12.821 -7.622 -13.445 1.00 0.00

ATOM 205 HH2 TRP A 61 -13.546 -6.820 -13.529 1.00 0.00

ATOM 206 CZ2 TRP A 61 -13.102 -8.861 -14.018 1.00 0.00

ATOM 207 HZ2 TRP A 61 -14.045 -9.026 -14.525 1.00 0.00

ATOM 208 N GLU A 62 -10.690 -13.758 -10.938 1.00 0.00

ATOM 209 HN GLU A 62 -11.012 -13.065 -10.289 1.00 0.00

ATOM 210 CA GLU A 62 -11.431 -15.004 -11.118 1.00 0.00

ATOM 211 HA GLU A 62 -10.865 -15.667 -11.779 1.00 0.00

ATOM 212 C GLU A 62 -12.776 -14.629 -11.757 1.00 0.00

ATOM 213 O GLU A 62 -13.573 -13.915 -11.159 1.00 0.00

ATOM 214 CB GLU A 62 -11.643 -15.697 -9.762 1.00 0.00

ATOM 215 HB1 GLU A 62 -12.023 -16.704 -9.945 1.00 0.00

ATOM 216 HB2 GLU A 62 -12.407 -15.151 -9.197 1.00 0.00

ATOM 217 CG GLU A 62 -10.387 -15.780 -8.878 1.00 0.00

ATOM 218 HG1 GLU A 62 -10.558 -16.434 -8.023 1.00 0.00

ATOM 219 HG2 GLU A 62 -10.209 -14.778 -8.525 1.00 0.00

ATOM 220 CD GLU A 62 -9.096 -16.203 -9.563 1.00 0.00

ATOM 221 OE1 GLU A 62 -9.035 -17.210 -10.259 1.00 0.00

ATOM 222 OE2 GLU A 62 -8.158 -15.459 -9.393 1.00 0.00

ATOM 223 N GLU A 63 -12.968 -15.072 -13.016 1.00 0.00

ATOM 224 HN GLU A 63 -12.368 -15.767 -13.403 1.00 0.00

ATOM 225 CA GLU A 63 -14.036 -14.478 -13.820 1.00 0.00

ATOM 226 HA GLU A 63 -14.115 -13.440 -13.476 1.00 0.00

ATOM 227 C GLU A 63 -15.444 -15.078 -13.543 1.00 0.00

ATOM 228 O GLU A 63 -16.369 -14.744 -14.281 1.00 0.00

ATOM 229 CB GLU A 63 -13.652 -14.364 -15.331 1.00 0.00

ATOM 230 HB1 GLU A 63 -12.794 -13.698 -15.387 1.00 0.00

ATOM 231 HB2 GLU A 63 -14.453 -13.851 -15.863 1.00 0.00

ATOM 232 CG GLU A 63 -13.285 -15.645 -16.118 1.00 0.00

ATOM 233 HG1 GLU A 63 -14.175 -16.253 -16.285 1.00 0.00

ATOM 234 HG2 GLU A 63 -12.571 -16.241 -15.551 1.00 0.00

ATOM 235 CD GLU A 63 -12.644 -15.298 -17.487 1.00 0.00

ATOM 236 OE1 GLU A 63 -13.330 -15.227 -18.508 1.00 0.00

ATOM 237 OE2 GLU A 63 -11.441 -15.048 -17.536 1.00 0.00

ATOM 238 N GLU A 64 -15.585 -15.912 -12.490 1.00 0.00

ATOM 239 HN GLU A 64 -14.801 -16.361 -12.057 1.00 0.00

ATOM 240 CA GLU A 64 -16.908 -16.312 -11.995 1.00 0.00

ATOM 241 HA GLU A 64 -17.665 -15.934 -12.685 1.00 0.00

ATOM 242 C GLU A 64 -17.216 -15.660 -10.626 1.00 0.00

ATOM 243 O GLU A 64 -17.762 -16.286 -9.726 1.00 0.00

ATOM 244 CB GLU A 64 -17.052 -17.852 -11.946 1.00 0.00

ATOM 245 HB1 GLU A 64 -17.014 -18.228 -12.967 1.00 0.00

ATOM 246 HB2 GLU A 64 -18.054 -18.077 -11.578 1.00 0.00

ATOM 247 CG GLU A 64 -16.031 -18.635 -11.089 1.00 0.00

ATOM 248 HG1 GLU A 64 -16.533 -19.506 -10.656 1.00 0.00

ATOM 249 HG2 GLU A 64 -15.664 -18.032 -10.254 1.00 0.00

ATOM 250 CD GLU A 64 -14.859 -19.141 -11.946 1.00 0.00

ATOM 251 OE1 GLU A 64 -14.936 -20.280 -12.419 1.00 0.00

ATOM 252 OE2 GLU A 64 -13.904 -18.391 -12.158 1.00 0.00

ATOM 253 N ALA A 65 -16.840 -14.379 -10.480 1.00 0.00

ATOM 254 HN ALA A 65 -16.502 -13.914 -11.296 1.00 0.00

ATOM 255 CA ALA A 65 -16.944 -13.732 -9.160 1.00 0.00

ATOM 256 HA ALA A 65 -16.728 -14.489 -8.404 1.00 0.00

ATOM 257 C ALA A 65 -18.360 -13.188 -8.858 1.00 0.00

ATOM 258 O ALA A 65 -19.228 -13.050 -9.711 1.00 0.00

ATOM 259 CB ALA A 65 -15.900 -12.606 -9.039 1.00 0.00

ATOM 260 HB1 ALA A 65 -15.118 -12.709 -9.789 1.00 0.00

ATOM 261 HB2 ALA A 65 -16.343 -11.618 -9.157 1.00 0.00

ATOM 262 HB3 ALA A 65 -15.419 -12.642 -8.061 1.00 0.00

ATOM 263 N GLU A 66 -18.506 -12.830 -7.574 1.00 0.00

ATOM 264 HN GLU A 66 -17.708 -12.945 -6.984 1.00 0.00

ATOM 265 CA GLU A 66 -19.693 -12.144 -7.060 1.00 0.00

ATOM 266 HA GLU A 66 -20.566 -12.737 -7.345 1.00 0.00

ATOM 267 C GLU A 66 -19.769 -10.747 -7.729 1.00 0.00

ATOM 268 O GLU A 66 -18.802 -10.003 -7.767 1.00 0.00

ATOM 269 CB GLU A 66 -19.495 -12.111 -5.514 1.00 0.00

ATOM 270 HB1 GLU A 66 -18.573 -11.572 -5.291 1.00 0.00

ATOM 271 HB2 GLU A 66 -19.314 -13.145 -5.218 1.00 0.00

ATOM 272 CG GLU A 66 -20.614 -11.573 -4.590 1.00 0.00

ATOM 273 HG1 GLU A 66 -21.567 -12.019 -4.889 1.00 0.00

ATOM 274 HG2 GLU A 66 -20.721 -10.490 -4.678 1.00 0.00

ATOM 275 CD GLU A 66 -20.332 -11.968 -3.109 1.00 0.00

ATOM 276 OE1 GLU A 66 -21.093 -12.768 -2.552 1.00 0.00

ATOM 277 OE2 GLU A 66 -19.339 -11.522 -2.517 1.00 0.00

ATOM 278 N HSD A 67 -20.957 -10.376 -8.257 1.00 0.00

ATOM 279 HN HSD A 67 -21.679 -11.063 -8.323 1.00 0.00

ATOM 280 CA HSD A 67 -21.157 -8.989 -8.724 1.00 0.00

ATOM 281 HA HSD A 67 -20.193 -8.517 -8.921 1.00 0.00

ATOM 282 C HSD A 67 -21.891 -8.193 -7.623 1.00 0.00

ATOM 283 O HSD A 67 -23.015 -8.531 -7.256 1.00 0.00

ATOM 284 CB HSD A 67 -21.975 -8.958 -10.036 1.00 0.00

ATOM 285 HB1 HSD A 67 -22.411 -7.969 -10.187 1.00 0.00

ATOM 286 HB2 HSD A 67 -22.800 -9.667 -10.015 1.00 0.00

ATOM 287 CG HSD A 67 -21.112 -9.228 -11.255 1.00 0.00

ATOM 288 ND1 HSD A 67 -21.180 -8.486 -12.386 1.00 0.00

ATOM 289 CE1 HSD A 67 -20.247 -9.007 -13.216 1.00 0.00

ATOM 290 HE1 HSD A 67 -20.011 -8.604 -14.190 1.00 0.00

ATOM 291 NE2 HSD A 67 -19.595 -10.057 -12.674 1.00 0.00

ATOM 292 HE2 HSD A 67 -18.848 -10.576 -13.068 1.00 0.00

ATOM 293 CD2 HSD A 67 -20.121 -10.216 -11.434 1.00 0.00

ATOM 294 HD2 HSD A 67 -19.802 -10.960 -10.716 1.00 0.00

ATOM 295 N SER A 68 -21.239 -7.129 -7.120 1.00 0.00

ATOM 296 HN SER A 68 -20.278 -6.947 -7.323 1.00 0.00

ATOM 297 CA SER A 68 -21.879 -6.265 -6.141 1.00 0.00

ATOM 298 HA SER A 68 -22.903 -6.097 -6.480 1.00 0.00

ATOM 299 C SER A 68 -21.151 -4.898 -6.050 1.00 0.00

ATOM 300 O SER A 68 -19.943 -4.820 -5.863 1.00 0.00

ATOM 301 CB SER A 68 -21.855 -6.988 -4.776 1.00 0.00

ATOM 302 HB1 SER A 68 -20.843 -7.338 -4.544 1.00 0.00

ATOM 303 HB2 SER A 68 -22.465 -7.895 -4.825 1.00 0.00

ATOM 304 OG SER A 68 -22.342 -6.191 -3.703 1.00 0.00

ATOM 305 HG SER A 68 -21.964 -5.315 -3.777 1.00 0.00

ATOM 306 N GLY A 69 -21.984 -3.810 -6.072 1.00 0.00

ATOM 307 HN GLY A 69 -22.946 -3.933 -6.318 1.00 0.00

ATOM 308 CA GLY A 69 -21.501 -2.457 -5.702 1.00 0.00

ATOM 309 HA1 GLY A 69 -20.429 -2.394 -5.894 1.00 0.00

ATOM 310 HA2 GLY A 69 -21.688 -2.335 -4.633 1.00 0.00

ATOM 311 C GLY A 69 -22.214 -1.320 -6.482 1.00 0.00

ATOM 312 O GLY A 69 -22.593 -1.492 -7.638 1.00 0.00

ATOM 313 N THR A 70 -22.436 -0.166 -5.803 1.00 0.00

ATOM 314 HN THR A 70 -22.073 -0.103 -4.874 1.00 0.00

ATOM 315 CA THR A 70 -23.140 0.964 -6.429 1.00 0.00

ATOM 316 HA THR A 70 -23.585 0.614 -7.363 1.00 0.00

ATOM 317 C THR A 70 -22.156 2.104 -6.769 1.00 0.00

ATOM 318 O THR A 70 -21.560 2.711 -5.880 1.00 0.00

ATOM 319 CB THR A 70 -24.267 1.501 -5.499 1.00 0.00

ATOM 320 HB THR A 70 -24.756 2.316 -6.035 1.00 0.00

ATOM 321 OG1 THR A 70 -23.812 2.033 -4.263 1.00 0.00

ATOM 322 HG1 THR A 70 -23.098 1.459 -3.988 1.00 0.00

ATOM 323 CG2 THR A 70 -25.358 0.471 -5.180 1.00 0.00

ATOM 324 HG21 THR A 70 -25.182 -0.480 -5.682 1.00 0.00

ATOM 325 HG22 THR A 70 -25.416 0.278 -4.110 1.00 0.00

ATOM 326 HG23 THR A 70 -26.336 0.831 -5.499 1.00 0.00

ATOM 327 N SER A 71 -22.053 2.429 -8.071 1.00 0.00

ATOM 328 HN SER A 71 -22.548 1.963 -8.809 1.00 0.00

ATOM 329 CA SER A 71 -21.201 3.537 -8.464 1.00 0.00

ATOM 330 HA SER A 71 -20.305 3.506 -7.849 1.00 0.00

ATOM 331 C SER A 71 -21.947 4.881 -8.229 1.00 0.00

ATOM 332 O SER A 71 -22.736 5.320 -9.058 1.00 0.00

ATOM 333 CB SER A 71 -20.826 3.302 -9.942 1.00 0.00

ATOM 334 HB1 SER A 71 -20.267 2.364 -10.049 1.00 0.00

ATOM 335 HB2 SER A 71 -20.167 4.091 -10.311 1.00 0.00

ATOM 336 OG SER A 71 -21.972 3.209 -10.770 1.00 0.00

ATOM 337 HG SER A 71 -22.602 3.874 -10.482 1.00 0.00

ATOM 338 N HSD A 72 -21.653 5.517 -7.066 1.00 0.00

ATOM 339 HN HSD A 72 -21.100 5.014 -6.393 1.00 0.00

ATOM 340 CA HSD A 72 -22.180 6.866 -6.779 1.00 0.00

ATOM 341 HA HSD A 72 -22.616 7.254 -7.701 1.00 0.00

ATOM 342 C HSD A 72 -21.018 7.815 -6.415 1.00 0.00

ATOM 343 O HSD A 72 -19.890 7.355 -6.337 1.00 0.00

ATOM 344 CB HSD A 72 -23.274 6.796 -5.696 1.00 0.00

ATOM 345 HB1 HSD A 72 -24.005 6.037 -5.976 1.00 0.00

ATOM 346 HB2 HSD A 72 -23.801 7.748 -5.607 1.00 0.00

ATOM 347 CG HSD A 72 -22.732 6.429 -4.332 1.00 0.00

ATOM 348 ND1 HSD A 72 -22.492 5.164 -3.935 1.00 0.00

ATOM 349 CE1 HSD A 72 -22.057 5.250 -2.663 1.00 0.00

ATOM 350 HE1 HSD A 72 -21.756 4.399 -2.067 1.00 0.00

ATOM 351 NE2 HSD A 72 -22.014 6.530 -2.224 1.00 0.00

ATOM 352 HE2 HSD A 72 -21.743 6.810 -1.314 1.00 0.00

ATOM 353 CD2 HSD A 72 -22.432 7.298 -3.262 1.00 0.00

ATOM 354 HD2 HSD A 72 -22.524 8.380 -3.275 1.00 0.00

ATOM 355 N ASN A 73 -21.344 9.121 -6.194 1.00 0.00

ATOM 356 HN ASN A 73 -22.295 9.376 -6.025 1.00 0.00

ATOM 357 CA ASN A 73 -20.347 10.214 -6.188 1.00 0.00

ATOM 358 HA ASN A 73 -19.375 9.775 -5.983 1.00 0.00

ATOM 359 C ASN A 73 -20.698 11.261 -5.097 1.00 0.00

ATOM 360 O ASN A 73 -21.882 11.534 -4.906 1.00 0.00

ATOM 361 CB ASN A 73 -20.360 10.996 -7.522 1.00 0.00

ATOM 362 HB1 ASN A 73 -19.493 11.658 -7.567 1.00 0.00

ATOM 363 HB2 ASN A 73 -21.240 11.640 -7.555 1.00 0.00

ATOM 364 CG ASN A 73 -20.429 10.149 -8.800 1.00 0.00

ATOM 365 OD1 ASN A 73 -21.502 9.952 -9.356 1.00 0.00

ATOM 366 ND2 ASN A 73 -19.252 9.789 -9.295 1.00 0.00

ATOM 367 HD21 ASN A 73 -19.159 9.351 -10.186 1.00 0.00

ATOM 368 HD22 ASN A 73 -18.398 9.945 -8.791 1.00 0.00

ATOM 369 N ILE A 74 -19.641 11.825 -4.424 1.00 0.00

ATOM 370 HN ILE A 74 -18.690 11.547 -4.597 1.00 0.00

ATOM 371 CA ILE A 74 -19.772 12.967 -3.490 1.00 0.00

ATOM 372 HA ILE A 74 -20.529 13.609 -3.946 1.00 0.00

ATOM 373 C ILE A 74 -18.432 13.787 -3.471 1.00 0.00

ATOM 374 O ILE A 74 -17.578 13.508 -4.313 1.00 0.00

ATOM 375 CB ILE A 74 -20.271 12.455 -2.111 1.00 0.00

ATOM 376 HB ILE A 74 -20.876 11.572 -2.318 1.00 0.00

ATOM 377 CG1 ILE A 74 -21.224 13.461 -1.437 1.00 0.00

ATOM 378 HG11 ILE A 74 -21.951 13.812 -2.169 1.00 0.00

ATOM 379 HG12 ILE A 74 -20.662 14.336 -1.114 1.00 0.00

ATOM 380 CG2 ILE A 74 -19.158 12.006 -1.143 1.00 0.00

ATOM 381 HG21 ILE A 74 -18.193 11.865 -1.632 1.00 0.00

ATOM 382 HG22 ILE A 74 -19.019 12.728 -0.334 1.00 0.00

ATOM 383 HG23 ILE A 74 -19.425 11.058 -0.676 1.00 0.00

ATOM 384 CD1 ILE A 74 -21.989 12.894 -0.234 1.00 0.00

ATOM 385 HD11 ILE A 74 -21.623 11.904 0.039 1.00 0.00

ATOM 386 HD12 ILE A 74 -21.877 13.542 0.637 1.00 0.00

ATOM 387 HD13 ILE A 74 -23.055 12.816 -0.450 1.00 0.00

ATOM 388 N LEU A 75 -18.314 14.781 -2.540 1.00 0.00

ATOM 389 HN LEU A 75 -19.054 14.969 -1.887 1.00 0.00

ATOM 390 CA LEU A 75 -17.135 15.674 -2.381 1.00 0.00

ATOM 391 HA LEU A 75 -16.481 15.573 -3.247 1.00 0.00

ATOM 392 C LEU A 75 -16.324 15.324 -1.083 1.00 0.00

ATOM 393 O LEU A 75 -16.908 15.236 -0.001 1.00 0.00

ATOM 394 CB LEU A 75 -17.648 17.134 -2.242 1.00 0.00

ATOM 395 HB1 LEU A 75 -16.917 17.688 -1.666 1.00 0.00

ATOM 396 HB2 LEU A 75 -18.555 17.171 -1.636 1.00 0.00

ATOM 397 CG LEU A 75 -17.817 17.976 -3.524 1.00 0.00

ATOM 398 HG LEU A 75 -16.909 17.844 -4.117 1.00 0.00

ATOM 399 CD1 LEU A 75 -19.015 17.536 -4.387 1.00 0.00

ATOM 400 HD11 LEU A 75 -19.590 16.736 -3.923 1.00 0.00

ATOM 401 HD12 LEU A 75 -19.704 18.361 -4.572 1.00 0.00

ATOM 402 HD13 LEU A 75 -18.662 17.191 -5.357 1.00 0.00

ATOM 403 CD2 LEU A 75 -17.879 19.489 -3.175 1.00 0.00

ATOM 404 HD21 LEU A 75 -17.769 19.688 -2.105 1.00 0.00

ATOM 405 HD22 LEU A 75 -17.070 20.041 -3.658 1.00 0.00

ATOM 406 HD23 LEU A 75 -18.810 19.968 -3.474 1.00 0.00

ATOM 407 N VAL A 76 -14.976 15.144 -1.189 1.00 0.00

ATOM 408 HN VAL A 76 -14.540 15.269 -2.086 1.00 0.00

ATOM 409 CA VAL A 76 -14.170 14.742 -0.021 1.00 0.00

ATOM 410 HA VAL A 76 -14.697 15.127 0.855 1.00 0.00

ATOM 411 C VAL A 76 -12.711 15.324 0.044 1.00 0.00

ATOM 412 O VAL A 76 -11.819 14.932 -0.699 1.00 0.00

ATOM 413 CB VAL A 76 -14.073 13.192 0.063 1.00 0.00

ATOM 414 HB VAL A 76 -13.435 12.972 0.922 1.00 0.00

ATOM 415 CG1 VAL A 76 -15.413 12.488 0.362 1.00 0.00

ATOM 416 HG11 VAL A 76 -16.106 13.155 0.877 1.00 0.00

ATOM 417 HG12 VAL A 76 -15.910 12.144 -0.551 1.00 0.00

ATOM 418 HG13 VAL A 76 -15.245 11.624 1.013 1.00 0.00

ATOM 419 CG2 VAL A 76 -13.373 12.569 -1.165 1.00 0.00

ATOM 420 HG21 VAL A 76 -13.179 13.297 -1.950 1.00 0.00

ATOM 421 HG22 VAL A 76 -12.406 12.148 -0.891 1.00 0.00

ATOM 422 HG23 VAL A 76 -13.955 11.778 -1.629 1.00 0.00

ATOM 423 N GLU A 77 -12.562 16.099 1.135 1.00 0.00

ATOM 424 HN GLU A 77 -13.335 16.641 1.470 1.00 0.00

ATOM 425 CA GLU A 77 -11.306 16.602 1.700 1.00 0.00

ATOM 426 HA GLU A 77 -10.864 17.305 0.988 1.00 0.00

ATOM 427 C GLU A 77 -10.330 15.449 2.008 1.00 0.00

ATOM 428 O GLU A 77 -10.748 14.399 2.517 1.00 0.00

ATOM 429 CB GLU A 77 -11.733 17.283 3.019 1.00 0.00

ATOM 430 HB1 GLU A 77 -12.370 16.572 3.535 1.00 0.00

ATOM 431 HB2 GLU A 77 -12.371 18.140 2.798 1.00 0.00

ATOM 432 CG GLU A 77 -10.622 17.777 3.969 1.00 0.00

ATOM 433 HG1 GLU A 77 -10.417 18.834 3.780 1.00 0.00

ATOM 434 HG2 GLU A 77 -9.692 17.229 3.813 1.00 0.00

ATOM 435 CD GLU A 77 -11.032 17.655 5.445 1.00 0.00

ATOM 436 OE1 GLU A 77 -10.247 17.113 6.229 1.00 0.00

ATOM 437 OE2 GLU A 77 -12.143 18.046 5.837 1.00 0.00

ATOM 438 N VAL A 78 -9.033 15.696 1.698 1.00 0.00

ATOM 439 HN VAL A 78 -8.815 16.581 1.283 1.00 0.00

ATOM 440 CA VAL A 78 -7.922 14.838 2.131 1.00 0.00

ATOM 441 HA VAL A 78 -8.358 13.996 2.667 1.00 0.00

ATOM 442 C VAL A 78 -6.942 15.555 3.094 1.00 0.00

ATOM 443 O VAL A 78 -5.838 15.977 2.740 1.00 0.00

ATOM 444 CB VAL A 78 -7.103 14.287 0.947 1.00 0.00

ATOM 445 HB VAL A 78 -6.687 15.147 0.418 1.00 0.00

ATOM 446 CG1 VAL A 78 -5.885 13.439 1.405 1.00 0.00

ATOM 447 HG11 VAL A 78 -5.805 13.319 2.487 1.00 0.00

ATOM 448 HG12 VAL A 78 -5.872 12.440 0.978 1.00 0.00

ATOM 449 HG13 VAL A 78 -4.974 13.954 1.097 1.00 0.00

ATOM 450 CG2 VAL A 78 -7.994 13.557 -0.069 1.00 0.00

ATOM 451 HG21 VAL A 78 -8.977 13.319 0.334 1.00 0.00

ATOM 452 HG22 VAL A 78 -8.162 14.208 -0.924 1.00 0.00

ATOM 453 HG23 VAL A 78 -7.552 12.631 -0.433 1.00 0.00

ATOM 454 N GLN A 79 -7.340 15.401 4.382 1.00 0.00

ATOM 455 HN GLN A 79 -8.319 15.209 4.541 1.00 0.00

ATOM 456 CA GLN A 79 -6.429 15.913 5.409 1.00 0.00

ATOM 457 HA GLN A 79 -5.931 16.796 4.987 1.00 0.00

ATOM 458 C GLN A 79 -5.319 14.928 5.816 1.00 0.00

ATOM 459 O GLN A 79 -5.308 13.721 5.560 1.00 0.00

ATOM 460 CB GLN A 79 -7.203 16.393 6.636 1.00 0.00

ATOM 461 HB1 GLN A 79 -7.906 17.125 6.240 1.00 0.00

ATOM 462 HB2 GLN A 79 -6.550 16.957 7.300 1.00 0.00

ATOM 463 CG GLN A 79 -7.938 15.303 7.430 1.00 0.00

ATOM 464 HG1 GLN A 79 -8.423 14.609 6.739 1.00 0.00

ATOM 465 HG2 GLN A 79 -8.730 15.778 7.998 1.00 0.00

ATOM 466 CD GLN A 79 -7.039 14.526 8.425 1.00 0.00

ATOM 467 OE1 GLN A 79 -6.372 15.037 9.322 1.00 0.00

ATOM 468 NE2 GLN A 79 -7.087 13.208 8.247 1.00 0.00

ATOM 469 HE21 GLN A 79 -6.486 12.690 8.847 1.00 0.00

ATOM 470 HE22 GLN A 79 -7.657 12.808 7.527 1.00 0.00

ATOM 471 N ASP A 80 -4.422 15.608 6.529 1.00 0.00

ATOM 472 HN ASP A 80 -4.564 16.596 6.660 1.00 0.00

ATOM 473 CA ASP A 80 -3.084 15.200 6.882 1.00 0.00

ATOM 474 HA ASP A 80 -2.513 15.251 5.951 1.00 0.00

ATOM 475 C ASP A 80 -2.999 13.768 7.424 1.00 0.00

ATOM 476 O ASP A 80 -2.255 12.947 6.913 1.00 0.00

ATOM 477 CB ASP A 80 -2.529 16.212 7.898 1.00 0.00

ATOM 478 HB1 ASP A 80 -1.489 15.961 8.116 1.00 0.00

ATOM 479 HB2 ASP A 80 -3.069 16.194 8.845 1.00 0.00

ATOM 480 CG ASP A 80 -2.571 17.648 7.329 1.00 0.00

ATOM 481 OD1 ASP A 80 -3.665 18.227 7.238 1.00 0.00

ATOM 482 OD2 ASP A 80 -1.499 18.157 6.998 1.00 0.00

ATOM 483 N ASP A 81 -3.758 13.449 8.498 1.00 0.00

ATOM 484 HN ASP A 81 -4.418 14.145 8.792 1.00 0.00

ATOM 485 CA ASP A 81 -3.523 12.144 9.164 1.00 0.00

ATOM 486 HA ASP A 81 -2.444 12.002 9.188 1.00 0.00

ATOM 487 C ASP A 81 -4.139 10.938 8.381 1.00 0.00

ATOM 488 O ASP A 81 -4.119 9.781 8.794 1.00 0.00

ATOM 489 CB ASP A 81 -3.963 12.218 10.633 1.00 0.00

ATOM 490 HB1 ASP A 81 -5.048 12.271 10.700 1.00 0.00

ATOM 491 HB2 ASP A 81 -3.562 13.130 11.081 1.00 0.00

ATOM 492 CG ASP A 81 -3.466 11.040 11.493 1.00 0.00

ATOM 493 OD1 ASP A 81 -2.283 10.684 11.407 1.00 0.00

ATOM 494 OD2 ASP A 81 -4.264 10.504 12.267 1.00 0.00

ATOM 495 N GLY A 82 -4.738 11.276 7.214 1.00 0.00

ATOM 496 HN GLY A 82 -4.705 12.234 6.907 1.00 0.00

ATOM 497 CA GLY A 82 -4.934 10.255 6.176 1.00 0.00

ATOM 498 HA1 GLY A 82 -4.258 9.419 6.368 1.00 0.00

ATOM 499 HA2 GLY A 82 -4.626 10.716 5.232 1.00 0.00

ATOM 500 C GLY A 82 -6.368 9.716 6.006 1.00 0.00

ATOM 501 O GLY A 82 -6.718 9.224 4.945 1.00 0.00

ATOM 502 N THR A 83 -7.191 9.769 7.073 1.00 0.00

ATOM 503 HN THR A 83 -6.876 10.015 7.982 1.00 0.00

ATOM 504 CA THR A 83 -8.608 9.493 6.805 1.00 0.00

ATOM 505 HA THR A 83 -8.690 8.632 6.135 1.00 0.00

ATOM 506 C THR A 83 -9.228 10.740 6.171 1.00 0.00

ATOM 507 O THR A 83 -9.205 11.803 6.795 1.00 0.00

ATOM 508 CB THR A 83 -9.389 9.259 8.113 1.00 0.00

ATOM 509 HB THR A 83 -9.415 10.214 8.648 1.00 0.00

ATOM 510 OG1 THR A 83 -8.829 8.303 9.001 1.00 0.00

ATOM 511 HG1 THR A 83 -7.932 8.069 8.709 1.00 0.00

ATOM 512 CG2 THR A 83 -10.844 8.825 7.852 1.00 0.00

ATOM 513 HG21 THR A 83 -11.069 8.783 6.783 1.00 0.00

ATOM 514 HG22 THR A 83 -11.052 7.829 8.243 1.00 0.00

ATOM 515 HG23 THR A 83 -11.555 9.526 8.286 1.00 0.00

ATOM 516 N MET A 84 -9.783 10.577 4.956 1.00 0.00

ATOM 517 HN MET A 84 -9.721 9.653 4.570 1.00 0.00

ATOM 518 CA MET A 84 -10.539 11.667 4.315 1.00 0.00

ATOM 519 HA MET A 84 -9.850 12.498 4.149 1.00 0.00

ATOM 520 C MET A 84 -11.688 12.160 5.224 1.00 0.00

ATOM 521 O MET A 84 -12.047 11.559 6.241 1.00 0.00

ATOM 522 CB MET A 84 -11.133 11.212 2.965 1.00 0.00

ATOM 523 HB1 MET A 84 -11.813 11.976 2.577 1.00 0.00

ATOM 524 HB2 MET A 84 -11.721 10.305 3.093 1.00 0.00

ATOM 525 CG MET A 84 -10.087 10.988 1.872 1.00 0.00

ATOM 526 HG1 MET A 84 -9.418 11.845 1.796 1.00 0.00

ATOM 527 HG2 MET A 84 -10.585 10.919 0.902 1.00 0.00

ATOM 528 SD MET A 84 -9.128 9.489 2.146 1.00 0.00

ATOM 529 CE MET A 84 -8.264 9.421 0.566 1.00 0.00

ATOM 530 HE1 MET A 84 -8.493 10.289 -0.056 1.00 0.00

ATOM 531 HE2 MET A 84 -7.186 9.388 0.734 1.00 0.00

ATOM 532 HE3 MET A 84 -8.546 8.514 0.031 1.00 0.00

ATOM 533 N LYS A 85 -12.276 13.271 4.783 1.00 0.00

ATOM 534 HN LYS A 85 -11.840 13.729 4.001 1.00 0.00

ATOM 535 CA LYS A 85 -13.452 13.848 5.413 1.00 0.00

ATOM 536 HA LYS A 85 -13.876 13.121 6.105 1.00 0.00

ATOM 537 C LYS A 85 -14.461 14.113 4.291 1.00 0.00

ATOM 538 O LYS A 85 -14.161 13.912 3.116 1.00 0.00

ATOM 539 CB LYS A 85 -13.078 15.126 6.148 1.00 0.00

ATOM 540 HB1 LYS A 85 -13.919 15.614 6.637 1.00 0.00

ATOM 541 HB2 LYS A 85 -12.749 15.798 5.367 1.00 0.00

ATOM 542 CG LYS A 85 -11.911 15.009 7.120 1.00 0.00

ATOM 543 HG1 LYS A 85 -11.732 16.022 7.468 1.00 0.00

ATOM 544 HG2 LYS A 85 -11.011 14.657 6.602 1.00 0.00

ATOM 545 CD LYS A 85 -12.118 14.280 8.425 1.00 0.00

ATOM 546 HD1 LYS A 85 -12.436 13.251 8.256 1.00 0.00

ATOM 547 HD2 LYS A 85 -12.909 14.798 8.979 1.00 0.00

ATOM 548 CE LYS A 85 -10.767 14.386 9.146 1.00 0.00

ATOM 549 HE1 LYS A 85 -10.468 15.440 9.197 1.00 0.00

ATOM 550 HE2 LYS A 85 -9.971 13.852 8.612 1.00 0.00

ATOM 551 NZ LYS A 85 -10.833 13.870 10.495 1.00 0.00

ATOM 552 HZ1 LYS A 85 -11.844 13.785 10.726 1.00 0.00

ATOM 553 HZ2 LYS A 85 -10.351 12.949 10.477 1.00 0.00

ATOM 554 HZ3 LYS A 85 -10.336 14.538 11.123 1.00 0.00

ATOM 555 N ILE A 86 -15.703 14.463 4.740 1.00 0.00

ATOM 556 HN ILE A 86 -15.909 14.985 5.586 1.00 0.00

ATOM 557 CA ILE A 86 -16.798 14.484 3.767 1.00 0.00

ATOM 558 HA ILE A 86 -16.409 14.251 2.776 1.00 0.00

ATOM 559 C ILE A 86 -17.351 15.914 3.712 1.00 0.00

ATOM 560 O ILE A 86 -17.375 16.607 4.725 1.00 0.00

ATOM 561 CB ILE A 86 -17.846 13.387 4.097 1.00 0.00

ATOM 562 HB ILE A 86 -17.286 12.470 4.289 1.00 0.00

ATOM 563 CG1 ILE A 86 -18.740 13.076 2.877 1.00 0.00

ATOM 564 HG11 ILE A 86 -18.152 13.082 1.956 1.00 0.00

ATOM 565 HG12 ILE A 86 -19.484 13.862 2.750 1.00 0.00

ATOM 566 CG2 ILE A 86 -18.691 13.660 5.361 1.00 0.00

ATOM 567 HG21 ILE A 86 -18.344 14.533 5.914 1.00 0.00

ATOM 568 HG22 ILE A 86 -19.738 13.835 5.103 1.00 0.00

ATOM 569 HG23 ILE A 86 -18.667 12.809 6.045 1.00 0.00

ATOM 570 CD1 ILE A 86 -19.455 11.727 2.968 1.00 0.00

ATOM 571 HD11 ILE A 86 -19.287 11.231 3.927 1.00 0.00

ATOM 572 HD12 ILE A 86 -20.531 11.868 2.838 1.00 0.00

ATOM 573 HD13 ILE A 86 -19.124 11.050 2.176 1.00 0.00

ATOM 574 N LYS A 87 -17.820 16.222 2.489 1.00 0.00

ATOM 575 HN LYS A 87 -17.561 15.625 1.719 1.00 0.00

ATOM 576 CA LYS A 87 -18.539 17.415 2.057 1.00 0.00

ATOM 577 HA LYS A 87 -19.298 17.022 1.382 1.00 0.00

ATOM 578 C LYS A 87 -17.634 18.310 1.231 1.00 0.00

ATOM 579 O LYS A 87 -18.060 18.806 0.207 1.00 0.00

ATOM 580 CB LYS A 87 -19.219 18.250 3.145 1.00 0.00

ATOM 581 HB1 LYS A 87 -19.535 19.204 2.716 1.00 0.00

ATOM 582 HB2 LYS A 87 -18.502 18.528 3.914 1.00 0.00

ATOM 583 CG LYS A 87 -20.472 17.571 3.710 1.00 0.00

ATOM 584 HG1 LYS A 87 -20.218 16.625 4.184 1.00 0.00

ATOM 585 HG2 LYS A 87 -21.144 17.340 2.881 1.00 0.00

ATOM 586 CD LYS A 87 -21.204 18.495 4.691 1.00 0.00

ATOM 587 HD1 LYS A 87 -22.198 18.104 4.898 1.00 0.00

ATOM 588 HD2 LYS A 87 -21.344 19.463 4.203 1.00 0.00

ATOM 589 CE LYS A 87 -20.421 18.692 5.993 1.00 0.00

ATOM 590 HE1 LYS A 87 -19.370 18.692 5.742 1.00 0.00

ATOM 591 HE2 LYS A 87 -20.545 17.865 6.687 1.00 0.00

ATOM 592 NZ LYS A 87 -20.671 19.968 6.661 1.00 0.00

ATOM 593 HZ1 LYS A 87 -20.737 20.690 5.898 1.00 0.00

ATOM 594 HZ2 LYS A 87 -21.489 20.047 7.300 1.00 0.00

ATOM 595 HZ3 LYS A 87 -19.761 20.281 7.097 1.00 0.00

ATOM 596 N ASP A 88 -16.391 18.451 1.752 1.00 0.00

ATOM 597 HN ASP A 88 -16.083 18.101 2.642 1.00 0.00

ATOM 598 CA ASP A 88 -15.417 19.318 1.085 1.00 0.00

ATOM 599 HA ASP A 88 -14.606 19.352 1.818 1.00 0.00

ATOM 600 C ASP A 88 -16.019 20.760 0.963 1.00 0.00

ATOM 601 O ASP A 88 -16.674 21.153 -0.012 1.00 0.00

ATOM 602 CB ASP A 88 -14.961 18.639 -0.242 1.00 0.00

ATOM 603 HB1 ASP A 88 -15.344 19.186 -1.109 1.00 0.00

ATOM 604 HB2 ASP A 88 -15.387 17.640 -0.278 1.00 0.00

ATOM 605 CG ASP A 88 -13.433 18.461 -0.396 1.00 0.00

ATOM 606 OD1 ASP A 88 -12.981 17.833 -1.359 1.00 0.00

ATOM 607 OD2 ASP A 88 -12.724 18.864 0.509 1.00 0.00

ATOM 608 N GLU A 89 -15.866 21.436 2.134 1.00 0.00

ATOM 609 HN GLU A 89 -15.222 21.144 2.855 1.00 0.00

ATOM 610 CA GLU A 89 -16.563 22.697 2.374 1.00 0.00

ATOM 611 HA GLU A 89 -16.996 23.049 1.436 1.00 0.00

ATOM 612 C GLU A 89 -15.515 23.746 2.784 1.00 0.00

ATOM 613 O GLU A 89 -15.418 24.136 3.940 1.00 0.00

ATOM 614 CB GLU A 89 -17.710 22.458 3.389 1.00 0.00

ATOM 615 HB1 GLU A 89 -18.384 21.712 2.961 1.00 0.00

ATOM 616 HB2 GLU A 89 -18.280 23.385 3.474 1.00 0.00

ATOM 617 CG GLU A 89 -17.271 21.984 4.792 1.00 0.00

ATOM 618 HG1 GLU A 89 -16.897 22.837 5.363 1.00 0.00

ATOM 619 HG2 GLU A 89 -16.456 21.269 4.705 1.00 0.00

ATOM 620 CD GLU A 89 -18.372 21.343 5.618 1.00 0.00

ATOM 621 OE1 GLU A 89 -18.097 20.339 6.306 1.00 0.00

ATOM 622 OE2 GLU A 89 -19.503 21.837 5.578 1.00 0.00

ATOM 623 N GLU A 90 -14.714 24.075 1.743 1.00 0.00

ATOM 624 HN GLU A 90 -14.953 23.670 0.859 1.00 0.00

ATOM 625 CA GLU A 90 -13.564 24.994 1.729 1.00 0.00

ATOM 626 HA GLU A 90 -12.997 24.652 0.861 1.00 0.00

ATOM 627 C GLU A 90 -12.572 24.815 2.903 1.00 0.00

ATOM 628 O GLU A 90 -11.464 24.330 2.728 1.00 0.00

ATOM 629 CB GLU A 90 -13.973 26.452 1.414 1.00 0.00

ATOM 630 HB1 GLU A 90 -14.400 26.456 0.410 1.00 0.00

ATOM 631 HB2 GLU A 90 -13.073 27.066 1.354 1.00 0.00

ATOM 632 CG GLU A 90 -14.986 27.120 2.369 1.00 0.00

ATOM 633 HG1 GLU A 90 -14.511 27.328 3.327 1.00 0.00

ATOM 634 HG2 GLU A 90 -15.821 26.445 2.546 1.00 0.00

ATOM 635 CD GLU A 90 -15.596 28.442 1.814 1.00 0.00

ATOM 636 OE1 GLU A 90 -15.265 29.531 2.306 1.00 0.00

ATOM 637 OE2 GLU A 90 -16.450 28.362 0.920 1.00 0.00

ATOM 638 N ARG A 91 -13.006 25.192 4.106 1.00 0.00

ATOM 639 HN ARG A 91 -13.937 25.565 4.120 1.00 0.00

ATOM 640 CA ARG A 91 -12.276 24.874 5.323 1.00 0.00

ATOM 641 HA ARG A 91 -12.921 25.292 6.096 1.00 0.00

ATOM 642 C ARG A 91 -10.898 25.584 5.471 1.00 0.00

ATOM 643 O ARG A 91 -10.839 26.562 6.201 1.00 0.00

ATOM 644 CB ARG A 91 -12.224 23.362 5.549 1.00 0.00

ATOM 645 HB1 ARG A 91 -11.488 22.892 4.896 1.00 0.00

ATOM 646 HB2 ARG A 91 -13.195 22.926 5.306 1.00 0.00

ATOM 647 CG ARG A 91 -11.907 23.034 6.996 1.00 0.00

ATOM 648 HG1 ARG A 91 -12.659 23.493 7.639 1.00 0.00

ATOM 649 HG2 ARG A 91 -10.940 23.458 7.270 1.00 0.00

ATOM 650 CD ARG A 91 -11.872 21.534 7.209 1.00 0.00

ATOM 651 HD1 ARG A 91 -11.078 21.079 6.608 1.00 0.00

ATOM 652 HD2 ARG A 91 -12.830 21.072 6.957 1.00 0.00

ATOM 653 NE ARG A 91 -11.594 21.295 8.614 1.00 0.00

ATOM 654 HE ARG A 91 -11.520 22.176 9.096 1.00 0.00

ATOM 655 CZ ARG A 91 -11.346 20.038 9.013 1.00 0.00

ATOM 656 NH1 ARG A 91 -11.382 19.060 8.121 1.00 0.00

ATOM 657 HH11 ARG A 91 -11.667 19.278 7.166 1.00 0.00

ATOM 658 HH12 ARG A 91 -11.111 18.104 8.243 1.00 0.00

ATOM 659 NH2 ARG A 91 -11.084 19.784 10.292 1.00 0.00

ATOM 660 HH21 ARG A 91 -11.018 20.542 10.936 1.00 0.00

ATOM 661 HH22 ARG A 91 -10.946 18.838 10.572 1.00 0.00

ATOM 662 N ASP A 92 -9.846 25.036 4.825 1.00 0.00

ATOM 663 HN ASP A 92 -10.023 24.317 4.151 1.00 0.00

ATOM 664 CA ASP A 92 -8.485 25.608 4.933 1.00 0.00

ATOM 665 HA ASP A 92 -8.564 26.626 5.317 1.00 0.00

ATOM 666 C ASP A 92 -7.867 25.715 3.517 1.00 0.00

ATOM 667 O ASP A 92 -6.652 25.760 3.339 1.00 0.00

ATOM 668 CB ASP A 92 -7.603 24.757 5.891 1.00 0.00

ATOM 669 HB1 ASP A 92 -7.255 23.866 5.371 1.00 0.00

ATOM 670 HB2 ASP A 92 -8.198 24.445 6.749 1.00 0.00

ATOM 671 CG ASP A 92 -6.367 25.486 6.474 1.00 0.00

ATOM 672 OD1 ASP A 92 -5.299 24.865 6.584 1.00 0.00

ATOM 673 OD2 ASP A 92 -6.483 26.648 6.865 1.00 0.00

ATOM 674 N ASP A 93 -8.772 25.720 2.506 1.00 0.00

ATOM 675 HN ASP A 93 -9.736 25.539 2.700 1.00 0.00

ATOM 676 CA ASP A 93 -8.435 25.749 1.073 1.00 0.00

ATOM 677 HA ASP A 93 -9.382 25.986 0.584 1.00 0.00

ATOM 678 C ASP A 93 -8.029 24.336 0.603 1.00 0.00

ATOM 679 O ASP A 93 -8.632 23.768 -0.295 1.00 0.00

ATOM 680 CB ASP A 93 -7.418 26.852 0.692 1.00 0.00

ATOM 681 HB1 ASP A 93 -6.419 26.584 1.032 1.00 0.00

ATOM 682 HB2 ASP A 93 -7.699 27.775 1.202 1.00 0.00

ATOM 683 CG ASP A 93 -7.357 27.180 -0.819 1.00 0.00

ATOM 684 OD1 ASP A 93 -6.323 26.937 -1.454 1.00 0.00

ATOM 685 OD2 ASP A 93 -8.330 27.725 -1.344 1.00 0.00

ATOM 686 N THR A 94 -7.000 23.767 1.262 1.00 0.00

ATOM 687 HN THR A 94 -6.564 24.306 1.988 1.00 0.00

ATOM 688 CA THR A 94 -6.600 22.382 1.004 1.00 0.00

ATOM 689 HA THR A 94 -7.513 21.779 1.026 1.00 0.00

ATOM 690 C THR A 94 -5.634 21.909 2.112 1.00 0.00

ATOM 691 O THR A 94 -4.650 22.560 2.457 1.00 0.00

ATOM 692 CB THR A 94 -5.882 22.251 -0.364 1.00 0.00

ATOM 693 HB THR A 94 -5.111 23.028 -0.401 1.00 0.00

ATOM 694 OG1 THR A 94 -6.727 22.372 -1.485 1.00 0.00

ATOM 695 HG1 THR A 94 -7.277 23.163 -1.394 1.00 0.00

ATOM 696 CG2 THR A 94 -5.235 20.876 -0.637 1.00 0.00

ATOM 697 HG21 THR A 94 -5.426 20.160 0.156 1.00 0.00

ATOM 698 HG22 THR A 94 -5.623 20.417 -1.548 1.00 0.00

ATOM 699 HG23 THR A 94 -4.157 20.959 -0.755 1.00 0.00

ATOM 700 N LEU A 95 -5.931 20.660 2.562 1.00 0.00

ATOM 701 HN LEU A 95 -6.830 20.324 2.250 1.00 0.00

ATOM 702 CA LEU A 95 -5.251 19.938 3.653 1.00 0.00

ATOM 703 HA LEU A 95 -5.218 18.894 3.344 1.00 0.00

ATOM 704 C LEU A 95 -6.220 20.031 4.836 1.00 0.00

ATOM 705 O LEU A 95 -7.424 20.004 4.619 1.00 0.00

ATOM 706 CB LEU A 95 -3.803 20.378 4.009 1.00 0.00

ATOM 707 HB1 LEU A 95 -3.442 19.818 4.876 1.00 0.00

ATOM 708 HB2 LEU A 95 -3.806 21.424 4.311 1.00 0.00

ATOM 709 CG LEU A 95 -2.753 20.174 2.905 1.00 0.00

ATOM 710 HG LEU A 95 -3.126 20.660 2.004 1.00 0.00

ATOM 711 CD1 LEU A 95 -1.426 20.871 3.287 1.00 0.00

ATOM 712 HD11 LEU A 95 -1.502 21.394 4.242 1.00 0.00

ATOM 713 HD12 LEU A 95 -0.598 20.172 3.395 1.00 0.00

ATOM 714 HD13 LEU A 95 -1.137 21.611 2.544 1.00 0.00

ATOM 715 CD2 LEU A 95 -2.557 18.677 2.597 1.00 0.00

ATOM 716 HD21 LEU A 95 -3.190 18.052 3.225 1.00 0.00

ATOM 717 HD22 LEU A 95 -2.802 18.459 1.559 1.00 0.00

ATOM 718 HD23 LEU A 95 -1.537 18.346 2.776 1.00 0.00

ATOM 719 N GLY A 96 -5.661 20.133 6.056 1.00 0.00

ATOM 720 HN GLY A 96 -4.673 20.113 6.227 1.00 0.00

ATOM 721 CA GLY A 96 -6.541 20.311 7.189 1.00 0.00

ATOM 722 HA1 GLY A 96 -7.337 19.568 7.105 1.00 0.00

ATOM 723 HA2 GLY A 96 -6.953 21.321 7.148 1.00 0.00

ATOM 724 C GLY A 96 -5.727 20.101 8.455 1.00 0.00

ATOM 725 O GLY A 96 -4.936 20.920 8.904 1.00 0.00

ATOM 726 N GLY A 97 -5.949 18.911 9.046 1.00 0.00

ATOM 727 HN GLY A 97 -6.604 18.325 8.574 1.00 0.00

ATOM 728 CA GLY A 97 -5.006 18.404 10.041 1.00 0.00

ATOM 729 HA1 GLY A 97 -4.026 18.311 9.575 1.00 0.00

ATOM 730 HA2 GLY A 97 -5.350 17.411 10.328 1.00 0.00

ATOM 731 C GLY A 97 -4.845 19.237 11.316 1.00 0.00

ATOM 732 O GLY A 97 -4.051 18.859 12.169 1.00 0.00

ATOM 733 N GLY A 98 -5.654 20.302 11.473 1.00 0.00

ATOM 734 HN GLY A 98 -6.117 20.666 10.653 1.00 0.00

ATOM 735 CA GLY A 98 -5.612 21.065 12.727 1.00 0.00

ATOM 736 HA1 GLY A 98 -5.122 20.507 13.528 1.00 0.00

ATOM 737 HA2 GLY A 98 -6.637 21.262 13.038 1.00 0.00

ATOM 738 C GLY A 98 -4.904 22.396 12.520 1.00 0.00

ATOM 739 O GLY A 98 -5.454 23.449 12.821 1.00 0.00

ATOM 740 N GLY A 99 -3.674 22.303 11.992 1.00 0.00

ATOM 741 HN GLY A 99 -3.286 21.410 11.774 1.00 0.00

ATOM 742 CA GLY A 99 -3.002 23.516 11.546 1.00 0.00

ATOM 743 HA1 GLY A 99 -3.409 24.388 12.059 1.00 0.00

ATOM 744 HA2 GLY A 99 -3.198 23.618 10.477 1.00 0.00

ATOM 745 C GLY A 99 -1.497 23.503 11.801 1.00 0.00

ATOM 746 O GLY A 99 -0.810 24.441 11.422 1.00 0.00

ATOM 747 N SER A 100 -0.994 22.455 12.480 1.00 0.00

ATOM 748 HN SER A 100 -1.556 21.676 12.751 1.00 0.00

ATOM 749 CA SER A 100 0.454 22.376 12.663 1.00 0.00

ATOM 750 HA SER A 100 0.876 23.382 12.668 1.00 0.00

ATOM 751 C SER A 100 1.040 21.620 11.455 1.00 0.00

ATOM 752 O SER A 100 0.481 20.625 10.998 1.00 0.00

ATOM 753 CB SER A 100 0.770 21.727 14.022 1.00 0.00

ATOM 754 HB1 SER A 100 0.650 22.480 14.809 1.00 0.00

ATOM 755 HB2 SER A 100 1.817 21.395 14.073 1.00 0.00

ATOM 756 OG SER A 100 -0.106 20.641 14.279 1.00 0.00

ATOM 757 HG SER A 100 -0.058 20.083 13.488 1.00 0.00

ATOM 758 N GLY A 101 2.160 22.212 10.972 1.00 0.00

ATOM 759 HN GLY A 101 2.485 23.030 11.450 1.00 0.00

ATOM 760 CA GLY A 101 2.803 21.771 9.736 1.00 0.00

ATOM 761 HA1 GLY A 101 3.205 22.665 9.257 1.00 0.00

ATOM 762 HA2 GLY A 101 2.062 21.304 9.085 1.00 0.00

ATOM 763 C GLY A 101 3.953 20.801 9.999 1.00 0.00

ATOM 764 O GLY A 101 3.863 19.591 9.836 1.00 0.00

ATOM 765 N GLY A 102 5.091 21.398 10.403 1.00 0.00

ATOM 766 HN GLY A 102 5.182 22.390 10.369 1.00 0.00

ATOM 767 CA GLY A 102 6.236 20.564 10.749 1.00 0.00

ATOM 768 HA1 GLY A 102 5.910 19.798 11.454 1.00 0.00

ATOM 769 HA2 GLY A 102 6.968 21.205 11.241 1.00 0.00

ATOM 770 C GLY A 102 6.880 19.947 9.500 1.00 0.00

ATOM 771 O GLY A 102 7.840 20.484 8.964 1.00 0.00

ATOM 772 N GLY A 103 6.341 18.789 9.063 1.00 0.00

ATOM 773 HN GLY A 103 5.474 18.512 9.488 1.00 0.00

ATOM 774 CA GLY A 103 6.991 18.138 7.929 1.00 0.00

ATOM 775 HA1 GLY A 103 8.063 18.357 7.945 1.00 0.00

ATOM 776 HA2 GLY A 103 6.552 18.546 7.019 1.00 0.00

ATOM 777 C GLY A 103 6.861 16.622 7.978 1.00 0.00

ATOM 778 O GLY A 103 7.792 15.915 8.338 1.00 0.00

ATOM 779 N GLY A 104 5.686 16.149 7.527 1.00 0.00

ATOM 780 HN GLY A 104 4.957 16.785 7.263 1.00 0.00

ATOM 781 CA GLY A 104 5.407 14.710 7.557 1.00 0.00

ATOM 782 HA1 GLY A 104 4.799 14.551 8.449 1.00 0.00

ATOM 783 HA2 GLY A 104 6.331 14.134 7.645 1.00 0.00

ATOM 784 C GLY A 104 4.593 14.223 6.348 1.00 0.00

ATOM 785 O GLY A 104 3.444 13.838 6.500 1.00 0.00

TER 785 GLY A 104

ATOM 786 N SER B 1 5.218 14.239 5.142 1.00 0.00

ATOM 787 HN SER B 1 6.126 14.660 5.125 1.00 0.00

ATOM 788 CA SER B 1 4.467 13.844 3.927 1.00 0.00

ATOM 789 HA SER B 1 3.408 13.977 4.153 1.00 0.00

ATOM 790 C SER B 1 4.666 12.342 3.518 1.00 0.00

ATOM 791 O SER B 1 3.783 11.688 2.962 1.00 0.00

ATOM 792 CB SER B 1 4.797 14.833 2.791 1.00 0.00

ATOM 793 HB1 SER B 1 4.786 15.847 3.214 1.00 0.00

ATOM 794 HB2 SER B 1 5.800 14.657 2.373 1.00 0.00

ATOM 795 OG SER B 1 3.803 14.772 1.774 1.00 0.00

ATOM 796 HG SER B 1 4.026 15.365 1.052 1.00 0.00

ATOM 797 N GLY B 2 5.898 11.883 3.826 1.00 0.00

ATOM 798 HN GLY B 2 6.542 12.490 4.294 1.00 0.00

ATOM 799 CA GLY B 2 6.284 10.485 3.620 1.00 0.00

ATOM 800 HA1 GLY B 2 6.313 10.285 2.549 1.00 0.00

ATOM 801 HA2 GLY B 2 5.552 9.848 4.114 1.00 0.00

ATOM 802 C GLY B 2 7.662 10.343 4.250 1.00 0.00

ATOM 803 O GLY B 2 8.262 11.388 4.490 1.00 0.00

ATOM 804 N VAL B 3 8.141 9.102 4.536 1.00 0.00

ATOM 805 HN VAL B 3 7.708 8.245 4.239 1.00 0.00

ATOM 806 CA VAL B 3 9.403 8.844 5.254 1.00 0.00

ATOM 807 HA VAL B 3 10.047 9.720 5.144 1.00 0.00

ATOM 808 C VAL B 3 10.066 7.611 4.591 1.00 0.00

ATOM 809 O VAL B 3 9.366 6.651 4.273 1.00 0.00

ATOM 810 CB VAL B 3 9.110 8.549 6.752 1.00 0.00

ATOM 811 HB VAL B 3 8.395 7.725 6.777 1.00 0.00

ATOM 812 CG1 VAL B 3 10.336 8.033 7.550 1.00 0.00

ATOM 813 HG11 VAL B 3 11.279 8.103 7.010 1.00 0.00

ATOM 814 HG12 VAL B 3 10.495 8.564 8.487 1.00 0.00

ATOM 815 HG13 VAL B 3 10.207 6.986 7.826 1.00 0.00

ATOM 816 CG2 VAL B 3 8.413 9.726 7.471 1.00 0.00

ATOM 817 HG21 VAL B 3 8.332 10.614 6.849 1.00 0.00

ATOM 818 HG22 VAL B 3 7.410 9.447 7.796 1.00 0.00

ATOM 819 HG23 VAL B 3 8.950 10.038 8.367 1.00 0.00

ATOM 820 N LEU B 4 11.396 7.748 4.450 1.00 0.00

ATOM 821 HN LEU B 4 11.897 8.451 4.949 1.00 0.00

ATOM 822 CA LEU B 4 12.223 6.806 3.710 1.00 0.00

ATOM 823 HA LEU B 4 11.594 6.156 3.108 1.00 0.00

ATOM 824 C LEU B 4 13.057 5.975 4.706 1.00 0.00

ATOM 825 O LEU B 4 13.664 6.586 5.575 1.00 0.00

ATOM 826 CB LEU B 4 13.197 7.671 2.867 1.00 0.00

ATOM 827 HB1 LEU B 4 13.873 7.034 2.307 1.00 0.00

ATOM 828 HB2 LEU B 4 13.850 8.220 3.549 1.00 0.00

ATOM 829 CG LEU B 4 12.533 8.718 1.942 1.00 0.00

ATOM 830 HG LEU B 4 11.942 9.383 2.573 1.00 0.00

ATOM 831 CD1 LEU B 4 13.582 9.613 1.249 1.00 0.00

ATOM 832 HD11 LEU B 4 14.568 9.546 1.715 1.00 0.00

ATOM 833 HD12 LEU B 4 13.724 9.354 0.198 1.00 0.00

ATOM 834 HD13 LEU B 4 13.287 10.664 1.281 1.00 0.00

ATOM 835 CD2 LEU B 4 11.558 8.091 0.928 1.00 0.00

ATOM 836 HD21 LEU B 4 11.375 7.039 1.141 1.00 0.00

ATOM 837 HD22 LEU B 4 10.588 8.581 0.969 1.00 0.00

ATOM 838 HD23 LEU B 4 11.924 8.139 -0.096 1.00 0.00

ATOM 839 N TRP B 5 13.136 4.623 4.522 1.00 0.00

ATOM 840 HN TRP B 5 12.326 4.103 4.805 1.00 0.00

ATOM 841 CA TRP B 5 14.398 3.944 4.114 1.00 0.00

ATOM 842 HA TRP B 5 14.442 4.080 3.032 1.00 0.00

ATOM 843 C TRP B 5 14.465 2.403 4.419 1.00 0.00

ATOM 844 O TRP B 5 14.656 1.990 5.557 1.00 0.00

ATOM 845 CB TRP B 5 15.687 4.566 4.719 1.00 0.00

ATOM 846 HB1 TRP B 5 15.731 4.403 5.799 1.00 0.00

ATOM 847 HB2 TRP B 5 15.738 5.642 4.547 1.00 0.00

ATOM 848 CG TRP B 5 16.887 3.932 4.037 1.00 0.00

ATOM 849 CD1 TRP B 5 17.377 4.301 2.783 1.00 0.00

ATOM 850 HD1 TRP B 5 16.993 5.128 2.203 1.00 0.00

ATOM 851 NE1 TRP B 5 18.383 3.465 2.431 1.00 0.00

ATOM 852 HE1 TRP B 5 18.872 3.527 1.587 1.00 0.00

ATOM 853 CE2 TRP B 5 18.593 2.522 3.430 1.00 0.00

ATOM 854 CD2 TRP B 5 17.681 2.802 4.476 1.00 0.00

ATOM 855 CE3 TRP B 5 17.674 2.015 5.606 1.00 0.00

ATOM 856 HE3 TRP B 5 16.989 2.218 6.418 1.00 0.00

ATOM 857 CZ3 TRP B 5 18.569 0.949 5.726 1.00 0.00

ATOM 858 HZ3 TRP B 5 18.569 0.323 6.606 1.00 0.00

ATOM 859 CH2 TRP B 5 19.456 0.674 4.686 1.00 0.00

ATOM 860 HH2 TRP B 5 20.144 -0.155 4.737 1.00 0.00

ATOM 861 CZ2 TRP B 5 19.464 1.474 3.546 1.00 0.00

ATOM 862 HZ2 TRP B 5 20.128 1.232 2.733 1.00 0.00

ATOM 863 N ASP B 6 14.482 1.592 3.314 1.00 0.00

ATOM 864 HN ASP B 6 14.259 1.909 2.389 1.00 0.00

ATOM 865 CA ASP B 6 15.049 0.224 3.371 1.00 0.00

ATOM 866 HA ASP B 6 15.841 0.251 4.126 1.00 0.00

ATOM 867 C ASP B 6 15.700 -0.144 2.003 1.00 0.00

ATOM 868 O ASP B 6 15.606 0.588 1.027 1.00 0.00

ATOM 869 CB ASP B 6 13.992 -0.839 3.739 1.00 0.00

ATOM 870 HB1 ASP B 6 13.342 -0.995 2.880 1.00 0.00

ATOM 871 HB2 ASP B 6 13.383 -0.508 4.580 1.00 0.00

ATOM 872 CG ASP B 6 14.609 -2.193 4.132 1.00 0.00

ATOM 873 OD1 ASP B 6 14.019 -3.228 3.830 1.00 0.00

ATOM 874 OD2 ASP B 6 15.709 -2.201 4.684 1.00 0.00

ATOM 875 N THR B 7 16.377 -1.299 1.997 1.00 0.00

ATOM 876 HN THR B 7 16.304 -1.847 2.829 1.00 0.00

ATOM 877 CA THR B 7 17.193 -1.827 0.919 1.00 0.00

ATOM 878 HA THR B 7 17.662 -0.974 0.427 1.00 0.00

ATOM 879 C THR B 7 16.377 -2.748 -0.064 1.00 0.00

ATOM 880 O THR B 7 15.716 -3.684 0.363 1.00 0.00

ATOM 881 CB THR B 7 18.204 -2.754 1.632 1.00 0.00

ATOM 882 HB THR B 7 17.587 -3.529 2.120 1.00 0.00

ATOM 883 OG1 THR B 7 19.082 -2.160 2.589 1.00 0.00

ATOM 884 HG1 THR B 7 18.589 -2.036 3.436 1.00 0.00

ATOM 885 CG2 THR B 7 19.170 -3.461 0.687 1.00 0.00

ATOM 886 HG21 THR B 7 18.858 -3.445 -0.355 1.00 0.00

ATOM 887 HG22 THR B 7 20.128 -2.946 0.738 1.00 0.00

ATOM 888 HG23 THR B 7 19.300 -4.494 1.015 1.00 0.00

ATOM 889 N PRO B 8 16.532 -2.560 -1.407 1.00 0.00

ATOM 890 CA PRO B 8 15.709 -3.239 -2.435 1.00 0.00

ATOM 891 HA PRO B 8 14.684 -2.915 -2.281 1.00 0.00

ATOM 892 CD PRO B 8 17.454 -1.588 -1.976 1.00 0.00

ATOM 893 HD1 PRO B 8 18.412 -1.586 -1.460 1.00 0.00

ATOM 894 HD2 PRO B 8 17.003 -0.599 -1.887 1.00 0.00

ATOM 895 C PRO B 8 15.638 -4.797 -2.621 1.00 0.00

ATOM 896 O PRO B 8 14.961 -5.245 -3.530 1.00 0.00

ATOM 897 CB PRO B 8 16.200 -2.603 -3.751 1.00 0.00

ATOM 898 HB1 PRO B 8 15.511 -1.799 -4.016 1.00 0.00

ATOM 899 HB2 PRO B 8 16.229 -3.300 -4.591 1.00 0.00

ATOM 900 CG PRO B 8 17.575 -2.017 -3.435 1.00 0.00

ATOM 901 HG1 PRO B 8 17.857 -1.211 -4.108 1.00 0.00

ATOM 902 HG2 PRO B 8 18.323 -2.802 -3.527 1.00 0.00

ATOM 903 N SER B 9 16.349 -5.594 -1.781 1.00 0.00

ATOM 904 HN SER B 9 16.469 -5.195 -0.869 1.00 0.00

ATOM 905 CA SER B 9 16.367 -7.074 -1.953 1.00 0.00

ATOM 906 HA SER B 9 16.990 -7.394 -1.124 1.00 0.00

ATOM 907 C SER B 9 16.975 -7.605 -3.325 1.00 0.00

ATOM 908 O SER B 9 16.699 -7.053 -4.377 1.00 0.00

ATOM 909 CB SER B 9 14.961 -7.663 -1.685 1.00 0.00

ATOM 910 HB1 SER B 9 14.495 -7.197 -0.810 1.00 0.00

ATOM 911 HB2 SER B 9 15.049 -8.721 -1.428 1.00 0.00

ATOM 912 OG SER B 9 14.083 -7.593 -2.794 1.00 0.00

ATOM 913 HG SER B 9 13.983 -6.684 -3.098 1.00 0.00

ATOM 914 N PRO B 10 17.808 -8.701 -3.287 1.00 0.00

ATOM 915 CA PRO B 10 18.476 -9.232 -4.505 1.00 0.00

ATOM 916 HA PRO B 10 18.652 -8.414 -5.198 1.00 0.00

ATOM 917 CD PRO B 10 18.305 -9.307 -2.053 1.00 0.00

ATOM 918 HD1 PRO B 10 17.515 -9.870 -1.556 1.00 0.00

ATOM 919 HD2 PRO B 10 18.690 -8.542 -1.380 1.00 0.00

ATOM 920 C PRO B 10 17.875 -10.476 -5.289 1.00 0.00

ATOM 921 O PRO B 10 18.011 -11.610 -4.831 1.00 0.00

ATOM 922 CB PRO B 10 19.817 -9.683 -3.902 1.00 0.00

ATOM 923 HB1 PRO B 10 20.468 -8.810 -3.827 1.00 0.00

ATOM 924 HB2 PRO B 10 20.323 -10.433 -4.512 1.00 0.00

ATOM 925 CG PRO B 10 19.470 -10.190 -2.493 1.00 0.00

ATOM 926 HG1 PRO B 10 20.318 -10.148 -1.806 1.00 0.00

ATOM 927 HG2 PRO B 10 19.128 -11.225 -2.545 1.00 0.00

ATOM 928 N PRO B 11 17.277 -10.258 -6.505 1.00 0.00

ATOM 929 CA PRO B 11 16.658 -11.331 -7.308 1.00 0.00

ATOM 930 HA PRO B 11 16.012 -11.923 -6.670 1.00 0.00

ATOM 931 CD PRO B 11 17.196 -8.956 -7.162 1.00 0.00

ATOM 932 HD1 PRO B 11 18.193 -8.544 -7.308 1.00 0.00

ATOM 933 HD2 PRO B 11 16.598 -8.273 -6.561 1.00 0.00

ATOM 934 C PRO B 11 17.610 -12.333 -8.025 1.00 0.00

ATOM 935 O PRO B 11 18.752 -12.017 -8.356 1.00 0.00

ATOM 936 CB PRO B 11 15.811 -10.562 -8.325 1.00 0.00

ATOM 937 HB1 PRO B 11 14.823 -10.380 -7.897 1.00 0.00

ATOM 938 HB2 PRO B 11 15.683 -11.099 -9.266 1.00 0.00

ATOM 939 CG PRO B 11 16.534 -9.229 -8.515 1.00 0.00

ATOM 940 HG1 PRO B 11 15.871 -8.426 -8.842 1.00 0.00

ATOM 941 HG2 PRO B 11 17.304 -9.353 -9.277 1.00 0.00

ATOM 942 N GLU B 12 17.064 -13.561 -8.202 1.00 0.00

ATOM 943 HN GLU B 12 16.111 -13.706 -7.943 1.00 0.00

ATOM 944 CA GLU B 12 17.724 -14.589 -8.999 1.00 0.00

ATOM 945 HA GLU B 12 18.247 -14.079 -9.810 1.00 0.00

ATOM 946 C GLU B 12 16.640 -15.457 -9.675 1.00 0.00

ATOM 947 O GLU B 12 15.619 -14.939 -10.108 1.00 0.00

ATOM 948 CB GLU B 12 18.760 -15.355 -8.160 1.00 0.00

ATOM 949 HB1 GLU B 12 19.561 -14.669 -7.881 1.00 0.00

ATOM 950 HB2 GLU B 12 19.241 -16.108 -8.782 1.00 0.00

ATOM 951 CG GLU B 12 18.205 -15.997 -6.883 1.00 0.00

ATOM 952 HG1 GLU B 12 17.407 -16.706 -7.103 1.00 0.00

ATOM 953 HG2 GLU B 12 17.807 -15.231 -6.220 1.00 0.00

ATOM 954 CD GLU B 12 19.326 -16.740 -6.162 1.00 0.00

ATOM 955 OE1 GLU B 12 19.639 -17.855 -6.577 1.00 0.00

ATOM 956 OE2 GLU B 12 19.879 -16.186 -5.211 1.00 0.00

ATOM 957 N VAL B 13 16.916 -16.777 -9.781 1.00 0.00

ATOM 958 HN VAL B 13 17.654 -17.131 -9.203 1.00 0.00

ATOM 959 CA VAL B 13 16.153 -17.615 -10.712 1.00 0.00

ATOM 960 HA VAL B 13 15.757 -16.959 -11.492 1.00 0.00

ATOM 961 C VAL B 13 14.948 -18.302 -10.035 1.00 0.00

ATOM 962 O VAL B 13 13.823 -18.220 -10.527 1.00 0.00

ATOM 963 CB VAL B 13 17.108 -18.634 -11.367 1.00 0.00

ATOM 964 HB VAL B 13 17.571 -19.217 -10.567 1.00 0.00

ATOM 965 CG1 VAL B 13 16.381 -19.655 -12.275 1.00 0.00

ATOM 966 HG11 VAL B 13 15.297 -19.540 -12.282 1.00 0.00

ATOM 967 HG12 VAL B 13 16.699 -19.599 -13.313 1.00 0.00

ATOM 968 HG13 VAL B 13 16.576 -20.671 -11.933 1.00 0.00

ATOM 969 CG2 VAL B 13 18.254 -17.911 -12.106 1.00 0.00

ATOM 970 HG21 VAL B 13 18.166 -16.824 -12.062 1.00 0.00

ATOM 971 HG22 VAL B 13 19.218 -18.171 -11.668 1.00 0.00

ATOM 972 HG23 VAL B 13 18.296 -18.174 -13.161 1.00 0.00

ATOM 973 N GLU B 14 15.280 -19.024 -8.942 1.00 0.00

ATOM 974 HN GLU B 14 16.222 -19.075 -8.597 1.00 0.00

ATOM 975 CA GLU B 14 14.252 -19.448 -7.993 1.00 0.00

ATOM 976 HA GLU B 14 13.267 -19.272 -8.428 1.00 0.00

ATOM 977 C GLU B 14 14.475 -18.504 -6.808 1.00 0.00

ATOM 978 O GLU B 14 15.407 -18.666 -6.032 1.00 0.00

ATOM 979 CB GLU B 14 14.443 -20.931 -7.619 1.00 0.00

ATOM 980 HB1 GLU B 14 15.333 -21.034 -6.992 1.00 0.00

ATOM 981 HB2 GLU B 14 14.632 -21.518 -8.521 1.00 0.00

ATOM 982 CG GLU B 14 13.227 -21.554 -6.907 1.00 0.00

ATOM 983 HG1 GLU B 14 12.872 -20.916 -6.093 1.00 0.00

ATOM 984 HG2 GLU B 14 13.521 -22.505 -6.455 1.00 0.00

ATOM 985 CD GLU B 14 12.075 -21.812 -7.907 1.00 0.00

ATOM 986 OE1 GLU B 14 11.848 -22.984 -8.235 1.00 0.00

ATOM 987 OE2 GLU B 14 11.446 -20.857 -8.378 1.00 0.00

ATOM 988 N ARG B 15 13.689 -17.423 -6.826 1.00 0.00

ATOM 989 HN ARG B 15 12.773 -17.502 -7.223 1.00 0.00

ATOM 990 CA ARG B 15 14.266 -16.149 -6.399 1.00 0.00

ATOM 991 HA ARG B 15 15.235 -16.082 -6.897 1.00 0.00

ATOM 992 C ARG B 15 14.498 -16.073 -4.881 1.00 0.00

ATOM 993 O ARG B 15 13.794 -16.659 -4.074 1.00 0.00

ATOM 994 CB ARG B 15 13.401 -14.977 -6.884 1.00 0.00

ATOM 995 HB1 ARG B 15 13.951 -14.050 -6.726 1.00 0.00

ATOM 996 HB2 ARG B 15 12.483 -14.898 -6.298 1.00 0.00

ATOM 997 CG ARG B 15 13.066 -15.098 -8.374 1.00 0.00

ATOM 998 HG1 ARG B 15 13.801 -15.745 -8.851 1.00 0.00

ATOM 999 HG2 ARG B 15 13.145 -14.134 -8.878 1.00 0.00

ATOM 1000 CD ARG B 15 11.671 -15.668 -8.604 1.00 0.00

ATOM 1001 HD1 ARG B 15 10.940 -14.870 -8.757 1.00 0.00

ATOM 1002 HD2 ARG B 15 11.352 -16.298 -7.768 1.00 0.00

ATOM 1003 NE ARG B 15 11.702 -16.531 -9.775 1.00 0.00

ATOM 1004 HE ARG B 15 12.597 -16.757 -10.176 1.00 0.00

ATOM 1005 CZ ARG B 15 10.584 -17.146 -10.179 1.00 0.00

ATOM 1006 NH1 ARG B 15 9.429 -16.776 -9.629 1.00 0.00

ATOM 1007 HH11 ARG B 15 9.465 -16.082 -8.899 1.00 0.00

ATOM 1008 HH12 ARG B 15 8.542 -17.152 -9.865 1.00 0.00

ATOM 1009 NH2 ARG B 15 10.670 -18.111 -11.085 1.00 0.00

ATOM 1010 HH21 ARG B 15 11.580 -18.392 -11.406 1.00 0.00

ATOM 1011 HH22 ARG B 15 9.861 -18.586 -11.416 1.00 0.00

ATOM 1012 N ALA B 16 15.507 -15.253 -4.546 1.00 0.00

ATOM 1013 HN ALA B 16 16.017 -14.785 -5.265 1.00 0.00

ATOM 1014 CA ALA B 16 15.839 -14.997 -3.142 1.00 0.00

ATOM 1015 HA ALA B 16 15.557 -15.883 -2.570 1.00 0.00

ATOM 1016 C ALA B 16 15.002 -13.798 -2.612 1.00 0.00

ATOM 1017 O ALA B 16 15.465 -12.918 -1.882 1.00 0.00

ATOM 1018 CB ALA B 16 17.363 -14.826 -3.012 1.00 0.00

ATOM 1019 HB1 ALA B 16 17.631 -13.941 -2.438 1.00 0.00

ATOM 1020 HB2 ALA B 16 17.794 -15.694 -2.514 1.00 0.00

ATOM 1021 HB3 ALA B 16 17.848 -14.758 -3.986 1.00 0.00

ATOM 1022 N VAL B 17 13.733 -13.856 -3.048 1.00 0.00

ATOM 1023 HN VAL B 17 13.352 -14.701 -3.431 1.00 0.00

ATOM 1024 CA VAL B 17 12.754 -12.807 -2.905 1.00 0.00

ATOM 1025 HA VAL B 17 13.103 -12.127 -2.125 1.00 0.00

ATOM 1026 C VAL B 17 11.491 -13.495 -2.395 1.00 0.00

ATOM 1027 O VAL B 17 11.265 -14.682 -2.600 1.00 0.00

ATOM 1028 CB VAL B 17 12.643 -12.068 -4.258 1.00 0.00

ATOM 1029 HB VAL B 17 12.538 -12.807 -5.055 1.00 0.00

ATOM 1030 CG1 VAL B 17 11.410 -11.153 -4.415 1.00 0.00

ATOM 1031 HG11 VAL B 17 10.799 -11.087 -3.522 1.00 0.00

ATOM 1032 HG12 VAL B 17 11.678 -10.137 -4.694 1.00 0.00

ATOM 1033 HG13 VAL B 17 10.750 -11.525 -5.201 1.00 0.00

ATOM 1034 CG2 VAL B 17 13.964 -11.314 -4.517 1.00 0.00

ATOM 1035 HG21 VAL B 17 14.552 -11.184 -3.604 1.00 0.00

ATOM 1036 HG22 VAL B 17 14.586 -11.865 -5.218 1.00 0.00

ATOM 1037 HG23 VAL B 17 13.799 -10.313 -4.915 1.00 0.00

ATOM 1038 N LEU B 18 10.811 -12.669 -1.597 1.00 0.00

ATOM 1039 HN LEU B 18 11.009 -11.702 -1.739 1.00 0.00

ATOM 1040 CA LEU B 18 9.725 -13.096 -0.747 1.00 0.00

ATOM 1041 HA LEU B 18 9.970 -14.080 -0.340 1.00 0.00

ATOM 1042 C LEU B 18 8.486 -13.218 -1.664 1.00 0.00

ATOM 1043 O LEU B 18 8.011 -12.248 -2.253 1.00 0.00

ATOM 1044 CB LEU B 18 9.610 -12.091 0.431 1.00 0.00

ATOM 1045 HB1 LEU B 18 8.633 -12.231 0.890 1.00 0.00

ATOM 1046 HB2 LEU B 18 9.632 -11.068 0.050 1.00 0.00

ATOM 1047 CG LEU B 18 10.650 -12.262 1.581 1.00 0.00

ATOM 1048 HG LEU B 18 10.464 -13.243 2.021 1.00 0.00

ATOM 1049 CD1 LEU B 18 12.132 -12.257 1.140 1.00 0.00

ATOM 1050 HD11 LEU B 18 12.270 -11.744 0.184 1.00 0.00

ATOM 1051 HD12 LEU B 18 12.774 -11.732 1.858 1.00 0.00

ATOM 1052 HD13 LEU B 18 12.516 -13.279 1.040 1.00 0.00

ATOM 1053 CD2 LEU B 18 10.461 -11.241 2.725 1.00 0.00

ATOM 1054 HD21 LEU B 18 9.401 -11.113 3.030 1.00 0.00

ATOM 1055 HD22 LEU B 18 11.031 -11.606 3.604 1.00 0.00

ATOM 1056 HD23 LEU B 18 10.882 -10.263 2.418 1.00 0.00

ATOM 1057 N ASP B 19 8.074 -14.507 -1.733 1.00 0.00

ATOM 1058 HN ASP B 19 8.534 -15.134 -1.110 1.00 0.00

ATOM 1059 CA ASP B 19 7.174 -15.081 -2.739 1.00 0.00

ATOM 1060 HA ASP B 19 7.751 -15.144 -3.665 1.00 0.00

ATOM 1061 C ASP B 19 5.951 -14.174 -2.940 1.00 0.00

ATOM 1062 O ASP B 19 5.219 -13.892 -1.984 1.00 0.00

ATOM 1063 CB ASP B 19 6.797 -16.496 -2.232 1.00 0.00

ATOM 1064 HB1 ASP B 19 6.205 -16.427 -1.318 1.00 0.00

ATOM 1065 HB2 ASP B 19 7.708 -17.034 -1.970 1.00 0.00

ATOM 1066 CG ASP B 19 6.046 -17.420 -3.209 1.00 0.00

ATOM 1067 OD1 ASP B 19 4.927 -17.833 -2.901 1.00 0.00

ATOM 1068 OD2 ASP B 19 6.603 -17.791 -4.236 1.00 0.00

ATOM 1069 N ASP B 20 5.804 -13.695 -4.186 1.00 0.00

ATOM 1070 HN ASP B 20 6.428 -13.975 -4.909 1.00 0.00

ATOM 1071 CA ASP B 20 4.780 -12.710 -4.454 1.00 0.00

ATOM 1072 HA ASP B 20 4.936 -11.915 -3.725 1.00 0.00

ATOM 1073 C ASP B 20 3.402 -13.317 -4.227 1.00 0.00

ATOM 1074 O ASP B 20 3.078 -14.405 -4.655 1.00 0.00

ATOM 1075 CB ASP B 20 4.906 -12.133 -5.877 1.00 0.00

ATOM 1076 HB1 ASP B 20 4.061 -11.476 -6.095 1.00 0.00

ATOM 1077 HB2 ASP B 20 4.895 -12.930 -6.619 1.00 0.00

ATOM 1078 CG ASP B 20 6.185 -11.296 -6.000 1.00 0.00

ATOM 1079 OD1 ASP B 20 7.003 -11.521 -6.887 1.00 0.00

ATOM 1080 OD2 ASP B 20 6.328 -10.363 -5.212 1.00 0.00

ATOM 1081 N GLY B 21 2.599 -12.485 -3.558 1.00 0.00

ATOM 1082 HN GLY B 21 3.046 -11.682 -3.163 1.00 0.00

ATOM 1083 CA GLY B 21 1.191 -12.739 -3.331 1.00 0.00

ATOM 1084 HA1 GLY B 21 1.036 -13.798 -3.162 1.00 0.00

ATOM 1085 HA2 GLY B 21 0.654 -12.430 -4.229 1.00 0.00

ATOM 1086 C GLY B 21 0.737 -11.911 -2.135 1.00 0.00

ATOM 1087 O GLY B 21 1.351 -10.910 -1.784 1.00 0.00

ATOM 1088 N ILE B 22 -0.383 -12.353 -1.565 1.00 0.00

ATOM 1089 HN ILE B 22 -0.763 -13.250 -1.801 1.00 0.00

ATOM 1090 CA ILE B 22 -1.168 -11.529 -0.655 1.00 0.00

ATOM 1091 HA ILE B 22 -0.782 -10.510 -0.667 1.00 0.00

ATOM 1092 C ILE B 22 -1.040 -12.123 0.756 1.00 0.00

ATOM 1093 O ILE B 22 -1.107 -13.337 0.924 1.00 0.00

ATOM 1094 CB ILE B 22 -2.625 -11.572 -1.173 1.00 0.00

ATOM 1095 HB ILE B 22 -2.968 -12.606 -1.063 1.00 0.00

ATOM 1096 CG2 ILE B 22 -3.558 -10.652 -0.364 1.00 0.00

ATOM 1097 HG21 ILE B 22 -2.992 -10.037 0.334 1.00 0.00

ATOM 1098 HG22 ILE B 22 -4.121 -9.973 -1.009 1.00 0.00

ATOM 1099 HG23 ILE B 22 -4.280 -11.212 0.234 1.00 0.00

ATOM 1100 CG1 ILE B 22 -2.713 -11.251 -2.686 1.00 0.00

ATOM 1101 HG11 ILE B 22 -2.034 -11.881 -3.256 1.00 0.00

ATOM 1102 HG12 ILE B 22 -2.406 -10.226 -2.895 1.00 0.00

ATOM 1103 CD1 ILE B 22 -4.107 -11.509 -3.266 1.00 0.00

ATOM 1104 HD11 ILE B 22 -4.660 -12.188 -2.614 1.00 0.00

ATOM 1105 HD12 ILE B 22 -4.666 -10.577 -3.376 1.00 0.00

ATOM 1106 HD13 ILE B 22 -4.031 -11.984 -4.250 1.00 0.00

ATOM 1107 N TYR B 23 -0.856 -11.289 1.793 1.00 0.00

ATOM 1108 HN TYR B 23 -1.142 -10.339 1.652 1.00 0.00

ATOM 1109 CA TYR B 23 -0.383 -11.788 3.088 1.00 0.00

ATOM 1110 HA TYR B 23 -0.526 -12.865 3.135 1.00 0.00

ATOM 1111 C TYR B 23 -1.203 -11.126 4.204 1.00 0.00

ATOM 1112 O TYR B 23 -1.451 -9.929 4.179 1.00 0.00

ATOM 1113 CB TYR B 23 1.098 -11.452 3.328 1.00 0.00

ATOM 1114 HB1 TYR B 23 1.306 -11.596 4.385 1.00 0.00

ATOM 1115 HB2 TYR B 23 1.255 -10.399 3.158 1.00 0.00

ATOM 1116 CG TYR B 23 2.113 -12.276 2.570 1.00 0.00

ATOM 1117 CD1 TYR B 23 2.869 -13.214 3.260 1.00 0.00

ATOM 1118 HD1 TYR B 23 2.726 -13.331 4.320 1.00 0.00

ATOM 1119 CE1 TYR B 23 3.795 -14.008 2.606 1.00 0.00

ATOM 1120 HE1 TYR B 23 4.347 -14.778 3.114 1.00 0.00

ATOM 1121 CZ TYR B 23 4.007 -13.864 1.251 1.00 0.00

ATOM 1122 OH TYR B 23 4.955 -14.682 0.674 1.00 0.00

ATOM 1123 HH TYR B 23 5.032 -14.478 -0.262 1.00 0.00

ATOM 1124 CE2 TYR B 23 3.265 -12.924 0.540 1.00 0.00

ATOM 1125 HE2 TYR B 23 3.395 -12.802 -0.530 1.00 0.00

ATOM 1126 CD2 TYR B 23 2.329 -12.131 1.202 1.00 0.00

ATOM 1127 HD2 TYR B 23 1.776 -11.395 0.635 1.00 0.00

ATOM 1128 N ARG B 24 -1.578 -11.976 5.174 1.00 0.00

ATOM 1129 HN ARG B 24 -1.301 -12.926 5.034 1.00 0.00

ATOM 1130 CA ARG B 24 -2.186 -11.480 6.403 1.00 0.00

ATOM 1131 HA ARG B 24 -2.919 -10.716 6.137 1.00 0.00

ATOM 1132 C ARG B 24 -1.071 -10.881 7.300 1.00 0.00

ATOM 1133 O ARG B 24 -0.012 -11.493 7.406 1.00 0.00

ATOM 1134 CB ARG B 24 -2.861 -12.678 7.107 1.00 0.00

ATOM 1135 HB1 ARG B 24 -3.433 -12.319 7.963 1.00 0.00

ATOM 1136 HB2 ARG B 24 -2.096 -13.349 7.504 1.00 0.00

ATOM 1137 CG ARG B 24 -3.793 -13.494 6.184 1.00 0.00

ATOM 1138 HG1 ARG B 24 -3.204 -13.943 5.382 1.00 0.00

ATOM 1139 HG2 ARG B 24 -4.511 -12.829 5.708 1.00 0.00

ATOM 1140 CD ARG B 24 -4.528 -14.624 6.922 1.00 0.00

ATOM 1141 HD1 ARG B 24 -5.263 -14.263 7.646 1.00 0.00

ATOM 1142 HD2 ARG B 24 -3.798 -15.216 7.477 1.00 0.00

ATOM 1143 NE ARG B 24 -5.133 -15.583 5.988 1.00 0.00

ATOM 1144 HE ARG B 24 -4.449 -16.217 5.612 1.00 0.00

ATOM 1145 CZ ARG B 24 -6.447 -15.674 5.685 1.00 0.00

ATOM 1146 NH1 ARG B 24 -7.292 -14.721 6.082 1.00 0.00

ATOM 1147 HH11 ARG B 24 -6.902 -13.909 6.523 1.00 0.00

ATOM 1148 HH12 ARG B 24 -8.290 -14.737 5.931 1.00 0.00

ATOM 1149 NH2 ARG B 24 -6.880 -16.725 4.989 1.00 0.00

ATOM 1150 HH21 ARG B 24 -6.219 -17.412 4.670 1.00 0.00

ATOM 1151 HH22 ARG B 24 -7.857 -16.861 4.782 1.00 0.00

ATOM 1152 N ILE B 25 -1.381 -9.751 7.956 1.00 0.00

ATOM 1153 HN ILE B 25 -2.274 -9.315 7.849 1.00 0.00

ATOM 1154 CA ILE B 25 -0.492 -9.062 8.887 1.00 0.00

ATOM 1155 HA ILE B 25 0.494 -9.519 8.834 1.00 0.00

ATOM 1156 C ILE B 25 -1.112 -9.270 10.273 1.00 0.00

ATOM 1157 O ILE B 25 -2.311 -9.058 10.421 1.00 0.00

ATOM 1158 CB ILE B 25 -0.463 -7.562 8.508 1.00 0.00

ATOM 1159 HB ILE B 25 -1.498 -7.214 8.535 1.00 0.00

ATOM 1160 CG2 ILE B 25 0.326 -6.698 9.513 1.00 0.00

ATOM 1161 HG21 ILE B 25 0.851 -7.296 10.259 1.00 0.00

ATOM 1162 HG22 ILE B 25 1.058 -6.053 9.025 1.00 0.00

ATOM 1163 HG23 ILE B 25 -0.351 -6.031 10.048 1.00 0.00

ATOM 1164 CG1 ILE B 25 0.022 -7.337 7.061 1.00 0.00

ATOM 1165 HG11 ILE B 25 -0.508 -8.004 6.380 1.00 0.00

ATOM 1166 HG12 ILE B 25 1.081 -7.583 6.979 1.00 0.00

ATOM 1167 CD1 ILE B 25 -0.204 -5.899 6.570 1.00 0.00

ATOM 1168 HD11 ILE B 25 -1.026 -5.419 7.105 1.00 0.00

ATOM 1169 HD12 ILE B 25 0.698 -5.295 6.704 1.00 0.00

ATOM 1170 HD13 ILE B 25 -0.453 -5.876 5.504 1.00 0.00

ATOM 1171 N LEU B 26 -0.297 -9.763 11.232 1.00 0.00

ATOM 1172 HN LEU B 26 0.683 -9.723 11.064 1.00 0.00

ATOM 1173 CA LEU B 26 -0.801 -10.524 12.375 1.00 0.00

ATOM 1174 HA LEU B 26 -1.860 -10.314 12.473 1.00 0.00

ATOM 1175 C LEU B 26 -0.051 -10.102 13.654 1.00 0.00

ATOM 1176 O LEU B 26 1.096 -10.493 13.873 1.00 0.00

ATOM 1177 CB LEU B 26 -0.578 -12.040 12.153 1.00 0.00

ATOM 1178 HB1 LEU B 26 -0.689 -12.577 13.095 1.00 0.00

ATOM 1179 HB2 LEU B 26 0.460 -12.192 11.874 1.00 0.00

ATOM 1180 CG LEU B 26 -1.483 -12.711 11.097 1.00 0.00

ATOM 1181 HG LEU B 26 -1.622 -12.013 10.269 1.00 0.00

ATOM 1182 CD1 LEU B 26 -0.805 -13.969 10.500 1.00 0.00

ATOM 1183 HD11 LEU B 26 0.166 -14.175 10.954 1.00 0.00

ATOM 1184 HD12 LEU B 26 -1.408 -14.868 10.613 1.00 0.00

ATOM 1185 HD13 LEU B 26 -0.623 -13.838 9.432 1.00 0.00

ATOM 1186 CD2 LEU B 26 -2.877 -13.031 11.672 1.00 0.00

ATOM 1187 HD21 LEU B 26 -3.050 -12.547 12.637 1.00 0.00

ATOM 1188 HD22 LEU B 26 -3.653 -12.681 10.990 1.00 0.00

ATOM 1189 HD23 LEU B 26 -3.036 -14.101 11.817 1.00 0.00

ATOM 1190 N GLN B 27 -0.787 -9.327 14.470 1.00 0.00

ATOM 1191 HN GLN B 27 -1.765 -9.199 14.271 1.00 0.00

ATOM 1192 CA GLN B 27 -0.300 -8.889 15.769 1.00 0.00

ATOM 1193 HA GLN B 27 0.770 -8.687 15.696 1.00 0.00

ATOM 1194 C GLN B 27 -0.573 -10.025 16.752 1.00 0.00

ATOM 1195 O GLN B 27 -1.685 -10.539 16.791 1.00 0.00

ATOM 1196 CB GLN B 27 -1.086 -7.634 16.174 1.00 0.00

ATOM 1197 HB1 GLN B 27 -2.148 -7.867 16.291 1.00 0.00

ATOM 1198 HB2 GLN B 27 -1.041 -6.919 15.352 1.00 0.00

ATOM 1199 CG GLN B 27 -0.532 -6.972 17.446 1.00 0.00

ATOM 1200 HG1 GLN B 27 0.544 -6.822 17.350 1.00 0.00

ATOM 1201 HG2 GLN B 27 -0.684 -7.599 18.325 1.00 0.00

ATOM 1202 CD GLN B 27 -1.169 -5.592 17.642 1.00 0.00

ATOM 1203 OE1 GLN B 27 -0.952 -4.692 16.848 1.00 0.00

ATOM 1204 NE2 GLN B 27 -1.944 -5.488 18.729 1.00 0.00

ATOM 1205 HE21 GLN B 27 -2.497 -4.654 18.774 1.00 0.00

ATOM 1206 HE22 GLN B 27 -1.988 -6.137 19.492 1.00 0.00

ATOM 1207 N ARG B 28 0.465 -10.423 17.504 1.00 0.00

ATOM 1208 HN ARG B 28 1.331 -9.940 17.390 1.00 0.00

ATOM 1209 CA ARG B 28 0.304 -11.456 18.527 1.00 0.00

ATOM 1210 HA ARG B 28 -0.503 -12.123 18.228 1.00 0.00

ATOM 1211 C ARG B 28 -0.031 -10.786 19.872 1.00 0.00

ATOM 1212 O ARG B 28 0.674 -9.885 20.301 1.00 0.00

ATOM 1213 CB ARG B 28 1.620 -12.246 18.640 1.00 0.00

ATOM 1214 HB1 ARG B 28 2.428 -11.544 18.849 1.00 0.00

ATOM 1215 HB2 ARG B 28 1.851 -12.702 17.678 1.00 0.00

ATOM 1216 CG ARG B 28 1.591 -13.328 19.731 1.00 0.00

ATOM 1217 HG1 ARG B 28 0.767 -14.017 19.543 1.00 0.00

ATOM 1218 HG2 ARG B 28 1.377 -12.851 20.687 1.00 0.00

ATOM 1219 CD ARG B 28 2.919 -14.081 19.887 1.00 0.00

ATOM 1220 HD1 ARG B 28 2.986 -14.616 20.838 1.00 0.00

ATOM 1221 HD2 ARG B 28 3.760 -13.388 19.866 1.00 0.00

ATOM 1222 NE ARG B 28 3.135 -15.031 18.799 1.00 0.00

ATOM 1223 HE ARG B 28 3.768 -14.695 18.094 1.00 0.00

ATOM 1224 CZ ARG B 28 2.570 -16.264 18.824 1.00 0.00

ATOM 1225 NH1 ARG B 28 1.727 -16.602 19.796 1.00 0.00

ATOM 1226 HH11 ARG B 28 1.508 -15.922 20.500 1.00 0.00

ATOM 1227 HH12 ARG B 28 1.306 -17.510 19.852 1.00 0.00

ATOM 1228 NH2 ARG B 28 2.872 -17.143 17.870 1.00 0.00

ATOM 1229 HH21 ARG B 28 3.486 -16.891 17.116 1.00 0.00

ATOM 1230 HH22 ARG B 28 2.482 -18.071 17.923 1.00 0.00

ATOM 1231 N GLY B 29 -1.096 -11.303 20.516 1.00 0.00

ATOM 1232 HN GLY B 29 -1.548 -12.072 20.067 1.00 0.00

ATOM 1233 CA GLY B 29 -1.471 -10.913 21.868 1.00 0.00

ATOM 1234 HA1 GLY B 29 -2.277 -10.186 21.799 1.00 0.00

ATOM 1235 HA2 GLY B 29 -0.612 -10.472 22.374 1.00 0.00

ATOM 1236 C GLY B 29 -1.926 -12.160 22.619 1.00 0.00

ATOM 1237 O GLY B 29 -1.693 -13.275 22.163 1.00 0.00

ATOM 1238 N LEU B 30 -2.591 -11.924 23.766 1.00 0.00

ATOM 1239 HN LEU B 30 -2.755 -10.980 24.056 1.00 0.00

ATOM 1240 CA LEU B 30 -2.972 -13.044 24.629 1.00 0.00

ATOM 1241 HA LEU B 30 -2.052 -13.572 24.885 1.00 0.00

ATOM 1242 C LEU B 30 -3.884 -14.053 23.904 1.00 0.00

ATOM 1243 O LEU B 30 -3.797 -15.253 24.112 1.00 0.00

ATOM 1244 CB LEU B 30 -3.649 -12.522 25.912 1.00 0.00

ATOM 1245 HB1 LEU B 30 -4.103 -13.352 26.459 1.00 0.00

ATOM 1246 HB2 LEU B 30 -4.466 -11.853 25.637 1.00 0.00

ATOM 1247 CG LEU B 30 -2.679 -11.783 26.856 1.00 0.00

ATOM 1248 HG LEU B 30 -2.130 -11.054 26.257 1.00 0.00

ATOM 1249 CD1 LEU B 30 -3.429 -10.968 27.934 1.00 0.00

ATOM 1250 HD11 LEU B 30 -4.512 -10.997 27.806 1.00 0.00

ATOM 1251 HD12 LEU B 30 -3.221 -11.316 28.947 1.00 0.00

ATOM 1252 HD13 LEU B 30 -3.132 -9.918 27.898 1.00 0.00

ATOM 1253 CD2 LEU B 30 -1.639 -12.747 27.465 1.00 0.00

ATOM 1254 HD21 LEU B 30 -1.807 -13.778 27.150 1.00 0.00

ATOM 1255 HD22 LEU B 30 -0.631 -12.473 27.151 1.00 0.00

ATOM 1256 HD23 LEU B 30 -1.651 -12.746 28.556 1.00 0.00

ATOM 1257 N LEU B 31 -4.767 -13.512 23.048 1.00 0.00

ATOM 1258 HN LEU B 31 -4.723 -12.534 22.862 1.00 0.00

ATOM 1259 CA LEU B 31 -5.762 -14.354 22.385 1.00 0.00

ATOM 1260 HA LEU B 31 -6.097 -15.104 23.102 1.00 0.00

ATOM 1261 C LEU B 31 -5.190 -15.139 21.185 1.00 0.00

ATOM 1262 O LEU B 31 -5.926 -15.876 20.538 1.00 0.00

ATOM 1263 CB LEU B 31 -6.978 -13.510 21.949 1.00 0.00

ATOM 1264 HB1 LEU B 31 -7.420 -13.958 21.059 1.00 0.00

ATOM 1265 HB2 LEU B 31 -6.671 -12.509 21.644 1.00 0.00

ATOM 1266 CG LEU B 31 -8.111 -13.437 22.992 1.00 0.00

ATOM 1267 HG LEU B 31 -8.336 -14.462 23.297 1.00 0.00

ATOM 1268 CD1 LEU B 31 -7.706 -12.666 24.265 1.00 0.00

ATOM 1269 HD11 LEU B 31 -6.705 -12.238 24.192 1.00 0.00

ATOM 1270 HD12 LEU B 31 -8.387 -11.840 24.480 1.00 0.00

ATOM 1271 HD13 LEU B 31 -7.724 -13.327 25.134 1.00 0.00

ATOM 1272 CD2 LEU B 31 -9.413 -12.896 22.358 1.00 0.00

ATOM 1273 HD21 LEU B 31 -9.319 -12.737 21.280 1.00 0.00

ATOM 1274 HD22 LEU B 31 -10.234 -13.602 22.502 1.00 0.00

ATOM 1275 HD23 LEU B 31 -9.734 -11.949 22.793 1.00 0.00

ATOM 1276 N GLY B 32 -3.888 -14.970 20.904 1.00 0.00

ATOM 1277 HN GLY B 32 -3.293 -14.341 21.411 1.00 0.00

ATOM 1278 CA GLY B 32 -3.290 -15.623 19.747 1.00 0.00

ATOM 1279 HA1 GLY B 32 -3.975 -16.343 19.295 1.00 0.00

ATOM 1280 HA2 GLY B 32 -2.404 -16.145 20.110 1.00 0.00

ATOM 1281 C GLY B 32 -2.872 -14.532 18.765 1.00 0.00

ATOM 1282 O GLY B 32 -2.416 -13.478 19.188 1.00 0.00

ATOM 1283 N ARG B 33 -3.017 -14.826 17.459 1.00 0.00

ATOM 1284 HN ARG B 33 -3.578 -15.593 17.158 1.00 0.00

ATOM 1285 CA ARG B 33 -2.614 -13.884 16.420 1.00 0.00

ATOM 1286 HA ARG B 33 -2.101 -13.038 16.879 1.00 0.00

ATOM 1287 C ARG B 33 -3.863 -13.354 15.692 1.00 0.00

ATOM 1288 O ARG B 33 -4.592 -14.076 15.014 1.00 0.00

ATOM 1289 CB ARG B 33 -1.652 -14.563 15.436 1.00 0.00

ATOM 1290 HB1 ARG B 33 -1.542 -13.907 14.573 1.00 0.00

ATOM 1291 HB2 ARG B 33 -2.090 -15.491 15.065 1.00 0.00

ATOM 1292 CG ARG B 33 -0.257 -14.803 16.028 1.00 0.00

ATOM 1293 HG1 ARG B 33 -0.287 -15.627 16.744 1.00 0.00

ATOM 1294 HG2 ARG B 33 0.045 -13.909 16.578 1.00 0.00

ATOM 1295 CD ARG B 33 0.794 -15.036 14.939 1.00 0.00

ATOM 1296 HD1 ARG B 33 1.786 -15.239 15.352 1.00 0.00

ATOM 1297 HD2 ARG B 33 0.852 -14.144 14.305 1.00 0.00

ATOM 1298 NE ARG B 33 0.414 -16.160 14.086 1.00 0.00

ATOM 1299 HE ARG B 33 -0.411 -16.692 14.305 1.00 0.00

ATOM 1300 CZ ARG B 33 1.063 -16.358 12.911 1.00 0.00

ATOM 1301 NH1 ARG B 33 2.149 -15.646 12.596 1.00 0.00

ATOM 1302 HH11 ARG B 33 2.480 -14.864 13.143 1.00 0.00

ATOM 1303 HH12 ARG B 33 2.648 -15.918 11.760 1.00 0.00

ATOM 1304 NH2 ARG B 33 0.590 -17.280 12.072 1.00 0.00

ATOM 1305 HH21 ARG B 33 -0.228 -17.848 12.189 1.00 0.00

ATOM 1306 HH22 ARG B 33 1.077 -17.438 11.196 1.00 0.00

ATOM 1307 N SER B 34 -4.027 -12.037 15.870 1.00 0.00

ATOM 1308 HN SER B 34 -3.329 -11.555 16.410 1.00 0.00

ATOM 1309 CA SER B 34 -5.126 -11.287 15.272 1.00 0.00

ATOM 1310 HA SER B 34 -5.924 -11.990 15.023 1.00 0.00

ATOM 1311 C SER B 34 -4.604 -10.599 13.995 1.00 0.00

ATOM 1312 O SER B 34 -3.459 -10.163 13.970 1.00 0.00

ATOM 1313 CB SER B 34 -5.592 -10.271 16.333 1.00 0.00

ATOM 1314 HB1 SER B 34 -4.988 -9.348 16.286 1.00 0.00

ATOM 1315 HB2 SER B 34 -5.393 -10.695 17.323 1.00 0.00

ATOM 1316 OG SER B 34 -7.002 -10.057 16.292 1.00 0.00

ATOM 1317 HG SER B 34 -7.236 -9.140 16.066 1.00 0.00

ATOM 1318 N GLN B 35 -5.479 -10.556 12.965 1.00 0.00

ATOM 1319 HN GLN B 35 -6.414 -10.877 13.119 1.00 0.00

ATOM 1320 CA GLN B 35 -5.142 -9.961 11.671 1.00 0.00

ATOM 1321 HA GLN B 35 -4.076 -10.094 11.518 1.00 0.00

ATOM 1322 C GLN B 35 -5.515 -8.457 11.709 1.00 0.00

ATOM 1323 O GLN B 35 -6.681 -8.114 11.885 1.00 0.00

ATOM 1324 CB GLN B 35 -5.931 -10.711 10.578 1.00 0.00

ATOM 1325 HB1 GLN B 35 -6.989 -10.583 10.797 1.00 0.00

ATOM 1326 HB2 GLN B 35 -5.725 -11.780 10.637 1.00 0.00

ATOM 1327 CG GLN B 35 -5.644 -10.237 9.145 1.00 0.00

ATOM 1328 HG1 GLN B 35 -4.656 -10.550 8.816 1.00 0.00

ATOM 1329 HG2 GLN B 35 -5.665 -9.151 9.111 1.00 0.00

ATOM 1330 CD GLN B 35 -6.720 -10.741 8.173 1.00 0.00

ATOM 1331 OE1 GLN B 35 -7.899 -10.426 8.312 1.00 0.00

ATOM 1332 NE2 GLN B 35 -6.282 -11.543 7.186 1.00 0.00

ATOM 1333 HE21 GLN B 35 -6.958 -11.719 6.472 1.00 0.00

ATOM 1334 HE22 GLN B 35 -5.318 -11.830 7.164 1.00 0.00

ATOM 1335 N VAL B 36 -4.518 -7.573 11.559 1.00 0.00

ATOM 1336 HN VAL B 36 -3.595 -7.941 11.458 1.00 0.00

ATOM 1337 CA VAL B 36 -4.769 -6.130 11.623 1.00 0.00

ATOM 1338 HA VAL B 36 -5.761 -5.962 12.043 1.00 0.00

ATOM 1339 C VAL B 36 -4.783 -5.506 10.204 1.00 0.00

ATOM 1340 O VAL B 36 -4.985 -4.309 10.023 1.00 0.00

ATOM 1341 CB VAL B 36 -3.728 -5.493 12.570 1.00 0.00

ATOM 1342 HB VAL B 36 -3.847 -4.406 12.505 1.00 0.00

ATOM 1343 CG1 VAL B 36 -3.973 -5.875 14.055 1.00 0.00

ATOM 1344 HG11 VAL B 36 -4.882 -6.465 14.203 1.00 0.00

ATOM 1345 HG12 VAL B 36 -3.153 -6.446 14.494 1.00 0.00

ATOM 1346 HG13 VAL B 36 -4.093 -4.976 14.664 1.00 0.00

ATOM 1347 CG2 VAL B 36 -2.285 -5.797 12.126 1.00 0.00

ATOM 1348 HG21 VAL B 36 -2.244 -6.358 11.193 1.00 0.00

ATOM 1349 HG22 VAL B 36 -1.752 -4.861 11.945 1.00 0.00

ATOM 1350 HG23 VAL B 36 -1.722 -6.357 12.872 1.00 0.00

ATOM 1351 N GLY B 37 -4.549 -6.398 9.212 1.00 0.00

ATOM 1352 HN GLY B 37 -4.415 -7.383 9.371 1.00 0.00

ATOM 1353 CA GLY B 37 -4.695 -5.990 7.819 1.00 0.00

ATOM 1354 HA1 GLY B 37 -4.198 -5.037 7.655 1.00 0.00

ATOM 1355 HA2 GLY B 37 -5.756 -5.849 7.619 1.00 0.00

ATOM 1356 C GLY B 37 -4.122 -7.080 6.914 1.00 0.00

ATOM 1357 O GLY B 37 -3.775 -8.170 7.380 1.00 0.00

ATOM 1358 N VAL B 38 -4.034 -6.702 5.630 1.00 0.00

ATOM 1359 HN VAL B 38 -4.279 -5.769 5.357 1.00 0.00

ATOM 1360 CA VAL B 38 -3.555 -7.567 4.564 1.00 0.00

ATOM 1361 HA VAL B 38 -2.994 -8.381 5.020 1.00 0.00

ATOM 1362 C VAL B 38 -2.628 -6.696 3.702 1.00 0.00

ATOM 1363 O VAL B 38 -2.788 -5.480 3.661 1.00 0.00

ATOM 1364 CB VAL B 38 -4.766 -8.079 3.737 1.00 0.00

ATOM 1365 HB VAL B 38 -5.282 -7.191 3.370 1.00 0.00

ATOM 1366 CG1 VAL B 38 -4.362 -8.881 2.479 1.00 0.00

ATOM 1367 HG11 VAL B 38 -3.320 -9.196 2.503 1.00 0.00

ATOM 1368 HG12 VAL B 38 -4.929 -9.798 2.355 1.00 0.00

ATOM 1369 HG13 VAL B 38 -4.504 -8.287 1.574 1.00 0.00

ATOM 1370 CG2 VAL B 38 -5.803 -8.845 4.588 1.00 0.00

ATOM 1371 HG21 VAL B 38 -5.558 -8.840 5.646 1.00 0.00

ATOM 1372 HG22 VAL B 38 -6.791 -8.400 4.486 1.00 0.00

ATOM 1373 HG23 VAL B 38 -5.878 -9.894 4.318 1.00 0.00

ATOM 1374 N GLY B 39 -1.699 -7.364 3.000 1.00 0.00

ATOM 1375 HN GLY B 39 -1.561 -8.346 3.140 1.00 0.00

ATOM 1376 CA GLY B 39 -0.845 -6.610 2.093 1.00 0.00

ATOM 1377 HA1 GLY B 39 -0.113 -6.104 2.720 1.00 0.00

ATOM 1378 HA2 GLY B 39 -1.445 -5.859 1.573 1.00 0.00

ATOM 1379 C GLY B 39 -0.157 -7.552 1.098 1.00 0.00

ATOM 1380 O GLY B 39 -0.049 -8.756 1.340 1.00 0.00

ATOM 1381 N VAL B 40 0.322 -6.912 0.006 1.00 0.00

ATOM 1382 HN VAL B 40 0.348 -5.919 -0.144 1.00 0.00

ATOM 1383 CA VAL B 40 0.857 -7.674 -1.136 1.00 0.00

ATOM 1384 HA VAL B 40 0.676 -8.735 -0.968 1.00 0.00

ATOM 1385 C VAL B 40 2.372 -7.430 -1.348 1.00 0.00

ATOM 1386 O VAL B 40 2.799 -6.280 -1.470 1.00 0.00

ATOM 1387 CB VAL B 40 0.130 -7.220 -2.421 1.00 0.00

ATOM 1388 HB VAL B 40 0.439 -6.196 -2.628 1.00 0.00

ATOM 1389 CG1 VAL B 40 0.561 -8.039 -3.656 1.00 0.00

ATOM 1390 HG11 VAL B 40 1.242 -8.849 -3.398 1.00 0.00

ATOM 1391 HG12 VAL B 40 -0.283 -8.518 -4.143 1.00 0.00

ATOM 1392 HG13 VAL B 40 1.066 -7.424 -4.401 1.00 0.00

ATOM 1393 CG2 VAL B 40 -1.402 -7.151 -2.255 1.00 0.00

ATOM 1394 HG21 VAL B 40 -1.748 -7.577 -1.315 1.00 0.00

ATOM 1395 HG22 VAL B 40 -1.728 -6.110 -2.294 1.00 0.00

ATOM 1396 HG23 VAL B 40 -1.912 -7.670 -3.062 1.00 0.00

ATOM 1397 N PHE B 41 3.086 -8.585 -1.441 1.00 0.00

ATOM 1398 HN PHE B 41 2.610 -9.454 -1.345 1.00 0.00

ATOM 1399 CA PHE B 41 4.413 -8.670 -2.027 1.00 0.00

ATOM 1400 HA PHE B 41 4.963 -7.790 -1.713 1.00 0.00

ATOM 1401 C PHE B 41 4.255 -8.655 -3.555 1.00 0.00

ATOM 1402 O PHE B 41 3.490 -9.405 -4.158 1.00 0.00

ATOM 1403 CB PHE B 41 5.165 -9.944 -1.591 1.00 0.00

ATOM 1404 HB1 PHE B 41 5.791 -10.296 -2.413 1.00 0.00

ATOM 1405 HB2 PHE B 41 4.468 -10.752 -1.371 1.00 0.00

ATOM 1406 CG PHE B 41 6.094 -9.687 -0.436 1.00 0.00

ATOM 1407 CD1 PHE B 41 5.868 -10.246 0.810 1.00 0.00

ATOM 1408 HD1 PHE B 41 5.011 -10.878 0.958 1.00 0.00

ATOM 1409 CE1 PHE B 41 6.724 -9.992 1.870 1.00 0.00

ATOM 1410 HE1 PHE B 41 6.516 -10.425 2.835 1.00 0.00

ATOM 1411 CZ PHE B 41 7.820 -9.166 1.688 1.00 0.00

ATOM 1412 HZ PHE B 41 8.487 -8.946 2.503 1.00 0.00

ATOM 1413 CE2 PHE B 41 8.046 -8.590 0.453 1.00 0.00

ATOM 1414 HE2 PHE B 41 8.852 -7.885 0.343 1.00 0.00

ATOM 1415 CD2 PHE B 41 7.196 -8.864 -0.607 1.00 0.00

ATOM 1416 HD2 PHE B 41 7.363 -8.418 -1.575 1.00 0.00

ATOM 1417 N GLN B 42 5.031 -7.723 -4.126 1.00 0.00

ATOM 1418 HN GLN B 42 5.512 -7.094 -3.519 1.00 0.00

ATOM 1419 CA GLN B 42 5.281 -7.592 -5.546 1.00 0.00

ATOM 1420 HA GLN B 42 5.127 -8.572 -5.992 1.00 0.00

ATOM 1421 C GLN B 42 6.747 -7.111 -5.774 1.00 0.00

ATOM 1422 O GLN B 42 7.223 -6.118 -5.226 1.00 0.00

ATOM 1423 CB GLN B 42 4.245 -6.690 -6.202 1.00 0.00

ATOM 1424 HB1 GLN B 42 4.336 -5.661 -5.857 1.00 0.00

ATOM 1425 HB2 GLN B 42 3.248 -7.043 -5.927 1.00 0.00

ATOM 1426 CG GLN B 42 4.391 -6.758 -7.720 1.00 0.00

ATOM 1427 HG1 GLN B 42 4.246 -7.777 -8.085 1.00 0.00

ATOM 1428 HG2 GLN B 42 5.376 -6.416 -8.038 1.00 0.00

ATOM 1429 CD GLN B 42 3.352 -5.842 -8.310 1.00 0.00

ATOM 1430 OE1 GLN B 42 3.505 -4.631 -8.326 1.00 0.00

ATOM 1431 NE2 GLN B 42 2.231 -6.436 -8.707 1.00 0.00

ATOM 1432 HE21 GLN B 42 1.525 -5.832 -9.069 1.00 0.00

ATOM 1433 HE22 GLN B 42 2.125 -7.436 -8.618 1.00 0.00

ATOM 1434 N ASP B 43 7.399 -7.911 -6.642 1.00 0.00

ATOM 1435 HN ASP B 43 6.968 -8.809 -6.763 1.00 0.00

ATOM 1436 CA ASP B 43 8.805 -7.780 -7.045 1.00 0.00

ATOM 1437 HA ASP B 43 9.012 -8.769 -7.451 1.00 0.00

ATOM 1438 C ASP B 43 9.720 -7.577 -5.801 1.00 0.00

ATOM 1439 O ASP B 43 10.741 -6.887 -5.846 1.00 0.00

ATOM 1440 CB ASP B 43 9.121 -6.714 -8.139 1.00 0.00

ATOM 1441 HB1 ASP B 43 10.124 -6.895 -8.528 1.00 0.00

ATOM 1442 HB2 ASP B 43 9.169 -5.747 -7.645 1.00 0.00

ATOM 1443 CG ASP B 43 8.188 -6.575 -9.365 1.00 0.00

ATOM 1444 OD1 ASP B 43 7.958 -5.432 -9.791 1.00 0.00

ATOM 1445 OD2 ASP B 43 7.702 -7.581 -9.895 1.00 0.00

ATOM 1446 N GLY B 44 9.272 -8.200 -4.699 1.00 0.00

ATOM 1447 HN GLY B 44 8.427 -8.748 -4.733 1.00 0.00

ATOM 1448 CA GLY B 44 10.097 -8.191 -3.502 1.00 0.00

ATOM 1449 HA1 GLY B 44 11.149 -8.024 -3.745 1.00 0.00

ATOM 1450 HA2 GLY B 44 9.999 -9.173 -3.057 1.00 0.00

ATOM 1451 C GLY B 44 9.667 -7.171 -2.468 1.00 0.00

ATOM 1452 O GLY B 44 10.143 -7.234 -1.342 1.00 0.00

ATOM 1453 N VAL B 45 8.768 -6.251 -2.858 1.00 0.00

ATOM 1454 HN VAL B 45 8.179 -6.460 -3.634 1.00 0.00

ATOM 1455 CA VAL B 45 8.353 -5.159 -1.989 1.00 0.00

ATOM 1456 HA VAL B 45 9.035 -5.103 -1.140 1.00 0.00

ATOM 1457 C VAL B 45 6.941 -5.504 -1.510 1.00 0.00

ATOM 1458 O VAL B 45 6.059 -5.826 -2.309 1.00 0.00

ATOM 1459 CB VAL B 45 8.395 -3.819 -2.758 1.00 0.00

ATOM 1460 HB VAL B 45 7.627 -3.861 -3.525 1.00 0.00

ATOM 1461 CG1 VAL B 45 8.057 -2.595 -1.875 1.00 0.00

ATOM 1462 HG11 VAL B 45 7.469 -2.872 -0.993 1.00 0.00

ATOM 1463 HG12 VAL B 45 8.967 -2.113 -1.507 1.00 0.00

ATOM 1464 HG13 VAL B 45 7.485 -1.854 -2.447 1.00 0.00

ATOM 1465 CG2 VAL B 45 9.736 -3.633 -3.495 1.00 0.00

ATOM 1466 HG21 VAL B 45 10.490 -4.361 -3.183 1.00 0.00

ATOM 1467 HG22 VAL B 45 9.600 -3.754 -4.563 1.00 0.00

ATOM 1468 HG23 VAL B 45 10.152 -2.639 -3.341 1.00 0.00

ATOM 1469 N PHE B 46 6.826 -5.427 -0.171 1.00 0.00

ATOM 1470 HN PHE B 46 7.610 -5.032 0.315 1.00 0.00

ATOM 1471 CA PHE B 46 5.528 -5.544 0.453 1.00 0.00

ATOM 1472 HA PHE B 46 4.928 -6.233 -0.144 1.00 0.00

ATOM 1473 C PHE B 46 4.862 -4.160 0.425 1.00 0.00

ATOM 1474 O PHE B 46 5.482 -3.100 0.530 1.00 0.00

ATOM 1475 CB PHE B 46 5.707 -6.101 1.871 1.00 0.00

ATOM 1476 HB1 PHE B 46 6.202 -5.377 2.501 1.00 0.00

ATOM 1477 HB2 PHE B 46 6.359 -6.968 1.828 1.00 0.00

ATOM 1478 CG PHE B 46 4.429 -6.551 2.513 1.00 0.00

ATOM 1479 CD1 PHE B 46 3.750 -7.629 1.970 1.00 0.00

ATOM 1480 HD1 PHE B 46 4.106 -8.122 1.073 1.00 0.00

ATOM 1481 CE1 PHE B 46 2.601 -8.101 2.568 1.00 0.00

ATOM 1482 HE1 PHE B 46 2.125 -8.940 2.091 1.00 0.00

ATOM 1483 CZ PHE B 46 2.099 -7.506 3.716 1.00 0.00

ATOM 1484 HZ PHE B 46 1.191 -7.878 4.168 1.00 0.00

ATOM 1485 CE2 PHE B 46 2.766 -6.416 4.260 1.00 0.00

ATOM 1486 HE2 PHE B 46 2.388 -5.942 5.155 1.00 0.00

ATOM 1487 CD2 PHE B 46 3.925 -5.933 3.656 1.00 0.00

ATOM 1488 HD2 PHE B 46 4.434 -5.076 4.083 1.00 0.00

ATOM 1489 N HSD B 47 3.532 -4.256 0.257 1.00 0.00

ATOM 1490 HN HSD B 47 3.154 -5.133 -0.052 1.00 0.00

ATOM 1491 CA HSD B 47 2.640 -3.100 0.240 1.00 0.00

ATOM 1492 HA HSD B 47 3.176 -2.212 0.576 1.00 0.00

ATOM 1493 C HSD B 47 1.475 -3.385 1.211 1.00 0.00

ATOM 1494 O HSD B 47 0.985 -4.521 1.291 1.00 0.00

ATOM 1495 CB HSD B 47 2.154 -2.898 -1.214 1.00 0.00

ATOM 1496 HB1 HSD B 47 1.615 -1.959 -1.341 1.00 0.00

ATOM 1497 HB2 HSD B 47 1.484 -3.707 -1.508 1.00 0.00

ATOM 1498 CG HSD B 47 3.326 -2.913 -2.181 1.00 0.00

ATOM 1499 ND1 HSD B 47 3.691 -4.013 -2.870 1.00 0.00

ATOM 1500 CE1 HSD B 47 4.759 -3.651 -3.610 1.00 0.00

ATOM 1501 HE1 HSD B 47 5.293 -4.346 -4.247 1.00 0.00

ATOM 1502 NE2 HSD B 47 5.106 -2.355 -3.431 1.00 0.00

ATOM 1503 HE2 HSD B 47 5.862 -1.853 -3.830 1.00 0.00

ATOM 1504 CD2 HSD B 47 4.211 -1.869 -2.529 1.00 0.00

ATOM 1505 HD2 HSD B 47 4.209 -0.855 -2.152 1.00 0.00

ATOM 1506 N THR B 48 1.038 -2.318 1.922 1.00 0.00

ATOM 1507 HN THR B 48 1.573 -1.473 1.831 1.00 0.00

ATOM 1508 CA THR B 48 -0.137 -2.322 2.809 1.00 0.00

ATOM 1509 HA THR B 48 -0.962 -2.746 2.240 1.00 0.00

ATOM 1510 C THR B 48 -0.490 -0.853 3.143 1.00 0.00

ATOM 1511 O THR B 48 0.252 0.084 2.847 1.00 0.00

ATOM 1512 CB THR B 48 0.113 -3.160 4.100 1.00 0.00

ATOM 1513 HB THR B 48 0.297 -4.196 3.800 1.00 0.00

ATOM 1514 OG1 THR B 48 -0.987 -3.203 5.012 1.00 0.00

ATOM 1515 HG1 THR B 48 -1.222 -2.306 5.250 1.00 0.00

ATOM 1516 CG2 THR B 48 1.359 -2.686 4.861 1.00 0.00

ATOM 1517 HG21 THR B 48 1.796 -1.779 4.440 1.00 0.00

ATOM 1518 HG22 THR B 48 1.097 -2.486 5.896 1.00 0.00

ATOM 1519 HG23 THR B 48 2.117 -3.472 4.876 1.00 0.00

ATOM 1520 N MET B 49 -1.650 -0.703 3.815 1.00 0.00

ATOM 1521 HN MET B 49 -2.218 -1.501 4.036 1.00 0.00

ATOM 1522 CA MET B 49 -2.081 0.612 4.261 1.00 0.00

ATOM 1523 HA MET B 49 -2.020 1.281 3.403 1.00 0.00

ATOM 1524 C MET B 49 -1.159 1.147 5.360 1.00 0.00

ATOM 1525 O MET B 49 -0.493 0.390 6.068 1.00 0.00

ATOM 1526 CB MET B 49 -3.534 0.544 4.741 1.00 0.00

ATOM 1527 HB1 MET B 49 -3.907 1.561 4.882 1.00 0.00

ATOM 1528 HB2 MET B 49 -3.596 0.047 5.710 1.00 0.00

ATOM 1529 CG MET B 49 -4.445 -0.181 3.743 1.00 0.00

ATOM 1530 HG1 MET B 49 -5.486 -0.153 4.068 1.00 0.00

ATOM 1531 HG2 MET B 49 -4.190 -1.240 3.693 1.00 0.00

ATOM 1532 SD MET B 49 -4.303 0.524 2.087 1.00 0.00

ATOM 1533 CE MET B 49 -5.619 1.750 2.163 1.00 0.00

ATOM 1534 HE1 MET B 49 -6.023 1.826 3.174 1.00 0.00

ATOM 1535 HE2 MET B 49 -5.220 2.726 1.876 1.00 0.00

ATOM 1536 HE3 MET B 49 -6.405 1.487 1.455 1.00 0.00

ATOM 1537 N TRP B 50 -1.201 2.493 5.387 1.00 0.00

ATOM 1538 HN TRP B 50 -1.896 2.908 4.795 1.00 0.00

ATOM 1539 CA TRP B 50 -0.490 3.219 6.423 1.00 0.00

ATOM 1540 HA TRP B 50 0.527 2.833 6.471 1.00 0.00

ATOM 1541 C TRP B 50 -1.162 2.914 7.767 1.00 0.00

ATOM 1542 O TRP B 50 -0.479 2.592 8.735 1.00 0.00

ATOM 1543 CB TRP B 50 -0.445 4.709 6.059 1.00 0.00

ATOM 1544 HB1 TRP B 50 -1.447 5.045 5.796 1.00 0.00

ATOM 1545 HB2 TRP B 50 0.203 4.864 5.196 1.00 0.00

ATOM 1546 CG TRP B 50 0.036 5.582 7.197 1.00 0.00

ATOM 1547 CD1 TRP B 50 -0.781 6.477 7.889 1.00 0.00

ATOM 1548 HD1 TRP B 50 -1.838 6.623 7.701 1.00 0.00

ATOM 1549 NE1 TRP B 50 -0.035 7.124 8.809 1.00 0.00

ATOM 1550 HE1 TRP B 50 -0.370 7.788 9.437 1.00 0.00

ATOM 1551 CE2 TRP B 50 1.280 6.692 8.772 1.00 0.00

ATOM 1552 CD2 TRP B 50 1.368 5.719 7.749 1.00 0.00

ATOM 1553 CE3 TRP B 50 2.573 5.109 7.505 1.00 0.00

ATOM 1554 HE3 TRP B 50 2.666 4.340 6.754 1.00 0.00

ATOM 1555 CZ3 TRP B 50 3.709 5.474 8.231 1.00 0.00

ATOM 1556 HZ3 TRP B 50 4.656 4.979 8.057 1.00 0.00

ATOM 1557 CH2 TRP B 50 3.613 6.444 9.227 1.00 0.00

ATOM 1558 HH2 TRP B 50 4.480 6.727 9.806 1.00 0.00

ATOM 1559 CZ2 TRP B 50 2.390 7.059 9.482 1.00 0.00

ATOM 1560 HZ2 TRP B 50 2.316 7.813 10.253 1.00 0.00

ATOM 1561 N HSD B 51 -2.508 2.984 7.780 1.00 0.00

ATOM 1562 HN HSD B 51 -2.958 3.202 6.920 1.00 0.00

ATOM 1563 CA HSD B 51 -3.191 2.875 9.064 1.00 0.00

ATOM 1564 HA HSD B 51 -2.713 3.624 9.695 1.00 0.00

ATOM 1565 C HSD B 51 -2.948 1.529 9.778 1.00 0.00

ATOM 1566 O HSD B 51 -3.064 1.460 10.996 1.00 0.00

ATOM 1567 CB HSD B 51 -4.682 3.268 8.973 1.00 0.00

ATOM 1568 HB1 HSD B 51 -4.766 4.220 8.452 1.00 0.00

ATOM 1569 HB2 HSD B 51 -5.107 3.385 9.972 1.00 0.00

ATOM 1570 CG HSD B 51 -5.527 2.258 8.233 1.00 0.00

ATOM 1571 ND1 HSD B 51 -5.871 2.381 6.932 1.00 0.00

ATOM 1572 CE1 HSD B 51 -6.624 1.294 6.651 1.00 0.00

ATOM 1573 HE1 HSD B 51 -7.014 1.065 5.668 1.00 0.00

ATOM 1574 NE2 HSD B 51 -6.790 0.492 7.724 1.00 0.00

ATOM 1575 HE2 HSD B 51 -7.265 -0.382 7.765 1.00 0.00

ATOM 1576 CD2 HSD B 51 -6.108 1.077 8.741 1.00 0.00

ATOM 1577 HD2 HSD B 51 -6.050 0.698 9.758 1.00 0.00

ATOM 1578 N VAL B 52 -2.592 0.492 9.007 1.00 0.00

ATOM 1579 HN VAL B 52 -2.555 0.630 8.020 1.00 0.00

ATOM 1580 CA VAL B 52 -2.443 -0.856 9.560 1.00 0.00

ATOM 1581 HA VAL B 52 -3.294 -1.041 10.222 1.00 0.00

ATOM 1582 C VAL B 52 -1.138 -1.015 10.399 1.00 0.00

ATOM 1583 O VAL B 52 -1.133 -1.653 11.440 1.00 0.00

ATOM 1584 CB VAL B 52 -2.515 -1.839 8.361 1.00 0.00

ATOM 1585 HB VAL B 52 -1.925 -1.383 7.565 1.00 0.00

ATOM 1586 CG1 VAL B 52 -1.873 -3.227 8.611 1.00 0.00

ATOM 1587 HG11 VAL B 52 -1.661 -3.429 9.665 1.00 0.00

ATOM 1588 HG12 VAL B 52 -2.497 -4.044 8.260 1.00 0.00

ATOM 1589 HG13 VAL B 52 -0.950 -3.332 8.037 1.00 0.00

ATOM 1590 CG2 VAL B 52 -3.954 -1.964 7.792 1.00 0.00

ATOM 1591 HG21 VAL B 52 -4.602 -1.129 8.078 1.00 0.00

ATOM 1592 HG22 VAL B 52 -3.934 -2.028 6.697 1.00 0.00

ATOM 1593 HG23 VAL B 52 -4.457 -2.857 8.174 1.00 0.00

ATOM 1594 N THR B 53 -0.019 -0.490 9.840 1.00 0.00

ATOM 1595 HN THR B 53 -0.042 0.083 9.014 1.00 0.00

ATOM 1596 CA THR B 53 1.267 -0.729 10.520 1.00 0.00

ATOM 1597 HA THR B 53 1.132 -1.492 11.289 1.00 0.00

ATOM 1598 C THR B 53 1.765 0.518 11.252 1.00 0.00

ATOM 1599 O THR B 53 2.681 0.482 12.050 1.00 0.00

ATOM 1600 CB THR B 53 2.352 -1.257 9.535 1.00 0.00

ATOM 1601 HB THR B 53 3.215 -1.449 10.178 1.00 0.00

ATOM 1602 OG1 THR B 53 2.784 -0.379 8.497 1.00 0.00

ATOM 1603 HG1 THR B 53 3.359 -0.871 7.868 1.00 0.00

ATOM 1604 CG2 THR B 53 1.906 -2.560 8.846 1.00 0.00

ATOM 1605 HG21 THR B 53 0.872 -2.807 9.036 1.00 0.00

ATOM 1606 HG22 THR B 53 2.010 -2.551 7.762 1.00 0.00

ATOM 1607 HG23 THR B 53 2.516 -3.388 9.197 1.00 0.00

ATOM 1608 N ARG B 54 1.210 1.664 10.797 1.00 0.00

ATOM 1609 HN ARG B 54 0.355 1.630 10.275 1.00 0.00

ATOM 1610 CA ARG B 54 1.899 2.965 10.829 1.00 0.00

ATOM 1611 HA ARG B 54 1.731 3.387 9.841 1.00 0.00

ATOM 1612 C ARG B 54 3.443 2.834 10.993 1.00 0.00

ATOM 1613 O ARG B 54 4.016 3.318 11.956 1.00 0.00

ATOM 1614 CB ARG B 54 1.255 3.922 11.855 1.00 0.00

ATOM 1615 HB1 ARG B 54 1.883 4.809 11.955 1.00 0.00

ATOM 1616 HB2 ARG B 54 1.218 3.423 12.825 1.00 0.00

ATOM 1617 CG ARG B 54 -0.159 4.386 11.452 1.00 0.00

ATOM 1618 HG1 ARG B 54 -0.788 3.498 11.391 1.00 0.00

ATOM 1619 HG2 ARG B 54 -0.117 4.825 10.453 1.00 0.00

ATOM 1620 CD ARG B 54 -0.812 5.389 12.427 1.00 0.00

ATOM 1621 HD1 ARG B 54 -0.679 5.050 13.456 1.00 0.00

ATOM 1622 HD2 ARG B 54 -1.882 5.477 12.233 1.00 0.00

ATOM 1623 NE ARG B 54 -0.174 6.707 12.338 1.00 0.00

ATOM 1624 HE ARG B 54 0.826 6.631 12.423 1.00 0.00

ATOM 1625 CZ ARG B 54 -0.810 7.896 12.142 1.00 0.00

ATOM 1626 NH1 ARG B 54 -2.133 7.991 12.005 1.00 0.00

ATOM 1627 HH11 ARG B 54 -2.730 7.193 12.032 1.00 0.00

ATOM 1628 HH12 ARG B 54 -2.547 8.913 11.860 1.00 0.00

ATOM 1629 NH2 ARG B 54 -0.104 9.009 12.097 1.00 0.00

ATOM 1630 HH21 ARG B 54 0.876 9.052 12.305 1.00 0.00

ATOM 1631 HH22 ARG B 54 -0.599 9.849 11.856 1.00 0.00

ATOM 1632 N GLY B 55 4.039 2.113 10.030 1.00 0.00

ATOM 1633 HN GLY B 55 3.541 1.614 9.317 1.00 0.00

ATOM 1634 CA GLY B 55 5.491 1.973 10.003 1.00 0.00

ATOM 1635 HA1 GLY B 55 5.943 2.966 10.041 1.00 0.00

ATOM 1636 HA2 GLY B 55 5.727 1.515 9.045 1.00 0.00

ATOM 1637 C GLY B 55 6.102 1.097 11.117 1.00 0.00

ATOM 1638 O GLY B 55 7.270 1.272 11.450 1.00 0.00

ATOM 1639 N ALA B 56 5.330 0.142 11.667 1.00 0.00

ATOM 1640 HN ALA B 56 4.421 0.064 11.256 1.00 0.00

ATOM 1641 CA ALA B 56 5.904 -0.837 12.601 1.00 0.00

ATOM 1642 HA ALA B 56 6.592 -0.304 13.255 1.00 0.00

ATOM 1643 C ALA B 56 6.695 -1.867 11.800 1.00 0.00

ATOM 1644 O ALA B 56 6.345 -2.137 10.652 1.00 0.00

ATOM 1645 CB ALA B 56 4.821 -1.557 13.423 1.00 0.00

ATOM 1646 HB1 ALA B 56 3.838 -1.107 13.297 1.00 0.00

ATOM 1647 HB2 ALA B 56 4.725 -2.611 13.154 1.00 0.00

ATOM 1648 HB3 ALA B 56 5.073 -1.522 14.481 1.00 0.00

ATOM 1649 N VAL B 57 7.767 -2.391 12.407 1.00 0.00

ATOM 1650 HN VAL B 57 7.995 -2.165 13.351 1.00 0.00

ATOM 1651 CA VAL B 57 8.493 -3.437 11.717 1.00 0.00

ATOM 1652 HA VAL B 57 8.583 -3.111 10.676 1.00 0.00

ATOM 1653 C VAL B 57 7.664 -4.761 11.752 1.00 0.00

ATOM 1654 O VAL B 57 6.930 -5.105 12.688 1.00 0.00

ATOM 1655 CB VAL B 57 9.933 -3.517 12.261 1.00 0.00

ATOM 1656 HB VAL B 57 10.444 -4.302 11.699 1.00 0.00

ATOM 1657 CG1 VAL B 57 10.701 -2.203 11.978 1.00 0.00

ATOM 1658 HG11 VAL B 57 10.160 -1.540 11.298 1.00 0.00

ATOM 1659 HG12 VAL B 57 10.912 -1.628 12.879 1.00 0.00

ATOM 1660 HG13 VAL B 57 11.650 -2.402 11.483 1.00 0.00

ATOM 1661 CG2 VAL B 57 9.969 -3.887 13.752 1.00 0.00

ATOM 1662 HG21 VAL B 57 8.964 -4.057 14.134 1.00 0.00

ATOM 1663 HG22 VAL B 57 10.550 -4.793 13.915 1.00 0.00

ATOM 1664 HG23 VAL B 57 10.395 -3.101 14.374 1.00 0.00

ATOM 1665 N LEU B 58 7.848 -5.393 10.569 1.00 0.00

ATOM 1666 HN LEU B 58 8.466 -4.955 9.907 1.00 0.00

ATOM 1667 CA LEU B 58 7.151 -6.612 10.171 1.00 0.00

ATOM 1668 HA LEU B 58 6.377 -6.814 10.909 1.00 0.00

ATOM 1669 C LEU B 58 8.139 -7.790 10.124 1.00 0.00

ATOM 1670 O LEU B 58 9.176 -7.725 9.461 1.00 0.00

ATOM 1671 CB LEU B 58 6.549 -6.449 8.753 1.00 0.00

ATOM 1672 HB1 LEU B 58 6.249 -7.424 8.373 1.00 0.00

ATOM 1673 HB2 LEU B 58 7.308 -6.083 8.068 1.00 0.00

ATOM 1674 CG LEU B 58 5.340 -5.507 8.626 1.00 0.00

ATOM 1675 HG LEU B 58 5.660 -4.522 8.971 1.00 0.00

ATOM 1676 CD1 LEU B 58 4.910 -5.352 7.147 1.00 0.00

ATOM 1677 HD11 LEU B 58 5.596 -5.845 6.455 1.00 0.00

ATOM 1678 HD12 LEU B 58 3.919 -5.762 6.948 1.00 0.00

ATOM 1679 HD13 LEU B 58 4.876 -4.303 6.856 1.00 0.00

ATOM 1680 CD2 LEU B 58 4.163 -5.970 9.506 1.00 0.00

ATOM 1681 HD21 LEU B 58 4.387 -6.906 10.015 1.00 0.00

ATOM 1682 HD22 LEU B 58 3.925 -5.218 10.253 1.00 0.00

ATOM 1683 HD23 LEU B 58 3.258 -6.136 8.925 1.00 0.00

ATOM 1684 N MET B 59 7.737 -8.884 10.813 1.00 0.00

ATOM 1685 HN MET B 59 6.925 -8.785 11.386 1.00 0.00

ATOM 1686 CA MET B 59 8.490 -10.133 10.747 1.00 0.00

ATOM 1687 HA MET B 59 9.525 -9.888 10.536 1.00 0.00

ATOM 1688 C MET B 59 7.929 -11.062 9.643 1.00 0.00

ATOM 1689 O MET B 59 6.787 -11.513 9.724 1.00 0.00

ATOM 1690 CB MET B 59 8.427 -10.895 12.087 1.00 0.00

ATOM 1691 HB1 MET B 59 9.018 -11.807 11.998 1.00 0.00

ATOM 1692 HB2 MET B 59 7.394 -11.199 12.269 1.00 0.00

ATOM 1693 CG MET B 59 8.902 -10.116 13.325 1.00 0.00

ATOM 1694 HG1 MET B 59 8.946 -10.790 14.184 1.00 0.00

ATOM 1695 HG2 MET B 59 8.161 -9.356 13.578 1.00 0.00

ATOM 1696 SD MET B 59 10.513 -9.318 13.117 1.00 0.00

ATOM 1697 CE MET B 59 11.604 -10.751 13.091 1.00 0.00

ATOM 1698 HE1 MET B 59 11.058 -11.689 13.184 1.00 0.00

ATOM 1699 HE2 MET B 59 12.144 -10.750 12.147 1.00 0.00

ATOM 1700 HE3 MET B 59 12.353 -10.673 13.879 1.00 0.00

ATOM 1701 N TYR B 60 8.795 -11.414 8.668 1.00 0.00

ATOM 1702 HN TYR B 60 9.713 -11.008 8.653 1.00 0.00

ATOM 1703 CA TYR B 60 8.530 -12.563 7.799 1.00 0.00

ATOM 1704 HA TYR B 60 7.681 -13.112 8.206 1.00 0.00

ATOM 1705 C TYR B 60 9.770 -13.451 7.842 1.00 0.00

ATOM 1706 O TYR B 60 10.877 -12.949 7.751 1.00 0.00

ATOM 1707 CB TYR B 60 8.261 -12.137 6.354 1.00 0.00

ATOM 1708 HB1 TYR B 60 9.155 -11.691 5.913 1.00 0.00

ATOM 1709 HB2 TYR B 60 7.483 -11.375 6.312 1.00 0.00

ATOM 1710 CG TYR B 60 7.820 -13.296 5.501 1.00 0.00

ATOM 1711 CD1 TYR B 60 6.600 -13.914 5.740 1.00 0.00

ATOM 1712 HD1 TYR B 60 5.954 -13.565 6.523 1.00 0.00

ATOM 1713 CE1 TYR B 60 6.161 -14.943 4.925 1.00 0.00

ATOM 1714 HE1 TYR B 60 5.205 -15.410 5.110 1.00 0.00

ATOM 1715 CZ TYR B 60 6.924 -15.350 3.839 1.00 0.00

ATOM 1716 OH TYR B 60 6.423 -16.333 3.007 1.00 0.00

ATOM 1717 HH TYR B 60 6.760 -16.231 2.115 1.00 0.00

ATOM 1718 CE2 TYR B 60 8.161 -14.760 3.605 1.00 0.00

ATOM 1719 HE2 TYR B 60 8.796 -15.059 2.779 1.00 0.00

ATOM 1720 CD2 TYR B 60 8.604 -13.745 4.442 1.00 0.00

ATOM 1721 HD2 TYR B 60 9.562 -13.284 4.251 1.00 0.00

ATOM 1722 N GLN B 61 9.524 -14.763 8.062 1.00 0.00

ATOM 1723 HN GLN B 61 8.568 -15.041 8.071 1.00 0.00

ATOM 1724 CA GLN B 61 10.580 -15.775 8.096 1.00 0.00

ATOM 1725 HA GLN B 61 10.131 -16.661 8.546 1.00 0.00

ATOM 1726 C GLN B 61 11.733 -15.345 9.029 1.00 0.00

ATOM 1727 O GLN B 61 12.912 -15.595 8.812 1.00 0.00

ATOM 1728 CB GLN B 61 11.047 -16.143 6.680 1.00 0.00

ATOM 1729 HB1 GLN B 61 11.918 -16.789 6.771 1.00 0.00

ATOM 1730 HB2 GLN B 61 11.362 -15.244 6.145 1.00 0.00

ATOM 1731 CG GLN B 61 10.002 -16.913 5.853 1.00 0.00

ATOM 1732 HG1 GLN B 61 9.074 -16.347 5.789 1.00 0.00

ATOM 1733 HG2 GLN B 61 9.766 -17.862 6.340 1.00 0.00

ATOM 1734 CD GLN B 61 10.556 -17.195 4.438 1.00 0.00

ATOM 1735 OE1 GLN B 61 11.644 -16.754 4.076 1.00 0.00

ATOM 1736 NE2 GLN B 61 9.760 -17.958 3.685 1.00 0.00

ATOM 1737 HE21 GLN B 61 10.117 -18.200 2.776 1.00 0.00

ATOM 1738 HE22 GLN B 61 8.815 -18.220 3.897 1.00 0.00

ATOM 1739 N GLY B 62 11.320 -14.706 10.137 1.00 0.00

ATOM 1740 HN GLY B 62 10.392 -14.334 10.140 1.00 0.00

ATOM 1741 CA GLY B 62 12.315 -14.370 11.152 1.00 0.00

ATOM 1742 HA1 GLY B 62 13.041 -15.182 11.246 1.00 0.00

ATOM 1743 HA2 GLY B 62 11.791 -14.266 12.100 1.00 0.00

ATOM 1744 C GLY B 62 13.083 -13.075 10.854 1.00 0.00

ATOM 1745 O GLY B 62 13.753 -12.561 11.738 1.00 0.00

ATOM 1746 N LYS B 63 12.970 -12.593 9.602 1.00 0.00

ATOM 1747 HN LYS B 63 12.342 -13.008 8.941 1.00 0.00

ATOM 1748 CA LYS B 63 13.603 -11.364 9.152 1.00 0.00

ATOM 1749 HA LYS B 63 14.575 -11.276 9.638 1.00 0.00

ATOM 1750 C LYS B 63 12.699 -10.181 9.552 1.00 0.00

ATOM 1751 O LYS B 63 11.484 -10.246 9.401 1.00 0.00

ATOM 1752 CB LYS B 63 13.752 -11.406 7.615 1.00 0.00

ATOM 1753 HB1 LYS B 63 14.208 -10.475 7.272 1.00 0.00

ATOM 1754 HB2 LYS B 63 12.758 -11.438 7.165 1.00 0.00

ATOM 1755 CG LYS B 63 14.552 -12.599 7.052 1.00 0.00

ATOM 1756 HG1 LYS B 63 14.095 -13.531 7.394 1.00 0.00

ATOM 1757 HG2 LYS B 63 15.569 -12.570 7.443 1.00 0.00

ATOM 1758 CD LYS B 63 14.550 -12.577 5.511 1.00 0.00

ATOM 1759 HD1 LYS B 63 14.993 -11.648 5.147 1.00 0.00

ATOM 1760 HD2 LYS B 63 13.508 -12.566 5.185 1.00 0.00

ATOM 1761 CE LYS B 63 15.219 -13.779 4.829 1.00 0.00

ATOM 1762 HE1 LYS B 63 14.936 -13.803 3.773 1.00 0.00

ATOM 1763 HE2 LYS B 63 14.872 -14.716 5.269 1.00 0.00

ATOM 1764 NZ LYS B 63 16.678 -13.748 4.884 1.00 0.00

ATOM 1765 HZ1 LYS B 63 16.993 -12.949 5.458 1.00 0.00

ATOM 1766 HZ2 LYS B 63 17.022 -14.639 5.288 1.00 0.00

ATOM 1767 HZ3 LYS B 63 17.070 -13.607 3.932 1.00 0.00

ATOM 1768 N ARG B 64 13.369 -9.127 10.045 1.00 0.00

ATOM 1769 HN ARG B 64 14.366 -9.112 10.040 1.00 0.00

ATOM 1770 CA ARG B 64 12.723 -7.831 10.236 1.00 0.00

ATOM 1771 HA ARG B 64 11.735 -7.961 10.681 1.00 0.00

ATOM 1772 C ARG B 64 12.614 -7.192 8.841 1.00 0.00

ATOM 1773 O ARG B 64 13.584 -7.231 8.098 1.00 0.00

ATOM 1774 CB ARG B 64 13.650 -6.965 11.116 1.00 0.00

ATOM 1775 HB1 ARG B 64 13.522 -5.929 10.814 1.00 0.00

ATOM 1776 HB2 ARG B 64 14.691 -7.223 10.916 1.00 0.00

ATOM 1777 CG ARG B 64 13.379 -7.045 12.628 1.00 0.00

ATOM 1778 HG1 ARG B 64 13.454 -8.082 12.954 1.00 0.00

ATOM 1779 HG2 ARG B 64 12.351 -6.721 12.801 1.00 0.00

ATOM 1780 CD ARG B 64 14.326 -6.166 13.471 1.00 0.00

ATOM 1781 HD1 ARG B 64 15.372 -6.432 13.304 1.00 0.00

ATOM 1782 HD2 ARG B 64 14.105 -6.245 14.538 1.00 0.00

ATOM 1783 NE ARG B 64 14.170 -4.771 13.080 1.00 0.00

ATOM 1784 HE ARG B 64 13.691 -4.539 12.225 1.00 0.00

ATOM 1785 CZ ARG B 64 14.691 -3.694 13.708 1.00 0.00

ATOM 1786 NH1 ARG B 64 15.382 -3.863 14.835 1.00 0.00

ATOM 1787 HH11 ARG B 64 15.496 -4.797 15.173 1.00 0.00

ATOM 1788 HH12 ARG B 64 15.786 -3.091 15.332 1.00 0.00

ATOM 1789 NH2 ARG B 64 14.517 -2.488 13.193 1.00 0.00

ATOM 1790 HH21 ARG B 64 14.084 -2.394 12.281 1.00 0.00

ATOM 1791 HH22 ARG B 64 14.809 -1.628 13.598 1.00 0.00

ATOM 1792 N LEU B 65 11.452 -6.634 8.499 1.00 0.00

ATOM 1793 HN LEU B 65 10.680 -6.641 9.131 1.00 0.00

ATOM 1794 CA LEU B 65 11.271 -5.857 7.274 1.00 0.00

ATOM 1795 HA LEU B 65 12.166 -5.920 6.648 1.00 0.00

ATOM 1796 C LEU B 65 11.005 -4.419 7.738 1.00 0.00

ATOM 1797 O LEU B 65 10.108 -4.182 8.536 1.00 0.00

ATOM 1798 CB LEU B 65 10.030 -6.339 6.503 1.00 0.00

ATOM 1799 HB1 LEU B 65 9.824 -5.666 5.666 1.00 0.00

ATOM 1800 HB2 LEU B 65 9.185 -6.254 7.177 1.00 0.00

ATOM 1801 CG LEU B 65 10.079 -7.788 5.984 1.00 0.00

ATOM 1802 HG LEU B 65 10.449 -8.416 6.796 1.00 0.00

ATOM 1803 CD1 LEU B 65 8.657 -8.295 5.641 1.00 0.00

ATOM 1804 HD11 LEU B 65 7.883 -7.585 5.938 1.00 0.00

ATOM 1805 HD12 LEU B 65 8.506 -8.465 4.575 1.00 0.00

ATOM 1806 HD13 LEU B 65 8.442 -9.238 6.142 1.00 0.00

ATOM 1807 CD2 LEU B 65 11.046 -7.929 4.791 1.00 0.00

ATOM 1808 HD21 LEU B 65 11.525 -6.976 4.556 1.00 0.00

ATOM 1809 HD22 LEU B 65 11.833 -8.652 4.999 1.00 0.00

ATOM 1810 HD23 LEU B 65 10.543 -8.251 3.877 1.00 0.00

ATOM 1811 N GLU B 66 11.832 -3.501 7.224 1.00 0.00

ATOM 1812 HN GLU B 66 12.469 -3.714 6.477 1.00 0.00

ATOM 1813 CA GLU B 66 11.703 -2.083 7.554 1.00 0.00

ATOM 1814 HA GLU B 66 11.200 -2.019 8.518 1.00 0.00

ATOM 1815 C GLU B 66 10.843 -1.373 6.432 1.00 0.00

ATOM 1816 O GLU B 66 10.756 -1.849 5.301 1.00 0.00

ATOM 1817 CB GLU B 66 13.113 -1.475 7.743 1.00 0.00

ATOM 1818 HB1 GLU B 66 13.033 -0.500 8.224 1.00 0.00

ATOM 1819 HB2 GLU B 66 13.527 -1.298 6.749 1.00 0.00

ATOM 1820 CG GLU B 66 14.163 -2.320 8.523 1.00 0.00

ATOM 1821 HG1 GLU B 66 15.131 -1.826 8.392 1.00 0.00

ATOM 1822 HG2 GLU B 66 14.268 -3.304 8.067 1.00 0.00

ATOM 1823 CD GLU B 66 13.956 -2.460 10.048 1.00 0.00

ATOM 1824 OE1 GLU B 66 14.025 -1.465 10.764 1.00 0.00

ATOM 1825 OE2 GLU B 66 13.776 -3.571 10.555 1.00 0.00

ATOM 1826 N PRO B 67 10.137 -0.263 6.824 1.00 0.00

ATOM 1827 CA PRO B 67 9.312 0.550 5.910 1.00 0.00

ATOM 1828 HA PRO B 67 8.781 -0.089 5.202 1.00 0.00

ATOM 1829 CD PRO B 67 10.011 0.149 8.221 1.00 0.00

ATOM 1830 HD1 PRO B 67 10.989 0.349 8.659 1.00 0.00

ATOM 1831 HD2 PRO B 67 9.520 -0.645 8.786 1.00 0.00

ATOM 1832 C PRO B 67 10.078 1.649 5.126 1.00 0.00

ATOM 1833 O PRO B 67 10.706 2.529 5.701 1.00 0.00

ATOM 1834 CB PRO B 67 8.344 1.258 6.873 1.00 0.00

ATOM 1835 HB1 PRO B 67 7.474 0.628 7.049 1.00 0.00

ATOM 1836 HB2 PRO B 67 7.988 2.217 6.494 1.00 0.00

ATOM 1837 CG PRO B 67 9.119 1.390 8.189 1.00 0.00

ATOM 1838 HG1 PRO B 67 8.473 1.465 9.066 1.00 0.00

ATOM 1839 HG2 PRO B 67 9.750 2.278 8.153 1.00 0.00

ATOM 1840 N SER B 68 9.966 1.580 3.805 1.00 0.00

ATOM 1841 HN SER B 68 9.320 0.891 3.492 1.00 0.00

ATOM 1842 CA SER B 68 10.780 2.311 2.835 1.00 0.00

ATOM 1843 HA SER B 68 11.712 2.592 3.326 1.00 0.00

ATOM 1844 C SER B 68 10.112 3.557 2.224 1.00 0.00

ATOM 1845 O SER B 68 10.788 4.434 1.692 1.00 0.00

ATOM 1846 CB SER B 68 11.047 1.282 1.731 1.00 0.00

ATOM 1847 HB1 SER B 68 11.965 1.516 1.167 1.00 0.00

ATOM 1848 HB2 SER B 68 10.231 1.326 1.000 1.00 0.00

ATOM 1849 OG SER B 68 11.078 -0.029 2.316 1.00 0.00

ATOM 1850 HG SER B 68 11.284 0.065 3.262 1.00 0.00

ATOM 1851 N TRP B 69 8.763 3.599 2.283 1.00 0.00

ATOM 1852 HN TRP B 69 8.210 2.838 2.636 1.00 0.00

ATOM 1853 CA TRP B 69 8.056 4.728 1.693 1.00 0.00

ATOM 1854 HA TRP B 69 8.562 5.624 2.056 1.00 0.00

ATOM 1855 C TRP B 69 6.601 4.727 2.214 1.00 0.00

ATOM 1856 O TRP B 69 5.924 3.710 2.288 1.00 0.00

ATOM 1857 CB TRP B 69 8.163 4.641 0.153 1.00 0.00

ATOM 1858 HB1 TRP B 69 7.876 3.648 -0.175 1.00 0.00

ATOM 1859 HB2 TRP B 69 9.196 4.790 -0.159 1.00 0.00

ATOM 1860 CG TRP B 69 7.284 5.655 -0.541 1.00 0.00

ATOM 1861 CD1 TRP B 69 7.678 6.890 -1.060 1.00 0.00

ATOM 1862 HD1 TRP B 69 8.695 7.265 -1.046 1.00 0.00

ATOM 1863 NE1 TRP B 69 6.584 7.516 -1.574 1.00 0.00

ATOM 1864 HE1 TRP B 69 6.599 8.393 -2.002 1.00 0.00

ATOM 1865 CE2 TRP B 69 5.449 6.727 -1.413 1.00 0.00

ATOM 1866 CD2 TRP B 69 5.867 5.539 -0.770 1.00 0.00

ATOM 1867 CE3 TRP B 69 4.963 4.553 -0.479 1.00 0.00

ATOM 1868 HE3 TRP B 69 5.279 3.632 -0.003 1.00 0.00

ATOM 1869 CZ3 TRP B 69 3.621 4.724 -0.799 1.00 0.00

ATOM 1870 HZ3 TRP B 69 2.906 3.952 -0.562 1.00 0.00

ATOM 1871 CH2 TRP B 69 3.202 5.896 -1.424 1.00 0.00

ATOM 1872 HH2 TRP B 69 2.156 6.010 -1.672 1.00 0.00

ATOM 1873 CZ2 TRP B 69 4.124 6.900 -1.730 1.00 0.00

ATOM 1874 HZ2 TRP B 69 3.778 7.806 -2.209 1.00 0.00

ATOM 1875 N ALA B 70 6.124 5.931 2.600 1.00 0.00

ATOM 1876 HN ALA B 70 6.760 6.688 2.461 1.00 0.00

ATOM 1877 CA ALA B 70 4.757 6.017 3.124 1.00 0.00

ATOM 1878 HA ALA B 70 4.181 5.170 2.750 1.00 0.00

ATOM 1879 C ALA B 70 4.094 7.297 2.607 1.00 0.00

ATOM 1880 O ALA B 70 4.788 8.279 2.388 1.00 0.00

ATOM 1881 CB ALA B 70 4.805 5.985 4.664 1.00 0.00

ATOM 1882 HB1 ALA B 70 5.810 5.779 5.040 1.00 0.00

ATOM 1883 HB2 ALA B 70 4.473 6.919 5.120 1.00 0.00

ATOM 1884 HB3 ALA B 70 4.173 5.180 5.044 1.00 0.00

ATOM 1885 N SER B 71 2.761 7.234 2.463 1.00 0.00

ATOM 1886 HN SER B 71 2.347 6.329 2.611 1.00 0.00

ATOM 1887 CA SER B 71 1.934 8.411 2.218 1.00 0.00

ATOM 1888 HA SER B 71 2.531 9.309 2.388 1.00 0.00

ATOM 1889 C SER B 71 0.786 8.325 3.218 1.00 0.00

ATOM 1890 O SER B 71 -0.107 7.497 3.075 1.00 0.00

ATOM 1891 CB SER B 71 1.334 8.421 0.796 1.00 0.00

ATOM 1892 HB1 SER B 71 0.755 7.509 0.647 1.00 0.00

ATOM 1893 HB2 SER B 71 2.117 8.393 0.025 1.00 0.00

ATOM 1894 OG SER B 71 0.448 9.528 0.594 1.00 0.00

ATOM 1895 HG SER B 71 0.970 10.328 0.461 1.00 0.00

ATOM 1896 N VAL B 72 0.821 9.241 4.194 1.00 0.00

ATOM 1897 HN VAL B 72 1.561 9.911 4.263 1.00 0.00

ATOM 1898 CA VAL B 72 -0.284 9.260 5.139 1.00 0.00

ATOM 1899 HA VAL B 72 -0.417 8.234 5.496 1.00 0.00

ATOM 1900 C VAL B 72 -1.591 9.709 4.446 1.00 0.00

ATOM 1901 O VAL B 72 -2.667 9.197 4.725 1.00 0.00

ATOM 1902 CB VAL B 72 0.080 10.186 6.310 1.00 0.00

ATOM 1903 HB VAL B 72 0.047 11.221 5.957 1.00 0.00

ATOM 1904 CG1 VAL B 72 -0.949 10.068 7.448 1.00 0.00

ATOM 1905 HG11 VAL B 72 -1.875 9.591 7.114 1.00 0.00

ATOM 1906 HG12 VAL B 72 -0.568 9.489 8.290 1.00 0.00

ATOM 1907 HG13 VAL B 72 -1.196 11.063 7.817 1.00 0.00

ATOM 1908 CG2 VAL B 72 1.521 9.937 6.806 1.00 0.00

ATOM 1909 HG21 VAL B 72 1.893 8.949 6.518 1.00 0.00

ATOM 1910 HG22 VAL B 72 2.211 10.689 6.406 1.00 0.00

ATOM 1911 HG23 VAL B 72 1.585 9.993 7.895 1.00 0.00

ATOM 1912 N LYS B 73 -1.428 10.662 3.504 1.00 0.00

ATOM 1913 HN LYS B 73 -0.497 10.891 3.231 1.00 0.00

ATOM 1914 CA LYS B 73 -2.611 11.151 2.821 1.00 0.00

ATOM 1915 HA LYS B 73 -3.308 11.451 3.605 1.00 0.00

ATOM 1916 C LYS B 73 -3.311 9.979 2.094 1.00 0.00

ATOM 1917 O LYS B 73 -4.369 9.512 2.510 1.00 0.00

ATOM 1918 CB LYS B 73 -2.322 12.443 2.033 1.00 0.00

ATOM 1919 HB1 LYS B 73 -2.190 13.228 2.782 1.00 0.00

ATOM 1920 HB2 LYS B 73 -3.224 12.710 1.485 1.00 0.00

ATOM 1921 CG LYS B 73 -1.121 12.543 1.067 1.00 0.00

ATOM 1922 HG1 LYS B 73 -1.227 11.815 0.260 1.00 0.00

ATOM 1923 HG2 LYS B 73 -0.185 12.345 1.595 1.00 0.00

ATOM 1924 CD LYS B 73 -1.139 13.982 0.508 1.00 0.00

ATOM 1925 HD1 LYS B 73 -1.103 14.682 1.348 1.00 0.00

ATOM 1926 HD2 LYS B 73 -2.113 14.136 0.035 1.00 0.00

ATOM 1927 CE LYS B 73 -0.075 14.402 -0.506 1.00 0.00

ATOM 1928 HE1 LYS B 73 -0.114 13.756 -1.390 1.00 0.00

ATOM 1929 HE2 LYS B 73 0.937 14.372 -0.078 1.00 0.00

ATOM 1930 NZ LYS B 73 -0.446 15.777 -0.897 1.00 0.00

ATOM 1931 HZ1 LYS B 73 -1.342 15.998 -0.406 1.00 0.00

ATOM 1932 HZ2 LYS B 73 0.203 16.521 -0.569 1.00 0.00

ATOM 1933 HZ3 LYS B 73 -0.663 15.837 -1.922 1.00 0.00

ATOM 1934 N LYS B 74 -2.594 9.469 1.068 1.00 0.00

ATOM 1935 HN LYS B 74 -1.713 9.882 0.815 1.00 0.00

ATOM 1936 CA LYS B 74 -3.193 8.330 0.363 1.00 0.00

ATOM 1937 HA LYS B 74 -4.184 8.672 0.049 1.00 0.00

ATOM 1938 C LYS B 74 -3.472 7.082 1.278 1.00 0.00

ATOM 1939 O LYS B 74 -4.229 6.207 0.884 1.00 0.00

ATOM 1940 CB LYS B 74 -2.377 8.014 -0.913 1.00 0.00

ATOM 1941 HB1 LYS B 74 -2.650 7.017 -1.265 1.00 0.00

ATOM 1942 HB2 LYS B 74 -1.313 7.968 -0.676 1.00 0.00

ATOM 1943 CG LYS B 74 -2.663 9.047 -2.030 1.00 0.00

ATOM 1944 HG1 LYS B 74 -2.588 10.053 -1.609 1.00 0.00

ATOM 1945 HG2 LYS B 74 -3.701 8.909 -2.345 1.00 0.00

ATOM 1946 CD LYS B 74 -1.742 8.943 -3.255 1.00 0.00

ATOM 1947 HD1 LYS B 74 -1.678 7.891 -3.552 1.00 0.00

ATOM 1948 HD2 LYS B 74 -0.728 9.239 -2.969 1.00 0.00

ATOM 1949 CE LYS B 74 -2.198 9.780 -4.482 1.00 0.00

ATOM 1950 HE1 LYS B 74 -3.093 9.335 -4.930 1.00 0.00

ATOM 1951 HE2 LYS B 74 -1.421 9.709 -5.249 1.00 0.00

ATOM 1952 NZ LYS B 74 -2.464 11.215 -4.230 1.00 0.00

ATOM 1953 HZ1 LYS B 74 -1.901 11.545 -3.420 1.00 0.00

ATOM 1954 HZ2 LYS B 74 -2.193 11.860 -5.019 1.00 0.00

ATOM 1955 HZ3 LYS B 74 -3.468 11.440 -4.026 1.00 0.00

ATOM 1956 N ASP B 75 -2.872 7.033 2.482 1.00 0.00

ATOM 1957 HN ASP B 75 -2.322 7.804 2.779 1.00 0.00

ATOM 1958 CA ASP B 75 -3.094 5.946 3.453 1.00 0.00

ATOM 1959 HA ASP B 75 -2.553 6.265 4.340 1.00 0.00

ATOM 1960 C ASP B 75 -2.437 4.631 2.947 1.00 0.00

ATOM 1961 O ASP B 75 -3.016 3.552 2.960 1.00 0.00

ATOM 1962 CB ASP B 75 -4.600 5.844 3.831 1.00 0.00

ATOM 1963 HB1 ASP B 75 -5.122 5.279 3.062 1.00 0.00

ATOM 1964 HB2 ASP B 75 -4.994 6.858 3.821 1.00 0.00

ATOM 1965 CG ASP B 75 -4.980 5.275 5.211 1.00 0.00

ATOM 1966 OD1 ASP B 75 -4.246 5.491 6.187 1.00 0.00

ATOM 1967 OD2 ASP B 75 -6.066 4.675 5.333 1.00 0.00

ATOM 1968 N LEU B 76 -1.158 4.770 2.513 1.00 0.00

ATOM 1969 HN LEU B 76 -0.710 5.642 2.713 1.00 0.00

ATOM 1970 CA LEU B 76 -0.395 3.664 1.900 1.00 0.00

ATOM 1971 HA LEU B 76 -0.897 2.713 2.073 1.00 0.00

ATOM 1972 C LEU B 76 1.029 3.595 2.478 1.00 0.00

ATOM 1973 O LEU B 76 1.580 4.616 2.867 1.00 0.00

ATOM 1974 CB LEU B 76 -0.206 3.946 0.402 1.00 0.00

ATOM 1975 HB1 LEU B 76 0.482 3.212 -0.018 1.00 0.00

ATOM 1976 HB2 LEU B 76 0.278 4.914 0.277 1.00 0.00

ATOM 1977 CG LEU B 76 -1.478 3.935 -0.449 1.00 0.00

ATOM 1978 HG LEU B 76 -2.159 4.694 -0.062 1.00 0.00

ATOM 1979 CD1 LEU B 76 -1.129 4.330 -1.900 1.00 0.00

ATOM 1980 HD11 LEU B 76 -0.082 4.617 -2.009 1.00 0.00

ATOM 1981 HD12 LEU B 76 -1.310 3.522 -2.607 1.00 0.00

ATOM 1982 HD13 LEU B 76 -1.718 5.184 -2.231 1.00 0.00

ATOM 1983 CD2 LEU B 76 -2.192 2.574 -0.360 1.00 0.00

ATOM 1984 HD21 LEU B 76 -1.538 1.801 0.038 1.00 0.00

ATOM 1985 HD22 LEU B 76 -3.043 2.622 0.321 1.00 0.00

ATOM 1986 HD23 LEU B 76 -2.559 2.229 -1.327 1.00 0.00

ATOM 1987 N ILE B 77 1.655 2.401 2.452 1.00 0.00

ATOM 1988 HN ILE B 77 1.207 1.567 2.115 1.00 0.00

ATOM 1989 CA ILE B 77 3.064 2.245 2.867 1.00 0.00

ATOM 1990 HA ILE B 77 3.583 3.129 2.509 1.00 0.00

ATOM 1991 C ILE B 77 3.681 1.017 2.143 1.00 0.00

ATOM 1992 O ILE B 77 2.920 0.122 1.754 1.00 0.00

ATOM 1993 CB ILE B 77 3.147 2.159 4.414 1.00 0.00

ATOM 1994 HB ILE B 77 2.703 3.078 4.794 1.00 0.00

ATOM 1995 CG2 ILE B 77 2.344 0.990 5.002 1.00 0.00

ATOM 1996 HG21 ILE B 77 1.742 0.482 4.275 1.00 0.00

ATOM 1997 HG22 ILE B 77 2.985 0.223 5.414 1.00 0.00

ATOM 1998 HG23 ILE B 77 1.710 1.312 5.818 1.00 0.00

ATOM 1999 CG1 ILE B 77 4.590 2.118 4.950 1.00 0.00

ATOM 2000 HG11 ILE B 77 5.156 2.958 4.548 1.00 0.00

ATOM 2001 HG12 ILE B 77 5.085 1.218 4.585 1.00 0.00

ATOM 2002 CD1 ILE B 77 4.693 2.131 6.483 1.00 0.00

ATOM 2003 HD11 ILE B 77 3.714 2.131 6.960 1.00 0.00

ATOM 2004 HD12 ILE B 77 5.223 1.246 6.839 1.00 0.00

ATOM 2005 HD13 ILE B 77 5.246 3.003 6.834 1.00 0.00

ATOM 2006 N SER B 78 5.034 1.072 1.961 1.00 0.00

ATOM 2007 HN SER B 78 5.569 1.812 2.386 1.00 0.00

ATOM 2008 CA SER B 78 5.810 0.023 1.279 1.00 0.00

ATOM 2009 HA SER B 78 5.184 -0.870 1.301 1.00 0.00

ATOM 2010 C SER B 78 7.106 -0.290 2.059 1.00 0.00

ATOM 2011 O SER B 78 7.772 0.624 2.553 1.00 0.00

ATOM 2012 CB SER B 78 6.120 0.420 -0.184 1.00 0.00

ATOM 2013 HB1 SER B 78 5.178 0.682 -0.653 1.00 0.00

ATOM 2014 HB2 SER B 78 6.447 -0.484 -0.719 1.00 0.00

ATOM 2015 OG SER B 78 7.018 1.523 -0.397 1.00 0.00

ATOM 2016 HG SER B 78 7.923 1.170 -0.458 1.00 0.00

ATOM 2017 N TYR B 79 7.388 -1.606 2.148 1.00 0.00

ATOM 2018 HN TYR B 79 6.863 -2.210 1.555 1.00 0.00

ATOM 2019 CA TYR B 79 8.520 -2.233 2.809 1.00 0.00

ATOM 2020 HA TYR B 79 9.104 -1.466 3.311 1.00 0.00

ATOM 2021 C TYR B 79 9.366 -2.924 1.702 1.00 0.00

ATOM 2022 O TYR B 79 8.869 -3.714 0.904 1.00 0.00

ATOM 2023 CB TYR B 79 8.022 -3.279 3.831 1.00 0.00

ATOM 2024 HB1 TYR B 79 8.869 -3.855 4.195 1.00 0.00

ATOM 2025 HB2 TYR B 79 7.391 -3.984 3.308 1.00 0.00

ATOM 2026 CG TYR B 79 7.256 -2.716 5.010 1.00 0.00

ATOM 2027 CD1 TYR B 79 7.805 -2.707 6.292 1.00 0.00

ATOM 2028 HD1 TYR B 79 8.792 -3.111 6.451 1.00 0.00

ATOM 2029 CE1 TYR B 79 7.105 -2.188 7.378 1.00 0.00

ATOM 2030 HE1 TYR B 79 7.585 -2.192 8.350 1.00 0.00

ATOM 2031 CZ TYR B 79 5.822 -1.679 7.194 1.00 0.00

ATOM 2032 OH TYR B 79 5.087 -1.173 8.248 1.00 0.00

ATOM 2033 HH TYR B 79 5.370 -1.569 9.067 1.00 0.00

ATOM 2034 CE2 TYR B 79 5.274 -1.690 5.916 1.00 0.00

ATOM 2035 HE2 TYR B 79 4.288 -1.313 5.723 1.00 0.00

ATOM 2036 CD2 TYR B 79 5.975 -2.207 4.840 1.00 0.00

ATOM 2037 HD2 TYR B 79 5.489 -2.221 3.872 1.00 0.00

ATOM 2038 N GLY B 80 10.674 -2.583 1.745 1.00 0.00

ATOM 2039 HN GLY B 80 10.941 -1.934 2.462 1.00 0.00

ATOM 2040 CA GLY B 80 11.675 -3.186 0.861 1.00 0.00

ATOM 2041 HA1 GLY B 80 11.442 -4.236 0.697 1.00 0.00

ATOM 2042 HA2 GLY B 80 12.618 -3.113 1.401 1.00 0.00

ATOM 2043 C GLY B 80 11.886 -2.502 -0.505 1.00 0.00

ATOM 2044 O GLY B 80 12.659 -2.999 -1.325 1.00 0.00

ATOM 2045 N GLY B 81 11.259 -1.313 -0.668 1.00 0.00

ATOM 2046 HN GLY B 81 10.669 -0.930 0.052 1.00 0.00

ATOM 2047 CA GLY B 81 11.518 -0.538 -1.887 1.00 0.00

ATOM 2048 HA1 GLY B 81 11.619 -1.210 -2.746 1.00 0.00

ATOM 2049 HA2 GLY B 81 12.458 0.004 -1.740 1.00 0.00

ATOM 2050 C GLY B 81 10.364 0.447 -2.109 1.00 0.00

ATOM 2051 O GLY B 81 9.544 0.668 -1.228 1.00 0.00

ATOM 2052 N GLY B 82 10.329 1.087 -3.297 1.00 0.00

ATOM 2053 HN GLY B 82 10.917 0.745 -4.034 1.00 0.00

ATOM 2054 CA GLY B 82 9.225 2.035 -3.518 1.00 0.00

ATOM 2055 HA1 GLY B 82 9.542 2.731 -4.295 1.00 0.00

ATOM 2056 HA2 GLY B 82 9.037 2.591 -2.595 1.00 0.00

ATOM 2057 C GLY B 82 7.954 1.285 -3.951 1.00 0.00

ATOM 2058 O GLY B 82 7.971 0.074 -4.109 1.00 0.00

ATOM 2059 N TRP B 83 6.874 2.060 -4.170 1.00 0.00

ATOM 2060 HN TRP B 83 6.985 3.040 -4.020 1.00 0.00

ATOM 2061 CA TRP B 83 5.633 1.451 -4.660 1.00 0.00

ATOM 2062 HA TRP B 83 5.359 0.664 -3.955 1.00 0.00

ATOM 2063 C TRP B 83 5.863 0.842 -6.073 1.00 0.00

ATOM 2064 O TRP B 83 6.364 1.520 -6.970 1.00 0.00

ATOM 2065 CB TRP B 83 4.594 2.583 -4.682 1.00 0.00

ATOM 2066 HB1 TRP B 83 4.734 3.205 -5.565 1.00 0.00

ATOM 2067 HB2 TRP B 83 4.754 3.242 -3.827 1.00 0.00

ATOM 2068 CG TRP B 83 3.164 2.084 -4.627 1.00 0.00

ATOM 2069 CD1 TRP B 83 2.270 2.021 -5.697 1.00 0.00

ATOM 2070 HD1 TRP B 83 2.508 2.256 -6.727 1.00 0.00

ATOM 2071 NE1 TRP B 83 1.060 1.616 -5.236 1.00 0.00

ATOM 2072 HE1 TRP B 83 0.260 1.469 -5.784 1.00 0.00

ATOM 2073 CE2 TRP B 83 1.106 1.374 -3.873 1.00 0.00

ATOM 2074 CD2 TRP B 83 2.420 1.688 -3.453 1.00 0.00

ATOM 2075 CE3 TRP B 83 2.772 1.518 -2.143 1.00 0.00

ATOM 2076 HE3 TRP B 83 3.783 1.750 -1.843 1.00 0.00

ATOM 2077 CZ3 TRP B 83 1.819 1.107 -1.211 1.00 0.00

ATOM 2078 HZ3 TRP B 83 2.091 0.943 -0.181 1.00 0.00

ATOM 2079 CH2 TRP B 83 0.517 0.828 -1.622 1.00 0.00

ATOM 2080 HH2 TRP B 83 -0.222 0.519 -0.899 1.00 0.00

ATOM 2081 CZ2 TRP B 83 0.155 0.989 -2.960 1.00 0.00

ATOM 2082 HZ2 TRP B 83 -0.868 0.777 -3.259 1.00 0.00

ATOM 2083 N ARG B 84 5.493 -0.441 -6.210 1.00 0.00

ATOM 2084 HN ARG B 84 5.127 -0.888 -5.397 1.00 0.00

ATOM 2085 CA ARG B 84 5.660 -1.200 -7.447 1.00 0.00

ATOM 2086 HA ARG B 84 6.518 -0.801 -7.980 1.00 0.00

ATOM 2087 C ARG B 84 4.449 -1.045 -8.389 1.00 0.00

ATOM 2088 O ARG B 84 4.610 -0.892 -9.599 1.00 0.00

ATOM 2089 CB ARG B 84 5.874 -2.689 -7.109 1.00 0.00

ATOM 2090 HB1 ARG B 84 5.935 -3.274 -8.030 1.00 0.00

ATOM 2091 HB2 ARG B 84 4.992 -3.054 -6.584 1.00 0.00

ATOM 2092 CG ARG B 84 7.110 -2.998 -6.243 1.00 0.00

ATOM 2093 HG1 ARG B 84 6.914 -3.903 -5.675 1.00 0.00

ATOM 2094 HG2 ARG B 84 7.296 -2.222 -5.502 1.00 0.00

ATOM 2095 CD ARG B 84 8.355 -3.262 -7.086 1.00 0.00

ATOM 2096 HD1 ARG B 84 8.118 -3.979 -7.864 1.00 0.00

ATOM 2097 HD2 ARG B 84 9.187 -3.678 -6.523 1.00 0.00

ATOM 2098 NE ARG B 84 8.831 -2.007 -7.638 1.00 0.00

ATOM 2099 HE ARG B 84 9.035 -1.339 -6.917 1.00 0.00

ATOM 2100 CZ ARG B 84 8.955 -1.781 -8.959 1.00 0.00

ATOM 2101 NH1 ARG B 84 8.734 -2.767 -9.836 1.00 0.00

ATOM 2102 HH11 ARG B 84 8.590 -3.719 -9.508 1.00 0.00

ATOM 2103 HH12 ARG B 84 8.695 -2.609 -10.821 1.00 0.00

ATOM 2104 NH2 ARG B 84 9.283 -0.547 -9.329 1.00 0.00

ATOM 2105 HH21 ARG B 84 9.491 0.150 -8.647 1.00 0.00

ATOM 2106 HH22 ARG B 84 9.327 -0.282 -10.289 1.00 0.00

ATOM 2107 N PHE B 85 3.235 -1.123 -7.775 1.00 0.00

ATOM 2108 HN PHE B 85 3.240 -1.166 -6.770 1.00 0.00

ATOM 2109 CA PHE B 85 1.992 -1.325 -8.552 1.00 0.00

ATOM 2110 HA PHE B 85 2.037 -2.337 -8.953 1.00 0.00

ATOM 2111 C PHE B 85 1.888 -0.333 -9.737 1.00 0.00

ATOM 2112 O PHE B 85 2.160 0.854 -9.578 1.00 0.00

ATOM 2113 CB PHE B 85 0.720 -1.138 -7.697 1.00 0.00

ATOM 2114 HB1 PHE B 85 -0.144 -1.331 -8.333 1.00 0.00

ATOM 2115 HB2 PHE B 85 0.667 -0.099 -7.379 1.00 0.00

ATOM 2116 CG PHE B 85 0.550 -2.002 -6.470 1.00 0.00

ATOM 2117 CD1 PHE B 85 1.197 -3.224 -6.324 1.00 0.00

ATOM 2118 HD1 PHE B 85 1.858 -3.584 -7.088 1.00 0.00

ATOM 2119 CE1 PHE B 85 1.003 -4.007 -5.195 1.00 0.00

ATOM 2120 HE1 PHE B 85 1.549 -4.937 -5.087 1.00 0.00

ATOM 2121 CZ PHE B 85 0.130 -3.588 -4.201 1.00 0.00

ATOM 2122 HZ PHE B 85 -0.035 -4.217 -3.339 1.00 0.00

ATOM 2123 CE2 PHE B 85 -0.517 -2.366 -4.325 1.00 0.00

ATOM 2124 HE2 PHE B 85 -1.179 -2.037 -3.536 1.00 0.00

ATOM 2125 CD2 PHE B 85 -0.302 -1.578 -5.452 1.00 0.00

ATOM 2126 HD2 PHE B 85 -0.814 -0.631 -5.528 1.00 0.00

ATOM 2127 N GLN B 86 1.505 -0.856 -10.918 1.00 0.00

ATOM 2128 HN GLN B 86 1.230 -1.814 -10.968 1.00 0.00

ATOM 2129 CA GLN B 86 1.421 0.000 -12.106 1.00 0.00

ATOM 2130 HA GLN B 86 1.753 1.012 -11.869 1.00 0.00

ATOM 2131 C GLN B 86 -0.029 0.145 -12.589 1.00 0.00

ATOM 2132 O GLN B 86 -0.297 0.914 -13.500 1.00 0.00

ATOM 2133 CB GLN B 86 2.292 -0.569 -13.241 1.00 0.00

ATOM 2134 HB1 GLN B 86 2.146 0.032 -14.142 1.00 0.00

ATOM 2135 HB2 GLN B 86 1.924 -1.570 -13.471 1.00 0.00

ATOM 2136 CG GLN B 86 3.799 -0.655 -12.924 1.00 0.00

ATOM 2137 HG1 GLN B 86 4.280 -1.281 -13.678 1.00 0.00

ATOM 2138 HG2 GLN B 86 3.956 -1.147 -11.966 1.00 0.00

ATOM 2139 CD GLN B 86 4.519 0.705 -12.912 1.00 0.00

ATOM 2140 OE1 GLN B 86 3.921 1.770 -12.937 1.00 0.00

ATOM 2141 NE2 GLN B 86 5.850 0.607 -13.014 1.00 0.00

ATOM 2142 HE21 GLN B 86 6.321 1.460 -13.225 1.00 0.00

ATOM 2143 HE22 GLN B 86 6.280 -0.287 -12.871 1.00 0.00

ATOM 2144 N GLY B 87 -0.968 -0.612 -11.989 1.00 0.00

ATOM 2145 HN GLY B 87 -0.838 -1.187 -11.184 1.00 0.00

ATOM 2146 CA GLY B 87 -2.332 -0.556 -12.515 1.00 0.00

ATOM 2147 HA1 GLY B 87 -2.911 -1.310 -11.986 1.00 0.00

ATOM 2148 HA2 GLY B 87 -2.310 -0.812 -13.575 1.00 0.00

ATOM 2149 C GLY B 87 -2.959 0.828 -12.298 1.00 0.00

ATOM 2150 O GLY B 87 -2.695 1.504 -11.307 1.00 0.00

ATOM 2151 N SER B 88 -3.780 1.245 -13.272 1.00 0.00

ATOM 2152 HN SER B 88 -3.942 0.716 -14.111 1.00 0.00

ATOM 2153 CA SER B 88 -4.570 2.463 -13.140 1.00 0.00

ATOM 2154 HA SER B 88 -4.562 2.771 -12.091 1.00 0.00

ATOM 2155 C SER B 88 -6.010 2.025 -13.503 1.00 0.00

ATOM 2156 O SER B 88 -6.194 1.208 -14.394 1.00 0.00

ATOM 2157 CB SER B 88 -3.897 3.526 -14.066 1.00 0.00

ATOM 2158 HB1 SER B 88 -4.066 3.284 -15.121 1.00 0.00

ATOM 2159 HB2 SER B 88 -2.815 3.429 -13.935 1.00 0.00

ATOM 2160 OG SER B 88 -4.167 4.905 -13.824 1.00 0.00

ATOM 2161 HG SER B 88 -4.976 4.965 -13.281 1.00 0.00

ATOM 2162 N TRP B 89 -6.978 2.545 -12.725 1.00 0.00

ATOM 2163 HN TRP B 89 -6.796 3.263 -12.052 1.00 0.00

ATOM 2164 CA TRP B 89 -8.383 2.210 -12.940 1.00 0.00

ATOM 2165 HA TRP B 89 -8.448 1.189 -13.326 1.00 0.00

ATOM 2166 C TRP B 89 -8.933 3.192 -13.986 1.00 0.00

ATOM 2167 O TRP B 89 -8.641 4.385 -13.946 1.00 0.00

ATOM 2168 CB TRP B 89 -9.115 2.310 -11.587 1.00 0.00

ATOM 2169 HB1 TRP B 89 -8.881 3.247 -11.080 1.00 0.00

ATOM 2170 HB2 TRP B 89 -8.787 1.501 -10.932 1.00 0.00

ATOM 2171 CG TRP B 89 -10.613 2.178 -11.753 1.00 0.00

ATOM 2172 CD1 TRP B 89 -11.274 1.021 -12.165 1.00 0.00

ATOM 2173 HD1 TRP B 89 -10.802 0.092 -12.449 1.00 0.00

ATOM 2174 NE1 TRP B 89 -12.606 1.246 -12.158 1.00 0.00

ATOM 2175 HE1 TRP B 89 -13.289 0.600 -12.436 1.00 0.00

ATOM 2176 CE2 TRP B 89 -12.879 2.544 -11.767 1.00 0.00

ATOM 2177 CD2 TRP B 89 -11.641 3.164 -11.483 1.00 0.00

ATOM 2178 CE3 TRP B 89 -11.618 4.480 -11.089 1.00 0.00

ATOM 2179 HE3 TRP B 89 -10.682 4.985 -10.903 1.00 0.00

ATOM 2180 CZ3 TRP B 89 -12.819 5.177 -10.923 1.00 0.00

ATOM 2181 HZ3 TRP B 89 -12.803 6.215 -10.625 1.00 0.00

ATOM 2182 CH2 TRP B 89 -14.038 4.547 -11.189 1.00 0.00

ATOM 2183 HH2 TRP B 89 -14.990 5.046 -11.061 1.00 0.00

ATOM 2184 CZ2 TRP B 89 -14.056 3.219 -11.598 1.00 0.00

ATOM 2185 HZ2 TRP B 89 -14.991 2.721 -11.814 1.00 0.00

ATOM 2186 N ASN B 90 -9.679 2.596 -14.938 1.00 0.00

ATOM 2187 HN ASN B 90 -9.943 1.638 -14.820 1.00 0.00

ATOM 2188 CA ASN B 90 -10.431 3.362 -15.922 1.00 0.00

ATOM 2189 HA ASN B 90 -9.997 4.361 -16.009 1.00 0.00

ATOM 2190 C ASN B 90 -11.850 3.440 -15.400 1.00 0.00

ATOM 2191 O ASN B 90 -12.436 2.470 -14.931 1.00 0.00

ATOM 2192 CB ASN B 90 -10.509 2.707 -17.304 1.00 0.00

ATOM 2193 HB1 ASN B 90 -11.177 3.281 -17.948 1.00 0.00

ATOM 2194 HB2 ASN B 90 -10.924 1.702 -17.235 1.00 0.00

ATOM 2195 CG ASN B 90 -9.150 2.661 -17.981 1.00 0.00

ATOM 2196 OD1 ASN B 90 -8.545 3.673 -18.309 1.00 0.00

ATOM 2197 ND2 ASN B 90 -8.680 1.429 -18.111 1.00 0.00

ATOM 2198 HD21 ASN B 90 -7.720 1.331 -18.345 1.00 0.00

ATOM 2199 HD22 ASN B 90 -9.269 0.619 -17.978 1.00 0.00

ATOM 2200 N THR B 91 -12.325 4.680 -15.496 1.00 0.00

ATOM 2201 HN THR B 91 -11.787 5.325 -16.029 1.00 0.00

ATOM 2202 CA THR B 91 -13.616 4.991 -14.949 1.00 0.00

ATOM 2203 HA THR B 91 -13.663 4.546 -13.957 1.00 0.00

ATOM 2204 C THR B 91 -14.723 4.408 -15.824 1.00 0.00

ATOM 2205 O THR B 91 -14.967 4.867 -16.941 1.00 0.00

ATOM 2206 CB THR B 91 -13.632 6.509 -14.806 1.00 0.00

ATOM 2207 HB THR B 91 -13.402 6.992 -15.767 1.00 0.00

ATOM 2208 OG1 THR B 91 -12.617 6.794 -13.855 1.00 0.00

ATOM 2209 HG1 THR B 91 -11.775 6.422 -14.128 1.00 0.00

ATOM 2210 CG2 THR B 91 -14.971 7.069 -14.308 1.00 0.00

ATOM 2211 HG21 THR B 91 -15.610 6.297 -13.869 1.00 0.00

ATOM 2212 HG22 THR B 91 -14.817 7.856 -13.569 1.00 0.00

ATOM 2213 HG23 THR B 91 -15.519 7.522 -15.137 1.00 0.00

ATOM 2214 N GLY B 92 -15.382 3.426 -15.172 1.00 0.00

ATOM 2215 HN GLY B 92 -14.940 2.991 -14.374 1.00 0.00

ATOM 2216 CA GLY B 92 -16.473 2.690 -15.779 1.00 0.00

ATOM 2217 HA1 GLY B 92 -16.505 2.870 -16.857 1.00 0.00

ATOM 2218 HA2 GLY B 92 -17.413 3.002 -15.320 1.00 0.00

ATOM 2219 C GLY B 92 -16.217 1.213 -15.514 1.00 0.00

ATOM 2220 O GLY B 92 -17.134 0.434 -15.286 1.00 0.00

ATOM 2221 N GLU B 93 -14.927 0.859 -15.566 1.00 0.00

ATOM 2222 HN GLU B 93 -14.204 1.548 -15.508 1.00 0.00

ATOM 2223 CA GLU B 93 -14.580 -0.538 -15.408 1.00 0.00

ATOM 2224 HA GLU B 93 -15.091 -1.094 -16.196 1.00 0.00

ATOM 2225 C GLU B 93 -15.018 -1.058 -14.033 1.00 0.00

ATOM 2226 O GLU B 93 -14.829 -0.431 -12.998 1.00 0.00

ATOM 2227 CB GLU B 93 -13.064 -0.729 -15.519 1.00 0.00

ATOM 2228 HB1 GLU B 93 -12.813 -1.722 -15.140 1.00 0.00

ATOM 2229 HB2 GLU B 93 -12.545 -0.001 -14.894 1.00 0.00

ATOM 2230 CG GLU B 93 -12.519 -0.653 -16.946 1.00 0.00

ATOM 2231 HG1 GLU B 93 -12.663 0.345 -17.364 1.00 0.00

ATOM 2232 HG2 GLU B 93 -13.069 -1.351 -17.581 1.00 0.00

ATOM 2233 CD GLU B 93 -11.019 -1.030 -16.998 1.00 0.00

ATOM 2234 OE1 GLU B 93 -10.395 -1.388 -15.994 1.00 0.00

ATOM 2235 OE2 GLU B 93 -10.462 -0.960 -18.080 1.00 0.00

ATOM 2236 N GLU B 94 -15.525 -2.295 -14.091 1.00 0.00

ATOM 2237 HN GLU B 94 -15.713 -2.725 -14.976 1.00 0.00

ATOM 2238 CA GLU B 94 -15.627 -3.045 -12.845 1.00 0.00

ATOM 2239 HA GLU B 94 -16.073 -2.415 -12.076 1.00 0.00

ATOM 2240 C GLU B 94 -14.207 -3.437 -12.417 1.00 0.00

ATOM 2241 O GLU B 94 -13.319 -3.440 -13.256 1.00 0.00

ATOM 2242 CB GLU B 94 -16.496 -4.271 -13.103 1.00 0.00

ATOM 2243 HB1 GLU B 94 -16.436 -4.923 -12.230 1.00 0.00

ATOM 2244 HB2 GLU B 94 -16.136 -4.845 -13.959 1.00 0.00

ATOM 2245 CG GLU B 94 -17.956 -3.873 -13.335 1.00 0.00

ATOM 2246 HG1 GLU B 94 -18.092 -3.352 -14.283 1.00 0.00

ATOM 2247 HG2 GLU B 94 -18.275 -3.193 -12.545 1.00 0.00

ATOM 2248 CD GLU B 94 -18.822 -5.122 -13.266 1.00 0.00

ATOM 2249 OE1 GLU B 94 -19.239 -5.617 -14.314 1.00 0.00

ATOM 2250 OE2 GLU B 94 -19.039 -5.581 -12.151 1.00 0.00

ATOM 2251 N VAL B 95 -14.035 -3.755 -11.119 1.00 0.00

ATOM 2252 HN VAL B 95 -14.805 -3.646 -10.479 1.00 0.00

ATOM 2253 CA VAL B 95 -12.760 -4.293 -10.633 1.00 0.00

ATOM 2254 HA VAL B 95 -12.228 -4.722 -11.479 1.00 0.00

ATOM 2255 C VAL B 95 -13.076 -5.394 -9.615 1.00 0.00

ATOM 2256 O VAL B 95 -14.202 -5.427 -9.120 1.00 0.00

ATOM 2257 CB VAL B 95 -11.907 -3.189 -9.966 1.00 0.00

ATOM 2258 HB VAL B 95 -11.073 -3.684 -9.469 1.00 0.00

ATOM 2259 CG1 VAL B 95 -11.247 -2.254 -10.995 1.00 0.00

ATOM 2260 HG11 VAL B 95 -11.475 -2.535 -12.021 1.00 0.00

ATOM 2261 HG12 VAL B 95 -11.571 -1.223 -10.882 1.00 0.00

ATOM 2262 HG13 VAL B 95 -10.162 -2.264 -10.895 1.00 0.00

ATOM 2263 CG2 VAL B 95 -12.675 -2.408 -8.880 1.00 0.00

ATOM 2264 HG21 VAL B 95 -13.753 -2.563 -8.938 1.00 0.00

ATOM 2265 HG22 VAL B 95 -12.383 -2.722 -7.881 1.00 0.00

ATOM 2266 HG23 VAL B 95 -12.507 -1.332 -8.951 1.00 0.00

ATOM 2267 N GLN B 96 -12.045 -6.194 -9.282 1.00 0.00

ATOM 2268 HN GLN B 96 -11.143 -6.067 -9.695 1.00 0.00

ATOM 2269 CA GLN B 96 -12.195 -7.210 -8.243 1.00 0.00

ATOM 2270 HA GLN B 96 -13.227 -7.203 -7.906 1.00 0.00

ATOM 2271 C GLN B 96 -11.307 -6.885 -7.007 1.00 0.00

ATOM 2272 O GLN B 96 -10.124 -6.522 -7.085 1.00 0.00

ATOM 2273 CB GLN B 96 -11.914 -8.595 -8.834 1.00 0.00

ATOM 2274 HB1 GLN B 96 -11.823 -9.279 -7.999 1.00 0.00

ATOM 2275 HB2 GLN B 96 -10.974 -8.607 -9.384 1.00 0.00

ATOM 2276 CG GLN B 96 -13.040 -9.131 -9.730 1.00 0.00

ATOM 2277 HG1 GLN B 96 -13.233 -8.467 -10.572 1.00 0.00

ATOM 2278 HG2 GLN B 96 -13.958 -9.202 -9.153 1.00 0.00

ATOM 2279 CD GLN B 96 -12.706 -10.541 -10.246 1.00 0.00

ATOM 2280 OE1 GLN B 96 -11.756 -11.183 -9.806 1.00 0.00

ATOM 2281 NE2 GLN B 96 -13.541 -10.993 -11.179 1.00 0.00

ATOM 2282 HE21 GLN B 96 -13.435 -11.928 -11.516 1.00 0.00

ATOM 2283 HE22 GLN B 96 -14.300 -10.453 -11.563 1.00 0.00

ATOM 2284 N VAL B 97 -11.945 -6.973 -5.824 1.00 0.00

ATOM 2285 HN VAL B 97 -12.902 -7.270 -5.861 1.00 0.00

ATOM 2286 CA VAL B 97 -11.237 -6.824 -4.547 1.00 0.00

ATOM 2287 HA VAL B 97 -10.327 -6.252 -4.717 1.00 0.00

ATOM 2288 C VAL B 97 -10.900 -8.243 -4.049 1.00 0.00

ATOM 2289 O VAL B 97 -11.802 -9.002 -3.719 1.00 0.00

ATOM 2290 CB VAL B 97 -12.091 -6.099 -3.463 1.00 0.00

ATOM 2291 HB VAL B 97 -12.960 -6.724 -3.244 1.00 0.00

ATOM 2292 CG1 VAL B 97 -11.309 -5.949 -2.129 1.00 0.00

ATOM 2293 HG11 VAL B 97 -10.285 -6.319 -2.206 1.00 0.00

ATOM 2294 HG12 VAL B 97 -11.239 -4.912 -1.800 1.00 0.00

ATOM 2295 HG13 VAL B 97 -11.793 -6.514 -1.332 1.00 0.00

ATOM 2296 CG2 VAL B 97 -12.693 -4.754 -3.945 1.00 0.00

ATOM 2297 HG21 VAL B 97 -12.380 -4.512 -4.960 1.00 0.00

ATOM 2298 HG22 VAL B 97 -13.783 -4.815 -3.959 1.00 0.00

ATOM 2299 HG23 VAL B 97 -12.427 -3.915 -3.304 1.00 0.00

ATOM 2300 N ILE B 98 -9.596 -8.592 -3.960 1.00 0.00

ATOM 2301 HN ILE B 98 -8.873 -7.937 -4.176 1.00 0.00

ATOM 2302 CA ILE B 98 -9.237 -9.938 -3.493 1.00 0.00

ATOM 2303 HA ILE B 98 -10.012 -10.618 -3.853 1.00 0.00

ATOM 2304 C ILE B 98 -9.223 -9.966 -1.941 1.00 0.00

ATOM 2305 O ILE B 98 -8.193 -9.922 -1.267 1.00 0.00

ATOM 2306 CB ILE B 98 -7.888 -10.413 -4.078 1.00 0.00

ATOM 2307 HB ILE B 98 -7.101 -9.832 -3.602 1.00 0.00

ATOM 2308 CG2 ILE B 98 -7.667 -11.908 -3.759 1.00 0.00

ATOM 2309 HG21 ILE B 98 -8.533 -12.350 -3.261 1.00 0.00

ATOM 2310 HG22 ILE B 98 -7.494 -12.505 -4.656 1.00 0.00

ATOM 2311 HG23 ILE B 98 -6.814 -12.036 -3.097 1.00 0.00

ATOM 2312 CG1 ILE B 98 -7.739 -10.169 -5.593 1.00 0.00

ATOM 2313 HG11 ILE B 98 -8.002 -9.144 -5.863 1.00 0.00

ATOM 2314 HG12 ILE B 98 -8.441 -10.804 -6.129 1.00 0.00

ATOM 2315 CD1 ILE B 98 -6.323 -10.467 -6.102 1.00 0.00

ATOM 2316 HD11 ILE B 98 -5.707 -10.899 -5.317 1.00 0.00

ATOM 2317 HD12 ILE B 98 -6.349 -11.195 -6.907 1.00 0.00

ATOM 2318 HD13 ILE B 98 -5.831 -9.572 -6.484 1.00 0.00

ATOM 2319 N ALA B 99 -10.468 -10.021 -1.429 1.00 0.00

ATOM 2320 HN ALA B 99 -11.198 -10.210 -2.101 1.00 0.00

ATOM 2321 CA ALA B 99 -10.732 -9.822 -0.009 1.00 0.00

ATOM 2322 HA ALA B 99 -10.131 -8.966 0.297 1.00 0.00

ATOM 2323 C ALA B 99 -10.319 -11.058 0.835 1.00 0.00

ATOM 2324 O ALA B 99 -10.845 -12.159 0.672 1.00 0.00

ATOM 2325 CB ALA B 99 -12.214 -9.453 0.161 1.00 0.00

ATOM 2326 HB1 ALA B 99 -12.735 -9.400 -0.799 1.00 0.00

ATOM 2327 HB2 ALA B 99 -12.749 -10.167 0.785 1.00 0.00

ATOM 2328 HB3 ALA B 99 -12.318 -8.471 0.624 1.00 0.00

ATOM 2329 N VAL B 100 -9.374 -10.788 1.765 1.00 0.00

ATOM 2330 HN VAL B 100 -8.925 -9.896 1.675 1.00 0.00

ATOM 2331 CA VAL B 100 -8.781 -11.833 2.601 1.00 0.00

ATOM 2332 HA VAL B 100 -9.199 -12.792 2.287 1.00 0.00

ATOM 2333 C VAL B 100 -9.144 -11.626 4.092 1.00 0.00

ATOM 2334 O VAL B 100 -8.289 -11.452 4.972 1.00 0.00

ATOM 2335 CB VAL B 100 -7.259 -11.842 2.344 1.00 0.00

ATOM 2336 HB VAL B 100 -6.874 -10.859 2.612 1.00 0.00

ATOM 2337 CG1 VAL B 100 -6.504 -12.851 3.232 1.00 0.00

ATOM 2338 HG11 VAL B 100 -7.171 -13.394 3.896 1.00 0.00

ATOM 2339 HG12 VAL B 100 -5.964 -13.590 2.646 1.00 0.00

ATOM 2340 HG13 VAL B 100 -5.771 -12.324 3.846 1.00 0.00

ATOM 2341 CG2 VAL B 100 -6.940 -12.035 0.841 1.00 0.00

ATOM 2342 HG21 VAL B 100 -7.793 -12.446 0.297 1.00 0.00

ATOM 2343 HG22 VAL B 100 -6.684 -11.087 0.356 1.00 0.00

ATOM 2344 HG23 VAL B 100 -6.092 -12.708 0.685 1.00 0.00

ATOM 2345 N GLU B 101 -10.479 -11.698 4.284 1.00 0.00

ATOM 2346 HN GLU B 101 -11.085 -11.928 3.512 1.00 0.00

ATOM 2347 CA GLU B 101 -11.052 -11.488 5.620 1.00 0.00

ATOM 2348 HA GLU B 101 -10.865 -10.440 5.857 1.00 0.00

ATOM 2349 C GLU B 101 -10.367 -12.422 6.677 1.00 0.00

ATOM 2350 O GLU B 101 -9.944 -13.551 6.383 1.00 0.00

ATOM 2351 CB GLU B 101 -12.581 -11.717 5.565 1.00 0.00

ATOM 2352 HB1 GLU B 101 -13.004 -11.504 6.549 1.00 0.00

ATOM 2353 HB2 GLU B 101 -12.774 -12.761 5.341 1.00 0.00

ATOM 2354 CG GLU B 101 -13.298 -10.834 4.524 1.00 0.00

ATOM 2355 HG1 GLU B 101 -12.848 -10.947 3.538 1.00 0.00

ATOM 2356 HG2 GLU B 101 -13.186 -9.789 4.819 1.00 0.00

ATOM 2357 CD GLU B 101 -14.796 -11.150 4.376 1.00 0.00

ATOM 2358 OE1 GLU B 101 -15.127 -12.134 3.702 1.00 0.00

ATOM 2359 OE2 GLU B 101 -15.636 -10.416 4.896 1.00 0.00

ATOM 2360 N PRO B 102 -10.254 -11.938 7.950 1.00 0.00

ATOM 2361 CA PRO B 102 -9.704 -12.773 9.015 1.00 0.00

ATOM 2362 HA PRO B 102 -8.637 -12.922 8.837 1.00 0.00

ATOM 2363 CD PRO B 102 -10.676 -10.611 8.415 1.00 0.00

ATOM 2364 HD1 PRO B 102 -11.755 -10.509 8.318 1.00 0.00

ATOM 2365 HD2 PRO B 102 -10.215 -9.825 7.816 1.00 0.00

ATOM 2366 C PRO B 102 -10.419 -14.136 9.033 1.00 0.00

ATOM 2367 O PRO B 102 -11.636 -14.190 8.894 1.00 0.00

ATOM 2368 CB PRO B 102 -9.952 -11.984 10.311 1.00 0.00

ATOM 2369 HB1 PRO B 102 -9.087 -12.045 10.973 1.00 0.00

ATOM 2370 HB2 PRO B 102 -10.821 -12.385 10.840 1.00 0.00

ATOM 2371 CG PRO B 102 -10.241 -10.533 9.895 1.00 0.00

ATOM 2372 HG1 PRO B 102 -9.348 -9.913 10.020 1.00 0.00

ATOM 2373 HG2 PRO B 102 -10.994 -10.058 10.528 1.00 0.00

ATOM 2374 N GLY B 103 -9.625 -15.223 9.093 1.00 0.00

ATOM 2375 HN GLY B 103 -8.637 -15.114 9.226 1.00 0.00

ATOM 2376 CA GLY B 103 -10.286 -16.523 9.257 1.00 0.00

ATOM 2377 HA1 GLY B 103 -10.960 -16.448 10.113 1.00 0.00

ATOM 2378 HA2 GLY B 103 -9.518 -17.263 9.493 1.00 0.00

ATOM 2379 C GLY B 103 -11.126 -17.018 8.055 1.00 0.00

ATOM 2380 O GLY B 103 -11.763 -18.061 8.150 1.00 0.00

ATOM 2381 N LYS B 104 -11.120 -16.283 6.930 1.00 0.00

ATOM 2382 HN LYS B 104 -10.862 -15.318 6.954 1.00 0.00

ATOM 2383 CA LYS B 104 -11.795 -16.763 5.724 1.00 0.00

ATOM 2384 HA LYS B 104 -12.375 -17.660 5.937 1.00 0.00

ATOM 2385 C LYS B 104 -10.729 -17.113 4.693 1.00 0.00

ATOM 2386 O LYS B 104 -9.590 -16.641 4.763 1.00 0.00

ATOM 2387 CB LYS B 104 -12.740 -15.679 5.185 1.00 0.00

ATOM 2388 HB1 LYS B 104 -13.179 -16.010 4.246 1.00 0.00

ATOM 2389 HB2 LYS B 104 -12.162 -14.787 4.945 1.00 0.00

ATOM 2390 CG LYS B 104 -13.859 -15.316 6.176 1.00 0.00

ATOM 2391 HG1 LYS B 104 -14.407 -14.444 5.815 1.00 0.00

ATOM 2392 HG2 LYS B 104 -13.408 -15.015 7.123 1.00 0.00

ATOM 2393 CD LYS B 104 -14.863 -16.449 6.429 1.00 0.00

ATOM 2394 HD1 LYS B 104 -15.533 -16.127 7.226 1.00 0.00

ATOM 2395 HD2 LYS B 104 -14.340 -17.330 6.802 1.00 0.00

ATOM 2396 CE LYS B 104 -15.689 -16.789 5.182 1.00 0.00

ATOM 2397 HE1 LYS B 104 -15.046 -17.181 4.390 1.00 0.00

ATOM 2398 HE2 LYS B 104 -16.158 -15.881 4.795 1.00 0.00

ATOM 2399 NZ LYS B 104 -16.744 -17.770 5.442 1.00 0.00

ATOM 2400 HZ1 LYS B 104 -16.844 -17.907 6.467 1.00 0.00

ATOM 2401 HZ2 LYS B 104 -17.654 -17.430 5.063 1.00 0.00

ATOM 2402 HZ3 LYS B 104 -16.496 -18.674 4.990 1.00 0.00

ATOM 2403 N ASN B 105 -11.178 -17.930 3.725 1.00 0.00

ATOM 2404 HN ASN B 105 -12.047 -18.417 3.815 1.00 0.00

ATOM 2405 CA ASN B 105 -10.451 -18.012 2.460 1.00 0.00

ATOM 2406 HA ASN B 105 -9.432 -18.317 2.686 1.00 0.00

ATOM 2407 C ASN B 105 -10.528 -16.622 1.827 1.00 0.00

ATOM 2408 O ASN B 105 -11.466 -15.891 2.139 1.00 0.00

ATOM 2409 CB ASN B 105 -11.102 -19.008 1.493 1.00 0.00

ATOM 2410 HB1 ASN B 105 -10.634 -18.978 0.507 1.00 0.00

ATOM 2411 HB2 ASN B 105 -12.154 -18.770 1.341 1.00 0.00

ATOM 2412 CG ASN B 105 -10.979 -20.411 2.060 1.00 0.00

ATOM 2413 OD1 ASN B 105 -11.622 -20.747 3.046 1.00 0.00

ATOM 2414 ND2 ASN B 105 -10.082 -21.203 1.476 1.00 0.00

ATOM 2415 HD21 ASN B 105 -9.804 -22.066 1.896 1.00 0.00

ATOM 2416 HD22 ASN B 105 -9.681 -20.895 0.609 1.00 0.00

ATOM 2417 N PRO B 106 -9.484 -16.296 1.019 1.00 0.00

ATOM 2418 CA PRO B 106 -9.567 -15.296 -0.039 1.00 0.00

ATOM 2419 HA PRO B 106 -9.730 -14.324 0.428 1.00 0.00

ATOM 2420 CD PRO B 106 -8.162 -16.910 1.113 1.00 0.00

ATOM 2421 HD1 PRO B 106 -8.178 -17.894 0.641 1.00 0.00

ATOM 2422 HD2 PRO B 106 -7.845 -17.010 2.151 1.00 0.00

ATOM 2423 C PRO B 106 -10.746 -15.592 -0.982 1.00 0.00

ATOM 2424 O PRO B 106 -11.271 -16.698 -1.016 1.00 0.00

ATOM 2425 CB PRO B 106 -8.191 -15.352 -0.721 1.00 0.00

ATOM 2426 HB1 PRO B 106 -7.858 -14.372 -1.069 1.00 0.00

ATOM 2427 HB2 PRO B 106 -8.228 -16.028 -1.582 1.00 0.00

ATOM 2428 CG PRO B 106 -7.262 -15.942 0.349 1.00 0.00

ATOM 2429 HG1 PRO B 106 -6.922 -15.146 1.017 1.00 0.00

ATOM 2430 HG2 PRO B 106 -6.378 -16.433 -0.063 1.00 0.00

ATOM 2431 N LYS B 107 -11.189 -14.481 -1.580 1.00 0.00

ATOM 2432 HN LYS B 107 -10.585 -13.680 -1.655 1.00 0.00

ATOM 2433 CA LYS B 107 -12.509 -14.384 -2.163 1.00 0.00

ATOM 2434 HA LYS B 107 -12.729 -15.306 -2.702 1.00 0.00

ATOM 2435 C LYS B 107 -12.415 -13.180 -3.099 1.00 0.00

ATOM 2436 O LYS B 107 -12.108 -12.094 -2.621 1.00 0.00

ATOM 2437 CB LYS B 107 -13.479 -14.127 -0.988 1.00 0.00

ATOM 2438 HB1 LYS B 107 -13.120 -13.259 -0.436 1.00 0.00

ATOM 2439 HB2 LYS B 107 -13.434 -14.966 -0.291 1.00 0.00

ATOM 2440 CG LYS B 107 -14.948 -13.911 -1.367 1.00 0.00

ATOM 2441 HG1 LYS B 107 -15.337 -14.825 -1.822 1.00 0.00

ATOM 2442 HG2 LYS B 107 -15.031 -13.129 -2.125 1.00 0.00

ATOM 2443 CD LYS B 107 -15.804 -13.525 -0.148 1.00 0.00

ATOM 2444 HD1 LYS B 107 -15.667 -14.269 0.638 1.00 0.00

ATOM 2445 HD2 LYS B 107 -16.858 -13.566 -0.433 1.00 0.00

ATOM 2446 CE LYS B 107 -15.453 -12.120 0.381 1.00 0.00

ATOM 2447 HE1 LYS B 107 -15.475 -11.395 -0.433 1.00 0.00

ATOM 2448 HE2 LYS B 107 -14.438 -12.100 0.782 1.00 0.00

ATOM 2449 NZ LYS B 107 -16.354 -11.641 1.427 1.00 0.00

ATOM 2450 HZ1 LYS B 107 -17.061 -12.374 1.619 1.00 0.00

ATOM 2451 HZ2 LYS B 107 -15.817 -11.395 2.290 1.00 0.00

ATOM 2452 HZ3 LYS B 107 -16.846 -10.791 1.101 1.00 0.00

ATOM 2453 N ASN B 108 -12.625 -13.413 -4.396 1.00 0.00

ATOM 2454 HN ASN B 108 -12.991 -14.279 -4.737 1.00 0.00

ATOM 2455 CA ASN B 108 -12.667 -12.294 -5.341 1.00 0.00

ATOM 2456 HA ASN B 108 -12.077 -11.455 -4.961 1.00 0.00

ATOM 2457 C ASN B 108 -14.124 -11.866 -5.367 1.00 0.00

ATOM 2458 O ASN B 108 -15.002 -12.696 -5.153 1.00 0.00

ATOM 2459 CB ASN B 108 -12.167 -12.721 -6.730 1.00 0.00

ATOM 2460 HB1 ASN B 108 -12.818 -12.322 -7.507 1.00 0.00

ATOM 2461 HB2 ASN B 108 -12.152 -13.804 -6.852 1.00 0.00

ATOM 2462 CG ASN B 108 -10.780 -12.120 -6.962 1.00 0.00

ATOM 2463 OD1 ASN B 108 -10.614 -10.905 -6.967 1.00 0.00

ATOM 2464 ND2 ASN B 108 -9.797 -13.003 -7.154 1.00 0.00

ATOM 2465 HD21 ASN B 108 -8.916 -12.704 -7.529 1.00 0.00

ATOM 2466 HD22 ASN B 108 -9.961 -13.974 -6.963 1.00 0.00

ATOM 2467 N VAL B 109 -14.325 -10.543 -5.501 1.00 0.00

ATOM 2468 HN VAL B 109 -13.556 -9.938 -5.712 1.00 0.00

ATOM 2469 CA VAL B 109 -15.657 -9.950 -5.573 1.00 0.00

ATOM 2470 HA VAL B 109 -16.350 -10.700 -5.969 1.00 0.00

ATOM 2471 C VAL B 109 -15.544 -8.761 -6.550 1.00 0.00

ATOM 2472 O VAL B 109 -14.746 -7.853 -6.328 1.00 0.00

ATOM 2473 CB VAL B 109 -16.137 -9.415 -4.190 1.00 0.00

ATOM 2474 HB VAL B 109 -15.474 -8.596 -3.902 1.00 0.00

ATOM 2475 CG1 VAL B 109 -17.561 -8.806 -4.271 1.00 0.00

ATOM 2476 HG11 VAL B 109 -17.928 -8.711 -5.295 1.00 0.00

ATOM 2477 HG12 VAL B 109 -18.298 -9.409 -3.740 1.00 0.00

ATOM 2478 HG13 VAL B 109 -17.605 -7.805 -3.838 1.00 0.00

ATOM 2479 CG2 VAL B 109 -16.018 -10.438 -3.034 1.00 0.00

ATOM 2480 HG21 VAL B 109 -15.911 -11.460 -3.401 1.00 0.00

ATOM 2481 HG22 VAL B 109 -15.149 -10.195 -2.415 1.00 0.00

ATOM 2482 HG23 VAL B 109 -16.905 -10.433 -2.394 1.00 0.00

ATOM 2483 N GLN B 110 -16.387 -8.821 -7.576 1.00 0.00

ATOM 2484 HN GLN B 110 -17.035 -9.581 -7.533 1.00 0.00

ATOM 2485 CA GLN B 110 -16.419 -7.837 -8.644 1.00 0.00

ATOM 2486 HA GLN B 110 -15.420 -7.425 -8.767 1.00 0.00

ATOM 2487 C GLN B 110 -17.407 -6.702 -8.306 1.00 0.00

ATOM 2488 O GLN B 110 -18.462 -6.940 -7.731 1.00 0.00

ATOM 2489 CB GLN B 110 -16.802 -8.625 -9.892 1.00 0.00

ATOM 2490 HB1 GLN B 110 -17.831 -8.982 -9.839 1.00 0.00

ATOM 2491 HB2 GLN B 110 -16.165 -9.507 -9.938 1.00 0.00

ATOM 2492 CG GLN B 110 -16.588 -7.833 -11.172 1.00 0.00

ATOM 2493 HG1 GLN B 110 -15.614 -7.346 -11.161 1.00 0.00

ATOM 2494 HG2 GLN B 110 -17.349 -7.062 -11.257 1.00 0.00

ATOM 2495 CD GLN B 110 -16.646 -8.756 -12.392 1.00 0.00

ATOM 2496 OE1 GLN B 110 -16.256 -9.919 -12.356 1.00 0.00

ATOM 2497 NE2 GLN B 110 -17.093 -8.136 -13.484 1.00 0.00

ATOM 2498 HE21 GLN B 110 -16.995 -8.569 -14.372 1.00 0.00

ATOM 2499 HE22 GLN B 110 -17.545 -7.247 -13.350 1.00 0.00

ATOM 2500 N THR B 111 -17.028 -5.458 -8.663 1.00 0.00

ATOM 2501 HN THR B 111 -16.215 -5.264 -9.217 1.00 0.00

ATOM 2502 CA THR B 111 -17.768 -4.290 -8.191 1.00 0.00

ATOM 2503 HA THR B 111 -18.833 -4.529 -8.194 1.00 0.00

ATOM 2504 C THR B 111 -17.493 -3.125 -9.170 1.00 0.00

ATOM 2505 O THR B 111 -16.365 -2.983 -9.630 1.00 0.00

ATOM 2506 CB THR B 111 -17.267 -3.996 -6.750 1.00 0.00

ATOM 2507 HB THR B 111 -17.471 -4.891 -6.136 1.00 0.00

ATOM 2508 OG1 THR B 111 -17.905 -2.848 -6.208 1.00 0.00

ATOM 2509 HG1 THR B 111 -17.947 -2.890 -5.254 1.00 0.00

ATOM 2510 CG2 THR B 111 -15.767 -3.685 -6.690 1.00 0.00

ATOM 2511 HG21 THR B 111 -15.224 -4.040 -7.563 1.00 0.00

ATOM 2512 HG22 THR B 111 -15.630 -2.603 -6.673 1.00 0.00

ATOM 2513 HG23 THR B 111 -15.313 -4.108 -5.790 1.00 0.00

ATOM 2514 N THR B 112 -18.529 -2.311 -9.438 1.00 0.00

ATOM 2515 HN THR B 112 -19.428 -2.612 -9.135 1.00 0.00

ATOM 2516 CA THR B 112 -18.400 -1.080 -10.204 1.00 0.00

ATOM 2517 HA THR B 112 -17.591 -1.227 -10.918 1.00 0.00

ATOM 2518 C THR B 112 -18.134 0.117 -9.241 1.00 0.00

ATOM 2519 O THR B 112 -19.047 0.575 -8.552 1.00 0.00

ATOM 2520 CB THR B 112 -19.708 -0.835 -10.994 1.00 0.00

ATOM 2521 HB THR B 112 -20.477 -0.466 -10.308 1.00 0.00

ATOM 2522 OG1 THR B 112 -20.139 -2.087 -11.498 1.00 0.00

ATOM 2523 HG1 THR B 112 -20.863 -2.024 -12.121 1.00 0.00

ATOM 2524 CG2 THR B 112 -19.496 0.207 -12.105 1.00 0.00

ATOM 2525 HG21 THR B 112 -18.525 0.696 -12.012 1.00 0.00

ATOM 2526 HG22 THR B 112 -19.522 -0.233 -13.102 1.00 0.00

ATOM 2527 HG23 THR B 112 -20.249 0.997 -12.058 1.00 0.00

ATOM 2528 N PRO B 113 -16.888 0.655 -9.222 1.00 0.00

ATOM 2529 CA PRO B 113 -16.597 1.846 -8.435 1.00 0.00

ATOM 2530 HA PRO B 113 -16.970 1.698 -7.420 1.00 0.00

ATOM 2531 CD PRO B 113 -15.715 0.160 -9.934 1.00 0.00

ATOM 2532 HD1 PRO B 113 -15.692 0.577 -10.939 1.00 0.00

ATOM 2533 HD2 PRO B 113 -15.684 -0.915 -10.022 1.00 0.00

ATOM 2534 C PRO B 113 -17.239 3.110 -9.052 1.00 0.00

ATOM 2535 O PRO B 113 -17.135 3.378 -10.246 1.00 0.00

ATOM 2536 CB PRO B 113 -15.058 1.959 -8.441 1.00 0.00

ATOM 2537 HB1 PRO B 113 -14.657 2.080 -7.437 1.00 0.00

ATOM 2538 HB2 PRO B 113 -14.722 2.817 -9.022 1.00 0.00

ATOM 2539 CG PRO B 113 -14.552 0.663 -9.084 1.00 0.00

ATOM 2540 HG1 PRO B 113 -14.346 -0.067 -8.301 1.00 0.00

ATOM 2541 HG2 PRO B 113 -13.639 0.792 -9.664 1.00 0.00

ATOM 2542 N GLY B 114 -17.881 3.900 -8.174 1.00 0.00

ATOM 2543 HN GLY B 114 -17.861 3.569 -7.231 1.00 0.00

ATOM 2544 CA GLY B 114 -18.149 5.288 -8.534 1.00 0.00

ATOM 2545 HA1 GLY B 114 -18.956 5.648 -7.897 1.00 0.00

ATOM 2546 HA2 GLY B 114 -18.448 5.337 -9.580 1.00 0.00

ATOM 2547 C GLY B 114 -16.872 6.101 -8.312 1.00 0.00

ATOM 2548 O GLY B 114 -15.847 5.551 -7.916 1.00 0.00

ATOM 2549 N THR B 115 -17.005 7.424 -8.534 1.00 0.00

ATOM 2550 HN THR B 115 -17.885 7.841 -8.729 1.00 0.00

ATOM 2551 CA THR B 115 -15.895 8.343 -8.338 1.00 0.00

ATOM 2552 HA THR B 115 -15.047 7.784 -7.934 1.00 0.00

ATOM 2553 C THR B 115 -16.285 9.417 -7.315 1.00 0.00

ATOM 2554 O THR B 115 -17.429 9.860 -7.217 1.00 0.00

ATOM 2555 CB THR B 115 -15.519 8.984 -9.680 1.00 0.00

ATOM 2556 HB THR B 115 -14.693 9.670 -9.447 1.00 0.00

ATOM 2557 OG1 THR B 115 -16.640 9.631 -10.285 1.00 0.00

ATOM 2558 HG1 THR B 115 -16.354 10.467 -10.696 1.00 0.00

ATOM 2559 CG2 THR B 115 -15.033 7.959 -10.704 1.00 0.00

ATOM 2560 HG21 THR B 115 -15.013 6.964 -10.274 1.00 0.00

ATOM 2561 HG22 THR B 115 -15.687 7.928 -11.577 1.00 0.00

ATOM 2562 HG23 THR B 115 -14.026 8.184 -11.063 1.00 0.00

ATOM 2563 N PHE B 116 -15.271 9.761 -6.505 1.00 0.00

ATOM 2564 HN PHE B 116 -14.355 9.413 -6.734 1.00 0.00

ATOM 2565 CA PHE B 116 -15.383 10.753 -5.450 1.00 0.00

ATOM 2566 HA PHE B 116 -16.381 11.192 -5.453 1.00 0.00

ATOM 2567 C PHE B 116 -14.348 11.837 -5.796 1.00 0.00

ATOM 2568 O PHE B 116 -13.244 11.528 -6.227 1.00 0.00

ATOM 2569 CB PHE B 116 -15.087 10.065 -4.109 1.00 0.00

ATOM 2570 HB1 PHE B 116 -14.629 10.789 -3.438 1.00 0.00

ATOM 2571 HB2 PHE B 116 -14.345 9.277 -4.242 1.00 0.00

ATOM 2572 CG PHE B 116 -16.299 9.506 -3.396 1.00 0.00

ATOM 2573 CD1 PHE B 116 -17.372 8.910 -4.058 1.00 0.00

ATOM 2574 HD1 PHE B 116 -17.389 8.793 -5.124 1.00 0.00

ATOM 2575 CE1 PHE B 116 -18.463 8.416 -3.359 1.00 0.00

ATOM 2576 HE1 PHE B 116 -19.284 7.958 -3.888 1.00 0.00

ATOM 2577 CZ PHE B 116 -18.500 8.502 -1.980 1.00 0.00

ATOM 2578 HZ PHE B 116 -19.343 8.098 -1.442 1.00 0.00

ATOM 2579 CE2 PHE B 116 -17.443 9.098 -1.311 1.00 0.00

ATOM 2580 HE2 PHE B 116 -17.455 9.208 -0.240 1.00 0.00

ATOM 2581 CD2 PHE B 116 -16.353 9.584 -2.011 1.00 0.00

ATOM 2582 HD2 PHE B 116 -15.537 10.031 -1.463 1.00 0.00

ATOM 2583 N LYS B 117 -14.796 13.097 -5.661 1.00 0.00

ATOM 2584 HN LYS B 117 -15.602 13.259 -5.093 1.00 0.00

ATOM 2585 CA LYS B 117 -13.942 14.219 -6.043 1.00 0.00

ATOM 2586 HA LYS B 117 -13.188 13.861 -6.744 1.00 0.00

ATOM 2587 C LYS B 117 -13.210 14.701 -4.798 1.00 0.00

ATOM 2588 O LYS B 117 -13.767 14.792 -3.712 1.00 0.00

ATOM 2589 CB LYS B 117 -14.687 15.377 -6.731 1.00 0.00

ATOM 2590 HB1 LYS B 117 -14.513 15.308 -7.807 1.00 0.00

ATOM 2591 HB2 LYS B 117 -14.228 16.318 -6.423 1.00 0.00

ATOM 2592 CG LYS B 117 -16.194 15.459 -6.470 1.00 0.00

ATOM 2593 HG1 LYS B 117 -16.506 16.503 -6.525 1.00 0.00

ATOM 2594 HG2 LYS B 117 -16.417 15.145 -5.455 1.00 0.00

ATOM 2595 CD LYS B 117 -16.979 14.667 -7.519 1.00 0.00

ATOM 2596 HD1 LYS B 117 -16.771 13.599 -7.424 1.00 0.00

ATOM 2597 HD2 LYS B 117 -16.627 14.966 -8.510 1.00 0.00

ATOM 2598 CE LYS B 117 -18.479 14.918 -7.437 1.00 0.00

ATOM 2599 HE1 LYS B 117 -18.690 15.991 -7.432 1.00 0.00

ATOM 2600 HE2 LYS B 117 -18.905 14.478 -6.530 1.00 0.00

ATOM 2601 NZ LYS B 117 -19.128 14.324 -8.599 1.00 0.00

ATOM 2602 HZ1 LYS B 117 -18.434 13.753 -9.135 1.00 0.00

ATOM 2603 HZ2 LYS B 117 -19.900 13.718 -8.257 1.00 0.00

ATOM 2604 HZ3 LYS B 117 -19.490 15.079 -9.215 1.00 0.00

ATOM 2605 N THR B 118 -11.927 14.977 -5.059 1.00 0.00

ATOM 2606 HN THR B 118 -11.519 14.902 -5.969 1.00 0.00

ATOM 2607 CA THR B 118 -10.906 15.150 -4.053 1.00 0.00

ATOM 2608 HA THR B 118 -11.384 15.542 -3.151 1.00 0.00

ATOM 2609 C THR B 118 -9.938 16.218 -4.629 1.00 0.00

ATOM 2610 O THR B 118 -9.910 16.474 -5.831 1.00 0.00

ATOM 2611 CB THR B 118 -10.311 13.708 -3.856 1.00 0.00

ATOM 2612 HB THR B 118 -11.150 13.079 -3.537 1.00 0.00

ATOM 2613 OG1 THR B 118 -9.303 13.598 -2.863 1.00 0.00

ATOM 2614 HG1 THR B 118 -8.459 13.831 -3.285 1.00 0.00

ATOM 2615 CG2 THR B 118 -9.781 13.007 -5.128 1.00 0.00

ATOM 2616 HG21 THR B 118 -10.596 12.865 -5.866 1.00 0.00

ATOM 2617 HG22 THR B 118 -8.986 13.641 -5.570 1.00 0.00

ATOM 2618 HG23 THR B 118 -9.357 12.018 -4.829 1.00 0.00

ATOM 2619 N PRO B 119 -9.121 16.864 -3.771 1.00 0.00

ATOM 2620 CA PRO B 119 -7.893 17.498 -4.242 1.00 0.00

ATOM 2621 HA PRO B 119 -8.150 18.231 -5.011 1.00 0.00

ATOM 2622 CD PRO B 119 -9.367 17.022 -2.341 1.00 0.00

ATOM 2623 HD1 PRO B 119 -9.640 16.086 -1.859 1.00 0.00

ATOM 2624 HD2 PRO B 119 -10.182 17.734 -2.192 1.00 0.00

ATOM 2625 C PRO B 119 -6.934 16.456 -4.903 1.00 0.00

ATOM 2626 O PRO B 119 -7.125 15.240 -4.825 1.00 0.00

ATOM 2627 CB PRO B 119 -7.347 18.220 -3.005 1.00 0.00

ATOM 2628 HB1 PRO B 119 -7.642 19.272 -3.057 1.00 0.00

ATOM 2629 HB2 PRO B 119 -6.262 18.179 -2.930 1.00 0.00

ATOM 2630 CG PRO B 119 -8.055 17.588 -1.799 1.00 0.00

ATOM 2631 HG1 PRO B 119 -8.231 18.310 -0.998 1.00 0.00

ATOM 2632 HG2 PRO B 119 -7.443 16.775 -1.414 1.00 0.00

ATOM 2633 N GLU B 120 -5.986 17.011 -5.676 1.00 0.00

ATOM 2634 HN GLU B 120 -5.945 18.004 -5.572 1.00 0.00

ATOM 2635 CA GLU B 120 -5.059 16.213 -6.485 1.00 0.00

ATOM 2636 HA GLU B 120 -4.383 16.936 -6.941 1.00 0.00

ATOM 2637 C GLU B 120 -5.725 15.442 -7.667 1.00 0.00

ATOM 2638 O GLU B 120 -4.992 14.824 -8.435 1.00 0.00

ATOM 2639 CB GLU B 120 -4.228 15.184 -5.670 1.00 0.00

ATOM 2640 HB1 GLU B 120 -3.642 14.630 -6.402 1.00 0.00

ATOM 2641 HB2 GLU B 120 -4.896 14.464 -5.199 1.00 0.00

ATOM 2642 CG GLU B 120 -3.235 15.714 -4.613 1.00 0.00

ATOM 2643 HG1 GLU B 120 -3.790 16.108 -3.755 1.00 0.00

ATOM 2644 HG2 GLU B 120 -2.638 16.525 -5.031 1.00 0.00

ATOM 2645 CD GLU B 120 -2.250 14.622 -4.112 1.00 0.00

ATOM 2646 OE1 GLU B 120 -1.960 13.656 -4.834 1.00 0.00

ATOM 2647 OE2 GLU B 120 -1.746 14.731 -2.985 1.00 0.00

ATOM 2648 N GLY B 121 -7.072 15.456 -7.805 1.00 0.00

ATOM 2649 HN GLY B 121 -7.696 15.884 -7.142 1.00 0.00

ATOM 2650 CA GLY B 121 -7.705 14.683 -8.883 1.00 0.00

ATOM 2651 HA1 GLY B 121 -7.045 13.862 -9.173 1.00 0.00

ATOM 2652 HA2 GLY B 121 -7.844 15.340 -9.743 1.00 0.00

ATOM 2653 C GLY B 121 -9.050 14.109 -8.407 1.00 0.00

ATOM 2654 O GLY B 121 -9.587 14.529 -7.397 1.00 0.00

ATOM 2655 N GLU B 122 -9.524 13.102 -9.173 1.00 0.00

ATOM 2656 HN GLU B 122 -9.022 12.715 -9.944 1.00 0.00

ATOM 2657 CA GLU B 122 -10.714 12.325 -8.781 1.00 0.00

ATOM 2658 HA GLU B 122 -11.119 12.754 -7.867 1.00 0.00

ATOM 2659 C GLU B 122 -10.212 10.896 -8.474 1.00 0.00

ATOM 2660 O GLU B 122 -9.229 10.451 -9.057 1.00 0.00

ATOM 2661 CB GLU B 122 -11.832 12.343 -9.860 1.00 0.00

ATOM 2662 HB1 GLU B 122 -12.023 11.335 -10.231 1.00 0.00

ATOM 2663 HB2 GLU B 122 -11.516 12.942 -10.713 1.00 0.00

ATOM 2664 CG GLU B 122 -13.153 12.916 -9.309 1.00 0.00

ATOM 2665 HG1 GLU B 122 -13.043 13.987 -9.137 1.00 0.00

ATOM 2666 HG2 GLU B 122 -13.349 12.443 -8.354 1.00 0.00

ATOM 2667 CD GLU B 122 -14.381 12.672 -10.197 1.00 0.00

ATOM 2668 OE1 GLU B 122 -15.219 11.837 -9.860 1.00 0.00

ATOM 2669 OE2 GLU B 122 -14.501 13.333 -11.223 1.00 0.00

ATOM 2670 N VAL B 123 -10.885 10.223 -7.517 1.00 0.00

ATOM 2671 HN VAL B 123 -11.763 10.592 -7.201 1.00 0.00

ATOM 2672 CA VAL B 123 -10.473 8.888 -7.076 1.00 0.00

ATOM 2673 HA VAL B 123 -9.693 8.536 -7.753 1.00 0.00

ATOM 2674 C VAL B 123 -11.663 7.920 -7.178 1.00 0.00

ATOM 2675 O VAL B 123 -12.810 8.276 -6.920 1.00 0.00

ATOM 2676 CB VAL B 123 -9.895 8.949 -5.640 1.00 0.00

ATOM 2677 HB VAL B 123 -9.104 9.701 -5.661 1.00 0.00

ATOM 2678 CG1 VAL B 123 -10.923 9.414 -4.578 1.00 0.00

ATOM 2679 HG11 VAL B 123 -11.820 9.857 -5.018 1.00 0.00

ATOM 2680 HG12 VAL B 123 -11.262 8.589 -3.944 1.00 0.00

ATOM 2681 HG13 VAL B 123 -10.502 10.168 -3.908 1.00 0.00

ATOM 2682 CG2 VAL B 123 -9.196 7.629 -5.236 1.00 0.00

ATOM 2683 HG21 VAL B 123 -8.989 6.988 -6.096 1.00 0.00

ATOM 2684 HG22 VAL B 123 -8.235 7.817 -4.751 1.00 0.00

ATOM 2685 HG23 VAL B 123 -9.796 7.041 -4.538 1.00 0.00

ATOM 2686 N GLY B 124 -11.306 6.666 -7.541 1.00 0.00

ATOM 2687 HN GLY B 124 -10.341 6.428 -7.684 1.00 0.00

ATOM 2688 CA GLY B 124 -12.285 5.597 -7.452 1.00 0.00

ATOM 2689 HA1 GLY B 124 -11.826 4.726 -7.912 1.00 0.00

ATOM 2690 HA2 GLY B 124 -13.172 5.884 -8.025 1.00 0.00

ATOM 2691 C GLY B 124 -12.662 5.280 -5.990 1.00 0.00

ATOM 2692 O GLY B 124 -11.874 5.421 -5.041 1.00 0.00

ATOM 2693 N ALA B 125 -13.939 4.853 -5.932 1.00 0.00

ATOM 2694 HN ALA B 125 -14.396 4.720 -6.816 1.00 0.00

ATOM 2695 CA ALA B 125 -14.629 4.621 -4.681 1.00 0.00

ATOM 2696 HA ALA B 125 -13.868 4.393 -3.941 1.00 0.00

ATOM 2697 C ALA B 125 -15.558 3.406 -4.853 1.00 0.00

ATOM 2698 O ALA B 125 -16.371 3.328 -5.769 1.00 0.00

ATOM 2699 CB ALA B 125 -15.397 5.883 -4.253 1.00 0.00

ATOM 2700 HB1 ALA B 125 -15.338 6.670 -5.005 1.00 0.00

ATOM 2701 HB2 ALA B 125 -16.452 5.686 -4.070 1.00 0.00

ATOM 2702 HB3 ALA B 125 -14.978 6.303 -3.336 1.00 0.00

ATOM 2703 N ILE B 126 -15.383 2.468 -3.913 1.00 0.00

ATOM 2704 HN ILE B 126 -14.768 2.672 -3.146 1.00 0.00

ATOM 2705 CA ILE B 126 -16.069 1.185 -3.949 1.00 0.00

ATOM 2706 HA ILE B 126 -16.676 1.119 -4.855 1.00 0.00

ATOM 2707 C ILE B 126 -16.969 1.138 -2.713 1.00 0.00

ATOM 2708 O ILE B 126 -16.494 1.387 -1.609 1.00 0.00

ATOM 2709 CB ILE B 126 -14.995 0.067 -3.902 1.00 0.00

ATOM 2710 HB ILE B 126 -14.300 0.312 -3.095 1.00 0.00

ATOM 2711 CG2 ILE B 126 -15.569 -1.327 -3.575 1.00 0.00

ATOM 2712 HG21 ILE B 126 -16.651 -1.340 -3.475 1.00 0.00

ATOM 2713 HG22 ILE B 126 -15.326 -2.060 -4.342 1.00 0.00

ATOM 2714 HG23 ILE B 126 -15.159 -1.710 -2.641 1.00 0.00

ATOM 2715 CG1 ILE B 126 -14.157 0.030 -5.195 1.00 0.00

ATOM 2716 HG11 ILE B 126 -13.695 1.003 -5.367 1.00 0.00

ATOM 2717 HG12 ILE B 126 -14.815 -0.168 -6.043 1.00 0.00

ATOM 2718 CD1 ILE B 126 -13.057 -1.043 -5.180 1.00 0.00

ATOM 2719 HD11 ILE B 126 -12.815 -1.331 -4.152 1.00 0.00

ATOM 2720 HD12 ILE B 126 -13.391 -1.933 -5.722 1.00 0.00

ATOM 2721 HD13 ILE B 126 -12.148 -0.680 -5.665 1.00 0.00

ATOM 2722 N ALA B 127 -18.243 0.781 -2.944 1.00 0.00

ATOM 2723 HN ALA B 127 -18.492 0.661 -3.909 1.00 0.00

ATOM 2724 CA ALA B 127 -19.182 0.638 -1.829 1.00 0.00

ATOM 2725 HA ALA B 127 -18.739 1.082 -0.932 1.00 0.00

ATOM 2726 C ALA B 127 -19.444 -0.864 -1.536 1.00 0.00

ATOM 2727 O ALA B 127 -20.440 -1.460 -1.946 1.00 0.00

ATOM 2728 CB ALA B 127 -20.457 1.430 -2.137 1.00 0.00

ATOM 2729 HB1 ALA B 127 -20.324 2.129 -2.965 1.00 0.00

ATOM 2730 HB2 ALA B 127 -21.279 0.766 -2.392 1.00 0.00

ATOM 2731 HB3 ALA B 127 -20.757 2.012 -1.267 1.00 0.00

ATOM 2732 N LEU B 128 -18.461 -1.399 -0.795 1.00 0.00

ATOM 2733 HN LEU B 128 -17.776 -0.756 -0.467 1.00 0.00

ATOM 2734 CA LEU B 128 -18.482 -2.755 -0.243 1.00 0.00

ATOM 2735 HA LEU B 128 -19.492 -3.156 -0.330 1.00 0.00

ATOM 2736 C LEU B 128 -18.083 -2.642 1.225 1.00 0.00

ATOM 2737 O LEU B 128 -17.298 -1.752 1.546 1.00 0.00

ATOM 2738 CB LEU B 128 -17.429 -3.646 -0.935 1.00 0.00

ATOM 2739 HB1 LEU B 128 -17.203 -4.522 -0.322 1.00 0.00

ATOM 2740 HB2 LEU B 128 -16.489 -3.100 -1.037 1.00 0.00

ATOM 2741 CG LEU B 128 -17.866 -4.118 -2.323 1.00 0.00

ATOM 2742 HG LEU B 128 -18.164 -3.227 -2.875 1.00 0.00

ATOM 2743 CD1 LEU B 128 -16.706 -4.768 -3.109 1.00 0.00

ATOM 2744 HD11 LEU B 128 -15.787 -4.819 -2.529 1.00 0.00

ATOM 2745 HD12 LEU B 128 -16.930 -5.770 -3.471 1.00 0.00

ATOM 2746 HD13 LEU B 128 -16.474 -4.188 -3.995 1.00 0.00

ATOM 2747 CD2 LEU B 128 -19.104 -5.021 -2.231 1.00 0.00

ATOM 2748 HD21 LEU B 128 -19.364 -5.276 -1.205 1.00 0.00

ATOM 2749 HD22 LEU B 128 -19.951 -4.487 -2.658 1.00 0.00

ATOM 2750 HD23 LEU B 128 -18.992 -5.951 -2.791 1.00 0.00

ATOM 2751 N ASP B 129 -18.611 -3.582 2.047 1.00 0.00

ATOM 2752 HN ASP B 129 -19.224 -4.267 1.645 1.00 0.00

ATOM 2753 CA ASP B 129 -18.240 -3.671 3.466 1.00 0.00

ATOM 2754 HA ASP B 129 -17.432 -2.958 3.654 1.00 0.00

ATOM 2755 C ASP B 129 -17.726 -5.098 3.751 1.00 0.00

ATOM 2756 O ASP B 129 -18.474 -6.077 3.753 1.00 0.00

ATOM 2757 CB ASP B 129 -19.449 -3.343 4.376 1.00 0.00

ATOM 2758 HB1 ASP B 129 -19.874 -4.249 4.811 1.00 0.00

ATOM 2759 HB2 ASP B 129 -20.258 -2.904 3.795 1.00 0.00

ATOM 2760 CG ASP B 129 -19.077 -2.352 5.484 1.00 0.00

ATOM 2761 OD1 ASP B 129 -18.724 -2.769 6.587 1.00 0.00

ATOM 2762 OD2 ASP B 129 -19.130 -1.150 5.256 1.00 0.00

ATOM 2763 N PHE B 130 -16.409 -5.167 4.005 1.00 0.00

ATOM 2764 HN PHE B 130 -15.850 -4.374 3.761 1.00 0.00

ATOM 2765 CA PHE B 130 -15.825 -6.384 4.568 1.00 0.00

ATOM 2766 HA PHE B 130 -16.544 -7.199 4.475 1.00 0.00

ATOM 2767 C PHE B 130 -15.585 -6.242 6.093 1.00 0.00

ATOM 2768 O PHE B 130 -15.759 -5.216 6.749 1.00 0.00

ATOM 2769 CB PHE B 130 -14.565 -6.784 3.765 1.00 0.00

ATOM 2770 HB1 PHE B 130 -14.084 -7.660 4.199 1.00 0.00

ATOM 2771 HB2 PHE B 130 -13.833 -5.977 3.786 1.00 0.00

ATOM 2772 CG PHE B 130 -14.906 -7.108 2.331 1.00 0.00

ATOM 2773 CD1 PHE B 130 -15.819 -8.119 2.036 1.00 0.00

ATOM 2774 HD1 PHE B 130 -16.233 -8.703 2.847 1.00 0.00

ATOM 2775 CE1 PHE B 130 -16.205 -8.368 0.725 1.00 0.00

ATOM 2776 HE1 PHE B 130 -16.963 -9.099 0.476 1.00 0.00

ATOM 2777 CZ PHE B 130 -15.644 -7.634 -0.307 1.00 0.00

ATOM 2778 HZ PHE B 130 -15.938 -7.810 -1.334 1.00 0.00

ATOM 2779 CE2 PHE B 130 -14.707 -6.648 -0.035 1.00 0.00

ATOM 2780 HE2 PHE B 130 -14.287 -6.072 -0.855 1.00 0.00

ATOM 2781 CD2 PHE B 130 -14.345 -6.387 1.280 1.00 0.00

ATOM 2782 HD2 PHE B 130 -13.631 -5.598 1.470 1.00 0.00

ATOM 2783 N LYS B 131 -15.162 -7.414 6.627 1.00 0.00

ATOM 2784 HN LYS B 131 -14.947 -8.194 6.025 1.00 0.00

ATOM 2785 CA LYS B 131 -14.899 -7.469 8.064 1.00 0.00

ATOM 2786 HA LYS B 131 -15.766 -7.039 8.558 1.00 0.00

ATOM 2787 C LYS B 131 -13.624 -6.658 8.373 1.00 0.00

ATOM 2788 O LYS B 131 -12.691 -6.683 7.588 1.00 0.00

ATOM 2789 CB LYS B 131 -14.696 -8.929 8.508 1.00 0.00

ATOM 2790 HB1 LYS B 131 -14.440 -8.965 9.568 1.00 0.00

ATOM 2791 HB2 LYS B 131 -13.843 -9.340 7.965 1.00 0.00

ATOM 2792 CG LYS B 131 -15.903 -9.840 8.252 1.00 0.00

ATOM 2793 HG1 LYS B 131 -15.608 -10.882 8.403 1.00 0.00

ATOM 2794 HG2 LYS B 131 -16.185 -9.752 7.206 1.00 0.00

ATOM 2795 CD LYS B 131 -17.115 -9.536 9.140 1.00 0.00

ATOM 2796 HD1 LYS B 131 -17.322 -8.467 9.142 1.00 0.00

ATOM 2797 HD2 LYS B 131 -16.896 -9.820 10.173 1.00 0.00

ATOM 2798 CE LYS B 131 -18.368 -10.271 8.652 1.00 0.00

ATOM 2799 HE1 LYS B 131 -19.188 -10.126 9.362 1.00 0.00

ATOM 2800 HE2 LYS B 131 -18.170 -11.347 8.598 1.00 0.00

ATOM 2801 NZ LYS B 131 -18.815 -9.802 7.330 1.00 0.00

ATOM 2802 HZ1 LYS B 131 -18.277 -8.963 7.019 1.00 0.00

ATOM 2803 HZ2 LYS B 131 -18.679 -10.562 6.627 1.00 0.00

ATOM 2804 HZ3 LYS B 131 -19.810 -9.500 7.349 1.00 0.00

ATOM 2805 N PRO B 132 -13.582 -5.960 9.536 1.00 0.00

ATOM 2806 CA PRO B 132 -12.324 -5.388 10.020 1.00 0.00

ATOM 2807 HA PRO B 132 -12.095 -4.517 9.407 1.00 0.00

ATOM 2808 CD PRO B 132 -14.709 -5.719 10.435 1.00 0.00

ATOM 2809 HD1 PRO B 132 -14.974 -6.649 10.937 1.00 0.00

ATOM 2810 HD2 PRO B 132 -15.577 -5.336 9.898 1.00 0.00

ATOM 2811 C PRO B 132 -11.133 -6.381 9.939 1.00 0.00

ATOM 2812 O PRO B 132 -11.293 -7.576 10.177 1.00 0.00

ATOM 2813 CB PRO B 132 -12.656 -4.936 11.446 1.00 0.00

ATOM 2814 HB1 PRO B 132 -12.101 -4.046 11.746 1.00 0.00

ATOM 2815 HB2 PRO B 132 -12.425 -5.733 12.155 1.00 0.00

ATOM 2816 CG PRO B 132 -14.168 -4.690 11.431 1.00 0.00

ATOM 2817 HG1 PRO B 132 -14.360 -3.679 11.062 1.00 0.00

ATOM 2818 HG2 PRO B 132 -14.618 -4.763 12.421 1.00 0.00

ATOM 2819 N GLY B 133 -10.000 -5.819 9.477 1.00 0.00

ATOM 2820 HN GLY B 133 -9.995 -4.836 9.272 1.00 0.00

ATOM 2821 CA GLY B 133 -8.755 -6.529 9.214 1.00 0.00

ATOM 2822 HA1 GLY B 133 -8.762 -7.485 9.739 1.00 0.00

ATOM 2823 HA2 GLY B 133 -7.942 -5.907 9.590 1.00 0.00

ATOM 2824 C GLY B 133 -8.535 -6.768 7.714 1.00 0.00

ATOM 2825 O GLY B 133 -7.505 -7.298 7.297 1.00 0.00

ATOM 2826 N THR B 134 -9.580 -6.432 6.932 1.00 0.00

ATOM 2827 HN THR B 134 -10.233 -5.744 7.253 1.00 0.00

ATOM 2828 CA THR B 134 -9.537 -6.721 5.509 1.00 0.00

ATOM 2829 HA THR B 134 -9.041 -7.684 5.381 1.00 0.00

ATOM 2830 C THR B 134 -8.716 -5.646 4.762 1.00 0.00

ATOM 2831 O THR B 134 -8.339 -5.888 3.613 1.00 0.00

ATOM 2832 CB THR B 134 -10.977 -6.841 4.946 1.00 0.00

ATOM 2833 HB THR B 134 -11.489 -5.872 5.057 1.00 0.00

ATOM 2834 OG1 THR B 134 -11.723 -7.849 5.607 1.00 0.00

ATOM 2835 HG1 THR B 134 -11.631 -7.750 6.555 1.00 0.00

ATOM 2836 CG2 THR B 134 -11.030 -7.287 3.468 1.00 0.00

ATOM 2837 HG21 THR B 134 -10.084 -7.718 3.156 1.00 0.00

ATOM 2838 HG22 THR B 134 -11.761 -8.083 3.290 1.00 0.00

ATOM 2839 HG23 THR B 134 -11.264 -6.466 2.785 1.00 0.00

ATOM 2840 N SER B 135 -8.531 -4.484 5.431 1.00 0.00

ATOM 2841 HN SER B 135 -8.892 -4.408 6.364 1.00 0.00

ATOM 2842 CA SER B 135 -7.911 -3.343 4.759 1.00 0.00

ATOM 2843 HA SER B 135 -8.493 -3.225 3.848 1.00 0.00

ATOM 2844 C SER B 135 -6.466 -3.637 4.316 1.00 0.00

ATOM 2845 O SER B 135 -5.621 -4.123 5.068 1.00 0.00

ATOM 2846 CB SER B 135 -8.061 -2.010 5.525 1.00 0.00

ATOM 2847 HB1 SER B 135 -9.107 -1.684 5.525 1.00 0.00

ATOM 2848 HB2 SER B 135 -7.533 -1.246 4.950 1.00 0.00

ATOM 2849 OG SER B 135 -7.575 -1.982 6.860 1.00 0.00

ATOM 2850 HG SER B 135 -7.919 -2.757 7.353 1.00 0.00

ATOM 2851 N GLY B 136 -6.288 -3.316 3.024 1.00 0.00

ATOM 2852 HN GLY B 136 -7.025 -2.886 2.501 1.00 0.00

ATOM 2853 CA GLY B 136 -5.070 -3.638 2.316 1.00 0.00

ATOM 2854 HA1 GLY B 136 -4.269 -3.861 3.017 1.00 0.00

ATOM 2855 HA2 GLY B 136 -4.780 -2.761 1.742 1.00 0.00

ATOM 2856 C GLY B 136 -5.318 -4.792 1.352 1.00 0.00

ATOM 2857 O GLY B 136 -4.429 -5.190 0.604 1.00 0.00

ATOM 2858 N SER B 137 -6.547 -5.345 1.303 1.00 0.00

ATOM 2859 HN SER B 137 -7.223 -5.042 1.980 1.00 0.00

ATOM 2860 CA SER B 137 -6.791 -6.248 0.163 1.00 0.00

ATOM 2861 HA SER B 137 -6.063 -7.055 0.235 1.00 0.00

ATOM 2862 C SER B 137 -6.625 -5.472 -1.185 1.00 0.00

ATOM 2863 O SER B 137 -7.054 -4.322 -1.264 1.00 0.00

ATOM 2864 CB SER B 137 -8.206 -6.817 0.263 1.00 0.00

ATOM 2865 HB1 SER B 137 -8.397 -7.404 -0.639 1.00 0.00

ATOM 2866 HB2 SER B 137 -8.924 -5.989 0.257 1.00 0.00

ATOM 2867 OG SER B 137 -8.404 -7.647 1.411 1.00 0.00

ATOM 2868 HG SER B 137 -7.935 -7.243 2.150 1.00 0.00

ATOM 2869 N PRO B 138 -5.952 -6.105 -2.192 1.00 0.00

ATOM 2870 CA PRO B 138 -5.675 -5.495 -3.496 1.00 0.00

ATOM 2871 HA PRO B 138 -5.334 -4.468 -3.367 1.00 0.00

ATOM 2872 CD PRO B 138 -5.372 -7.445 -2.069 1.00 0.00

ATOM 2873 HD1 PRO B 138 -6.116 -8.178 -1.754 1.00 0.00

ATOM 2874 HD2 PRO B 138 -4.563 -7.423 -1.339 1.00 0.00

ATOM 2875 C PRO B 138 -6.913 -5.506 -4.415 1.00 0.00

ATOM 2876 O PRO B 138 -7.688 -6.449 -4.421 1.00 0.00

ATOM 2877 CB PRO B 138 -4.564 -6.386 -4.084 1.00 0.00

ATOM 2878 HB1 PRO B 138 -3.584 -6.020 -3.768 1.00 0.00

ATOM 2879 HB2 PRO B 138 -4.572 -6.436 -5.175 1.00 0.00

ATOM 2880 CG PRO B 138 -4.812 -7.759 -3.456 1.00 0.00

ATOM 2881 HG1 PRO B 138 -3.932 -8.403 -3.432 1.00 0.00

ATOM 2882 HG2 PRO B 138 -5.575 -8.273 -4.039 1.00 0.00

ATOM 2883 N ILE B 139 -7.058 -4.424 -5.174 1.00 0.00

ATOM 2884 HN ILE B 139 -6.381 -3.700 -5.218 1.00 0.00

ATOM 2885 CA ILE B 139 -8.098 -4.276 -6.176 1.00 0.00

ATOM 2886 HA ILE B 139 -8.835 -5.049 -6.008 1.00 0.00

ATOM 2887 C ILE B 139 -7.413 -4.422 -7.558 1.00 0.00

ATOM 2888 O ILE B 139 -6.428 -3.715 -7.782 1.00 0.00

ATOM 2889 CB ILE B 139 -8.698 -2.870 -5.981 1.00 0.00

ATOM 2890 HB ILE B 139 -7.911 -2.154 -6.225 1.00 0.00

ATOM 2891 CG2 ILE B 139 -9.877 -2.623 -6.932 1.00 0.00

ATOM 2892 HG21 ILE B 139 -10.055 -3.483 -7.575 1.00 0.00

ATOM 2893 HG22 ILE B 139 -10.794 -2.412 -6.388 1.00 0.00

ATOM 2894 HG23 ILE B 139 -9.665 -1.778 -7.588 1.00 0.00

ATOM 2895 CG1 ILE B 139 -9.100 -2.575 -4.517 1.00 0.00

ATOM 2896 HG11 ILE B 139 -8.301 -2.835 -3.821 1.00 0.00

ATOM 2897 HG12 ILE B 139 -9.960 -3.192 -4.246 1.00 0.00

ATOM 2898 CD1 ILE B 139 -9.434 -1.092 -4.294 1.00 0.00

ATOM 2899 HD11 ILE B 139 -9.296 -0.518 -5.212 1.00 0.00

ATOM 2900 HD12 ILE B 139 -10.470 -0.965 -3.971 1.00 0.00

ATOM 2901 HD13 ILE B 139 -8.795 -0.638 -3.534 1.00 0.00

ATOM 2902 N VAL B 140 -7.898 -5.367 -8.406 1.00 0.00

ATOM 2903 HN VAL B 140 -8.777 -5.812 -8.234 1.00 0.00

ATOM 2904 CA VAL B 140 -7.269 -5.691 -9.691 1.00 0.00

ATOM 2905 HA VAL B 140 -6.412 -5.036 -9.809 1.00 0.00

ATOM 2906 C VAL B 140 -8.262 -5.417 -10.827 1.00 0.00

ATOM 2907 O VAL B 140 -9.470 -5.551 -10.644 1.00 0.00

ATOM 2908 CB VAL B 140 -6.822 -7.177 -9.762 1.00 0.00

ATOM 2909 HB VAL B 140 -6.569 -7.401 -10.802 1.00 0.00

ATOM 2910 CG1 VAL B 140 -5.529 -7.432 -8.968 1.00 0.00

ATOM 2911 HG11 VAL B 140 -5.206 -6.528 -8.455 1.00 0.00

ATOM 2912 HG12 VAL B 140 -5.643 -8.203 -8.205 1.00 0.00

ATOM 2913 HG13 VAL B 140 -4.711 -7.747 -9.622 1.00 0.00

ATOM 2914 CG2 VAL B 140 -7.940 -8.176 -9.393 1.00 0.00

ATOM 2915 HG21 VAL B 140 -8.913 -7.695 -9.277 1.00 0.00

ATOM 2916 HG22 VAL B 140 -8.072 -8.930 -10.173 1.00 0.00

ATOM 2917 HG23 VAL B 140 -7.744 -8.721 -8.468 1.00 0.00

ATOM 2918 N ASN B 141 -7.665 -5.066 -11.981 1.00 0.00

ATOM 2919 HN ASN B 141 -6.662 -5.111 -12.019 1.00 0.00

ATOM 2920 CA ASN B 141 -8.430 -4.970 -13.221 1.00 0.00

ATOM 2921 HA ASN B 141 -9.481 -4.788 -12.986 1.00 0.00

ATOM 2922 C ASN B 141 -8.341 -6.328 -13.905 1.00 0.00

ATOM 2923 O ASN B 141 -7.673 -7.235 -13.412 1.00 0.00

ATOM 2924 CB ASN B 141 -7.888 -3.845 -14.133 1.00 0.00

ATOM 2925 HB1 ASN B 141 -7.946 -2.883 -13.624 1.00 0.00

ATOM 2926 HB2 ASN B 141 -8.532 -3.747 -15.008 1.00 0.00

ATOM 2927 CG ASN B 141 -6.442 -4.086 -14.627 1.00 0.00

ATOM 2928 OD1 ASN B 141 -5.705 -4.901 -14.078 1.00 0.00

ATOM 2929 ND2 ASN B 141 -6.110 -3.383 -15.719 1.00 0.00

ATOM 2930 HD21 ASN B 141 -5.389 -3.626 -16.372 1.00 0.00

ATOM 2931 HD22 ASN B 141 -6.670 -2.598 -15.989 1.00 0.00

ATOM 2932 N ARG B 142 -9.013 -6.376 -15.069 1.00 0.00

ATOM 2933 HN ARG B 142 -9.536 -5.575 -15.360 1.00 0.00

ATOM 2934 CA ARG B 142 -9.150 -7.583 -15.872 1.00 0.00

ATOM 2935 HA ARG B 142 -9.710 -8.294 -15.268 1.00 0.00

ATOM 2936 C ARG B 142 -7.785 -8.216 -16.169 1.00 0.00

ATOM 2937 O ARG B 142 -7.604 -9.419 -16.013 1.00 0.00

ATOM 2938 CB ARG B 142 -9.961 -7.234 -17.130 1.00 0.00

ATOM 2939 HB1 ARG B 142 -9.400 -6.507 -17.720 1.00 0.00

ATOM 2940 HB2 ARG B 142 -10.875 -6.734 -16.805 1.00 0.00

ATOM 2941 CG ARG B 142 -10.362 -8.419 -18.031 1.00 0.00

ATOM 2942 HG1 ARG B 142 -9.469 -8.838 -18.497 1.00 0.00

ATOM 2943 HG2 ARG B 142 -10.975 -8.030 -18.846 1.00 0.00

ATOM 2944 CD ARG B 142 -11.157 -9.512 -17.288 1.00 0.00

ATOM 2945 HD1 ARG B 142 -11.955 -9.068 -16.688 1.00 0.00

ATOM 2946 HD2 ARG B 142 -10.503 -10.070 -16.617 1.00 0.00

ATOM 2947 NE ARG B 142 -11.849 -10.420 -18.216 1.00 0.00

ATOM 2948 HE ARG B 142 -12.455 -9.949 -18.854 1.00 0.00

ATOM 2949 CZ ARG B 142 -11.894 -11.773 -18.076 1.00 0.00

ATOM 2950 NH1 ARG B 142 -11.182 -12.364 -17.128 1.00 0.00

ATOM 2951 HH11 ARG B 142 -10.629 -11.836 -16.481 1.00 0.00

ATOM 2952 HH12 ARG B 142 -11.227 -13.368 -17.036 1.00 0.00

ATOM 2953 NH2 ARG B 142 -12.657 -12.519 -18.877 1.00 0.00

ATOM 2954 HH21 ARG B 142 -13.258 -12.126 -19.568 1.00 0.00

ATOM 2955 HH22 ARG B 142 -12.649 -13.529 -18.772 1.00 0.00

ATOM 2956 N GLU B 143 -6.799 -7.379 -16.515 1.00 0.00

ATOM 2957 HN GLU B 143 -6.990 -6.409 -16.694 1.00 0.00

ATOM 2958 CA GLU B 143 -5.483 -7.942 -16.765 1.00 0.00

ATOM 2959 HA GLU B 143 -5.614 -8.766 -17.469 1.00 0.00

ATOM 2960 C GLU B 143 -4.827 -8.529 -15.500 1.00 0.00

ATOM 2961 O GLU B 143 -3.657 -8.877 -15.568 1.00 0.00

ATOM 2962 CB GLU B 143 -4.521 -6.899 -17.370 1.00 0.00

ATOM 2963 HB1 GLU B 143 -3.561 -7.388 -17.526 1.00 0.00

ATOM 2964 HB2 GLU B 143 -4.344 -6.099 -16.644 1.00 0.00

ATOM 2965 CG GLU B 143 -4.952 -6.317 -18.729 1.00 0.00

ATOM 2966 HG1 GLU B 143 -5.595 -7.017 -19.265 1.00 0.00

ATOM 2967 HG2 GLU B 143 -4.085 -6.123 -19.364 1.00 0.00

ATOM 2968 CD GLU B 143 -5.696 -5.000 -18.529 1.00 0.00

ATOM 2969 OE1 GLU B 143 -5.103 -3.944 -18.707 1.00 0.00

ATOM 2970 OE2 GLU B 143 -6.850 -5.043 -18.150 1.00 0.00

ATOM 2971 N GLY B 144 -5.545 -8.619 -14.366 1.00 0.00

ATOM 2972 HN GLY B 144 -6.520 -8.384 -14.353 1.00 0.00

ATOM 2973 CA GLY B 144 -4.926 -9.029 -13.112 1.00 0.00

ATOM 2974 HA1 GLY B 144 -4.608 -10.072 -13.186 1.00 0.00

ATOM 2975 HA2 GLY B 144 -5.689 -8.949 -12.341 1.00 0.00

ATOM 2976 C GLY B 144 -3.742 -8.118 -12.772 1.00 0.00

ATOM 2977 O GLY B 144 -2.634 -8.581 -12.529 1.00 0.00

ATOM 2978 N LYS B 145 -4.028 -6.804 -12.827 1.00 0.00

ATOM 2979 HN LYS B 145 -4.932 -6.450 -13.083 1.00 0.00

ATOM 2980 CA LYS B 145 -3.063 -5.798 -12.419 1.00 0.00

ATOM 2981 HA LYS B 145 -2.228 -6.317 -11.954 1.00 0.00

ATOM 2982 C LYS B 145 -3.742 -4.923 -11.345 1.00 0.00

ATOM 2983 O LYS B 145 -4.895 -4.511 -11.453 1.00 0.00

ATOM 2984 CB LYS B 145 -2.540 -5.010 -13.641 1.00 0.00

ATOM 2985 HB1 LYS B 145 -1.760 -4.314 -13.331 1.00 0.00

ATOM 2986 HB2 LYS B 145 -3.348 -4.402 -14.042 1.00 0.00

ATOM 2987 CG LYS B 145 -2.000 -5.897 -14.787 1.00 0.00

ATOM 2988 HG1 LYS B 145 -1.907 -5.290 -15.692 1.00 0.00

ATOM 2989 HG2 LYS B 145 -2.734 -6.667 -15.011 1.00 0.00

ATOM 2990 CD LYS B 145 -0.637 -6.553 -14.505 1.00 0.00

ATOM 2991 HD1 LYS B 145 -0.522 -6.700 -13.429 1.00 0.00

ATOM 2992 HD2 LYS B 145 0.152 -5.862 -14.810 1.00 0.00

ATOM 2993 CE LYS B 145 -0.435 -7.906 -15.214 1.00 0.00

ATOM 2994 HE1 LYS B 145 0.611 -8.207 -15.137 1.00 0.00

ATOM 2995 HE2 LYS B 145 -0.699 -7.858 -16.275 1.00 0.00

ATOM 2996 NZ LYS B 145 -1.212 -8.966 -14.588 1.00 0.00

ATOM 2997 HZ1 LYS B 145 -1.374 -8.706 -13.591 1.00 0.00

ATOM 2998 HZ2 LYS B 145 -2.151 -9.051 -15.023 1.00 0.00

ATOM 2999 HZ3 LYS B 145 -0.759 -9.896 -14.622 1.00 0.00

ATOM 3000 N ILE B 146 -2.947 -4.717 -10.289 1.00 0.00

ATOM 3001 HN ILE B 146 -1.995 -4.970 -10.376 1.00 0.00

ATOM 3002 CA ILE B 146 -3.445 -4.098 -9.083 1.00 0.00

ATOM 3003 HA ILE B 146 -4.447 -4.484 -8.920 1.00 0.00

ATOM 3004 C ILE B 146 -3.522 -2.591 -9.325 1.00 0.00

ATOM 3005 O ILE B 146 -2.520 -1.904 -9.528 1.00 0.00

ATOM 3006 CB ILE B 146 -2.561 -4.481 -7.878 1.00 0.00

ATOM 3007 HB ILE B 146 -1.562 -4.086 -8.058 1.00 0.00

ATOM 3008 CG2 ILE B 146 -3.106 -3.859 -6.582 1.00 0.00

ATOM 3009 HG21 ILE B 146 -4.071 -3.388 -6.750 1.00 0.00

ATOM 3010 HG22 ILE B 146 -3.255 -4.608 -5.807 1.00 0.00

ATOM 3011 HG23 ILE B 146 -2.430 -3.101 -6.202 1.00 0.00

ATOM 3012 CG1 ILE B 146 -2.439 -6.013 -7.721 1.00 0.00

ATOM 3013 HG11 ILE B 146 -2.227 -6.488 -8.677 1.00 0.00

ATOM 3014 HG12 ILE B 146 -3.395 -6.416 -7.394 1.00 0.00

ATOM 3015 CD1 ILE B 146 -1.377 -6.460 -6.709 1.00 0.00

ATOM 3016 HD11 ILE B 146 -0.792 -5.614 -6.357 1.00 0.00

ATOM 3017 HD12 ILE B 146 -1.854 -6.929 -5.846 1.00 0.00

ATOM 3018 HD13 ILE B 146 -0.695 -7.196 -7.140 1.00 0.00

ATOM 3019 N VAL B 147 -4.780 -2.150 -9.339 1.00 0.00

ATOM 3020 HN VAL B 147 -5.486 -2.847 -9.222 1.00 0.00

ATOM 3021 CA VAL B 147 -5.113 -0.746 -9.439 1.00 0.00

ATOM 3022 HA VAL B 147 -4.303 -0.235 -9.959 1.00 0.00

ATOM 3023 C VAL B 147 -5.220 -0.092 -8.043 1.00 0.00

ATOM 3024 O VAL B 147 -5.511 1.102 -7.952 1.00 0.00

ATOM 3025 CB VAL B 147 -6.402 -0.622 -10.283 1.00 0.00

ATOM 3026 HB VAL B 147 -6.594 0.447 -10.411 1.00 0.00

ATOM 3027 CG1 VAL B 147 -6.217 -1.226 -11.700 1.00 0.00

ATOM 3028 HG11 VAL B 147 -5.168 -1.420 -11.939 1.00 0.00

ATOM 3029 HG12 VAL B 147 -6.752 -2.171 -11.829 1.00 0.00

ATOM 3030 HG13 VAL B 147 -6.602 -0.539 -12.459 1.00 0.00

ATOM 3031 CG2 VAL B 147 -7.640 -1.223 -9.580 1.00 0.00

ATOM 3032 HG21 VAL B 147 -7.466 -1.411 -8.519 1.00 0.00

ATOM 3033 HG22 VAL B 147 -8.487 -0.534 -9.652 1.00 0.00

ATOM 3034 HG23 VAL B 147 -7.953 -2.175 -10.020 1.00 0.00

ATOM 3035 N GLY B 148 -4.995 -0.874 -6.958 1.00 0.00

ATOM 3036 HN GLY B 148 -4.920 -1.862 -7.101 1.00 0.00

ATOM 3037 CA GLY B 148 -4.918 -0.242 -5.640 1.00 0.00

ATOM 3038 HA1 GLY B 148 -5.685 0.526 -5.572 1.00 0.00

ATOM 3039 HA2 GLY B 148 -3.945 0.234 -5.553 1.00 0.00

ATOM 3040 C GLY B 148 -5.107 -1.246 -4.507 1.00 0.00

ATOM 3041 O GLY B 148 -5.119 -2.463 -4.722 1.00 0.00

ATOM 3042 N LEU B 149 -5.252 -0.631 -3.315 1.00 0.00

ATOM 3043 HN LEU B 149 -5.280 0.360 -3.408 1.00 0.00

ATOM 3044 CA LEU B 149 -5.647 -1.278 -2.074 1.00 0.00

ATOM 3045 HA LEU B 149 -5.865 -2.320 -2.294 1.00 0.00

ATOM 3046 C LEU B 149 -6.915 -0.605 -1.535 1.00 0.00

ATOM 3047 O LEU B 149 -7.018 0.620 -1.429 1.00 0.00

ATOM 3048 CB LEU B 149 -4.579 -1.179 -0.974 1.00 0.00

ATOM 3049 HB1 LEU B 149 -4.979 -1.672 -0.086 1.00 0.00

ATOM 3050 HB2 LEU B 149 -4.413 -0.143 -0.682 1.00 0.00

ATOM 3051 CG LEU B 149 -3.220 -1.820 -1.296 1.00 0.00

ATOM 3052 HG LEU B 149 -2.771 -1.255 -2.111 1.00 0.00

ATOM 3053 CD1 LEU B 149 -2.281 -1.702 -0.076 1.00 0.00

ATOM 3054 HD11 LEU B 149 -2.762 -1.202 0.763 1.00 0.00

ATOM 3055 HD12 LEU B 149 -1.962 -2.675 0.297 1.00 0.00

ATOM 3056 HD13 LEU B 149 -1.382 -1.137 -0.308 1.00 0.00

ATOM 3057 CD2 LEU B 149 -3.355 -3.282 -1.765 1.00 0.00

ATOM 3058 HD21 LEU B 149 -4.318 -3.696 -1.465 1.00 0.00

ATOM 3059 HD22 LEU B 149 -3.269 -3.352 -2.853 1.00 0.00

ATOM 3060 HD23 LEU B 149 -2.588 -3.931 -1.334 1.00 0.00

ATOM 3061 N TYR B 150 -7.821 -1.536 -1.196 1.00 0.00

ATOM 3062 HN TYR B 150 -7.526 -2.475 -1.313 1.00 0.00

ATOM 3063 CA TYR B 150 -9.039 -1.317 -0.448 1.00 0.00

ATOM 3064 HA TYR B 150 -9.563 -0.466 -0.881 1.00 0.00

ATOM 3065 C TYR B 150 -8.627 -0.972 0.990 1.00 0.00

ATOM 3066 O TYR B 150 -7.525 -1.285 1.450 1.00 0.00

ATOM 3067 CB TYR B 150 -9.864 -2.623 -0.571 1.00 0.00

ATOM 3068 HB1 TYR B 150 -9.238 -3.477 -0.305 1.00 0.00

ATOM 3069 HB2 TYR B 150 -10.144 -2.778 -1.613 1.00 0.00

ATOM 3070 CG TYR B 150 -11.120 -2.680 0.261 1.00 0.00

ATOM 3071 CD1 TYR B 150 -12.355 -2.298 -0.259 1.00 0.00

ATOM 3072 HD1 TYR B 150 -12.446 -1.977 -1.293 1.00 0.00

ATOM 3073 CE1 TYR B 150 -13.493 -2.337 0.543 1.00 0.00

ATOM 3074 HE1 TYR B 150 -14.443 -2.036 0.125 1.00 0.00

ATOM 3075 CZ TYR B 150 -13.418 -2.753 1.868 1.00 0.00

ATOM 3076 OH TYR B 150 -14.553 -2.727 2.668 1.00 0.00

ATOM 3077 HH TYR B 150 -14.293 -2.693 3.580 1.00 0.00

ATOM 3078 CE2 TYR B 150 -12.190 -3.172 2.377 1.00 0.00

ATOM 3079 HE2 TYR B 150 -12.070 -3.536 3.389 1.00 0.00

ATOM 3080 CD2 TYR B 150 -11.057 -3.132 1.576 1.00 0.00

ATOM 3081 HD2 TYR B 150 -10.115 -3.460 1.987 1.00 0.00

ATOM 3082 N GLY B 151 -9.586 -0.267 1.629 1.00 0.00

ATOM 3083 HN GLY B 151 -10.403 -0.180 1.057 1.00 0.00

ATOM 3084 CA GLY B 151 -9.606 -0.074 3.075 1.00 0.00

ATOM 3085 HA1 GLY B 151 -8.694 -0.497 3.474 1.00 0.00

ATOM 3086 HA2 GLY B 151 -10.468 -0.634 3.448 1.00 0.00

ATOM 3087 C GLY B 151 -9.719 1.377 3.577 1.00 0.00

ATOM 3088 O GLY B 151 -10.293 1.615 4.636 1.00 0.00

ATOM 3089 N ASN B 152 -9.132 2.344 2.845 1.00 0.00

ATOM 3090 HN ASN B 152 -8.795 2.146 1.924 1.00 0.00

ATOM 3091 CA ASN B 152 -9.172 3.731 3.330 1.00 0.00

ATOM 3092 HA ASN B 152 -9.193 3.714 4.420 1.00 0.00

ATOM 3093 C ASN B 152 -10.449 4.426 2.816 1.00 0.00

ATOM 3094 O ASN B 152 -10.447 5.066 1.763 1.00 0.00

ATOM 3095 CB ASN B 152 -7.918 4.516 2.903 1.00 0.00

ATOM 3096 HB1 ASN B 152 -7.831 4.583 1.821 1.00 0.00

ATOM 3097 HB2 ASN B 152 -7.024 3.996 3.244 1.00 0.00

ATOM 3098 CG ASN B 152 -7.979 5.935 3.516 1.00 0.00

ATOM 3099 OD1 ASN B 152 -8.651 6.154 4.526 1.00 0.00

ATOM 3100 ND2 ASN B 152 -7.284 6.869 2.872 1.00 0.00

ATOM 3101 HD21 ASN B 152 -7.262 7.843 3.099 1.00 0.00

ATOM 3102 HD22 ASN B 152 -6.616 6.537 2.197 1.00 0.00

ATOM 3103 N GLY B 153 -11.540 4.296 3.610 1.00 0.00

ATOM 3104 HN GLY B 153 -11.451 3.794 4.479 1.00 0.00

ATOM 3105 CA GLY B 153 -12.853 4.763 3.158 1.00 0.00

ATOM 3106 HA1 GLY B 153 -13.426 3.868 2.916 1.00 0.00

ATOM 3107 HA2 GLY B 153 -12.735 5.395 2.277 1.00 0.00

ATOM 3108 C GLY B 153 -13.601 5.551 4.231 1.00 0.00

ATOM 3109 O GLY B 153 -13.354 5.372 5.414 1.00 0.00

ATOM 3110 N VAL B 154 -14.511 6.437 3.785 1.00 0.00

ATOM 3111 HN VAL B 154 -14.891 6.316 2.866 1.00 0.00

ATOM 3112 CA VAL B 154 -15.327 7.234 4.717 1.00 0.00

ATOM 3113 HA VAL B 154 -14.916 7.145 5.723 1.00 0.00

ATOM 3114 C VAL B 154 -16.744 6.649 4.721 1.00 0.00

ATOM 3115 O VAL B 154 -17.053 5.735 3.970 1.00 0.00

ATOM 3116 CB VAL B 154 -15.391 8.727 4.311 1.00 0.00

ATOM 3117 HB VAL B 154 -16.184 9.204 4.887 1.00 0.00

ATOM 3118 CG1 VAL B 154 -14.129 9.518 4.703 1.00 0.00

ATOM 3119 HG11 VAL B 154 -13.370 8.904 5.189 1.00 0.00

ATOM 3120 HG12 VAL B 154 -13.673 10.002 3.844 1.00 0.00

ATOM 3121 HG13 VAL B 154 -14.379 10.326 5.392 1.00 0.00

ATOM 3122 CG2 VAL B 154 -15.794 8.923 2.839 1.00 0.00

ATOM 3123 HG21 VAL B 154 -16.124 7.994 2.374 1.00 0.00

ATOM 3124 HG22 VAL B 154 -16.612 9.638 2.749 1.00 0.00

ATOM 3125 HG23 VAL B 154 -14.968 9.304 2.239 1.00 0.00

ATOM 3126 N VAL B 155 -17.616 7.243 5.576 1.00 0.00

ATOM 3127 HN VAL B 155 -17.321 8.113 5.963 1.00 0.00

ATOM 3128 CA VAL B 155 -19.042 6.882 5.565 1.00 0.00

ATOM 3129 HA VAL B 155 -19.199 6.134 4.789 1.00 0.00

ATOM 3130 C VAL B 155 -19.870 8.136 5.209 1.00 0.00

ATOM 3131 O VAL B 155 -19.746 9.171 5.857 1.00 0.00

ATOM 3132 CB VAL B 155 -19.475 6.280 6.925 1.00 0.00

ATOM 3133 HB VAL B 155 -19.343 7.037 7.698 1.00 0.00

ATOM 3134 CG1 VAL B 155 -20.976 5.902 6.956 1.00 0.00

ATOM 3135 HG11 VAL B 155 -21.474 6.047 5.997 1.00 0.00

ATOM 3136 HG12 VAL B 155 -21.154 4.862 7.225 1.00 0.00

ATOM 3137 HG13 VAL B 155 -21.519 6.506 7.685 1.00 0.00

ATOM 3138 CG2 VAL B 155 -18.577 5.090 7.322 1.00 0.00

ATOM 3139 HG21 VAL B 155 -17.918 4.788 6.509 1.00 0.00

ATOM 3140 HG22 VAL B 155 -17.961 5.326 8.189 1.00 0.00

ATOM 3141 HG23 VAL B 155 -19.155 4.203 7.575 1.00 0.00

ATOM 3142 N THR B 156 -20.708 7.967 4.173 1.00 0.00

ATOM 3143 HN THR B 156 -20.772 7.070 3.727 1.00 0.00

ATOM 3144 CA THR B 156 -21.627 9.002 3.711 1.00 0.00

ATOM 3145 HA THR B 156 -21.053 9.907 3.551 1.00 0.00

ATOM 3146 C THR B 156 -22.675 9.282 4.806 1.00 0.00

ATOM 3147 O THR B 156 -22.671 8.612 5.833 1.00 0.00

ATOM 3148 CB THR B 156 -22.213 8.468 2.388 1.00 0.00

ATOM 3149 HB THR B 156 -22.960 7.708 2.622 1.00 0.00

ATOM 3150 OG1 THR B 156 -21.191 7.795 1.671 1.00 0.00

ATOM 3151 HG1 THR B 156 -20.482 8.418 1.516 1.00 0.00

ATOM 3152 CG2 THR B 156 -22.923 9.519 1.512 1.00 0.00

ATOM 3153 HG21 THR B 156 -22.937 10.512 1.963 1.00 0.00

ATOM 3154 HG22 THR B 156 -22.459 9.607 0.529 1.00 0.00

ATOM 3155 HG23 THR B 156 -23.956 9.216 1.338 1.00 0.00

ATOM 3156 N THR B 157 -23.550 10.269 4.550 1.00 0.00

ATOM 3157 HN THR B 157 -23.384 10.851 3.754 1.00 0.00

ATOM 3158 CA THR B 157 -24.569 10.609 5.536 1.00 0.00

ATOM 3159 HA THR B 157 -24.125 10.451 6.524 1.00 0.00

ATOM 3160 C THR B 157 -25.786 9.649 5.477 1.00 0.00

ATOM 3161 O THR B 157 -26.627 9.710 6.362 1.00 0.00

ATOM 3162 CB THR B 157 -24.912 12.112 5.348 1.00 0.00

ATOM 3163 HB THR B 157 -23.965 12.658 5.408 1.00 0.00

ATOM 3164 OG1 THR B 157 -25.737 12.672 6.357 1.00 0.00

ATOM 3165 HG1 THR B 157 -25.537 12.251 7.200 1.00 0.00

ATOM 3166 CG2 THR B 157 -25.488 12.420 3.949 1.00 0.00

ATOM 3167 HG21 THR B 157 -25.537 11.542 3.304 1.00 0.00

ATOM 3168 HG22 THR B 157 -26.495 12.831 4.008 1.00 0.00

ATOM 3169 HG23 THR B 157 -24.877 13.160 3.433 1.00 0.00

ATOM 3170 N SER B 158 -25.829 8.775 4.432 1.00 0.00

ATOM 3171 HN SER B 158 -25.105 8.724 3.744 1.00 0.00

ATOM 3172 CA SER B 158 -26.881 7.744 4.358 1.00 0.00

ATOM 3173 HA SER B 158 -27.729 8.043 4.979 1.00 0.00

ATOM 3174 C SER B 158 -26.383 6.394 4.965 1.00 0.00

ATOM 3175 O SER B 158 -27.133 5.431 5.020 1.00 0.00

ATOM 3176 CB SER B 158 -27.405 7.615 2.900 1.00 0.00

ATOM 3177 HB1 SER B 158 -28.107 8.433 2.693 1.00 0.00

ATOM 3178 HB2 SER B 158 -27.998 6.683 2.801 1.00 0.00

ATOM 3179 OG SER B 158 -26.333 7.682 1.959 1.00 0.00

ATOM 3180 HG SER B 158 -25.722 6.988 2.276 1.00 0.00

ATOM 3181 N GLY B 159 -25.096 6.367 5.414 1.00 0.00

ATOM 3182 HN GLY B 159 -24.492 7.166 5.390 1.00 0.00

ATOM 3183 CA GLY B 159 -24.615 5.209 6.189 1.00 0.00

ATOM 3184 HA1 GLY B 159 -25.453 4.662 6.625 1.00 0.00

ATOM 3185 HA2 GLY B 159 -24.008 5.613 6.999 1.00 0.00

ATOM 3186 C GLY B 159 -23.759 4.221 5.382 1.00 0.00

ATOM 3187 O GLY B 159 -23.235 3.241 5.896 1.00 0.00

ATOM 3188 N THR B 160 -23.620 4.535 4.074 1.00 0.00

ATOM 3189 HN THR B 160 -23.960 5.404 3.744 1.00 0.00

ATOM 3190 CA THR B 160 -22.778 3.671 3.241 1.00 0.00

ATOM 3191 HA THR B 160 -22.920 2.645 3.584 1.00 0.00

ATOM 3192 C THR B 160 -21.283 4.043 3.445 1.00 0.00

ATOM 3193 O THR B 160 -20.881 5.210 3.417 1.00 0.00

ATOM 3194 CB THR B 160 -23.256 3.648 1.775 1.00 0.00

ATOM 3195 HB THR B 160 -24.304 3.313 1.789 1.00 0.00

ATOM 3196 OG1 THR B 160 -22.466 2.703 1.066 1.00 0.00

ATOM 3197 HG1 THR B 160 -22.856 2.493 0.215 1.00 0.00

ATOM 3198 CG2 THR B 160 -23.209 5.010 1.079 1.00 0.00

ATOM 3199 HG21 THR B 160 -22.646 5.744 1.649 1.00 0.00

ATOM 3200 HG22 THR B 160 -22.703 4.912 0.124 1.00 0.00

ATOM 3201 HG23 THR B 160 -24.215 5.381 0.873 1.00 0.00

ATOM 3202 N TYR B 161 -20.519 2.971 3.686 1.00 0.00

ATOM 3203 HN TYR B 161 -20.983 2.088 3.702 1.00 0.00

ATOM 3204 CA TYR B 161 -19.070 3.005 3.673 1.00 0.00

ATOM 3205 HA TYR B 161 -18.741 3.924 4.152 1.00 0.00

ATOM 3206 C TYR B 161 -18.593 2.940 2.214 1.00 0.00

ATOM 3207 O TYR B 161 -19.136 2.194 1.412 1.00 0.00

ATOM 3208 CB TYR B 161 -18.590 1.766 4.430 1.00 0.00

ATOM 3209 HB1 TYR B 161 -19.069 0.898 3.976 1.00 0.00

ATOM 3210 HB2 TYR B 161 -18.896 1.793 5.474 1.00 0.00

ATOM 3211 CG TYR B 161 -17.104 1.585 4.351 1.00 0.00

ATOM 3212 CD1 TYR B 161 -16.240 2.490 4.948 1.00 0.00

ATOM 3213 HD1 TYR B 161 -16.629 3.338 5.485 1.00 0.00

ATOM 3214 CE1 TYR B 161 -14.870 2.317 4.831 1.00 0.00

ATOM 3215 HE1 TYR B 161 -14.208 3.029 5.278 1.00 0.00

ATOM 3216 CZ TYR B 161 -14.355 1.246 4.120 1.00 0.00

ATOM 3217 OH TYR B 161 -12.998 1.020 4.004 1.00 0.00

ATOM 3218 HH TYR B 161 -12.449 1.333 4.722 1.00 0.00

ATOM 3219 CE2 TYR B 161 -15.215 0.357 3.511 1.00 0.00

ATOM 3220 HE2 TYR B 161 -14.817 -0.442 2.908 1.00 0.00

ATOM 3221 CD2 TYR B 161 -16.581 0.515 3.643 1.00 0.00

ATOM 3222 HD2 TYR B 161 -17.248 -0.194 3.186 1.00 0.00

ATOM 3223 N VAL B 162 -17.548 3.734 1.927 1.00 0.00

ATOM 3224 HN VAL B 162 -17.137 4.251 2.678 1.00 0.00

ATOM 3225 CA VAL B 162 -16.961 3.878 0.597 1.00 0.00

ATOM 3226 HA VAL B 162 -17.177 2.949 0.071 1.00 0.00

ATOM 3227 C VAL B 162 -15.421 4.062 0.709 1.00 0.00

ATOM 3228 O VAL B 162 -14.932 5.019 1.324 1.00 0.00

ATOM 3229 CB VAL B 162 -17.616 5.066 -0.149 1.00 0.00

ATOM 3230 HB VAL B 162 -16.940 5.374 -0.950 1.00 0.00

ATOM 3231 CG1 VAL B 162 -18.917 4.652 -0.861 1.00 0.00

ATOM 3232 HG11 VAL B 162 -18.990 3.566 -0.961 1.00 0.00

ATOM 3233 HG12 VAL B 162 -19.817 4.982 -0.334 1.00 0.00

ATOM 3234 HG13 VAL B 162 -18.936 5.056 -1.875 1.00 0.00

ATOM 3235 CG2 VAL B 162 -17.827 6.307 0.740 1.00 0.00

ATOM 3236 HG21 VAL B 162 -17.550 6.139 1.772 1.00 0.00

ATOM 3237 HG22 VAL B 162 -17.195 7.126 0.411 1.00 0.00

ATOM 3238 HG23 VAL B 162 -18.861 6.645 0.741 1.00 0.00

ATOM 3239 N SER B 163 -14.742 3.071 0.089 1.00 0.00

ATOM 3240 HN SER B 163 -15.295 2.476 -0.492 1.00 0.00

ATOM 3241 CA SER B 163 -13.299 2.863 0.098 1.00 0.00

ATOM 3242 HA SER B 163 -12.899 3.481 0.899 1.00 0.00

ATOM 3243 C SER B 163 -12.656 3.338 -1.225 1.00 0.00

ATOM 3244 O SER B 163 -12.982 2.944 -2.350 1.00 0.00

ATOM 3245 CB SER B 163 -12.985 1.373 0.374 1.00 0.00

ATOM 3246 HB1 SER B 163 -13.383 0.758 -0.435 1.00 0.00

ATOM 3247 HB2 SER B 163 -13.493 1.012 1.276 1.00 0.00

ATOM 3248 OG SER B 163 -11.598 1.092 0.490 1.00 0.00

ATOM 3249 HG SER B 163 -11.203 1.746 1.069 1.00 0.00

ATOM 3250 N ALA B 164 -11.681 4.238 -0.964 1.00 0.00

ATOM 3251 HN ALA B 164 -11.455 4.387 -0.003 1.00 0.00

ATOM 3252 CA ALA B 164 -10.802 4.790 -1.983 1.00 0.00

ATOM 3253 HA ALA B 164 -11.452 5.191 -2.757 1.00 0.00

ATOM 3254 C ALA B 164 -9.877 3.694 -2.550 1.00 0.00

ATOM 3255 O ALA B 164 -9.138 3.047 -1.805 1.00 0.00

ATOM 3256 CB ALA B 164 -9.942 5.924 -1.385 1.00 0.00

ATOM 3257 HB1 ALA B 164 -10.383 6.338 -0.473 1.00 0.00

ATOM 3258 HB2 ALA B 164 -8.926 5.590 -1.149 1.00 0.00

ATOM 3259 HB3 ALA B 164 -9.874 6.750 -2.100 1.00 0.00

ATOM 3260 N ILE B 165 -9.937 3.584 -3.890 1.00 0.00

ATOM 3261 HN ILE B 165 -10.524 4.200 -4.419 1.00 0.00

ATOM 3262 CA ILE B 165 -8.972 2.718 -4.548 1.00 0.00

ATOM 3263 HA ILE B 165 -9.056 1.735 -4.082 1.00 0.00

ATOM 3264 C ILE B 165 -7.516 3.237 -4.335 1.00 0.00

ATOM 3265 O ILE B 165 -6.899 3.812 -5.230 1.00 0.00

ATOM 3266 CB ILE B 165 -9.360 2.640 -6.043 1.00 0.00

ATOM 3267 HB ILE B 165 -9.358 3.662 -6.435 1.00 0.00

ATOM 3268 CG2 ILE B 165 -8.333 1.820 -6.852 1.00 0.00

ATOM 3269 HG21 ILE B 165 -7.442 1.560 -6.283 1.00 0.00

ATOM 3270 HG22 ILE B 165 -8.773 0.891 -7.217 1.00 0.00

ATOM 3271 HG23 ILE B 165 -7.997 2.393 -7.719 1.00 0.00

ATOM 3272 CG1 ILE B 165 -10.802 2.096 -6.225 1.00 0.00

ATOM 3273 HG11 ILE B 165 -11.518 2.758 -5.735 1.00 0.00

ATOM 3274 HG12 ILE B 165 -10.890 1.135 -5.721 1.00 0.00

ATOM 3275 CD1 ILE B 165 -11.242 1.919 -7.690 1.00 0.00

ATOM 3276 HD11 ILE B 165 -10.518 2.352 -8.382 1.00 0.00

ATOM 3277 HD12 ILE B 165 -11.351 0.860 -7.943 1.00 0.00

ATOM 3278 HD13 ILE B 165 -12.207 2.390 -7.888 1.00 0.00

ATOM 3279 N ALA B 166 -6.959 2.935 -3.145 1.00 0.00

ATOM 3280 HN ALA B 166 -7.483 2.303 -2.555 1.00 0.00

ATOM 3281 CA ALA B 166 -5.761 3.652 -2.714 1.00 0.00

ATOM 3282 HA ALA B 166 -5.915 4.699 -2.971 1.00 0.00

ATOM 3283 C ALA B 166 -4.504 3.116 -3.446 1.00 0.00

ATOM 3284 O ALA B 166 -4.135 1.956 -3.238 1.00 0.00

ATOM 3285 CB ALA B 166 -5.666 3.540 -1.185 1.00 0.00

ATOM 3286 HB1 ALA B 166 -6.604 3.201 -0.738 1.00 0.00

ATOM 3287 HB2 ALA B 166 -4.886 2.849 -0.876 1.00 0.00

ATOM 3288 HB3 ALA B 166 -5.448 4.513 -0.741 1.00 0.00

ATOM 3289 N GLN B 167 -3.914 3.999 -4.288 1.00 0.00

ATOM 3290 HN GLN B 167 -4.223 4.942 -4.272 1.00 0.00

ATOM 3291 CA GLN B 167 -2.860 3.674 -5.252 1.00 0.00

ATOM 3292 HA GLN B 167 -2.351 2.794 -4.867 1.00 0.00

ATOM 3293 C GLN B 167 -1.883 4.854 -5.294 1.00 0.00

ATOM 3294 O GLN B 167 -2.282 5.983 -5.011 1.00 0.00

ATOM 3295 CB GLN B 167 -3.513 3.479 -6.647 1.00 0.00

ATOM 3296 HB1 GLN B 167 -4.013 4.414 -6.908 1.00 0.00

ATOM 3297 HB2 GLN B 167 -4.308 2.741 -6.556 1.00 0.00

ATOM 3298 CG GLN B 167 -2.599 3.090 -7.838 1.00 0.00

ATOM 3299 HG1 GLN B 167 -1.821 3.835 -7.984 1.00 0.00

ATOM 3300 HG2 GLN B 167 -3.176 3.062 -8.764 1.00 0.00

ATOM 3301 CD GLN B 167 -1.941 1.727 -7.618 1.00 0.00

ATOM 3302 OE1 GLN B 167 -1.478 1.454 -6.519 1.00 0.00

ATOM 3303 NE2 GLN B 167 -1.983 0.850 -8.628 1.00 0.00

ATOM 3304 HE21 GLN B 167 -1.857 -0.150 -8.595 1.00 0.00

ATOM 3305 HE22 GLN B 167 -2.171 1.255 -9.526 1.00 0.00

ATOM 3306 N ALA B 168 -0.620 4.539 -5.674 1.00 0.00

ATOM 3307 HN ALA B 168 -0.441 3.581 -5.905 1.00 0.00

ATOM 3308 CA ALA B 168 0.444 5.528 -5.866 1.00 0.00

ATOM 3309 HA ALA B 168 -0.034 6.506 -5.953 1.00 0.00

ATOM 3310 C ALA B 168 1.181 5.218 -7.183 1.00 0.00

ATOM 3311 O ALA B 168 1.164 4.107 -7.691 1.00 0.00

ATOM 3312 CB ALA B 168 1.462 5.525 -4.702 1.00 0.00

ATOM 3313 HB1 ALA B 168 1.109 4.919 -3.870 1.00 0.00

ATOM 3314 HB2 ALA B 168 2.441 5.130 -4.987 1.00 0.00

ATOM 3315 HB3 ALA B 168 1.622 6.528 -4.304 1.00 0.00

ATOM 3316 N LYS B 169 1.850 6.269 -7.685 1.00 0.00

ATOM 3317 HN LYS B 169 1.850 7.124 -7.173 1.00 0.00

ATOM 3318 CA LYS B 169 2.689 6.072 -8.873 1.00 0.00

ATOM 3319 HA LYS B 169 2.085 5.570 -9.630 1.00 0.00

ATOM 3320 C LYS B 169 3.921 5.215 -8.515 1.00 0.00

ATOM 3321 O LYS B 169 4.581 5.508 -7.536 1.00 0.00

ATOM 3322 CB LYS B 169 3.201 7.446 -9.362 1.00 0.00

ATOM 3323 HB1 LYS B 169 3.742 7.914 -8.536 1.00 0.00

ATOM 3324 HB2 LYS B 169 2.362 8.107 -9.588 1.00 0.00

ATOM 3325 CG LYS B 169 4.140 7.377 -10.588 1.00 0.00

ATOM 3326 HG1 LYS B 169 3.546 7.270 -11.496 1.00 0.00

ATOM 3327 HG2 LYS B 169 4.771 6.496 -10.531 1.00 0.00

ATOM 3328 CD LYS B 169 5.081 8.586 -10.691 1.00 0.00

ATOM 3329 HD1 LYS B 169 5.493 8.811 -9.705 1.00 0.00

ATOM 3330 HD2 LYS B 169 4.507 9.464 -10.986 1.00 0.00

ATOM 3331 CE LYS B 169 6.255 8.383 -11.663 1.00 0.00

ATOM 3332 HE1 LYS B 169 6.844 9.301 -11.732 1.00 0.00

ATOM 3333 HE2 LYS B 169 5.907 8.138 -12.666 1.00 0.00

ATOM 3334 NZ LYS B 169 7.181 7.342 -11.250 1.00 0.00

ATOM 3335 HZ1 LYS B 169 6.975 6.946 -10.306 1.00 0.00

ATOM 3336 HZ2 LYS B 169 7.220 6.524 -11.886 1.00 0.00

ATOM 3337 HZ3 LYS B 169 8.140 7.720 -11.114 1.00 0.00

ATOM 3338 N ALA B 170 4.234 4.253 -9.404 1.00 0.00

ATOM 3339 HN ALA B 170 3.508 3.949 -10.010 1.00 0.00

ATOM 3340 CA ALA B 170 5.488 3.516 -9.421 1.00 0.00

ATOM 3341 HA ALA B 170 5.901 3.533 -8.412 1.00 0.00

ATOM 3342 C ALA B 170 6.518 4.176 -10.369 1.00 0.00

ATOM 3343 O ALA B 170 7.087 5.221 -10.099 1.00 0.00

ATOM 3344 CB ALA B 170 5.171 2.062 -9.803 1.00 0.00

ATOM 3345 HB1 ALA B 170 4.270 2.007 -10.430 1.00 0.00

ATOM 3346 HB2 ALA B 170 6.002 1.538 -10.292 1.00 0.00

ATOM 3347 HB3 ALA B 170 4.966 1.502 -8.890 1.00 0.00

ATOM 3348 N SER B 171 6.775 3.482 -11.479 1.00 0.00

ATOM 3349 HN SER B 171 6.240 2.636 -11.583 1.00 0.00

ATOM 3350 CA SER B 171 8.105 3.556 -12.080 1.00 0.00

ATOM 3351 HA SER B 171 7.972 2.913 -12.948 1.00 0.00

ATOM 3352 C SER B 171 9.118 2.849 -11.144 1.00 0.00

ATOM 3353 O SER B 171 9.164 1.622 -11.149 1.00 0.00

ATOM 3354 CB SER B 171 8.610 4.915 -12.637 1.00 0.00

ATOM 3355 HB1 SER B 171 9.431 4.711 -13.337 1.00 0.00

ATOM 3356 HB2 SER B 171 9.089 5.522 -11.874 1.00 0.00

ATOM 3357 OG SER B 171 7.582 5.711 -13.241 1.00 0.00

ATOM 3358 HG SER B 171 7.761 5.956 -14.154 1.00 0.00

ATOM 3359 N GLN B 172 9.923 3.611 -10.369 1.00 0.00

ATOM 3360 HN GLN B 172 9.810 4.605 -10.313 1.00 0.00

ATOM 3361 CA GLN B 172 10.829 3.018 -9.383 1.00 0.00

ATOM 3362 HA GLN B 172 10.304 2.175 -8.929 1.00 0.00

ATOM 3363 C GLN B 172 11.105 4.037 -8.254 1.00 0.00

ATOM 3364 O GLN B 172 10.571 3.921 -7.153 1.00 0.00

ATOM 3365 CB GLN B 172 12.127 2.528 -10.046 1.00 0.00

ATOM 3366 HB1 GLN B 172 12.657 3.379 -10.479 1.00 0.00

ATOM 3367 HB2 GLN B 172 11.870 1.887 -10.889 1.00 0.00

ATOM 3368 CG GLN B 172 13.029 1.740 -9.078 1.00 0.00

ATOM 3369 HG1 GLN B 172 13.248 0.757 -9.491 1.00 0.00

ATOM 3370 HG2 GLN B 172 12.551 1.604 -8.109 1.00 0.00

ATOM 3371 CD GLN B 172 14.350 2.471 -8.905 1.00 0.00

ATOM 3372 OE1 GLN B 172 15.028 2.759 -9.879 1.00 0.00

ATOM 3373 NE2 GLN B 172 14.646 2.858 -7.667 1.00 0.00

ATOM 3374 HE21 GLN B 172 15.387 3.529 -7.662 1.00 0.00

ATOM 3375 HE22 GLN B 172 14.267 2.565 -6.764 1.00 0.00

ATOM 3376 N GLU B 173 11.976 5.009 -8.608 1.00 0.00

ATOM 3377 HN GLU B 173 12.097 5.144 -9.592 1.00 0.00

ATOM 3378 CA GLU B 173 12.473 6.065 -7.711 1.00 0.00

ATOM 3379 HA GLU B 173 13.227 6.563 -8.322 1.00 0.00

ATOM 3380 C GLU B 173 13.244 5.538 -6.449 1.00 0.00

ATOM 3381 O GLU B 173 14.473 5.608 -6.403 1.00 0.00

ATOM 3382 CB GLU B 173 11.460 7.205 -7.432 1.00 0.00

ATOM 3383 HB1 GLU B 173 11.827 8.092 -7.952 1.00 0.00

ATOM 3384 HB2 GLU B 173 11.506 7.470 -6.374 1.00 0.00

ATOM 3385 CG GLU B 173 9.983 6.998 -7.838 1.00 0.00

ATOM 3386 HG1 GLU B 173 9.360 7.673 -7.245 1.00 0.00

ATOM 3387 HG2 GLU B 173 9.629 6.006 -7.592 1.00 0.00

ATOM 3388 CD GLU B 173 9.678 7.266 -9.313 1.00 0.00

ATOM 3389 OE1 GLU B 173 10.229 6.605 -10.192 1.00 0.00

ATOM 3390 OE2 GLU B 173 8.863 8.135 -9.591 1.00 0.00

ATOM 3391 N GLU B 174 12.445 5.051 -5.476 1.00 0.00

ATOM 3392 HN GLU B 174 11.480 4.889 -5.688 1.00 0.00

ATOM 3393 CA GLU B 174 12.849 4.501 -4.170 1.00 0.00

ATOM 3394 HA GLU B 174 11.881 4.345 -3.692 1.00 0.00

ATOM 3395 C GLU B 174 13.625 5.538 -3.290 1.00 0.00

ATOM 3396 O GLU B 174 13.770 6.681 -3.704 1.00 0.00

ATOM 3397 CB GLU B 174 13.484 3.103 -4.365 1.00 0.00

ATOM 3398 HB1 GLU B 174 13.128 2.701 -5.311 1.00 0.00

ATOM 3399 HB2 GLU B 174 13.093 2.405 -3.628 1.00 0.00

ATOM 3400 CG GLU B 174 15.015 3.038 -4.343 1.00 0.00

ATOM 3401 HG1 GLU B 174 15.412 3.308 -3.363 1.00 0.00

ATOM 3402 HG2 GLU B 174 15.426 3.748 -5.059 1.00 0.00

ATOM 3403 CD GLU B 174 15.499 1.630 -4.690 1.00 0.00

ATOM 3404 OE1 GLU B 174 16.301 1.056 -3.964 1.00 0.00

ATOM 3405 OE2 GLU B 174 15.141 1.111 -5.738 1.00 0.00

ATOM 3406 N PRO B 175 14.055 5.129 -2.048 1.00 0.00

ATOM 3407 CA PRO B 175 14.879 5.975 -1.151 1.00 0.00

ATOM 3408 HA PRO B 175 14.651 7.033 -1.297 1.00 0.00

ATOM 3409 C PRO B 175 16.441 5.793 -1.192 1.00 0.00

ATOM 3410 O PRO B 175 16.968 4.709 -0.944 1.00 0.00

ATOM 3411 CB PRO B 175 14.375 5.517 0.225 1.00 0.00

ATOM 3412 HB1 PRO B 175 13.458 6.057 0.457 1.00 0.00

ATOM 3413 HB2 PRO B 175 15.092 5.685 1.025 1.00 0.00

ATOM 3414 CG PRO B 175 14.014 4.039 0.076 1.00 0.00

ATOM 3415 HG1 PRO B 175 13.263 3.701 0.797 1.00 0.00

ATOM 3416 HG2 PRO B 175 14.901 3.422 0.225 1.00 0.00

ATOM 3417 CD PRO B 175 13.554 3.919 -1.377 1.00 0.00

ATOM 3418 HD1 PRO B 175 13.960 3.011 -1.819 1.00 0.00

ATOM 3419 HD2 PRO B 175 12.464 3.902 -1.426 1.00 0.00

ATOM 3420 N LEU B 176 17.173 6.898 -1.473 1.00 0.00

ATOM 3421 HN LEU B 176 16.746 7.781 -1.677 1.00 0.00

ATOM 3422 CA LEU B 176 18.638 6.841 -1.476 1.00 0.00

ATOM 3423 HA LEU B 176 18.951 6.027 -0.822 1.00 0.00

ATOM 3424 C LEU B 176 19.229 8.189 -0.969 1.00 0.00

ATOM 3425 O LEU B 176 19.679 8.991 -1.776 1.00 0.00

ATOM 3426 CB LEU B 176 19.104 6.531 -2.915 1.00 0.00

ATOM 3427 HB1 LEU B 176 19.099 7.440 -3.518 1.00 0.00

ATOM 3428 HB2 LEU B 176 18.365 5.877 -3.378 1.00 0.00

ATOM 3429 CG LEU B 176 20.468 5.824 -3.055 1.00 0.00

ATOM 3430 HG LEU B 176 20.654 5.793 -4.129 1.00 0.00

ATOM 3431 CD1 LEU B 176 21.656 6.589 -2.439 1.00 0.00

ATOM 3432 HD11 LEU B 176 21.521 7.679 -2.464 1.00 0.00

ATOM 3433 HD12 LEU B 176 21.824 6.293 -1.394 1.00 0.00

ATOM 3434 HD13 LEU B 176 22.581 6.382 -2.991 1.00 0.00

ATOM 3435 CD2 LEU B 176 20.409 4.360 -2.577 1.00 0.00

ATOM 3436 HD21 LEU B 176 19.385 4.057 -2.352 1.00 0.00

ATOM 3437 HD22 LEU B 176 20.798 3.692 -3.342 1.00 0.00

ATOM 3438 HD23 LEU B 176 20.993 4.219 -1.666 1.00 0.00

ATOM 3439 N PRO B 177 19.211 8.397 0.371 1.00 0.00

ATOM 3440 CA PRO B 177 19.908 9.537 0.974 1.00 0.00

ATOM 3441 HA PRO B 177 19.727 10.462 0.423 1.00 0.00

ATOM 3442 C PRO B 177 21.416 9.206 0.961 1.00 0.00

ATOM 3443 O PRO B 177 21.781 8.106 0.547 1.00 0.00

ATOM 3444 CB PRO B 177 19.345 9.558 2.404 1.00 0.00

ATOM 3445 HB1 PRO B 177 18.448 10.176 2.425 1.00 0.00

ATOM 3446 HB2 PRO B 177 20.057 9.978 3.118 1.00 0.00

ATOM 3447 CG PRO B 177 18.974 8.104 2.718 1.00 0.00

ATOM 3448 HG1 PRO B 177 18.160 8.008 3.441 1.00 0.00

ATOM 3449 HG2 PRO B 177 19.856 7.622 3.145 1.00 0.00

ATOM 3450 CD PRO B 177 18.648 7.479 1.360 1.00 0.00

ATOM 3451 HD1 PRO B 177 19.119 6.500 1.272 1.00 0.00

ATOM 3452 HD2 PRO B 177 17.570 7.392 1.231 1.00 0.00

ATOM 3453 N GLU B 178 22.245 10.146 1.437 1.00 0.00

ATOM 3454 HN GLU B 178 21.959 11.036 1.787 1.00 0.00

ATOM 3455 CA GLU B 178 23.618 9.790 1.764 1.00 0.00

ATOM 3456 HA GLU B 178 24.005 9.155 0.964 1.00 0.00

ATOM 3457 C GLU B 178 23.560 8.972 3.067 1.00 0.00

ATOM 3458 O GLU B 178 22.667 9.187 3.887 1.00 0.00

ATOM 3459 CB GLU B 178 24.460 11.075 1.868 1.00 0.00

ATOM 3460 HB1 GLU B 178 24.428 11.580 0.900 1.00 0.00

ATOM 3461 HB2 GLU B 178 25.503 10.816 2.051 1.00 0.00

ATOM 3462 CG GLU B 178 24.023 12.089 2.941 1.00 0.00

ATOM 3463 HG1 GLU B 178 24.134 11.670 3.942 1.00 0.00

ATOM 3464 HG2 GLU B 178 22.984 12.388 2.805 1.00 0.00

ATOM 3465 CD GLU B 178 24.908 13.338 2.844 1.00 0.00

ATOM 3466 OE1 GLU B 178 24.391 14.406 2.508 1.00 0.00

ATOM 3467 OE2 GLU B 178 26.109 13.218 3.082 1.00 0.00

ATOM 3468 N ILE B 179 24.489 7.979 3.181 1.00 0.00

ATOM 3469 HN ILE B 179 25.176 7.874 2.450 1.00 0.00

ATOM 3470 CA ILE B 179 24.623 7.200 4.422 1.00 0.00

ATOM 3471 HA ILE B 179 23.899 7.577 5.146 1.00 0.00

ATOM 3472 C ILE B 179 26.027 7.481 4.999 1.00 0.00

ATOM 3473 O ILE B 179 27.023 6.989 4.472 1.00 0.00

ATOM 3474 CB ILE B 179 24.308 5.694 4.178 1.00 0.00

ATOM 3475 HB ILE B 179 23.226 5.632 4.050 1.00 0.00

ATOM 3476 CG1 ILE B 179 24.637 4.800 5.386 1.00 0.00

ATOM 3477 HG11 ILE B 179 24.248 5.260 6.295 1.00 0.00

ATOM 3478 HG12 ILE B 179 25.718 4.724 5.516 1.00 0.00

ATOM 3479 CG2 ILE B 179 24.923 5.113 2.895 1.00 0.00

ATOM 3480 HG21 ILE B 179 25.679 5.774 2.491 1.00 0.00

ATOM 3481 HG22 ILE B 179 25.406 4.150 3.065 1.00 0.00

ATOM 3482 HG23 ILE B 179 24.163 4.966 2.124 1.00 0.00

ATOM 3483 CD1 ILE B 179 24.048 3.390 5.259 1.00 0.00

ATOM 3484 HD11 ILE B 179 23.492 3.275 4.328 1.00 0.00

ATOM 3485 HD12 ILE B 179 24.826 2.626 5.284 1.00 0.00

ATOM 3486 HD13 ILE B 179 23.356 3.181 6.076 1.00 0.00

ATOM 3487 N GLU B 180 26.006 8.299 6.075 1.00 0.00

ATOM 3488 HN GLU B 180 25.128 8.508 6.503 1.00 0.00

ATOM 3489 CA GLU B 180 27.086 9.150 6.581 1.00 0.00

ATOM 3490 HA GLU B 180 27.183 9.931 5.820 1.00 0.00

ATOM 3491 C GLU B 180 28.479 8.487 6.676 1.00 0.00

ATOM 3492 O GLU B 180 29.498 9.171 6.574 1.00 0.00

ATOM 3493 CB GLU B 180 26.654 9.836 7.906 1.00 0.00

ATOM 3494 HB1 GLU B 180 25.752 10.419 7.710 1.00 0.00

ATOM 3495 HB2 GLU B 180 27.428 10.550 8.189 1.00 0.00

ATOM 3496 CG GLU B 180 26.373 8.921 9.120 1.00 0.00

ATOM 3497 HG1 GLU B 180 27.267 8.350 9.368 1.00 0.00

ATOM 3498 HG2 GLU B 180 25.576 8.218 8.877 1.00 0.00

ATOM 3499 CD GLU B 180 25.976 9.719 10.393 1.00 0.00

ATOM 3500 OE1 GLU B 180 26.804 10.493 10.887 1.00 0.00

ATOM 3501 OE2 GLU B 180 24.857 9.543 10.894 1.00 0.00

ATOM 3502 N ASP B 181 28.491 7.137 6.814 1.00 0.00

ATOM 3503 HN ASP B 181 27.600 6.688 6.889 1.00 0.00

ATOM 3504 CA ASP B 181 29.754 6.372 6.746 1.00 0.00

ATOM 3505 HA ASP B 181 30.258 6.474 7.707 1.00 0.00

ATOM 3506 C ASP B 181 30.692 6.888 5.623 1.00 0.00

ATOM 3507 O ASP B 181 31.913 6.959 5.765 1.00 0.00

ATOM 3508 CB ASP B 181 29.544 4.892 6.308 1.00 0.00

ATOM 3509 HB1 ASP B 181 30.515 4.486 6.001 1.00 0.00

ATOM 3510 HB2 ASP B 181 28.921 4.952 5.417 1.00 0.00

ATOM 3511 CG ASP B 181 28.976 3.833 7.300 1.00 0.00

ATOM 3512 OD1 ASP B 181 29.343 3.807 8.457 1.00 0.00

ATOM 3513 OD2 ASP B 181 28.196 2.968 6.900 1.00 0.00

ATOM 3514 N GLU B 182 30.064 7.104 4.450 1.00 0.00

ATOM 3515 HN GLU B 182 29.063 7.157 4.492 1.00 0.00

ATOM 3516 CA GLU B 182 30.877 7.152 3.242 1.00 0.00

ATOM 3517 HA GLU B 182 31.625 6.367 3.390 1.00 0.00

ATOM 3518 C GLU B 182 31.735 8.431 3.127 1.00 0.00

ATOM 3519 O GLU B 182 32.757 8.408 2.444 1.00 0.00

ATOM 3520 CB GLU B 182 30.109 6.669 1.998 1.00 0.00

ATOM 3521 HB1 GLU B 182 29.934 5.626 2.207 1.00 0.00

ATOM 3522 HB2 GLU B 182 30.734 6.716 1.109 1.00 0.00

ATOM 3523 CG GLU B 182 28.746 7.284 1.662 1.00 0.00

ATOM 3524 HG1 GLU B 182 28.892 8.207 1.099 1.00 0.00

ATOM 3525 HG2 GLU B 182 28.218 7.556 2.568 1.00 0.00

ATOM 3526 CD GLU B 182 27.820 6.354 0.843 1.00 0.00

ATOM 3527 OE1 GLU B 182 26.675 6.731 0.646 1.00 0.00

ATOM 3528 OE2 GLU B 182 28.201 5.240 0.443 1.00 0.00

ATOM 3529 N VAL B 183 31.363 9.484 3.894 1.00 0.00

ATOM 3530 HN VAL B 183 30.487 9.470 4.387 1.00 0.00

ATOM 3531 CA VAL B 183 32.230 10.671 3.954 1.00 0.00

ATOM 3532 HA VAL B 183 32.612 10.811 2.942 1.00 0.00

ATOM 3533 C VAL B 183 33.488 10.445 4.866 1.00 0.00

ATOM 3534 O VAL B 183 34.385 11.285 5.008 1.00 0.00

ATOM 3535 CB VAL B 183 31.403 11.942 4.290 1.00 0.00

ATOM 3536 HB VAL B 183 32.116 12.765 4.347 1.00 0.00

ATOM 3537 CG1 VAL B 183 30.438 12.320 3.140 1.00 0.00

ATOM 3538 HG11 VAL B 183 30.601 11.714 2.247 1.00 0.00

ATOM 3539 HG12 VAL B 183 29.385 12.204 3.408 1.00 0.00

ATOM 3540 HG13 VAL B 183 30.568 13.364 2.847 1.00 0.00

ATOM 3541 CG2 VAL B 183 30.671 11.905 5.649 1.00 0.00

ATOM 3542 HG21 VAL B 183 31.024 11.101 6.295 1.00 0.00

ATOM 3543 HG22 VAL B 183 30.809 12.850 6.180 1.00 0.00

ATOM 3544 HG23 VAL B 183 29.590 11.770 5.535 1.00 0.00

ATOM 3545 N PHE B 184 33.549 9.246 5.485 1.00 0.00

ATOM 3546 HN PHE B 184 32.795 8.595 5.372 1.00 0.00

ATOM 3547 CA PHE B 184 34.667 8.942 6.370 1.00 0.00

ATOM 3548 HA PHE B 184 34.845 9.831 6.980 1.00 0.00

ATOM 3549 C PHE B 184 35.946 8.621 5.567 1.00 0.00

ATOM 3550 O PHE B 184 36.066 7.669 4.796 1.00 0.00

ATOM 3551 CB PHE B 184 34.370 7.760 7.301 1.00 0.00

ATOM 3552 HB1 PHE B 184 35.266 7.541 7.881 1.00 0.00

ATOM 3553 HB2 PHE B 184 34.158 6.877 6.699 1.00 0.00

ATOM 3554 CG PHE B 184 33.235 7.944 8.283 1.00 0.00

ATOM 3555 CD1 PHE B 184 32.928 6.890 9.139 1.00 0.00

ATOM 3556 HD1 PHE B 184 33.492 5.972 9.084 1.00 0.00

ATOM 3557 CE1 PHE B 184 31.883 6.987 10.049 1.00 0.00

ATOM 3558 HE1 PHE B 184 31.627 6.154 10.692 1.00 0.00

ATOM 3559 CZ PHE B 184 31.126 8.148 10.113 1.00 0.00

ATOM 3560 HZ PHE B 184 30.291 8.222 10.801 1.00 0.00

ATOM 3561 CE2 PHE B 184 31.420 9.208 9.270 1.00 0.00

ATOM 3562 HE2 PHE B 184 30.807 10.100 9.312 1.00 0.00

ATOM 3563 CD2 PHE B 184 32.470 9.109 8.366 1.00 0.00

ATOM 3564 HD2 PHE B 184 32.648 9.959 7.728 1.00 0.00

ATOM 3565 N LYS B 185 36.950 9.464 5.855 1.00 0.00

ATOM 3566 HN LYS B 185 36.686 10.230 6.441 1.00 0.00

ATOM 3567 CA LYS B 185 38.242 9.276 5.216 1.00 0.00

ATOM 3568 HA LYS B 185 38.061 9.390 4.146 1.00 0.00

ATOM 3569 C LYS B 185 38.836 7.874 5.488 1.00 0.00

ATOM 3570 O LYS B 185 39.032 7.460 6.624 1.00 0.00

ATOM 3571 CB LYS B 185 39.222 10.350 5.705 1.00 0.00

ATOM 3572 HB1 LYS B 185 40.213 10.127 5.312 1.00 0.00

ATOM 3573 HB2 LYS B 185 39.292 10.300 6.793 1.00 0.00

ATOM 3574 CG LYS B 185 38.809 11.761 5.270 1.00 0.00

ATOM 3575 HG1 LYS B 185 37.798 11.963 5.629 1.00 0.00

ATOM 3576 HG2 LYS B 185 38.755 11.802 4.180 1.00 0.00

ATOM 3577 CD LYS B 185 39.761 12.844 5.788 1.00 0.00

ATOM 3578 HD1 LYS B 185 40.771 12.651 5.418 1.00 0.00

ATOM 3579 HD2 LYS B 185 39.804 12.782 6.878 1.00 0.00

ATOM 3580 CE LYS B 185 39.310 14.251 5.371 1.00 0.00

ATOM 3581 HE1 LYS B 185 38.293 14.447 5.729 1.00 0.00

ATOM 3582 HE2 LYS B 185 39.285 14.343 4.280 1.00 0.00

ATOM 3583 NZ LYS B 185 40.201 15.289 5.895 1.00 0.00

ATOM 3584 HZ1 LYS B 185 40.898 14.874 6.547 1.00 0.00

ATOM 3585 HZ2 LYS B 185 40.701 15.736 5.100 1.00 0.00

ATOM 3586 HZ3 LYS B 185 39.653 16.012 6.406 1.00 0.00

ATOM 3587 N LYS B 186 39.195 7.246 4.352 1.00 0.00

ATOM 3588 HN LYS B 186 38.824 7.628 3.499 1.00 0.00

ATOM 3589 CA LYS B 186 39.892 5.959 4.264 1.00 0.00

ATOM 3590 HA LYS B 186 40.622 6.109 3.471 1.00 0.00

ATOM 3591 C LYS B 186 38.918 4.886 3.768 1.00 0.00

ATOM 3592 O LYS B 186 39.355 3.845 3.295 1.00 0.00

ATOM 3593 CB LYS B 186 40.661 5.488 5.531 1.00 0.00

ATOM 3594 HB1 LYS B 186 39.949 5.237 6.319 1.00 0.00

ATOM 3595 HB2 LYS B 186 41.265 6.318 5.898 1.00 0.00

ATOM 3596 CG LYS B 186 41.592 4.275 5.299 1.00 0.00

ATOM 3597 HG1 LYS B 186 42.269 4.492 4.470 1.00 0.00

ATOM 3598 HG2 LYS B 186 40.990 3.417 5.000 1.00 0.00

ATOM 3599 CD LYS B 186 42.417 3.892 6.539 1.00 0.00

ATOM 3600 HD1 LYS B 186 41.759 3.670 7.382 1.00 0.00

ATOM 3601 HD2 LYS B 186 43.018 4.754 6.831 1.00 0.00

ATOM 3602 CE LYS B 186 43.363 2.705 6.299 1.00 0.00

ATOM 3603 HE1 LYS B 186 44.060 2.613 7.134 1.00 0.00

ATOM 3604 HE2 LYS B 186 43.966 2.876 5.403 1.00 0.00

ATOM 3605 NZ LYS B 186 42.671 1.422 6.159 1.00 0.00

ATOM 3606 HZ1 LYS B 186 41.640 1.547 6.244 1.00 0.00

ATOM 3607 HZ2 LYS B 186 42.899 1.011 5.230 1.00 0.00

ATOM 3608 HZ3 LYS B 186 42.988 0.774 6.909 1.00 0.00

ATOM 3609 N GLY B 187 37.609 5.185 3.908 1.00 0.00

ATOM 3610 HN GLY B 187 37.257 6.073 4.226 1.00 0.00

ATOM 3611 CA GLY B 187 36.584 4.228 3.510 1.00 0.00

ATOM 3612 HA1 GLY B 187 36.925 3.208 3.708 1.00 0.00

ATOM 3613 HA2 GLY B 187 35.712 4.446 4.127 1.00 0.00

ATOM 3614 C GLY B 187 36.236 4.440 2.034 1.00 0.00

ATOM 3615 O GLY B 187 35.744 5.494 1.647 1.00 0.00

ATOM 3616 N SER B 188 36.586 3.400 1.248 1.00 0.00

ATOM 3617 HN SER B 188 37.049 2.585 1.614 1.00 0.00

ATOM 3618 CA SER B 188 36.403 3.427 -0.201 1.00 0.00

ATOM 3619 HA SER B 188 36.089 4.431 -0.491 1.00 0.00

ATOM 3620 C SER B 188 35.312 2.393 -0.587 1.00 0.00

ATOM 3621 O SER B 188 35.503 1.194 -0.420 1.00 0.00

ATOM 3622 CB SER B 188 37.790 3.081 -0.791 1.00 0.00

ATOM 3623 HB1 SER B 188 37.975 2.009 -0.674 1.00 0.00

ATOM 3624 HB2 SER B 188 38.542 3.590 -0.182 1.00 0.00

ATOM 3625 OG SER B 188 38.052 3.474 -2.132 1.00 0.00

ATOM 3626 HG SER B 188 37.213 3.721 -2.540 1.00 0.00

ATOM 3627 N ARG B 189 34.159 2.903 -1.078 1.00 0.00

ATOM 3628 HN ARG B 189 34.061 3.889 -1.235 1.00 0.00

ATOM 3629 CA ARG B 189 33.018 2.036 -1.405 1.00 0.00

ATOM 3630 HA ARG B 189 33.193 1.060 -0.952 1.00 0.00

ATOM 3631 C ARG B 189 32.858 1.841 -2.937 1.00 0.00

ATOM 3632 O ARG B 189 33.118 2.734 -3.728 1.00 0.00

ATOM 3633 CB ARG B 189 31.735 2.650 -0.816 1.00 0.00

ATOM 3634 HB1 ARG B 189 30.859 2.175 -1.260 1.00 0.00

ATOM 3635 HB2 ARG B 189 31.668 3.708 -1.085 1.00 0.00

ATOM 3636 CG ARG B 189 31.630 2.496 0.711 1.00 0.00

ATOM 3637 HG1 ARG B 189 32.372 3.130 1.202 1.00 0.00

ATOM 3638 HG2 ARG B 189 31.846 1.462 0.984 1.00 0.00

ATOM 3639 CD ARG B 189 30.231 2.881 1.214 1.00 0.00

ATOM 3640 HD1 ARG B 189 29.446 2.343 0.683 1.00 0.00

ATOM 3641 HD2 ARG B 189 30.081 3.948 1.076 1.00 0.00

ATOM 3642 NE ARG B 189 30.061 2.562 2.625 1.00 0.00

ATOM 3643 HE ARG B 189 30.706 1.971 3.102 1.00 0.00

ATOM 3644 CZ ARG B 189 29.070 3.078 3.386 1.00 0.00

ATOM 3645 NH1 ARG B 189 28.218 4.002 2.947 1.00 0.00

ATOM 3646 HH11 ARG B 189 28.314 4.373 2.012 1.00 0.00

ATOM 3647 HH12 ARG B 189 27.517 4.418 3.531 1.00 0.00

ATOM 3648 NH2 ARG B 189 28.998 2.631 4.616 1.00 0.00

ATOM 3649 HH21 ARG B 189 29.580 1.956 5.068 1.00 0.00

ATOM 3650 HH22 ARG B 189 28.340 3.053 5.256 1.00 0.00

ATOM 3651 N LEU B 190 32.385 0.621 -3.295 1.00 0.00

ATOM 3652 HN LEU B 190 32.243 -0.082 -2.603 1.00 0.00

ATOM 3653 CA LEU B 190 31.965 0.291 -4.663 1.00 0.00

ATOM 3654 HA LEU B 190 32.295 1.080 -5.340 1.00 0.00

ATOM 3655 C LEU B 190 30.433 0.172 -4.732 1.00 0.00

ATOM 3656 O LEU B 190 29.848 -0.839 -4.361 1.00 0.00

ATOM 3657 CB LEU B 190 32.515 -1.077 -5.110 1.00 0.00

ATOM 3658 HB1 LEU B 190 32.049 -1.358 -6.058 1.00 0.00

ATOM 3659 HB2 LEU B 190 32.207 -1.830 -4.384 1.00 0.00

ATOM 3660 CG LEU B 190 34.037 -1.172 -5.282 1.00 0.00

ATOM 3661 HG LEU B 190 34.504 -0.851 -4.349 1.00 0.00

ATOM 3662 CD1 LEU B 190 34.451 -2.642 -5.517 1.00 0.00

ATOM 3663 HD11 LEU B 190 33.609 -3.332 -5.435 1.00 0.00

ATOM 3664 HD12 LEU B 190 34.891 -2.806 -6.502 1.00 0.00

ATOM 3665 HD13 LEU B 190 35.194 -2.962 -4.786 1.00 0.00

ATOM 3666 CD2 LEU B 190 34.548 -0.239 -6.397 1.00 0.00

ATOM 3667 HD21 LEU B 190 33.739 0.282 -6.909 1.00 0.00

ATOM 3668 HD22 LEU B 190 35.219 0.521 -5.996 1.00 0.00

ATOM 3669 HD23 LEU B 190 35.104 -0.771 -7.169 1.00 0.00

ATOM 3670 N LEU B 191 29.816 1.231 -5.278 1.00 0.00

ATOM 3671 HN LEU B 191 30.437 1.956 -5.585 1.00 0.00

ATOM 3672 CA LEU B 191 28.367 1.262 -5.433 1.00 0.00

ATOM 3673 HA LEU B 191 27.940 0.950 -4.476 1.00 0.00

ATOM 3674 C LEU B 191 27.941 0.263 -6.538 1.00 0.00

ATOM 3675 O LEU B 191 27.924 0.562 -7.725 1.00 0.00

ATOM 3676 CB LEU B 191 27.976 2.713 -5.745 1.00 0.00

ATOM 3677 HB1 LEU B 191 28.290 2.950 -6.762 1.00 0.00

ATOM 3678 HB2 LEU B 191 28.529 3.387 -5.085 1.00 0.00

ATOM 3679 CG LEU B 191 26.474 3.022 -5.612 1.00 0.00

ATOM 3680 HG LEU B 191 25.925 2.299 -6.217 1.00 0.00

ATOM 3681 CD1 LEU B 191 25.981 2.884 -4.154 1.00 0.00

ATOM 3682 HD11 LEU B 191 26.784 2.616 -3.467 1.00 0.00

ATOM 3683 HD12 LEU B 191 25.573 3.821 -3.772 1.00 0.00

ATOM 3684 HD13 LEU B 191 25.199 2.132 -4.062 1.00 0.00

ATOM 3685 CD2 LEU B 191 26.168 4.414 -6.206 1.00 0.00

ATOM 3686 HD21 LEU B 191 27.046 4.872 -6.665 1.00 0.00

ATOM 3687 HD22 LEU B 191 25.402 4.357 -6.979 1.00 0.00

ATOM 3688 HD23 LEU B 191 25.813 5.121 -5.456 1.00 0.00

ATOM 3689 N THR B 192 27.670 -0.948 -6.047 1.00 0.00

ATOM 3690 HN THR B 192 27.864 -1.150 -5.082 1.00 0.00

ATOM 3691 CA THR B 192 27.326 -2.090 -6.867 1.00 0.00

ATOM 3692 HA THR B 192 27.629 -1.905 -7.904 1.00 0.00

ATOM 3693 C THR B 192 25.788 -2.270 -6.824 1.00 0.00

ATOM 3694 O THR B 192 25.254 -2.907 -5.923 1.00 0.00

ATOM 3695 CB THR B 192 28.167 -3.233 -6.239 1.00 0.00

ATOM 3696 HB THR B 192 27.646 -4.204 -6.317 1.00 0.00

ATOM 3697 OG1 THR B 192 28.435 -2.967 -4.845 1.00 0.00

ATOM 3698 HG1 THR B 192 29.114 -2.284 -4.743 1.00 0.00

ATOM 3699 CG2 THR B 192 29.500 -3.354 -7.007 1.00 0.00

ATOM 3700 HG21 THR B 192 29.888 -2.357 -7.261 1.00 0.00

ATOM 3701 HG22 THR B 192 30.238 -3.878 -6.394 1.00 0.00

ATOM 3702 HG23 THR B 192 29.394 -3.930 -7.939 1.00 0.00

ATOM 3703 N LYS B 193 25.131 -1.632 -7.811 1.00 0.00

ATOM 3704 HN LYS B 193 25.604 -1.092 -8.511 1.00 0.00

ATOM 3705 CA LYS B 193 23.682 -1.717 -7.978 1.00 0.00

ATOM 3706 HA LYS B 193 23.335 -2.581 -7.418 1.00 0.00

ATOM 3707 C LYS B 193 23.453 -1.970 -9.481 1.00 0.00

ATOM 3708 O LYS B 193 24.273 -1.565 -10.286 1.00 0.00

ATOM 3709 CB LYS B 193 23.001 -0.418 -7.484 1.00 0.00

ATOM 3710 HB1 LYS B 193 21.924 -0.497 -7.625 1.00 0.00

ATOM 3711 HB2 LYS B 193 23.324 0.407 -8.120 1.00 0.00

ATOM 3712 CG LYS B 193 23.306 -0.058 -6.015 1.00 0.00

ATOM 3713 HG1 LYS B 193 22.893 0.926 -5.791 1.00 0.00

ATOM 3714 HG2 LYS B 193 24.386 0.035 -5.896 1.00 0.00

ATOM 3715 CD LYS B 193 22.750 -1.097 -5.024 1.00 0.00

ATOM 3716 HD1 LYS B 193 23.107 -2.076 -5.333 1.00 0.00

ATOM 3717 HD2 LYS B 193 21.662 -1.137 -5.091 1.00 0.00

ATOM 3718 CE LYS B 193 23.198 -0.892 -3.569 1.00 0.00

ATOM 3719 HE1 LYS B 193 22.758 0.010 -3.142 1.00 0.00

ATOM 3720 HE2 LYS B 193 24.283 -0.779 -3.537 1.00 0.00

ATOM 3721 NZ LYS B 193 22.853 -2.033 -2.712 1.00 0.00

ATOM 3722 HZ1 LYS B 193 22.549 -2.830 -3.312 1.00 0.00

ATOM 3723 HZ2 LYS B 193 23.696 -2.341 -2.175 1.00 0.00

ATOM 3724 HZ3 LYS B 193 22.079 -1.796 -2.054 1.00 0.00

ATOM 3725 N PRO B 194 22.359 -2.691 -9.844 1.00 0.00

ATOM 3726 CA PRO B 194 22.161 -3.161 -11.218 1.00 0.00

ATOM 3727 HA PRO B 194 23.089 -3.607 -11.590 1.00 0.00

ATOM 3728 C PRO B 194 21.724 -2.076 -12.221 1.00 0.00

ATOM 3729 O PRO B 194 21.119 -1.060 -11.880 1.00 0.00

ATOM 3730 CB PRO B 194 21.077 -4.238 -11.091 1.00 0.00

ATOM 3731 HB1 PRO B 194 21.561 -5.209 -10.970 1.00 0.00

ATOM 3732 HB2 PRO B 194 20.433 -4.302 -11.968 1.00 0.00

ATOM 3733 CG PRO B 194 20.313 -3.896 -9.811 1.00 0.00

ATOM 3734 HG1 PRO B 194 19.861 -4.778 -9.354 1.00 0.00

ATOM 3735 HG2 PRO B 194 19.510 -3.198 -10.045 1.00 0.00

ATOM 3736 CD PRO B 194 21.360 -3.224 -8.919 1.00 0.00

ATOM 3737 HD1 PRO B 194 20.926 -2.443 -8.300 1.00 0.00

ATOM 3738 HD2 PRO B 194 21.833 -3.973 -8.283 1.00 0.00

ATOM 3739 N GLU B 195 22.054 -2.392 -13.471 1.00 0.00

ATOM 3740 HN GLU B 195 22.514 -3.272 -13.604 1.00 0.00

ATOM 3741 CA GLU B 195 21.788 -1.590 -14.648 1.00 0.00

ATOM 3742 HA GLU B 195 22.466 -0.742 -14.581 1.00 0.00

ATOM 3743 C GLU B 195 20.331 -1.082 -14.656 1.00 0.00

ATOM 3744 O GLU B 195 19.399 -1.765 -14.245 1.00 0.00

ATOM 3745 CB GLU B 195 22.131 -2.432 -15.893 1.00 0.00

ATOM 3746 HB1 GLU B 195 21.993 -1.824 -16.790 1.00 0.00

ATOM 3747 HB2 GLU B 195 21.450 -3.283 -15.974 1.00 0.00

ATOM 3748 CG GLU B 195 23.580 -2.968 -15.885 1.00 0.00

ATOM 3749 HG1 GLU B 195 24.256 -2.231 -15.452 1.00 0.00

ATOM 3750 HG2 GLU B 195 23.914 -3.144 -16.909 1.00 0.00

ATOM 3751 CD GLU B 195 23.714 -4.299 -15.118 1.00 0.00

ATOM 3752 OE1 GLU B 195 23.342 -5.334 -15.668 1.00 0.00

ATOM 3753 OE2 GLU B 195 24.137 -4.270 -13.955 1.00 0.00

ATOM 3754 N ARG B 196 20.199 0.179 -15.108 1.00 0.00

ATOM 3755 HN ARG B 196 21.051 0.653 -15.330 1.00 0.00

ATOM 3756 CA ARG B 196 18.914 0.870 -15.225 1.00 0.00

ATOM 3757 HA ARG B 196 19.173 1.760 -15.798 1.00 0.00

ATOM 3758 C ARG B 196 18.356 1.371 -13.874 1.00 0.00

ATOM 3759 O ARG B 196 17.429 2.175 -13.860 1.00 0.00

ATOM 3760 CB ARG B 196 17.857 0.075 -16.027 1.00 0.00

ATOM 3761 HB1 ARG B 196 17.463 -0.758 -15.437 1.00 0.00

ATOM 3762 HB2 ARG B 196 18.339 -0.352 -16.907 1.00 0.00

ATOM 3763 CG ARG B 196 16.690 0.969 -16.475 1.00 0.00

ATOM 3764 HG1 ARG B 196 17.093 1.848 -16.981 1.00 0.00

ATOM 3765 HG2 ARG B 196 16.143 1.326 -15.601 1.00 0.00

ATOM 3766 CD ARG B 196 15.678 0.271 -17.380 1.00 0.00

ATOM 3767 HD1 ARG B 196 15.278 -0.622 -16.890 1.00 0.00

ATOM 3768 HD2 ARG B 196 16.106 0.010 -18.352 1.00 0.00

ATOM 3769 NE ARG B 196 14.566 1.188 -17.611 1.00 0.00

ATOM 3770 HE ARG B 196 14.656 2.086 -17.169 1.00 0.00

ATOM 3771 CZ ARG B 196 13.458 0.796 -18.287 1.00 0.00

ATOM 3772 NH1 ARG B 196 13.392 -0.446 -18.767 1.00 0.00

ATOM 3773 HH11 ARG B 196 14.121 -1.077 -18.484 1.00 0.00

ATOM 3774 HH12 ARG B 196 12.665 -0.797 -19.359 1.00 0.00

ATOM 3775 NH2 ARG B 196 12.448 1.652 -18.474 1.00 0.00

ATOM 3776 HH21 ARG B 196 12.485 2.575 -18.080 1.00 0.00

ATOM 3777 HH22 ARG B 196 11.621 1.392 -18.975 1.00 0.00

ATOM 3778 N LYS B 197 18.976 0.927 -12.762 1.00 0.00

ATOM 3779 HN LYS B 197 19.720 0.257 -12.837 1.00 0.00

ATOM 3780 CA LYS B 197 18.526 1.309 -11.424 1.00 0.00

ATOM 3781 HA LYS B 197 17.434 1.353 -11.451 1.00 0.00

ATOM 3782 C LYS B 197 19.080 2.711 -11.028 1.00 0.00

ATOM 3783 O LYS B 197 19.741 2.877 -10.016 1.00 0.00

ATOM 3784 CB LYS B 197 18.967 0.175 -10.469 1.00 0.00

ATOM 3785 HB1 LYS B 197 20.022 0.272 -10.211 1.00 0.00

ATOM 3786 HB2 LYS B 197 18.872 -0.773 -11.007 1.00 0.00

ATOM 3787 CG LYS B 197 18.123 0.041 -9.198 1.00 0.00

ATOM 3788 HG1 LYS B 197 17.843 -1.005 -9.056 1.00 0.00

ATOM 3789 HG2 LYS B 197 17.190 0.588 -9.334 1.00 0.00

ATOM 3790 CD LYS B 197 18.813 0.520 -7.914 1.00 0.00

ATOM 3791 HD1 LYS B 197 19.224 1.518 -8.062 1.00 0.00

ATOM 3792 HD2 LYS B 197 19.639 -0.140 -7.649 1.00 0.00

ATOM 3793 CE LYS B 197 17.778 0.571 -6.794 1.00 0.00

ATOM 3794 HE1 LYS B 197 17.415 -0.425 -6.523 1.00 0.00

ATOM 3795 HE2 LYS B 197 16.924 1.108 -7.200 1.00 0.00

ATOM 3796 NZ LYS B 197 18.162 1.276 -5.575 1.00 0.00

ATOM 3797 HZ1 LYS B 197 19.112 1.671 -5.561 1.00 0.00

ATOM 3798 HZ2 LYS B 197 17.453 2.018 -5.398 1.00 0.00

ATOM 3799 HZ3 LYS B 197 17.949 0.690 -4.738 1.00 0.00

ATOM 3800 N LEU B 198 18.782 3.694 -11.899 1.00 0.00

ATOM 3801 HN LEU B 198 18.263 3.421 -12.709 1.00 0.00

ATOM 3802 CA LEU B 198 19.017 5.131 -11.738 1.00 0.00

ATOM 3803 HA LEU B 198 18.920 5.522 -12.749 1.00 0.00

ATOM 3804 C LEU B 198 20.398 5.587 -11.227 1.00 0.00

ATOM 3805 O LEU B 198 20.511 6.726 -10.795 1.00 0.00

ATOM 3806 CB LEU B 198 17.946 5.748 -10.818 1.00 0.00

ATOM 3807 HB1 LEU B 198 18.155 6.808 -10.647 1.00 0.00

ATOM 3808 HB2 LEU B 198 18.005 5.266 -9.842 1.00 0.00

ATOM 3809 CG LEU B 198 16.514 5.645 -11.354 1.00 0.00

ATOM 3810 HG LEU B 198 16.297 4.591 -11.535 1.00 0.00

ATOM 3811 CD1 LEU B 198 15.508 6.140 -10.292 1.00 0.00

ATOM 3812 HD11 LEU B 198 15.940 6.214 -9.289 1.00 0.00

ATOM 3813 HD12 LEU B 198 15.123 7.133 -10.527 1.00 0.00

ATOM 3814 HD13 LEU B 198 14.658 5.457 -10.221 1.00 0.00

ATOM 3815 CD2 LEU B 198 16.376 6.401 -12.693 1.00 0.00

ATOM 3816 HD21 LEU B 198 17.303 6.903 -12.974 1.00 0.00

ATOM 3817 HD22 LEU B 198 16.115 5.725 -13.509 1.00 0.00

ATOM 3818 HD23 LEU B 198 15.619 7.183 -12.651 1.00 0.00

ATOM 3819 N SER B 199 21.429 4.723 -11.272 1.00 0.00

ATOM 3820 HN SER B 199 21.319 3.756 -11.505 1.00 0.00

ATOM 3821 CA SER B 199 22.647 5.085 -10.544 1.00 0.00

ATOM 3822 HA SER B 199 22.320 5.178 -9.505 1.00 0.00

ATOM 3823 C SER B 199 23.272 6.454 -10.954 1.00 0.00

ATOM 3824 O SER B 199 23.723 7.200 -10.097 1.00 0.00

ATOM 3825 CB SER B 199 23.614 3.898 -10.623 1.00 0.00

ATOM 3826 HB1 SER B 199 24.499 4.037 -9.989 1.00 0.00

ATOM 3827 HB2 SER B 199 24.009 3.761 -11.632 1.00 0.00

ATOM 3828 OG SER B 199 22.887 2.724 -10.270 1.00 0.00

ATOM 3829 HG SER B 199 22.595 2.768 -9.359 1.00 0.00

ATOM 3830 N TRP B 200 23.244 6.769 -12.274 1.00 0.00

ATOM 3831 HN TRP B 200 22.748 6.185 -12.913 1.00 0.00

ATOM 3832 CA TRP B 200 23.851 8.050 -12.672 1.00 0.00

ATOM 3833 HA TRP B 200 24.723 8.229 -12.037 1.00 0.00

ATOM 3834 C TRP B 200 22.884 9.239 -12.479 1.00 0.00

ATOM 3835 O TRP B 200 23.280 10.379 -12.653 1.00 0.00

ATOM 3836 CB TRP B 200 24.304 8.048 -14.145 1.00 0.00

ATOM 3837 HB1 TRP B 200 24.542 9.069 -14.460 1.00 0.00

ATOM 3838 HB2 TRP B 200 23.509 7.705 -14.804 1.00 0.00

ATOM 3839 CG TRP B 200 25.546 7.212 -14.362 1.00 0.00

ATOM 3840 CD1 TRP B 200 25.603 5.965 -14.991 1.00 0.00

ATOM 3841 HD1 TRP B 200 24.753 5.434 -15.393 1.00 0.00

ATOM 3842 NE1 TRP B 200 26.891 5.548 -15.032 1.00 0.00

ATOM 3843 HE1 TRP B 200 27.199 4.698 -15.401 1.00 0.00

ATOM 3844 CE2 TRP B 200 27.722 6.489 -14.441 1.00 0.00

ATOM 3845 CD2 TRP B 200 26.902 7.558 -14.007 1.00 0.00

ATOM 3846 CE3 TRP B 200 27.461 8.645 -13.384 1.00 0.00

ATOM 3847 HE3 TRP B 200 26.851 9.471 -13.042 1.00 0.00

ATOM 3848 CZ3 TRP B 200 28.841 8.697 -13.191 1.00 0.00

ATOM 3849 HZ3 TRP B 200 29.298 9.552 -12.713 1.00 0.00

ATOM 3850 CH2 TRP B 200 29.646 7.643 -13.624 1.00 0.00

ATOM 3851 HH2 TRP B 200 30.715 7.686 -13.487 1.00 0.00

ATOM 3852 CZ2 TRP B 200 29.075 6.538 -14.250 1.00 0.00

ATOM 3853 HZ2 TRP B 200 29.695 5.716 -14.573 1.00 0.00

ATOM 3854 N LEU B 201 21.609 8.924 -12.185 1.00 0.00

ATOM 3855 HN LEU B 201 21.382 8.033 -11.785 1.00 0.00

ATOM 3856 CA LEU B 201 20.577 9.965 -12.304 1.00 0.00

ATOM 3857 HA LEU B 201 21.015 10.879 -12.717 1.00 0.00

ATOM 3858 C LEU B 201 19.970 10.351 -10.947 1.00 0.00

ATOM 3859 O LEU B 201 19.109 11.225 -10.925 1.00 0.00

ATOM 3860 CB LEU B 201 19.428 9.502 -13.240 1.00 0.00

ATOM 3861 HB1 LEU B 201 18.461 9.772 -12.808 1.00 0.00

ATOM 3862 HB2 LEU B 201 19.406 8.415 -13.320 1.00 0.00

ATOM 3863 CG LEU B 201 19.436 10.163 -14.632 1.00 0.00

ATOM 3864 HG LEU B 201 19.539 11.241 -14.469 1.00 0.00

ATOM 3865 CD1 LEU B 201 20.638 9.720 -15.487 1.00 0.00

ATOM 3866 HD11 LEU B 201 21.320 9.070 -14.939 1.00 0.00

ATOM 3867 HD12 LEU B 201 20.342 9.196 -16.396 1.00 0.00

ATOM 3868 HD13 LEU B 201 21.220 10.587 -15.798 1.00 0.00

ATOM 3869 CD2 LEU B 201 18.082 9.965 -15.355 1.00 0.00

ATOM 3870 HD21 LEU B 201 17.336 9.504 -14.704 1.00 0.00

ATOM 3871 HD22 LEU B 201 17.668 10.927 -15.663 1.00 0.00

ATOM 3872 HD23 LEU B 201 18.161 9.340 -16.245 1.00 0.00

ATOM 3873 N LEU B 202 20.375 9.624 -9.881 1.00 0.00

ATOM 3874 HN LEU B 202 21.096 8.939 -9.994 1.00 0.00

ATOM 3875 CA LEU B 202 19.791 9.895 -8.561 1.00 0.00

ATOM 3876 HA LEU B 202 19.356 10.897 -8.598 1.00 0.00

ATOM 3877 C LEU B 202 20.900 10.043 -7.485 1.00 0.00

ATOM 3878 O LEU B 202 21.180 11.152 -7.057 1.00 0.00

ATOM 3879 CB LEU B 202 18.620 8.916 -8.303 1.00 0.00

ATOM 3880 HB1 LEU B 202 18.951 7.889 -8.203 1.00 0.00

ATOM 3881 HB2 LEU B 202 18.017 8.928 -9.211 1.00 0.00

ATOM 3882 CG LEU B 202 17.691 9.318 -7.139 1.00 0.00

ATOM 3883 HG LEU B 202 17.625 10.408 -7.164 1.00 0.00

ATOM 3884 CD1 LEU B 202 16.252 8.786 -7.332 1.00 0.00

ATOM 3885 HD11 LEU B 202 16.040 8.459 -8.353 1.00 0.00

ATOM 3886 HD12 LEU B 202 16.023 7.948 -6.672 1.00 0.00

ATOM 3887 HD13 LEU B 202 15.538 9.581 -7.106 1.00 0.00

ATOM 3888 CD2 LEU B 202 18.258 8.952 -5.752 1.00 0.00

ATOM 3889 HD21 LEU B 202 19.262 8.533 -5.801 1.00 0.00

ATOM 3890 HD22 LEU B 202 18.348 9.847 -5.132 1.00 0.00

ATOM 3891 HD23 LEU B 202 17.632 8.250 -5.204 1.00 0.00

ATOM 3892 N PRO B 203 21.585 8.925 -7.095 1.00 0.00

ATOM 3893 CA PRO B 203 22.606 9.011 -6.056 1.00 0.00

ATOM 3894 HA PRO B 203 22.051 9.121 -5.122 1.00 0.00

ATOM 3895 C PRO B 203 23.577 10.228 -6.101 1.00 0.00

ATOM 3896 O PRO B 203 23.589 11.020 -5.177 1.00 0.00

ATOM 3897 CB PRO B 203 23.288 7.635 -6.029 1.00 0.00

ATOM 3898 HB1 PRO B 203 23.118 7.185 -5.052 1.00 0.00

ATOM 3899 HB2 PRO B 203 24.367 7.712 -6.168 1.00 0.00

ATOM 3900 CG PRO B 203 22.633 6.805 -7.138 1.00 0.00

ATOM 3901 HG1 PRO B 203 22.412 5.785 -6.831 1.00 0.00

ATOM 3902 HG2 PRO B 203 23.341 6.757 -7.953 1.00 0.00

ATOM 3903 CD PRO B 203 21.382 7.566 -7.572 1.00 0.00

ATOM 3904 HD1 PRO B 203 21.230 7.530 -8.647 1.00 0.00

ATOM 3905 HD2 PRO B 203 20.522 7.142 -7.061 1.00 0.00

ATOM 3906 N PRO B 204 24.374 10.422 -7.188 1.00 0.00

ATOM 3907 CA PRO B 204 25.331 11.540 -7.219 1.00 0.00

ATOM 3908 HA PRO B 204 25.958 11.514 -6.323 1.00 0.00

ATOM 3909 C PRO B 204 24.697 12.950 -7.290 1.00 0.00

ATOM 3910 O PRO B 204 25.394 13.955 -7.357 1.00 0.00

ATOM 3911 CB PRO B 204 26.153 11.276 -8.487 1.00 0.00

ATOM 3912 HB1 PRO B 204 27.047 10.724 -8.204 1.00 0.00

ATOM 3913 HB2 PRO B 204 26.481 12.201 -8.967 1.00 0.00

ATOM 3914 CG PRO B 204 25.250 10.414 -9.373 1.00 0.00

ATOM 3915 HG1 PRO B 204 25.812 9.799 -10.074 1.00 0.00

ATOM 3916 HG2 PRO B 204 24.614 11.089 -9.947 1.00 0.00

ATOM 3917 CD PRO B 204 24.391 9.606 -8.396 1.00 0.00

ATOM 3918 HD1 PRO B 204 23.391 9.457 -8.798 1.00 0.00

ATOM 3919 HD2 PRO B 204 24.850 8.643 -8.174 1.00 0.00

ATOM 3920 N LEU B 205 23.347 12.956 -7.314 1.00 0.00

ATOM 3921 HN LEU B 205 22.824 12.110 -7.233 1.00 0.00

ATOM 3922 CA LEU B 205 22.636 14.210 -7.118 1.00 0.00

ATOM 3923 HA LEU B 205 23.282 15.031 -7.437 1.00 0.00

ATOM 3924 C LEU B 205 22.348 14.391 -5.624 1.00 0.00

ATOM 3925 O LEU B 205 22.282 15.512 -5.146 1.00 0.00

ATOM 3926 CB LEU B 205 21.318 14.263 -7.914 1.00 0.00

ATOM 3927 HB1 LEU B 205 20.650 14.960 -7.407 1.00 0.00

ATOM 3928 HB2 LEU B 205 20.799 13.307 -7.903 1.00 0.00

ATOM 3929 CG LEU B 205 21.485 14.790 -9.347 1.00 0.00

ATOM 3930 HG LEU B 205 22.035 15.728 -9.255 1.00 0.00

ATOM 3931 CD1 LEU B 205 22.328 13.859 -10.242 1.00 0.00

ATOM 3932 HD11 LEU B 205 22.622 12.942 -9.735 1.00 0.00

ATOM 3933 HD12 LEU B 205 21.802 13.560 -11.147 1.00 0.00

ATOM 3934 HD13 LEU B 205 23.244 14.360 -10.551 1.00 0.00

ATOM 3935 CD2 LEU B 205 20.123 15.151 -9.973 1.00 0.00

ATOM 3936 HD21 LEU B 205 19.306 15.078 -9.252 1.00 0.00

ATOM 3937 HD22 LEU B 205 20.126 16.173 -10.351 1.00 0.00

ATOM 3938 HD23 LEU B 205 19.860 14.493 -10.801 1.00 0.00

ATOM 3939 N SER B 206 22.135 13.253 -4.928 1.00 0.00

ATOM 3940 HN SER B 206 22.173 12.365 -5.392 1.00 0.00

ATOM 3941 CA SER B 206 21.976 13.367 -3.480 1.00 0.00

ATOM 3942 HA SER B 206 21.361 14.248 -3.280 1.00 0.00

ATOM 3943 C SER B 206 23.333 13.576 -2.777 1.00 0.00

ATOM 3944 O SER B 206 23.383 14.188 -1.728 1.00 0.00

ATOM 3945 CB SER B 206 21.271 12.128 -2.885 1.00 0.00

ATOM 3946 HB1 SER B 206 21.872 11.227 -3.051 1.00 0.00

ATOM 3947 HB2 SER B 206 20.317 11.964 -3.397 1.00 0.00

ATOM 3948 OG SER B 206 20.974 12.246 -1.495 1.00 0.00

ATOM 3949 HG SER B 206 21.630 12.800 -1.049 1.00 0.00

ATOM 3950 N ASN B 207 24.403 12.971 -3.330 1.00 0.00

ATOM 3951 HN ASN B 207 24.310 12.597 -4.253 1.00 0.00

ATOM 3952 CA ASN B 207 25.656 12.851 -2.561 1.00 0.00

ATOM 3953 HA ASN B 207 25.460 13.009 -1.497 1.00 0.00

ATOM 3954 C ASN B 207 26.659 13.920 -3.052 1.00 0.00

ATOM 3955 O ASN B 207 27.776 13.579 -3.429 1.00 0.00

ATOM 3956 CB ASN B 207 26.307 11.458 -2.759 1.00 0.00

ATOM 3957 HB1 ASN B 207 27.313 11.462 -2.338 1.00 0.00

ATOM 3958 HB2 ASN B 207 26.432 11.277 -3.826 1.00 0.00

ATOM 3959 CG ASN B 207 25.570 10.242 -2.165 1.00 0.00

ATOM 3960 OD1 ASN B 207 24.762 9.604 -2.839 1.00 0.00

ATOM 3961 ND2 ASN B 207 26.023 9.837 -0.968 1.00 0.00

ATOM 3962 HD21 ASN B 207 25.737 8.930 -0.635 1.00 0.00

ATOM 3963 HD22 ASN B 207 26.617 10.415 -0.403 1.00 0.00

ATOM 3964 N ASN B 208 26.185 15.187 -3.085 1.00 0.00

ATOM 3965 HN ASN B 208 25.318 15.468 -2.660 1.00 0.00

ATOM 3966 CA ASN B 208 26.967 16.283 -3.674 1.00 0.00

ATOM 3967 HA ASN B 208 27.985 16.194 -3.289 1.00 0.00

ATOM 3968 C ASN B 208 26.368 17.643 -3.216 1.00 0.00

ATOM 3969 CB ASN B 208 26.948 16.158 -5.220 1.00 0.00

ATOM 3970 HB1 ASN B 208 25.921 16.258 -5.576 1.00 0.00

ATOM 3971 HB2 ASN B 208 27.278 15.163 -5.519 1.00 0.00

ATOM 3972 CG ASN B 208 27.825 17.191 -5.967 1.00 0.00

ATOM 3973 OD1 ASN B 208 28.798 16.879 -6.652 1.00 0.00

ATOM 3974 ND2 ASN B 208 27.366 18.445 -5.900 1.00 0.00

ATOM 3975 HD21 ASN B 208 27.698 19.180 -6.474 1.00 0.00

ATOM 3976 HD22 ASN B 208 26.692 18.610 -5.168 1.00 0.00

ATOM 3977 OCT1 ASN B 208 27.114 18.629 -3.126 1.00 0.00

ATOM 3978 OCT2 ASN B 208 25.158 17.717 -2.993 1.00 0.00

TER 3978 ASN B 208

ATOM 3979 OG NDL N 1 -11.101 -4.184 6.967 1.00 0.00

ATOM 3980 O2 NDL N 1 -14.931 0.950 12.398 1.00 0.00

ATOM 3981 C1 NDL N 1 -16.151 0.853 12.336 1.00 0.00

ATOM 3982 N9 NDL N 1 -16.935 1.940 12.312 1.00 0.00

ATOM 3983 H9 NDL N 1 -17.924 1.829 12.376 1.00 0.00

ATOM 3984 C10 NDL N 1 -16.378 3.265 12.129 1.00 0.00

ATOM 3985 H10 NDL N 1 -17.249 3.921 12.125 1.00 0.00

ATOM 3986 C12 NDL N 1 -15.466 3.702 13.298 1.00 0.00

ATOM 3987 H121 NDL N 1 -15.085 4.698 13.092 1.00 0.00

ATOM 3988 H122 NDL N 1 -14.603 3.042 13.370 1.00 0.00

ATOM 3989 C11 NDL N 1 -16.190 3.731 14.654 1.00 0.00

ATOM 3990 H111 NDL N 1 -16.588 2.736 14.859 1.00 0.00

ATOM 3991 H112 NDL N 1 -17.046 4.401 14.600 1.00 0.00

ATOM 3992 C14 NDL N 1 -15.289 4.141 15.829 1.00 0.00

ATOM 3993 H141 NDL N 1 -14.418 3.486 15.838 1.00 0.00

ATOM 3994 H142 NDL N 1 -15.827 3.961 16.759 1.00 0.00

ATOM 3995 C13 NDL N 1 -14.844 5.606 15.783 1.00 0.00

ATOM 3996 H131 NDL N 1 -14.203 5.854 16.628 1.00 0.00

ATOM 3997 H132 NDL N 1 -14.281 5.800 14.872 1.00 0.00

ATOM 3998 H133 NDL N 1 -15.702 6.276 15.803 1.00 0.00

ATOM 3999 C15 NDL N 1 -15.781 3.372 10.709 1.00 0.00

ATOM 4000 O16 NDL N 1 -16.521 3.306 9.733 1.00 0.00

ATOM 4001 N17 NDL N 1 -14.467 3.599 10.599 1.00 0.00

ATOM 4002 H17 NDL N 1 -13.908 3.142 11.292 1.00 0.00

ATOM 4003 C18 NDL N 1 -14.000 4.303 9.396 1.00 0.00

ATOM 4004 H18 NDL N 1 -14.856 4.667 8.823 1.00 0.00

ATOM 4005 C19 NDL N 1 -13.192 5.528 9.783 1.00 0.00

ATOM 4006 H191 NDL N 1 -12.831 6.021 8.877 1.00 0.00

ATOM 4007 H192 NDL N 1 -12.310 5.224 10.349 1.00 0.00

ATOM 4008 C20 NDL N 1 -14.040 6.513 10.587 1.00 0.00

ATOM 4009 H201 NDL N 1 -14.437 6.011 11.466 1.00 0.00

ATOM 4010 H202 NDL N 1 -14.897 6.843 9.999 1.00 0.00

ATOM 4011 C21 NDL N 1 -13.194 7.700 11.011 1.00 0.00

ATOM 4012 H211 NDL N 1 -13.024 8.357 10.158 1.00 0.00

ATOM 4013 H212 NDL N 1 -12.222 7.300 11.304 1.00 0.00

ATOM 4014 C22 NDL N 1 -13.801 8.439 12.198 1.00 0.00

ATOM 4015 H221 NDL N 1 -14.259 7.726 12.890 1.00 0.00

ATOM 4016 H222 NDL N 1 -14.568 9.158 11.897 1.00 0.00

ATOM 4017 N23 NDL N 1 -12.730 9.112 12.923 1.00 0.00

ATOM 4018 H231 NDL N 1 -13.102 9.488 13.814 1.00 0.00

ATOM 4019 H232 NDL N 1 -11.989 8.411 13.131 1.00 0.00

ATOM 4020 H233 NDL N 1 -12.309 9.864 12.346 1.00 0.00

ATOM 4021 C24 NDL N 1 -13.204 3.445 8.423 1.00 0.00

ATOM 4022 O25 NDL N 1 -13.279 3.673 7.232 1.00 0.00

ATOM 4023 N26 NDL N 1 -12.418 2.492 8.925 1.00 0.00

ATOM 4024 H26 NDL N 1 -12.412 2.215 9.883 1.00 0.00

ATOM 4025 C27 NDL N 1 -11.689 1.610 8.015 1.00 0.00

ATOM 4026 H27 NDL N 1 -11.958 1.825 6.985 1.00 0.00

ATOM 4027 C28 NDL N 1 -10.187 1.780 8.195 1.00 0.00

ATOM 4028 H281 NDL N 1 -9.656 0.898 7.834 1.00 0.00

ATOM 4029 H282 NDL N 1 -9.965 1.854 9.260 1.00 0.00

ATOM 4030 C29 NDL N 1 -9.667 2.992 7.418 1.00 0.00

ATOM 4031 H291 NDL N 1 -10.483 3.648 7.110 1.00 0.00

ATOM 4032 H292 NDL N 1 -9.163 2.655 6.510 1.00 0.00

ATOM 4033 C30 NDL N 1 -8.700 3.791 8.260 1.00 0.00

ATOM 4034 H301 NDL N 1 -8.002 3.142 8.791 1.00 0.00

ATOM 4035 H302 NDL N 1 -9.270 4.384 8.981 1.00 0.00

ATOM 4036 N31 NDL N 1 -7.911 4.679 7.418 1.00 0.00

ATOM 4037 H31 NDL N 1 -7.863 4.552 6.419 1.00 0.00

ATOM 4038 C32 NDL N 1 -7.226 5.668 8.109 1.00 0.00

ATOM 4039 N33 NDL N 1 -7.182 5.764 9.448 1.00 0.00

ATOM 4040 H331 NDL N 1 -6.610 6.512 9.827 1.00 0.00

ATOM 4041 H332 NDL N 1 -7.429 4.995 10.048 1.00 0.00

ATOM 4042 N34 NDL N 1 -6.442 6.518 7.442 1.00 0.00

ATOM 4043 H341 NDL N 1 -5.741 7.093 7.874 1.00 0.00

ATOM 4044 H342 NDL N 1 -6.145 6.248 6.503 1.00 0.00

ATOM 4045 C35 NDL N 1 -12.146 0.209 8.348 1.00 0.00

ATOM 4046 O36 NDL N 1 -12.912 0.036 9.294 1.00 0.00

ATOM 4047 N37 NDL N 1 -11.682 -0.746 7.521 1.00 0.00

ATOM 4048 H37 NDL N 1 -10.955 -0.514 6.873 1.00 0.00

ATOM 4049 C38 NDL N 1 -12.111 -2.128 7.600 1.00 0.00

ATOM 4050 H38 NDL N 1 -12.584 -2.306 8.567 1.00 0.00

ATOM 4051 C46 NDL N 1 -10.846 -2.999 7.538 1.00 0.00

ATOM 4052 O47 NDL N 1 -10.084 -2.982 8.501 1.00 0.00

ATOM 4053 C39 NDL N 1 -13.116 -2.386 6.460 1.00 0.00

ATOM 4054 H391 NDL N 1 -13.382 -3.444 6.441 1.00 0.00

ATOM 4055 H392 NDL N 1 -12.653 -2.155 5.498 1.00 0.00

ATOM 4056 C40 NDL N 1 -14.409 -1.568 6.601 1.00 0.00

ATOM 4057 H401 NDL N 1 -15.082 -1.841 5.788 1.00 0.00

ATOM 4058 H402 NDL N 1 -14.195 -0.507 6.472 1.00 0.00

ATOM 4059 C41 NDL N 1 -15.138 -1.819 7.931 1.00 0.00

ATOM 4060 H411 NDL N 1 -14.745 -1.236 8.759 1.00 0.00

ATOM 4061 H412 NDL N 1 -15.065 -2.871 8.211 1.00 0.00

ATOM 4062 N42 NDL N 1 -16.556 -1.534 7.768 1.00 0.00

ATOM 4063 H42 NDL N 1 -17.026 -2.286 7.304 1.00 0.00

ATOM 4064 C43 NDL N 1 -17.128 -0.281 7.966 1.00 0.00

ATOM 4065 N44 NDL N 1 -16.445 0.692 8.592 1.00 0.00

ATOM 4066 H441 NDL N 1 -16.746 1.651 8.637 1.00 0.00

ATOM 4067 H442 NDL N 1 -15.497 0.476 8.877 1.00 0.00

ATOM 4068 N45 NDL N 1 -18.354 -0.019 7.489 1.00 0.00

ATOM 4069 H451 NDL N 1 -18.842 0.840 7.635 1.00 0.00

ATOM 4070 H452 NDL N 1 -18.797 -0.615 6.784 1.00 0.00

ATOM 4071 C1B NDL N 1 -16.814 -2.714 13.176 1.00 0.00

ATOM 4072 H1B NDL N 1 -16.288 -3.553 13.613 1.00 0.00

ATOM 4073 C2B NDL N 1 -18.148 -2.853 12.825 1.00 0.00

ATOM 4074 H2B NDL N 1 -18.635 -3.808 12.967 1.00 0.00

ATOM 4075 C3B NDL N 1 -18.845 -1.779 12.291 1.00 0.00

ATOM 4076 H3B NDL N 1 -19.890 -1.892 12.022 1.00 0.00

ATOM 4077 C4B NDL N 1 -18.210 -0.556 12.125 1.00 0.00

ATOM 4078 H4B NDL N 1 -18.789 0.265 11.728 1.00 0.00

ATOM 4079 C5B NDL N 1 -16.865 -0.416 12.474 1.00 0.00

ATOM 4080 C6B NDL N 1 -16.168 -1.503 12.979 1.00 0.00

ATOM 4081 H6B NDL N 1 -15.122 -1.399 13.243 1.00 0.00

TER 4081 NDL N 1

END
